# ToxCodAn-Genome: an automated pipeline for toxin-gene annotation in genome assembly of venomous lineages

**DOI:** 10.1093/gigascience/giad116

**Published:** 2024-01-18

**Authors:** Pedro G Nachtigall, Alan M Durham, Darin R Rokyta, Inácio L M Junqueira-de-Azevedo

**Affiliations:** Laboratório de Toxinologia Aplicada, CeTICS, Instituto Butantan, São Paulo, 05503-900 SP, Brazil; Department of Biological Science, Florida State University, Tallahassee, 32306-4295 FL, USA; Departamento de Ciência da Computação, Instituto de Matemática e Estatística, Universidade de São Paulo (USP), São Paulo, 05508-090 SP, Brazil; Department of Biological Science, Florida State University, Tallahassee, 32306-4295 FL, USA; Laboratório de Toxinologia Aplicada, CeTICS, Instituto Butantan, São Paulo, 05503-900 SP, Brazil

**Keywords:** WGS, DNA-seq, genome annotation, gene model, venomics

## Abstract

**Background:**

The rapid development of sequencing technologies resulted in a wide expansion of genomics studies using venomous lineages. This facilitated research focusing on understanding the evolution of adaptive traits and the search for novel compounds that can be applied in agriculture and medicine. However, the toxin annotation of genomes is a laborious and time-consuming task, and no consensus pipeline is currently available. No computational tool currently exists to address the challenges specific to toxin annotation and to ensure the reproducibility of the process.

**Results:**

Here, we present ToxCodAn-Genome, the first software designed to perform automated toxin annotation in genomes of venomous lineages. This pipeline was designed to retrieve the full-length coding sequences of toxins and to allow the detection of novel truncated paralogs and pseudogenes. We tested ToxCodAn-Genome using 12 genomes of venomous lineages and achieved high performance on recovering their current toxin annotations. This tool can be easily customized to allow improvements in the final toxin annotation set and can be expanded to virtually any venomous lineage. ToxCodAn-Genome is fast, allowing it to run on any personal computer, but it can also be executed in multicore mode, taking advantage of large high-performance servers. In addition, we provide a guide to direct future research in the venomics field to ensure a confident toxin annotation in the genome being studied. As a case study, we sequenced and annotated the toxin repertoire of *Bothrops alternatus*, which may facilitate future evolutionary and biomedical studies using vipers as models.

**Conclusions:**

ToxCodAn-Genome is suitable to perform toxin annotation in the genome of venomous species and may help to improve the reproducibility of further studies. ToxCodAn-Genome and the guide are freely available at https://github.com/pedronachtigall/ToxCodAn-Genome.

Key Points

- We present ToxCodAn-Genome, the first automated computational pipeline designed specifically for toxin-gene annotation in genome assemblies of venomous species.
- The analysis using 12 available genomes from snakes, stingrays, scorpions, Hymenoptera, and Anthozoa showed that ToxCodAn-Genome is suitable for use on any venomous species.
- The proof-of-concept test showed that ToxCodAn-Genome can annotate most of the toxins in the genome, which integrates the set of highly expressed toxins in the venom–tissue transcriptome.
- ToxCodAn-Genome is fast, is accurate, and can be used on any personal computer or taking advantage of supercomputers.
- Our case study based on sequencing the genome of *Bothrops alternatus* revealed that ToxCodAn-Genome and our guide can be applied to understand the genomic context and evolution of venom genes.
- The draft genome of *B. alternatus* allowed the recovery of the first complete SVMP loci in lancehead vipers.

## Introduction

Over the past 2 decades, the rapid development of sequencing technologies, which includes wet- and dry-bench protocols, has decreased the cost and time to generate high-quality genome assemblies (reviewed in [1]). This resulted in a wide expansion of the number of species in the Tree of Life with a sequenced genome [2]. In particular, the genome sequencing of venomous lineages has become an useful approach to search for novel toxin compounds, which may help in the development of new medicines (reviewed in [3]), ensure the production of effective antivenoms [4], elucidate the genetic regulatory mechanisms related to complex phenotypes [5–7], and understand the evolutionary history of adaptive traits [8].

Venoms, along with their production and injection apparatus, have evolved independently more than 100 times in diverse lineages throughout the Tree of Life (reviewed in [9]). They are composed of a complex cocktail of proteins and peptides (also known as toxins) and are mainly used for prey capture and defense against predators but may also be used in intraspecific competition [8, 10, 11]. The toxin composition of venom is a polygenic trait, frequently evolving under strong selection, and represents a key adaptive innovation [11]. Moreover, venoms and their toxins are excellent model systems to trace the impact of gene sequence mutations over protein function, as the majority of proteinaceous toxins are adapted to specific functions when injected into their targets [10, 12]. The biological effects of toxins and their remarkable target specificity are of high interest to the research community due to their potential in the fields of pharmacology, medicine, biotechnology, and agrochemistry [3, 4, 13, 14]. Sequencing the genomes of venomous lineages and deciphering their toxin repertoire within the genomic context, therefore, represents an outstanding opportunity across diverse research fields.

Despite the relevance of genomic studies to venomics, only a small percentage of venomous species have had their genomes sequenced and used to understand the genomic context of their toxin repertoire (reviewed in [9]). Of these, snakes represent the venomous clade with the most representatives studied, which has revealed some remarkable features in the evolution and novelty of venom systems [15–21]. However, many other snakes and venomous species are being studied, and their genomes are being widely sequenced to generate a high-quality assembly. In this context, a tool for performing fast and accurate toxin annotation will help to improve our knowledge of the biological roles and track the evolutionary history of venom and its toxic compounds.

Genome annotation is an important step for many biological studies, because it helps to decipher the biological pathways that lead to specific phenotypes [22]. Characterizing genes in bacterial genomes is relatively easy, because most of their genes do not present exon–intron structures and have short intergenic regions [23]. On the other hand, characterizing genes in eukaryotes is far more complex, because the genes are sparse in the genome (i.e., there are long intergenic regions) and the genes are structured into an exon–intron context. Thus, the precise identification of exon–intron boundaries and exact localization of genes are not easily determined. These features make the annotation of eukaryotic genomes error-prone by nature and require development of suitable tools to help mitigate erroneous annotations [24].

Currently, several tools exist to perform gene annotation in the genomes of eukaryotic species (reviewed in [25]). These tools comprise distinct strategies that may range from *ab initio* prediction using pretrained models to self-training algorithms to similarity search. The *ab initio* prediction tools, such as AUGUSTUS [26] and SNAP [27], search for genes based on a generic gene model, but they may also integrate protein and transcript sequences as evidence to validate the predicted genes. Some tools, like BRAKER [28, 29], MAKER [30], GeneMark−ES [31], and AUGUSTUS, can perform self-training of gene models specific to the genome being analyzed by using the outputs of preliminary runs to improve the performance of gene prediction on subsequent runs. These approaches may also integrate alignments of proteins and transcripts to use as evidence in the gene-prediction process. Other tools rely on using pretrained species-specific models that can be integrated with protein evidence of closely related species, such as FGENESH+ [32]. These tools have been used in several published genomes, but they are dependent on powerful computing resources. This feature may result in a slow running time that may take up to a few weeks when insufficient computing resources are available. Other applications, such as GEMOMA [33], Liftoff [34], and TOGA [35], rely on the use of similarity searches using a high-quality and well-annotated genome of a closely related species as a reference. These tools consider genome alignment and the homology and orthology inference of genes to build gene models and/or transfer annotation to the target species. However, in nonmodel organisms and less studied groups, a well-annotated genome from a closely related species is commonly not available. Moreover, if the genome used as reference is not well annotated and contains erroneous and incomplete annotations, these may be propagated to the target genome [36]. Independently of the strategy adopted, it is known that automated genome annotation tools do not accurately characterize complex gene families [37, 38], which requires laborious manual curation for a reliable and comprehensive annotation of a genome [39]. Therefore, the genome annotation task is a puzzle not easily solved that can benefit from improvements for specific cases [24].

Despite the plethora of available tools to perform an automated annotation of genomes, none of them were designed to solve the issues specific to the toxin annotation task [40]. The annotation of toxin genes presents some tricky issues when compared to general gene annotation in eukaryotes that may lead to incorrect or incomplete identification of gene structure and wrong assignment of gene names. These errors may result from the following features not adopted by the general annotation tools: (i) toxin repertoire is highly diverse among venomous taxa, which leads to difficulties in setting a reliable “toxin” feature to assist in toxin gene identification; (ii) toxin genes may share high similarity with their ancestral gene [19], which make it difficult to distinguish toxin genes from related nontoxin genes; (iii) toxin genes may have originated from *in locus* duplication of an ancestral nontoxin gene [19, 41, 42]; (iv) the duplicated toxin genes are commonly arranged in tandem arrays and can be highly similar [19, 43–45]; (v) the genomic regions of these highly duplicated toxin genes are marked by the presence of orphan exons and pseudogenes (e.g., commonly observed in metalloproteinases, serine proteases, phospholipases, and 3-finger toxins loci of snake genomes), which complicates the correct annotation of these regions [21, 41, 42, 45–47]; (vi) the high mutation rate of these toxin loci may also result in truncated paralogs, which may present a complete gene structure with a premature stop codon [45, 48]; and (vii) the toxin loci can present high levels of genomic rearrangements [41, 42, 45]. All these features together introduce extra layers of complexity when annotating toxin genes in genomes. In fact, the genomes of venomous lineages published so far revealed that general annotation tools do not perform well in correctly characterizing toxin genes in genomes, which must be further checked using several distinct strategies and approaches that are not easily reproducible, require strong programming skills, and are laborious and time-consuming [15–17, 19, 21, 43–45, 48, 49]. In this sense, the development of a tool able to quickly characterize the toxin repertoire in the genome of venomous lineages will help to minimize efforts in checking toxin annotations, mitigate the effects of erroneous annotations, and improve the reproducibility of analyses.

Here, we present ToxCodAn-Genome, an automated computational pipeline to annotate toxin loci in genomes of virtually any venomous lineage. Using genomic data from snakes, stingrays, scorpions, Hymenoptera, and Anthozoa species, we show that ToxCodAn-Genome has high performance and can be used to annotate toxin genes in different lineages. In fact, it can be easily configured to use on any venomous lineage by designing specific toxin databases and/or using venom–tissue transcriptomic data. To facilitate the use of ToxCodAn-Genome and help in future venomics research, we also provide an extended guide to perform toxin annotation of genomes. Finally, we sequenced and assembled the genome of *Bothrops alternatus* and annotated its toxin gene repertoire as a case study.

## Materials and Methods

### Software implementation

**Figure 1:**
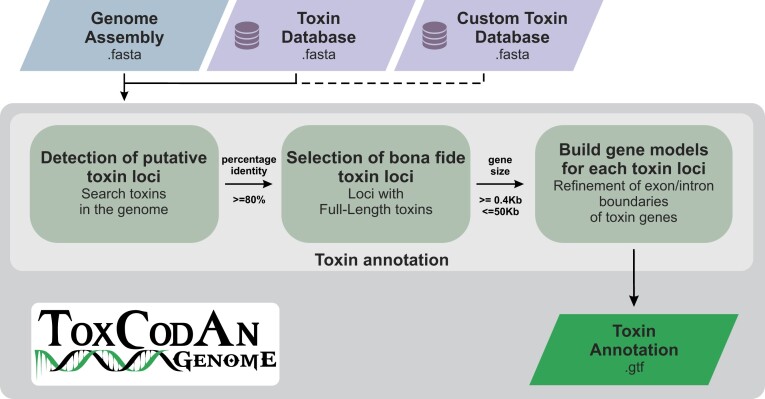
Flowchart of the ToxCodAn-Genome pipeline. The genome assembly is searched to detect putative toxin loci using a toxin database containing full-length toxin CDSs from several species. Putative toxin loci are analyzed to select bona fide toxin loci that are used to build gene models specific to that toxin loci and output the final toxin annotation.

ToxCodAn-Genome was developed using Python (v3.6) and third-party tools to perform the automated analysis (Fig. 1). The pipeline consists of a step to detect putative toxin loci in the genome using a comprehensive toxin database, followed by a step to select bona fide toxin loci that are used to build gene models specific to each toxin locus and generate the toxin annotation file. Specifically, the “detection of putative toxin loci” step performs a similarity search using BLAST (v2.9 or higher) against toxin coding sequences (CDS) present in the toxin database (toxinDB; see “Toxin Databases” section). Then, all putative toxin loci are analyzed in the “selection of bona fide toxin loci” step, which consists of keeping matching regions containing only full-length toxin CDSs for the next step (i.e., matching regions with partial toxin CDSs are not considered for building gene models). The “build gene models for each toxin loci” step uses the putative toxin loci containing full-length toxin CDSs to build gene models using Exonerate (v2.4.0 or higher; [50]), which performs refinement of the intron/exon boundaries and generates the annotation file in GTF format containing the CDSs of the identified toxin loci.

ToxCodAn-Genome can also use a user-designed toxin database to complement any of the provided toxin databases. The custom toxin database can help improve annotations with the inclusion of more specific data from public or private databases, published manuscripts, and/or the user’s own unpublished data. In particular, it can be built using venom–tissue transcriptomic data specific to the lineage/species being studied. The venom–tissue transcriptome can be analyzed using tools designed specifically for this task, such as ToxCodAn [51] and/or Venomix [52]. However, to help users analyze the transcriptomic data, we implemented 2 scripts to assemble venom–tissue transcripts and identify their toxin CDSs (Supplementary Fig. S1 in Supplementary File 1). The script to assemble transcripts (named “TRassembly.py”) performs 4 assemblies considering genome-guided and *de novo* strategies to ensure the recovery of most toxin transcripts [53]. The genome-guided strategy uses Hisat2 (v2.2.1; [54]) to map reads against the genome and use the mapped read information to recover transcripts using StringTie (v2.2.1; [55]) and the genome-guided mode of Trinity (v2.8.5; [56]). The *de novo* strategy performs 2 *de novo* transcriptome assemblies using the *de novo* mode of Trinity and rnaSPAdes (v3.15.5; [57]). Then, all 4 assemblies are concatenated to generate the final set of transcripts to be used in the toxin screening step. The script designed to identify toxin CDSs in the assembled transcripts (named “CDSscreening.py”) performs a BLAST search against a ToxinDB and identifies the full-length toxin CDSs. Both additional scripts can be run independently by the user or set to run directly within the main ToxCodAn-Genome pipeline.

By default, ToxCodAn-Genome outputs the toxin annotation file in GTF format, the CDS and peptide sequences in a FASTA format, and a file with “warning” annotations in TXT format, which contains information about annotations that may represent truncated isoforms, pseudogenes, or novelties that need further inspection. It also generates an annotation file containing all genomic regions matching full-length toxin CDSs in GTF format to be further inspected as needed by the user.

### Guide to annotate toxins in genomes

To complement ToxCodAn-Genome, we produced a detailed guide for toxin annotation. Specifically, we provide the command-line code and links to useful resources to learn basic bioinformatics, to build a toxin database from public resources and/or using a venom–tissue transcriptomic data, to perform toxin and nontoxin annotation, to perform inspection of specific annotations when needed, and to perform quantification of annotated genes using transcriptomic data. We also provide an R script containing useful functions for plotting the toxin loci annotated through our pipeline. Our guide is available in Markdown format on our ToxCodAn-Genome GitHub repository [58] and in an archived PDF format in Supplementary File 2.

### Toxin databases

We built ToxinDB using sequences from species of the widely studied venomous clades of Viperidae, Elapidae, Myliobatoidea, Scorpiones, Hymenoptera, and Anthozoa. To build the ToxinDB, we retrieved full-length toxin CDSs from the nucleotide archive and the TSA databases of NCBI [59]. The full-length toxin CDSs of each lineage were clusterized with 99% similarity using cd-hit (v4.8.1; [60]) to reduce redundancy and generate a final toxin database for each lineage. The Viperidae database was composed of 1,546 toxin CDSs from 108 species that clustered into a total of 1,278 toxin CDSs. The Elapidae database was composed of 1,592 toxin CDSs from 76 species that clustered into a total of 1,150 toxin CDSs. The Myliobatoidei database was composed of 254 toxin CDSs from 7 species that clustered into a total of 192 toxin CDSs. The Scorpiones database was composed of 1,879 toxin CDSs from 39 species that clustered into a total of 1,122 toxin CDSs. The Hymenoptera database was composed of 432 toxin CDSs from 52 species that clustered into a total of 397 toxin CDSs. The Anthozoa database was composed of 1,506 toxin CDSs from 29 species that clustered into a total of 980 toxin CDSs. Of note, the classification of toxins and toxin-like components can vary from one study to another and from one lineage to another. Despite some rational nomenclature that has been proposed for spiders to be extended to other lineages [61], the current studies seem to not follow such standards. In this sense, we used the VenomZone resource [62] and published manuscripts to track down the toxin genes within each lineage studied in the present study. We strongly recommend the users of ToxCodAn-Genome to review the literature about venom components and databases such as VenomZone, ToxProt [63], and other resources [64, 65] to make a better choice of toxin annotation classes for the target lineage.

### Testing sets

To test the performance of ToxCodAn-Genome, we downloaded genomes of 3 Viperidae, 3 Elapidae, 1 Myliobatoidea, 1 Scorpiones, 3 Hymenoptera, and 1 Anthozoa species previously published along with the descriptions of their toxin gene repertoire (Table 1; Supplementary Table S1 in Supplementary File 3). The number of toxin genes in each species was considered as reported by the original publication, except for *Potamotrygon leopoldi*, where no toxin annotations were reported and the number of toxin genes was considered based on its venom–tissue transcriptome report [66], and for *Nematostella vectensis*, where the toxin annotations were considered as annotated in a recent venomics study of the Anthozoa lineage [45]. Then, we compared the total number of toxins identified by ToxCodAn-Genome to the number reported in the previously published annotations. The ToxCodAn-Genome’s annotations were characterized as “reliable” or “warning” based on the output files (see Supplementary Fig. S2 in Supplementary File 1 for more details). Briefly, the “reliable” annotations represent bona fide toxin annotations with a well-defined exon–intron structure and a full-length toxin CDS, whereas the “warning” annotations represents an annotation corresponding to a full-length toxin CDS, with a well-defined exon–intron structure but with a premature stop codon. The “warning” annotations may represent truncated paralogs, pseudogenes, or an erroneous annotation or may be a result of errors in the genomic region assembly.

**Table 1:** Genomic and transcriptomic data used to test the ToxCodAn-Genome

Lineage	Species	Genome	Venom–tissue RNA-seq (SRA)	Reference
Viperidae	*Azemiops feae*	GCA_023970755.1	SRR18397788	[20]
	*Bothrops jararaca*	GCA_018340635.1	SRR13839799	[19]
	*Crotalus tigris*	GCA_016545835.1	SRR11545022	[48]
Elapidae	*Hydrophis curtus*	PRJNA597425	SRR11659669	[18]
	*Hydrophis cyanocinctus*	JAAZTL000000000	SRR11659657	[67]
	*Naja naja*	GCA_009733165.1	SRR8754977	[68]
Myliobatoidei	*Potamotrygon leopoldi*	GWH:GWHAOTN00000000	SRR11049204	[69]
Scorpiones	*Mesobuthus martensii*	GCA_000484575.1	SRR2592960	[43]
Hymenoptera	*Apis cerana*	GCF_001442555.1	SRR1406762	[70]
	*Apis mellifera*	GCF_003254395.2	SRR13213116	[71]
	*Nasonia vitripennis*	GCF_009193385.2	SRR3046453	[72]
Anthozoa	*Nematostella vectensis*	GCF_932526225.1	SRR11600272	[73]

To assess the quality of ToxCodAn-Genome’s annotation, we computed the toxin recovery rate (TRR) for each major toxin family in the target testing set. The TRR is computed by dividing the number of ToxCodAn-Genome’s annotations by the number of toxin loci reported in the original genome annotation for each toxin family. A TRR value equal to 1 indicates an exact match between the number of toxin loci annotated and the number described in the original report. A TRR higher than 1 indicates that ToxCodAn-Genome detected more toxin loci than that originally reported. A TRR lower than 1 indicates that ToxCodAn-Genome detected fewer toxin loci than that originally reported.

To check the effects of database and transcriptomic data on ToxCodAn-Genome’s performance, we set 3 distinct scenarios: (i) using only the toxin database (DB), (ii) integrating the toxin database and venom–tissue transcriptomic data (DBTR), and (3) using only the toxin-annotated venom–tissue transcriptome (TR). Of note, to ensure a fair performance analysis, we removed the species-specific toxin CDSs of the target species from the toxinDB to perform the tests and annotate the venom–tissue transcriptomes.

### Proof-of-concept test

To evaluate the capability of ToxCodAn-Genome to perform a reliable toxin annotation in a more controlled way, we performed a proof-of-concept test by using the 3 Viperidae species from the initial testing set (Table 1). In this test, we specified their available and published toxin CDSs as the only database source to annotate their genomes and ran ToxCodAn-Genome with default parameters. We compared the published annotations and ToxCodAn-Genome’s output available in 2 files: “toxin_annotation.gtf” and “matched_regions.gtf.” The “toxin_annotation.gtf” contains the final gene model for each toxin and reveals if ToxCodAn-Genome was able to correctly annotate or not that specific toxin (i.e., toxins were labeled as “annotated” or “not annotated”). As an independent measure from the correct annotation, we also analyzed the “matched_regions.gtf” to check when ToxCodAn-Genome considered a toxin as a putative toxin locus (i.e., when identified their full-length toxin match in the genome). Here, we labeled each toxin as “matched and annotated” (i.e., when the toxin was also correctly annotated in the “toxin_annotation.gtf” file), “matched but not annotated” (i.e., when the toxin was not annotated in the “toxin_annotation.gtf” file but has a match in their correct genomic position), or “not matched” (i.e., when the toxin was not detected as a putative toxin locus). Additionally, we estimated the expression level of toxins using their venom–tissue transcriptomic data, their toxin CDSs, and RSEM (setting the mismatch rate parameter to 2%; v1.3.1; [74]) to measure when ToxCodAn-Genome was able to correctly annotate highly and/or lowly expressed toxin genes.

### Running time analysis

To assess the running time of ToxCodAn-Genome, we used the *Crotalus tigris* genome (genome size of 1.6 Gb) with the Viperidae toxinDB (containing 1,278 toxin CDSs) and its assembled venom gland transcriptome (total of 257,734 transcripts). We performed the analyses using a personal computer (Intel 6-Core i7 with 16 Gb memory) and set the number of threads to 6 (“-c 6”). Of note, we only considered the running times to generate the custom toxin database using the venom–tissue transcriptome assembly (i.e., running time of “CDSscreening.py”) and to perform the genome annotation (i.e., running time of ToxCodAn-Genome), because the running times of assembling transcripts is solely related to the processing times needed by each third-party tool used in the “transcriptome assembly” module (i.e., Hisat2, StringTie, Trinity, and rnaSPAdes).

### *Bothrops alternatus* case study

As a case study, we sequenced, assembled, and annotated the toxin repertoire of the genome of the urutu lancehead (Viperidae: *B. alternatus*). The urutu lancehead is a large pit viper, with an average size of 754.5 mm, and it is considered a dietary specialist, feeding almost exclusively on mammals [75]. Its geographical distribution ranges from northern Argentina to the South/Central Brazil, Paraguay, and Uruguay [76]. Although the venom of *B. alternatus* has been broadly investigated through transcriptomics [51, 77, 78], its genomic background has yet to be determined.

Here, we briefly describe each step of data analysis, but a detailed description can be found in our guide ([58]; Supplementary File 2).

#### Blood sampling and DNA extraction

One specimen (SB0060) was collected in September 2017 in Mato Grosso do Sul state, Brazil. Blood was extracted from the caudal vein, transferred to a tube containing 100% ethanol solution, and stored at −80°C until use. High-molecular-weight (HMW) genomic DNA (gDNA) was extracted by using a pipette-free protocol as previously described [48]. The snake was handled and collected under Protocol Number 4479020217 from the Ethics Committee on Animal Use of the Butantan Institute (CEUAIB).

The transcriptomic data of venom gland from the same individual used for genome sequencing were obtained as previously described [51], and they are available at the SRA database in the NCBI (access number SRR13153633).

#### Genome sequencing and assembling

HMW DNA was used to construct the PacBio HiFi sequencing libraries with the SMRTbell Express Template Prep Kit 2.0 following the manufacturer’s protocol. Sequencing was performed with 2 cells on the PacBio Sequel II system in CCS mode at the University of Delaware Sequencing and Genotyping Center. Two cells were sequenced and resulted in 3,446,639 reads (total of 48,922,414,841 bp, >28-fold coverage ,and an average read size of 14.2 Kb).

The PacBio HiFi reads were assembled using hifiasm (v0.16.1; [79]) and polished using Inspector (v1.0.1; [80]). The genome assembly statistics were obtained within Inspector, and the completeness was assessed using BUSCO (v5.2.2; [81]) with the tetrapoda gene set (odb10; total of 5,310 genes). We annotated repetitive elements using the EDTA pipeline (v2.0.0; [82]).

#### Genome annotation

For toxin annotation, we used ToxCodAn-Genome with default parameters, the venom gland transcriptome assembly, and the Viperidae database. We then inspected the annotated toxins as follows: (i) compared the toxin CDSs to the toxins annotated in previous transcriptomic studies of *B. alternatus* [51] and other *Bothrops* species (i.e., *Bothrops cotiara, Bothrops fonsecai, Bothrops jararaca*, and *Bothrops jararacussu*; [19, 83, 84]), to ensure it represents confident toxin annotations; (ii) checked the annotated toxins present in the output “annotation_warning.txt,” which may represent truncated paralogs, pseudogenes, or erroneous annotations, and to confirm its gene model, we reannotated it using the free version of FGENESH+ [32] with the protein sequence of toxin matched in that region, as stated in the output “matched_regions.gtf,” and the gene model designed for *Anolis carolinensis*, which is the closely related species with a trained model in the FGENESH+ server; and (iii) checked the regions matching to full-length toxin CDSs (i.e., available in the output “matched_regions.gtf”) with no toxin annotated in the final annotation output (i.e., “toxin_annotation.gtf”) using FGENESH+ with the protein sequence of the matched toxin to ensure the region does not contain any toxin gene and may represent an intergenic region.

To annotate nontoxin genes, we used the funannotate pipeline [85]. This annotation pipeline consists of the integration of several *ab initio* gene predictors to build gene models (i.e., AUGUSTUS, SNAP, GeneMark−ES, and GlimmerHMM) and uses transcript and protein evidence to generate the final annotation set. Then, we set to use the venom gland transcriptomic data of the species as transcript evidence and the protein sequences available for the Tetrapoda clade in Uniprot and NCBI databases as protein evidence. We also performed the functional annotation step using InterProScan5 (v5.54; [86]).

We also performed a phylogenetic inference for the CTL genes to better characterize them as alpha and beta chains. We retrieved available venom CTL sequences from other snakes, aligned their peptide sequences using MAFFT (v7.450; [87]), and used IQTree (v1.6.12; [88]) to search for the maximum likelihood tree. The final tree was adjusted using FigTree (v1.4.4; [89]).

## Results

### Toxin annotation performance

**Figure 2:**
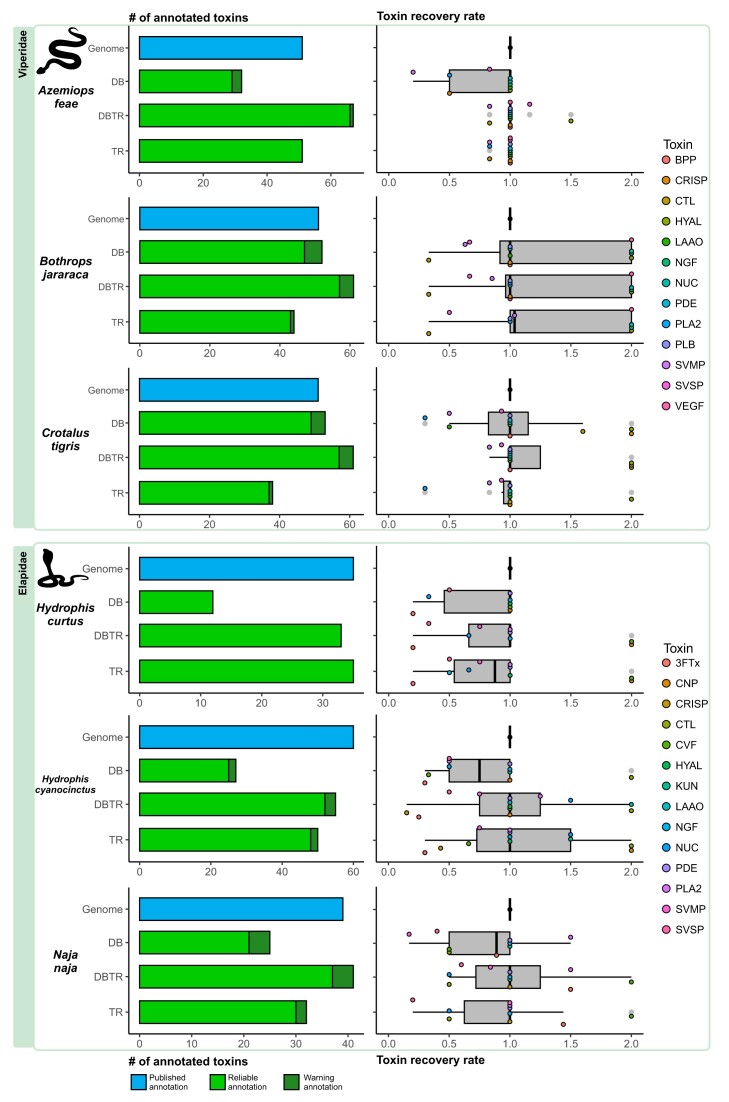
ToxCodAn-Genome performance in Viperidae and Elapidae testing sets. The barplots at the left indicate the number of toxins annotated in the published genome and in the tested scenarios. The genome represents the number of annotations as stated in the published manuscript (represented in blue), whereas the ToxCodAn-Genome outputs are classified as “reliable” (i.e., confident toxin annotations; represented in green) and “warning” (i.e., annotations that need further inspections; represented in dark green). The boxplots at right represent the toxin recovery rate (TRR) for major components of venom within each clade. The TRR is calculated as described in the Methods section. DB, ToxCodAn-Genome annotation using the toxin database only; DBTR, ToxCodAn-Genome annotation using the toxin database and the species-specific toxin-annotated transcriptome; TR, ToxCodAn-Genome annotations using the species-specific toxin-annotated transcriptome only.

**Figure 3:**
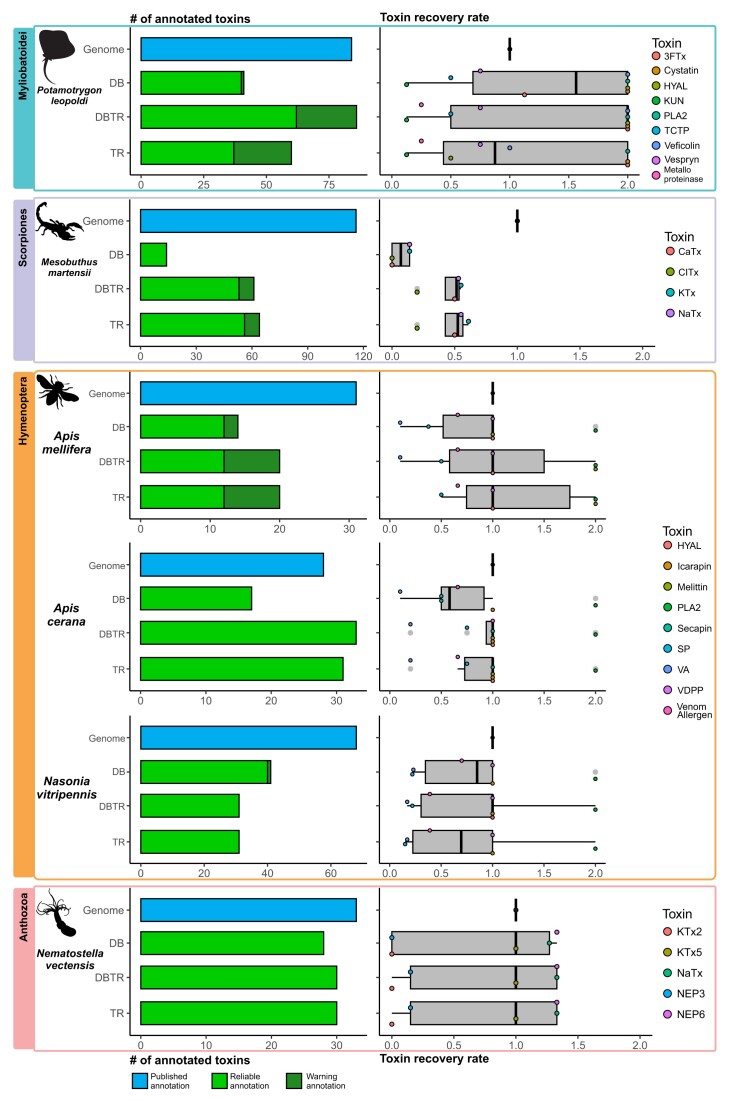
ToxCodAn-Genome performance in Myliobatoidea, Scorpiones, Hymenoptera, and Anthozoa testing sets. The barplots at the left indicate the number of toxins annotated in the published genome and in the tested scenarios. The genome represents the number of annotations as stated in the published manuscript (represented in blue), whereas the ToxCodAn-Genome outputs are classified as “reliable” (i.e., confident toxin annotations; represented in green) and “warning” (i.e., annotations that need further inspections; represented in dark green). The boxplots at right represent the toxin recovery rate (TRR) for major components of venom within each clade. The TRR is calculated as described in the Methods section. DB, ToxCodAn-Genome annotation using the toxin database only; DBTR, ToxCodAn-Genome annotation using the toxin database and the species-specific toxin-annotated transcriptome; TR, ToxCodAn-Genome annotations using the species-specific toxin-annotated transcriptome only.

Overall, the analysis using the genomes of 12 venomous species, including snakes (Fig. 2), stingrays, scorpions, hymenopterans, and anthozoans (Fig. 3), revealed that ToxCodAn-Genome can annotate most of their toxin gene repertoires in all tested scenarios (i.e., using the toxin database only and/or integrating venom–tissue transcriptomic data). In fact, ToxCodAn-Genome was able to match or surpass the number of toxin annotations in 7 of the 12 testing sets. This is particularly relevant when we consider that ToxCodAn-Genome can be executed in a single step, while the original genome annotations applied distinct protocols not easily reproducible. When using only the database or only the transcriptome, we noticed a lower number of annotated toxins when compared to the expected annotations in all testing sets. The integration of a database and venom–tissue transcriptomic data obtained the best performance for recovering the toxin gene repertoire in all testing sets. These results indicate that RNA-seq experiments alone are not able to identify low-expression toxin loci and that the combined use of homologous and orthologous sequences of closely related species (i.e., a toxinDB) improves the final toxin annotations. Also, the use of a toxinDB containing homologous toxin sequences allows the detection of pseudogenes and truncated paralogs, which may represent novel toxin compounds.

In the snake testing sets (Fig. 2), ToxCodAn-Genome presented a high performance in all genomes analyzed. This result may derive from the fact that snakes represent the most studied venomous lineage [9], and the availability of diverse toxin CDSs from several species may improve the toxin annotation performance. A higher number of toxin CDSs in the database may increase the probability of identifying orthologous and homologous toxins for target species, which results in a better recovery of toxin loci when using only the database but also when integrating the transcriptome. Interestingly, ToxCodAn-Genome annotated extra loci for some toxins in the *B. jararaca* testing set (i.e., toxins with TRR values of 2). This may represent bona fide duplication events not previously detected or may be a result of duplicated genomic regions in the genome assembly as observed in the BUSCO score of this genome assembly (Supplementary Table S1 in Supplementary File 3).

In the stingrays testing set (Fig. 3), the results revealed that most toxin genes were recovered when using the integration of database and transcriptome; however, most toxins were identified with more copies than expected (i.e., toxins with TRR values of 2). It may be related to the fact that we considered the annotations obtained from a *de novo* transcriptome assembly report as the expected number of toxins [66], which does not account for lowly or not expressed toxins in the genome. Therefore, efforts to perform a deep characterization and confirm the toxin repertoire of the *Potamotrygon leopoldi* genome may reveal a better picture of ToxCodAn-Genome performance within this venomous clade. Nonetheless, the availability of a Myliobatoidea toxin database will certainly help further genomic studies to retrieve the complete toxin repertoire of freshwater and saltwater stingrays.

In the Scorpiones testing set (Fig. 3), ToxCodAn-Genome returned a lower number of annotations than expected. This result may be related to the *Mesobuthus martensii* genome assembly quality, which presents only 53% of the BUSCO score (Supplementary Table S1 in Supplementary File 3). Their genome publication did not describe if all annotated toxins were full-length CDSs or if they may also be represented by partial CDSs in fragmented genomic regions [43]. The assembly quality is a feature shown to affect the annotation of complex genes [38], which may affect the toxin annotation performance of ToxCodAn-Genome as well. However, the TRR was consistent with the number of toxin annotations, which indicates that ToxCodAn-Genome may be able to recover most toxin loci in a high-quality genome assembly of scorpion species using the complete Scorpiones toxin database integrated with a species-specific venom–tissue transcriptome.

In the Hymenoptera testing set (Fig. 3), the number of toxin loci annotated was below the expected annotations in 2 datasets (i.e., *Apis mellifera* and *Nasonia vitripennis*) and achieved a good match in *Apis cerana*. However, the TRR value was close to 1 for most toxins in all testing sets. Interestingly, the *A. cerana* testing set returned the best performance in toxin annotation within the Hymenoptera clade. This may be related to the fact that most toxins studied and available for this clade in the toxin database are from *A. mellifera* (95 from 397) and *N. vitripennis* (71 from 397). In this sense, the lower performance obtained in the *A. mellifera* and *N. vitripennis* testing sets when compared to *A. cerana* may be related to the lower diversity of toxin sequences in the testing database when removing these target species. It indicates that the diversity of sequences in the database being used (i.e., the abundance of homologous and orthologous sequences) may interfere in the final toxin annotation set.

In the Anthozoa testing set (Fig. 3), the number of annotated toxins presented a good match to the number of expected annotations for most toxins. The TRR of the main toxins composing the venom of *Nemastotella vectensis* was close to 1 in all scenarios tested. Two toxin families were underrepresented in the final toxin annotation set (i.e., the TRR is lower than 1 for toxin families NEP3 and KTx2), but they represent lowly expressed components in the species [45]. The low performance to retrieve these 2 lowly expressed venom components of *N. vectensis* may be related to the high divergence observed in toxins among Anthozoa species [45]. However, ToxCodAn-Genome was able to fully annotate the most abundant toxin gene of the venom–tissue of *N. vectensis* (i.e., the toxin NaTx), which also present the most number of copies in their genome (i.e., 18 genes of NaTx in a total of 33 toxin genes) and a higher diversity of paralogs among *N. vectensis* populations [45]. In this sense, our tests indicate that ToxCodAn-Genome has high performance to retrieve the most complex toxin families in the genomes of Anthozoa species.

In summary, our tests revealed that any strategy alone allows a confident toxin annotation. Nonetheless, the integration of a toxin database with species-specific venom–tissue transcriptomic data presented the best performance and allowed the recovery of most toxin loci. In this sense, ToxCodAn-Genome is suitable for the toxin gene annotation task and can be applied to virtually any venomous lineage with the availability of curated toxin sequences from closely related species and/or a species-specific venom–tissue transcriptomic data.

### Proof-of-concept test of ToxCodAn-Genome

**Figure 4:**
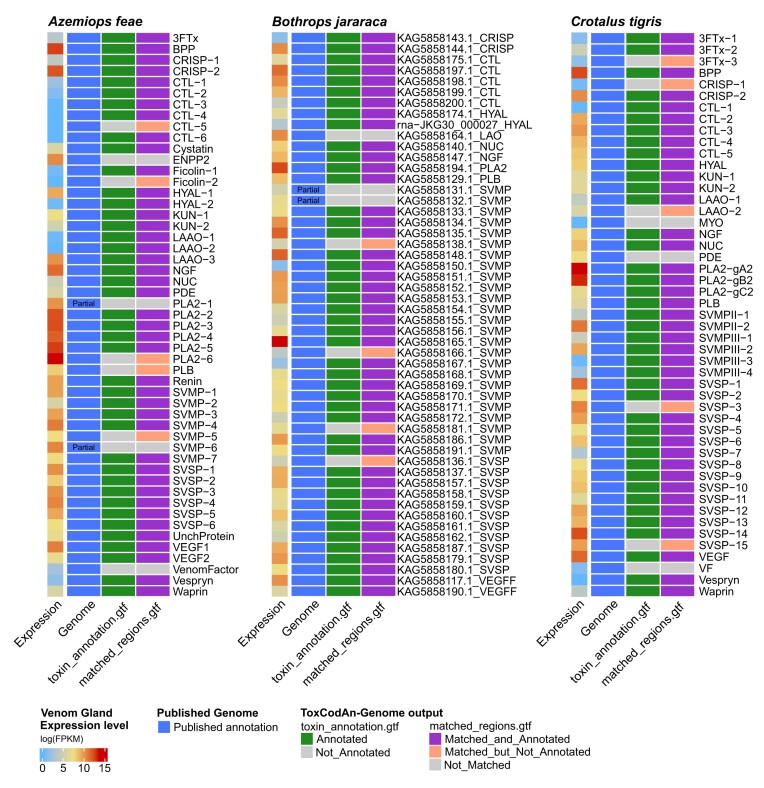
ToxCodAn-Genome performance to correctly annotate toxin genes and detect them as putative toxin loci using the Viperidae testing sets. The rows represent the toxin genes within each species’ genome, whereas the columns show the expression level, published genome annotation, and ToxCodAn-Genome output. The expression level was estimated using the venom gland transcriptome. The genome annotation shows mainly full-length toxins annotated in the genome but also shows when that specific toxin was partially annotated due to fragmented or incomplete genomic regions. The “toxin_annotation.gtf” shows when the toxin was annotated, in green, or not annotated, in gray. The “matched_regions.gtf” shows when the toxin was identified as a putative toxin loci and annotated, in purple, or not annotated, in salmon, or when it was not identified as a putative toxin loci, in gray.

The proof-of-concept test revealed that ToxCodAn-Genome is able to correctly annotate most of the toxins (Fig. 4). For the toxins not annotated by ToxCodAn-Genome, most of them were identified as matching into their specific genomic positions, which allows the user to easily retrieve their annotations by inspecting the “matched_regions.gtf” file when performing a manual curation. For the few toxins missing a confident match in the genome, we noticed that they were missing due to their partial annotation in the original publications or their gene sizes ranging out of the ToxCodAn-Genome default parameters (as discussed below in details for each testing set); however, it is a feature that can be modified to allow the annotation of such toxin genes with longer or shorter gene sizes. It is noticeable that most of the toxin genes annotated are among the highly expressed toxin genes, which shows the capability of ToxCodAn-Genome to correctly annotate major venom components in the genome.

In the *A. feae* testing set, ToxCodAn-Genome was able to confidently annotate 42 from a total of 51 toxin genes in the original publication (82.4% of precision in automatic annotation; Fig. 4). From the toxins not automatically annotated, 5 were identified in their correct positions in the “matched_regions.gtf” file, which indicate them as putative toxins (92.1% of precision in supervised annotation). Among the 4 missing toxins, 2 were marked as “partial” (PLA2-1 and SVMP-6; see Supplementary Information of [20]) and 2 presented a gene size longer than the threshold considered by ToxCodAn-Genome (i.e., ENPP2 has a gene size around 52 Kb and VenomFactor has a gene size around 380 Kb in the *A. feae* genome, whereas the threshold considers only genes with size shorter than 50 Kb). In this sense, modifying the gene size parameter when running ToxCodAn-Genome may allow to correctly identify both missing toxin genes.

The *B. jararaca* testing set revealed that 44 of the 51 toxin genes annotated in the publication were correctly annotated, which represents around 86.3% of precision in automatic annotation (Fig. 4). From the 7 toxins not properly annotated, 4 were correctly identified in the “matched_regions.gtf” file, which indicates putative toxins and they can be annotated with further inspections (94.1% of precision in supervised annotation). From the 3 toxins missing in the match file, we noticed that 2 were partial in the published annotation (i.e., KAG5858131.1_SVMP and KAG5858131.1_SVMP; [19]). The other missing toxin gene, KAG5858164.1_LAO, is marked as nonconclusive by the authors of their original publication (see Supplementary Information from [19]), which indicates that the gene may be split into 2 or more scaffolds. Although the authors were able to annotate parts of the gene and retrieve a full-length CDS for this toxin gene, their draft assembly and their approach to detect venom genes did not allow them to retrieve a complete annotation of this gene. In this sense, the missing toxin genes in the *B. jararaca* testing set can be related to incomplete genomic loci in the assembled genome.

Analyzing the *C. tigris* testing set showed that 42 toxin genes were correctly annotated from the total of 50 toxin genes, representing 84% of precision in automatic annotation (Fig. 4). From the 8 toxins not annotated, 5 had their genomic positions detected in the “matched_regions.gtf” file, whereas 3 were missed (94.0% of precision in supervised annotation). The 3 missing annotations all presented annotations outside the gene size and CDS size range. Specifically, PDE and VF have a gene size longer than the gene size threshold (i.e., greater than 50 Kb), whereas MYO has a CDS shorter than the CDS size threshold (i.e., MYO has a CDS size of 196 bp, whereas the minimum threshold is 200 bp). Adjusting such parameters may help to identify and annotate such toxin genes.

In summary, the proof-of-concept testing set allowed us to measure the capability of ToxCodAn-Genome to annotate most of the toxin genes, which included highly expressed toxin genes in the venom–tissue transcriptome. Among the missing genes, the user can deeply inspect the “matched_regions.gtf” file and modify some parameters to retrieve a complete set of annotated toxins. In this sense, ToxCodAn-Genome presents a high precision and also generated hints that allows the annotation of a complete set of toxins.

### Running time

We measured the processing times of ToxCodAn-Genome on annotating toxins in the *C. tigris* genome using a personal computer (Intel 6-Core i7 with 16 Gb memory). The test revealed that ToxCodAn-Genome can perform the toxin annotation task in 1 minute, 51 seconds when using only the database and 16 minutes, 23 seconds when also using the transcriptome assembly to complement the toxin survey, by using 6 threads (parameter “-c 6”). The running time of both strategies can be decreased by setting more CPUs to perform the annotation when available. It indicates that ToxCodAn-Genome is a fast tool that can be used on any personal computer with a UNIX operating system or can take advantage of supercomputers.

### *Bothrops alternatus* case study

The assembled genome of the urutu lancehead snake was of a higher quality than the available genome of the closely related species *B. jararaca* [19]. The assembled genome of *B. alternatus* had a total size of 1.7 Gb and is composed of 1,555 contigs with a N50 value of 13.9 Mb. BUSCO analysis revealed 95.8% of complete conserved tetrapoda orthologous genes, which indicates high contiguity and completeness (Supplementary Fig. S3 in Supplementary File 1 and Supplementary Table S2 in Supplementary File 3). We obtained a sequencing depth of 28.12 and a consensus quality score (QV) of 36.78, which indicates an accurate assembly with low error rate. The assembly revealed that 46.64% of the genome is composed of repetitive elements, which is in agreement with previous published genomes of vipers [17, 20, 48]. The funannotate pipeline allowed us to annotate 29,245 protein-coding genes, of which only 15 toxin genes were correctly annotated (Supplementary File 4). The toxin genes correctly annotated by funannotate were mainly composed of single-copy genes, which represent minor components of the venom.

**Figure 5:**
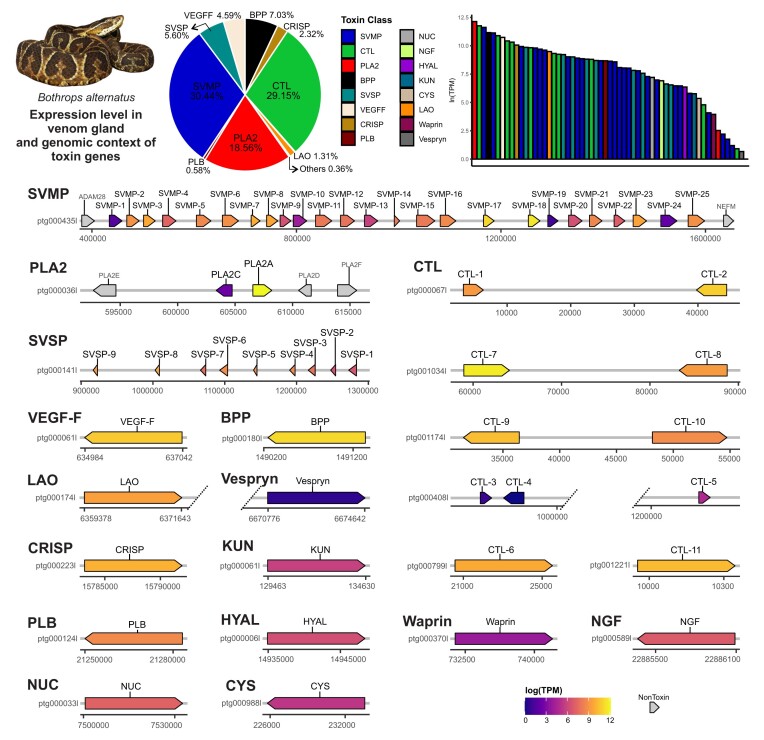
Expression level in venom gland and genomic context of toxin genes of *Bothrops alternatus*. The pie chart and barplot at the top refer to the expression level of toxin genes in the venom gland transcriptome, in which the toxins are color coded by their toxin class. The toxin loci plots at the bottom are color coded by expression level and the nontoxin genes flanking toxin loci are colored in light gray. BPP, bradykinin-potentiating peptides; CRISP, cysteine-rich secretory proteins; CTL, C-type lectins; CYS, cystatin; HYAL, hyaluronidase; KUN, Kunitz-type proteinase inhibitor; LAO, L-amino acid oxidase; NGF, nerve growth factor; NUC, Ecto 5′ nucleotidase; PDE, phosphodiesterase; PLA2, phospholipase A2; PLB, phospholipase B; SVMP, snake venom metalloproteinase; SVSP, snake venom serine protease; VEGF-F, vascular endothelial growth factor.

Using ToxCodAn-Genome, we annotated 59 toxin genes from 16 toxin families in the *B. alternatus* genome (Fig. 5; Supplementary Table S3 in Supplementary File 3). Similar to what was previously observed in *B. jararaca*, most toxin families were represented by a single locus (i.e., BPP, VEGF-F, LAO, PLB, HYAL, NGF, CRISP, KUN, NUC, CYS, and Waprin), whereas the other toxin families were organized as tandem arrays (i.e., SVMP, PLA2, and SVSP) and the CTLs were detected as pairs in several genomic regions. Among the expressed toxins, we noticed that PLA2s, SVMPs, SVSPs, and CTLs composed the major components of the venom gland transcriptome, which are also toxins with multiple copies in the genome (Fig. 5).

The PLA2s were arranged in tandem array between other nontoxin PLA2 genes (i.e., PLA2E, PLA2D, and PLA2F) and flanked by OTUD3 and MUL1 genes, which is a pattern broadly conserved across vipers and other nonvenomous tetrapods [19, 41, 48]. The PLA2A gene, which presents the highest expression level among all toxins, is an acidic D49 type and may be responsible for the phospholipase activity observed in the venom of *B. alternatus* [90]. Although we highlighted the PLA2C in the toxin set, it is lowly expressed and may have minor roles in the venom toxicity of the species, and it is hypothesized to be the precursor of PLA2s with high toxic functions in vipers [41, 46].

We were able to retrieve the complete snake venom metalloproteinase (SVMP) array in *B. alternatus* genome, which is composed of a total of 25 SVMP genes and flanked by ADAM28 and NEFM genes. This genomic context observed in SVMPs is broadly conserved among other vipers [17, 19, 42, 48], but it represents the first report of this complete locus in a *Bothrops* species. Of the total 25 SVMP genes, 20 represented a PIII class and 5 represented a PII class, corroborating a previous venom gland transcriptomics report [51]. Moreover, we did not identify any PI class, which is consistent with the *B. jararaca* genome [19]. Interestingly, the SVMP gene with the lowest expression level is a PIII neighboring the ADAM28 gene (SVMP-1), similar to that observed in other vipers [42]. On the other hand, the SVMPs with the highest expression level were detected in the central region of the SVMP loci. These SVMPs comprise one PIII class (SVMP-17) and one PII class (SVMP-18), and they are close to each other, which may indicate a similar evolutionary pressure shaping their expression levels. However, further epigenomics studies must be performed in *B. alternatus* to better understand the genetic regulatory network governing the expression level observed of SVMP genes.

We retrieved the complete snake venom serine protease (SVSP) array in *B. alternatus*, which is composed of 9 SVSP genes arranged in tandem arrays. This pattern was also reported in *B. jararaca* and other vipers [19]. Moreover, the number of SVSP genes detected is similar to that observed in the venom gland transcriptomic data of *B. alternatus* [51], but this number is lower than that identified in the *B. jararaca* genome [19]. This may be a result of lineage-specific duplications in *B. jararaca* or deletions in *B. alternatus*; however, further genomic studies including other *Bothrops* species must be performed to better understand the evolutionary history of SVSP genes in lancehead snakes.

We identified 11 CTL genes, from which 8 were highly expressed and 3 were lowly expressed. Differently from the other multicopy toxin families (PLA2, SVMP, and SVSP) that were clustered in a single contig, CTLs were spread in different genomic contigs and arranged in pairs with an inverted pattern. Each pair has an average distance of  30 Kb between them. Moreover, we also detected that 2 pairs of CTLs (i.e., CTL-1 and CTL-2 pair and CTL-9 and CTL-10 pair) were composed of alpha and beta chain representatives (Supplementary Fig. S4 in Supplementary File 1; see Supplementary File 5 for alignment and tree). Interestingly, the genomic context observed in CTLs has a similar pattern to that observed in the crotamine toxin genes in *C. viridis* [91], which is a toxin uniquely identified in some *Crotalus* species. It indicates that such arrangements may be also present in other toxins not deeply analyzed in genomics studies. The genomic context and arrangement of CTLs have not been previously described, but the draft assembly still leaves an open question of whether the CTL loci are located in the same chromosome region and if they are organized in tandem arrays. In this sense, assembling a chromosome-level genome of *B. alternatus* and other *Bothrops* species may help to elucidate whether this arrangement is broadly conserved and decipher the evolutionary history of CTL genes. This will bring fruitful insights about their biological roles and the regulatory mechanisms shaping the expression levels of CTLs in the venom gland of the *Bothrops* genus and other viperids as well.

All other toxin genes were identified as single-copy genes with a similar genomic context to that previously observed in *B. jararaca* [19] and other *Crotalus* species [17, 48]. In summary, we were able to characterize the toxin repertoire of the species, which may help in future research focusing on the evolution of toxins and solving the common ancestor toxin repertoire of *Bothrops* as well as in viper species.

## Discussion

The revolution in genome sequencing technologies has broadly transformed biological studies across research fields, including venomics, which mainly focuses on nonmodel organisms. However, the genome annotation tools currently available do not handle the issues accompanying the annotation of toxins, which can be extremely laborious and challenging. In fact, none of the genomes of venomous lineages published provide an automated script or a detailed description and documentation of the toxin annotation strategies applied, which hinders the reproducibility of results. Here, we provide a convenient computational tool, ToxCodAn-Genome, that can quickly identify most toxins in the genome, thereby minimizing the workload of checking toxin annotations and allowing improved reproducibility in further studies.

Our tests revealed that ToxCodAn-Genome can retrieve toxin annotations on virtually any venomous lineage by using a custom toxin database and/or species-specific venom–tissue transcriptomic data. We noticed that the integration of both datasets (i.e., a toxin database and a venom–tissue transcriptome) improves the final toxin annotation. Despite the high performance of ToxCodAn-Genome across all venomous clades tested, we noticed that a few limitations emerged, which can be related to (i) the diversity of toxin sequence availability in the toxin database, which can be improved by surveying more toxin sequences in literature and by the use a well-curated venom–tissue transcriptome of the target species, and (ii) the quality of the genome assembly being analyzed, which may disrupt the final toxin annotation; however, this is an extrinsic issue not related to the capabilities of ToxCodAn-Genome. To bypass such limitations, we designed a guide to help the users to improve the final toxin set. This guide was designed to direct the user to take advantage of all outputs generated by ToxCodAn-Genome, to learn how to improve the final toxin annotation by checking specific genomic regions (i.e., toxin-matched regions with no annotations as identified by ToxCodAn-Genome), and to ensure a well-annotated genome. The guide also contains detailed descriptions of the processes to build a custom toxin database when venom–tissue transcriptomic data are available or not, to perform nontoxin annotation, to quantify gene expression, and to plot the toxin loci for reports and publications.

The proof-of-concept test showed that ToxCodAn-Genome can annotate most of the toxins in the genome, which integrates the set of highly expressed toxins in the venom–tissue transcriptome. The few missing toxin genes were not annotated due to fragmented or unresolved genome assembly in the toxin regions, which may generate partial toxin annotations, or due to their genomic architecture that were not in the default range considered by ToxCodan-Genome. The genome assembly quality is a feature extrinsic to ToxCodAn-Genome, whereas the genomic architecture parameters can be modified to allow the user to retrieve a complete set of annotated toxins.

It is important to note that ToxCodAn-Genome was designed to be customizable and the user can test distinct parameters to improve the final toxin annotation set for the studied lineage. For instance, the user can set different percent identity thresholds, gene sizes, and CDS lengths, as well as include or not a custom toxin database generated with published and/or unpublished data. Additionally, the user can include the UniProt or ToxProt databases to generate a report containing the best match between the annotated toxins and the database entries. Finally, the user can follow the guide to better interpret the outputs and fill the gaps of the limitations observed in the current tests.

Despite the availability of toxin databases for only a few venomous lineages to date, ToxCodAn-Genome can be expanded to annotate any venomous clade and species by using a specific set of full-length toxin CDSs. The user can follow our guide to design specific toxin databases by surveying sequences and/or analyzing venom–tissue transcriptomic data available in several databases, such as GenBank and TSA from NCBI, ENA from EMBL, and China National GeneBank DataBase (CNGBdb). Moreover, the constant expansion of genomic and transcriptomic data deposited and available for venomous lineages in these databases will allow us to keep these predesigned toxin databases up to date and also expand the set of toxin databases to encompass other venomous clades in the near future [40].

ToxCodAn-Genome can be easily installed on any UNIX-like operating system and is fast, taking only a few minutes to analyze a genome in a personal computer (Intel 6-Core i7 with 16 Gb memory). These resources are available on most modern desktop and laptop computers, demonstrating the applicability of ToxCodAn-Genome for projects of any size, regardless of available computational resources. Moreover, the fast running time allows the user to perform several tests with distinct parameters to reach a high-quality final toxin annotation set.

ToxCodAn-Genome allowed us to easily characterize the toxin gene repertoire of *B. alternatus*. It revealed that the most abundant toxin families comprising the venom of *B. alternatus* and also in other Viperidae species are those that underwent more expansion (i.e., SVMP, SVSP, PLA2, and CTL). The first complete SVMP locus obtained for a lancehead revealed a similar genomic context to that observed in other viperids [17, 19, 42, 48], with the SVMP gene located closer to the ADAM28 gene being the lowest expressed among all SVMP genes. The other loci presented a similar genomic context as previously described, but we also identified an interesting genomic arrangement of CTL genes, which may be further analyzed using chromosome-level genomes to confirm if this pattern is widely conserved among vipers. Moreover, the draft genome assembly and the complete toxin repertoire obtained for *B. alternatus* in the present study can be a useful resource for further experiments focusing on better understanding the intraspecific variation of venom composition observed in *B. alternatus* [51, 77, 78]. Such experiments can increase the sampling size and apply genomic and epigenomic approaches to reveal if it may be related to deletion and/or duplication events within toxin genes [42] or if it may be related to nucleotide changes in promoter and enhancer regions of these toxin genes [6]. Furthermore, the current assembly and toxin annotation can be integrated into comparative analysis with other *Bothrops* and viper species to reconstruct the toxin genomic repertoire of their common ancestor and improve the evolutionary history of venom components within the genus and also within viperids [42].

The genome annotation step of *B. alternatus* revealed that even sophisticated approaches, like funannotate, which integrates several tools and strategies in their pipeline to perform an automated genome annotation, fails to correctly annotate the entire set of toxin genes (i.e., only 14 from a total of 59 toxin genes; Supplementary File 4). It reveals that common features considered when annotating most genes do not fit well when annotating toxin genes, which are genomic regions commonly accompanied by high mutation rates, recent duplication and loss events, and the presence of orphan exons. Our case study and previous reports show that extra features are needed to be considered when annotating and studying toxin genes [15–17, 19, 21, 43, 44, 48, 49]. In this sense, ToxCodAn-Genome takes into consideration key features to correctly annotate toxins (i.e., 49 from 59 toxin genes in *B. alternatus*; Supplementary File 4), but it still needs improvements to solve some pitfalls related to automatically confirm the status of “warning” annotations as truncated paralogs or pseudogenes (i.e., 1 from 59 toxin genes in *B. alternatus*) and to better interpret matched regions with no annotations (i.e., 9 from 59 toxin genes in *B. alternatus*). Of note, the constant expansion of available high-quality genomes and well-annotated toxin annotations of venomous lineages may represent an outstanding opportunity to apply machine learning algorithms to help on the toxin annotation task in the near future [92].

Although ToxCodAn-Genome performed very satisfactory in the tests performed here, users should be aware of some limitations: (i) Our tool does not perform annotation of partial genes located in fragmented or incomplete genomic contigs; (2) ToxCodAn-Genome only considers canonical start and stop codons and splicing sites, which may inhibit the annotation of toxin genes with noncanonical signals; and (3) ToxCodAn-Genome is dependent on the user knowledge about the toxin gene repertoire of the species being studied to set and test the best parameters for the species being studied. Such limitations may be surpassed in further updates by integrating prebuilt and self-training gene models to predict the toxin gene structures and also integrating the possibility to consider noncanonical start and stop codons and splicing sites, which can be set by the user. Also, the user’s knowledge about the toxins and putative toxins may help to better characterize the complete set of toxins in the analyzed genome and can be acquired in scientific resources, like VenomZone [62], ToxProt [63], ConoServer [64], ArachnoServer [65], and scientific literature. Of note, we intend to keep ToxCodAn-Genome up to date by releasing a major update every year; such updates will include improvements in the code to retrieve better performance in toxin annotations and integration of novel high-performance tools, as well as increasing the toxin database entries, as soon as more genomes and transcriptomes of venomous lineages are available in the years to come. Also, we are open to receive feedback to improve the tool and add the toxin sequences annotated and/or entire custom toxin databases designed by users who want to assist ToxCodAn-Genome and the scientific community working with venomous lineages.

ToxCodAn-Genome was designed to annotate toxin genes, but we believe that it may also be applied to annotate analogous cases of functional gene categories presenting similar genomic features to those observed in toxin families. For example, chemosensory genes [67, 93], opsin genes [94], olfactory receptor genes [95], major histocompatibility complex genes [96], fetuin metalloproteinase inhibitor genes [97], hox genes [98], and other gene families expanded during evolution and adaptation of specific lineages can, in theory, be annotated by this tool. In fact, these genomic regions are poorly characterized by automated genome annotation tools and require laborious manual inspection to accurately annotate and identify the complete set of genes [38, 39]. In this sense, ToxCodAn-Genome may represent a suitable tool to help with specific gene-type annotation tasks and improve research on any genomic study.

## Conclusion

ToxCodAn-Genome is the first tool that can be easily applied to annotate toxin genes in genome assemblies of any venomous species. It is fast and suitable for use on projects of any size. We also provide a guide to help researchers perform such toxin gene annotations and also check for truncated paralogs and pseudogenes. We provide prebuilt toxin databases for snakes (Viperidae and Elapidae clades), Myliobatoidei, Scorpiones, Hymenoptera, and Anthozoa, which can be integrated to the use of venom–tissue transcriptomic data. Moreover, ToxCodAn-Genome can be expanded to use in any venomous lineages by designing novel and custom toxin databases and also using venom transcriptomic data specific to the studied lineage. In addition, through our study case, we revealed the toxin genomic repertoire of the urutu lancehead, a widely distributed pit viper in South America.

## Availability of Source Code and Requirements

- Project name: ToxCodAn-Genome
- Project homepage: https://github.com/pedronachtigall/ToxCodAn-Genome
- Operating system: UNIX
- Programming language: Python
- Other requirements: Biopython, Pandas, BLAST, Exonerate, and GffRead
- License: GNU GPLv3
- Biotools ID: toxcodan-genome
- RRID: SCR_024718

## Supplementary Material

giad116_GIGA-D-23-00178_Original_Submission

giad116_GIGA-D-23-00178_Revision_1

giad116_GIGA-D-23-00178_Revision_2

giad116_Response_to_Reviewer_Comments_Original_Submission

giad116_Response_to_Reviewer_Comments_Revision_1

giad116_Reviewer_1_Report_Original_Submission Choo Hock Tan -- 8/12/2023 Reviewed

giad116_Reviewer_2_Report_Original_Submission Zachary Kenneth Stewart -- 8/30/2023 Reviewed

giad116_Reviewer_2_Report_Revision_1 Zachary Kenneth Stewart -- 11/2/2023 Reviewed

giad116_Reviewer_3_Report_Original_Submission Jason Macrander, Ph. D. -- 9/18/2023 Reviewed

giad116_Supplemental_Files

## Data Availability

The genome assembly and the PacBio HiFi reads of *B. alternatus* are available under the accession numbers JARGCV000000000 and SRR23725375 in the NCBI [59]. In addition, the assembled genome [99], annotations [100, 101], and BUSCO analysis [102] are available in the figshare database. ToxCodAn-Genome and the guide are freely available via the GitHub repository [58, 103]. An archival copy of the code and supporting data is available via the *GigaScience* repository, GigaDB 102487 [104].

## Additional Files

**Supplementary Fig. S1**. The “Transcriptome assembly” and “Toxin CDS annotation” workflows that can be used to generate the custom toxin database through the use of a venom–tissue transcriptomic data. The transcriptome assembly performs 2 strategies to recover transcripts: (i) a genome-guided transcriptome assembly, which maps reads using Hisat2 and retrieves transcripts using StringTie and Trinity, and (ii) a *de novo* transcriptome assembly, which uses Trinity and rnaSPAdes to assemble transcripts. The “Toxin CDS annotation” step identifies full-length toxin CDSs in the assembled transcripts by performing BLAST search against a toxin database.

**Supplementary Fig. S2**. The annotations performed by ToxCodAn-Genome can be characterized into “reliable toxin annotation,” “warning annotation,” and “matched region with no annotation.” The “reliable toxin annotation” represents an annotation identified in a genomic region containing a full-length toxin CDS and a confident gene structure. The “warning annotation” indicates an annotation in a genomic region containing a full-length toxin CDS with a confident gene structure but containing a premature stop codon, which may reveal a putative novelty, a truncated paralog toxin gene, a pseudogene, or an erroneous annotation. The annotations characterized as “warning annotation” must be manually inspected to confirm its status. The “matched region with no annotation” represents a genomic region matching a full-length toxin CDS, but the refinement of the exon/intron boundaries step does not return a toxin annotation. The region marked as “matched region with no annotation” can be manually inspected to confirm if it has or not a toxin gene.

**Supplementary Fig. S3**. Basic assembly statistics of the *Bothrops alternatus* genome and BUSCO completeness using the tetrapoda gene set (odb10; total of 5,310 genes).

**Supplementary Fig. S4**. CTL phylogeny with known alpha and beta chain CTL homologs of several snake species. The support values of bootstrap are given in tree branches.

**Supplementary Table S1**. Basic genomic statistics, toxins annotations, and results of ToxCodAn-Genome of each testing set.

**Supplementary Table S2**. Genomics statistics of *Bothrops alternatus* obtained in the present study.

**Supplementary Table S3**. Toxin annotation of *Bothrops alternatus* genome.

## Abbreviations

BPP: bradykinin potentiating peptide; CDS: coding sequence; CRISP: cysteine-rich secretory protein; CTL: C-type lectin; HYAL: hyaluronidase; LAO/LAAO: L-amino acid oxidase; NGF: nerve growth factor; NUC: snake venom 5′ nucleotidase; PDE: phosphodiesterase; PLA2: phospholipase A_2_; PLB: phospholipase B; SVMP: snake venom metalloproteinase; SVSP - Snake venom serine protease; TPM: transcripts per million; VEGF-F: snake venom vascular endothelial growth factor.
