# Supplementary material for: ToxCodAn-Genome: an automated pipeline for toxin-gene annotation in genome assembly of venomous lineages
Source: Gigascience. 2024 Jan 18;13:giad116. doi: 10.1093/gigascience/giad116 (PMC10797961; doi:10.1093/gigascience/giad116)
Supplement: giad116_GIGA-D-23-00178_Revision_2 [file giad116_giga-d-23-00178_revision_2.pdf]

GigaScience

ToxCodAn-Genome: an automated pipeline for toxin-gene annotation in genome assembly of venomous lineages

--Manuscript Draft--

**Manuscript Number:**	GIGA-D-23-00178R2
**Full Title:**	ToxCodAn-Genome: an automated pipeline for toxin-gene annotation in genome assembly of venomous lineages
**Article Type:**	Technical Note
**Funding Information:**	Fundação de Amparo à Pesquisa do Estado de São Paulo (2018/26520-4) — Dr Pedro Gabriel Nachtigall<br>Fundação de Amparo à Pesquisa do Estado de São Paulo (2016/50127-5) — Dr Inácio L.M. Junqueira-de-Azevedo<br>Fundação de Amparo à Pesquisa do Estado de São Paulo (2022/04988-0) — Dr Pedro Gabriel Nachtigall<br>National Science Foundation (NSF DEB 1638902) — Dr Darin R. Rokyta
**Abstract:**	The rapid development of sequencing technologies resulted in a wide expansion of genomics studies using venomous lineages. This facilitated research focusing on understanding the evolution of adaptive traits and the search for novel compounds that can be applied in agriculture and medicine. However, the toxin annotation of genomes is a laborious and time-consuming task, and no consensus pipeline is currently available. No computational tool currently exists to address the challenges specific to toxin annotation and to ensure the reproducibility of the process. Here, we present ToxCodAn-Genome, the first software designed to perform automated toxin annotation in genomes of venomous lineages. This pipeline was designed to retrieve the full-length coding sequences of toxins and to allow the detection of novel truncated paralogs and pseudogenes. We tested ToxCodAn-Genome using 12 genomes of venomous lineages and achieved high performance on recovering their current toxin annotations. This tool can be easily customized to allow improvements in the final toxin annotation set and can be expanded to virtually any venomous lineage. ToxCodAn-Genome is fast, allowing it to run on any personal computer, but it can also be executed in multi-core mode, taking advantage of large high-performance servers. In addition, we provide a guide to direct future research in the venomics field to ensure a confident toxin annotation in the genome being studied. As a case study, we sequenced and annotated the toxin repertoire of Bothrops alternatus, which may facilitate future evolutionary and biomedical studies using vipers as models. ToxCodAn-Genome is suitable to perform toxin annotation in the genome of venomous species and may help to improve the reproducibility of further studies. ToxCodAn-Genome and the guide are freely available at https://github.com/pedronachtigall/ToxCodAn-Genome.
**Corresponding Author:**	Pedro Gabriel Nachtigall<br>Instituto Butantan<br>Sao Paulo, São Paulo BRAZIL
**Corresponding Author Secondary Information:**	
**Corresponding Author's Institution:**	Instituto Butantan
**Corresponding Author's Secondary Institution:**	
**First Author:**	Pedro Gabriel Nachtigall
**First Author Secondary Information:**	
**Order of Authors:**	Pedro Gabriel Nachtigall<br>Alan M Durham

Powered by Editorial Manager® and ProduXion Manager® from Aries Systems Corporation

	Darin R. Rokyta
	Inácio L.M. Junqueira-de-Azevedo
**Order of Authors Secondary Information:**	
**Response to Reviewers:**	We thank the editor and reviewers for bringing excellent points to improve the final code and manuscript and for accepting our manuscript for publication.
**Additional Information:**	
**Question**	**Response**
Are you submitting this manuscript to a special series or article collection?	No
**Experimental design and statistics**<br><br>Full details of the experimental design and statistical methods used should be given in the Methods section, as detailed in our Minimum Standards Reporting Checklist. Information essential to interpreting the data presented should be made available in the figure legends.<br><br>Have you included all the information requested in your manuscript?	Yes
**Resources**<br><br>A description of all resources used, including antibodies, cell lines, animals and software tools, with enough information to allow them to be uniquely identified, should be included in the Methods section. Authors are strongly encouraged to cite Research Resource Identifiers (RRIDs) for antibodies, model organisms and tools, where possible.<br><br>Have you included the information requested as detailed in our Minimum Standards Reporting Checklist?	Yes
**Availability of data and materials**<br><br>All datasets and code on which the conclusions of the paper rely must be either included in your submission or deposited in publicly available repositories	Yes

Powered by Editorial Manager® and ProduXion Manager® from Aries Systems Corporation

(where available and ethically appropriate), referencing such data using a unique identifier in the references and in the “Availability of Data and Materials” section of your manuscript.<br><br>Have you have met the above requirement as detailed in our Minimum Standards Reporting Checklist?	

Click here to access/download;LaTeX - Main Document (TeX file);main.tex

```
This is pdfTeX, Version 3.141592653-2.6-1.40.24 (TeX Live 2022)
(preloaded format=pdflatex 2023.3.8)  11 DEC 2023 13:22
entering extended mode
 restricted \write18 enabled.
 %&-line parsing enabled.
**main.tex
(./main.tex
LaTeX2e <2022-11-01> patch level 1
L3 programming layer <2023-02-22> (./oup-contemporary.cls
Document Class: oup-contemporary 2017/06/28, v1.1
(c:/TeXLive/2022/texmf-dist/tex/latex/base/article.cls
Document Class: article 2022/07/02 v1.4n Standard LaTeX document class
(c:/TeXLive/2022/texmf-dist/tex/latex/base/size10.clo
File: size10.clo 2022/07/02 v1.4n Standard LaTeX file (size option)
)
\c@part=\count185
\c@section=\count186
\c@subsection=\count187
\c@subsubsection=\count188
\c@paragraph=\count189
\c@subparagraph=\count190
\c@figure=\count191
\c@table=\count192
\abovecaptionskip=\skip48
\belowcaptionskip=\skip49
\bibindent=\dimen140
) (c:/TeXLive/2022/texmf-dist/tex/latex/base/inputenc.sty
Package: inputenc 2021/02/14 v1.3d Input encoding file
\inpenc@prehook=\toks16
\inpenc@posthook=\toks17
) (c:/TeXLive/2022/texmf-dist/tex/latex/base/fontenc.sty
Package: fontenc 2021/04/29 v2.0v Standard LaTeX package
) (c:/TeXLive/2022/texmf-dist/tex/generic/iftex/ifpdf.sty
Package: ifpdf 2019/10/25 v3.4 ifpdf legacy package. Use iftex instead.
(c:/TeXLive/2022/texmf-dist/tex/generic/iftex/iftex.sty
Package: iftex 2022/02/03 v1.0f TeX engine tests
)) (c:/TeXLive/2022/texmf-dist/tex/latex/microtype/microtype.sty
Package: microtype 2023/03/13 v3.1a Micro-typographical refinements (RS)
(c:/TeXLive/2022/texmf-dist/tex/latex/graphics/keyval.sty
Package: keyval 2022/05/29 v1.15 key=value parser (DPC)
\KV@toks@=\toks18
) (c:/TeXLive/2022/texmf-dist/tex/latex/etoolbox/etoolbox.sty
Package: etoolbox 2020/10/05 v2.5k e-TeX tools for LaTeX (JAW)
\etb@tempcnta=\count193
)
\MT@toks=\toks19
\MT@tempbox=\box51
\MT@count=\count194
LaTeX Info: Redefining \noprotrusionifhmode on input line 1059.
LaTeX Info: Redefining \leftprotrusion on input line 1060.
\MT@prot@toks=\toks20
LaTeX Info: Redefining \rightprotrusion on input line 1078.
LaTeX Info: Redefining \textls on input line 1368.
\MT@outer@kern=\dimen141
```

```
LaTeX Info: Redefining \textmicrotypecontext on input line 1988.
\MT@listname@count=\count195
(c:/TeXLive/2022/texmf-dist/tex/latex/microtype/microtype-pdftex.def
File: microtype-pdftex.def 2023/03/13 v3.1a Definitions specific to
pdftex (RS)

LaTeX Info: Redefining \lsstyle on input line 902.
LaTeX Info: Redefining \lslig on input line 902.
\MT@outer@space=\skip50
)
Package microtype Info: Loading configuration file microtype.cfg.
(c:/TeXLive/2022/texmf-dist/tex/latex/microtype/microtype.cfg
File: microtype.cfg 2023/03/13 v3.1a microtype main configuration file
(RS)
)) (c:/TeXLive/2022/texmf-dist/tex/latex/euler/euler.sty
Package: euler 1995/03/05 v2.5
Package: `euler' v2.5 <1995/03/05> (FJ and FMi)
LaTeX Font Info:    Redeclaring symbol font `letters' on input line 35.
LaTeX Font Info:    Encoding `OML' has changed to `U' for symbol font
(Font)              `letters' in the math version `normal' on input line
35.
LaTeX Font Info:    Overwriting symbol font `letters' in version `normal'
(Font)                  OML/cmm/m/it --> U/eur/m/n on input line 35.
LaTeX Font Info:    Encoding `OML' has changed to `U' for symbol font
(Font)              `letters' in the math version `bold' on input line
35.
LaTeX Font Info:    Overwriting symbol font `letters' in version `bold'
(Font)                  OML/cmm/b/it --> U/eur/m/n on input line 35.
LaTeX Font Info:    Overwriting symbol font `letters' in version `bold'
(Font)                  U/eur/m/n --> U/eur/b/n on input line 36.
LaTeX Font Info:    Redeclaring math symbol \Gamma on input line 47.
LaTeX Font Info:    Redeclaring math symbol \Delta on input line 48.
LaTeX Font Info:    Redeclaring math symbol \Theta on input line 49.
LaTeX Font Info:    Redeclaring math symbol \Lambda on input line 50.
LaTeX Font Info:    Redeclaring math symbol \Xi on input line 51.
LaTeX Font Info:    Redeclaring math symbol \Pi on input line 52.
LaTeX Font Info:    Redeclaring math symbol \Sigma on input line 53.
LaTeX Font Info:    Redeclaring math symbol \Upsilon on input line 54.
LaTeX Font Info:    Redeclaring math symbol \Phi on input line 55.
LaTeX Font Info:    Redeclaring math symbol \Psi on input line 56.
LaTeX Font Info:    Redeclaring math symbol \Omega on input line 57.
\symEulerFraktur=\mathgroup4
LaTeX Font Info:    Overwriting symbol font `EulerFraktur' in version
`bold'
(Font)                  U/euf/m/n --> U/euf/b/n on input line 63.
LaTeX Info: Redefining \oldstylenums on input line 85.
\symEulerScript=\mathgroup5
LaTeX Font Info:    Overwriting symbol font `EulerScript' in version
`bold'
(Font)                  U/eus/m/n --> U/eus/b/n on input line 93.
LaTeX Font Info:    Redeclaring math symbol \aleph on input line 97.
LaTeX Font Info:    Redeclaring math symbol \Re on input line 98.
LaTeX Font Info:    Redeclaring math symbol \Im on input line 99.
LaTeX Font Info:    Redeclaring math delimiter \vert on input line 101.
```

```
LaTeX Font Info:    Redeclaring math delimiter \backslash on input line
103.
LaTeX Font Info:    Redeclaring math symbol \neg on input line 106.
LaTeX Font Info:    Redeclaring math symbol \wedge on input line 108.
LaTeX Font Info:    Redeclaring math symbol \vee on input line 110.
LaTeX Font Info:    Redeclaring math symbol \setminus on input line 112.
LaTeX Font Info:    Redeclaring math symbol \sim on input line 113.
LaTeX Font Info:    Redeclaring math symbol \mid on input line 114.
LaTeX Font Info:    Redeclaring math delimiter \arrowvert on input line
116.
LaTeX Font Info:    Redeclaring math symbol \mathsection on input line
117.
\symEulerExtension=\mathgroup6
LaTeX Font Info:    Redeclaring math symbol \coprod on input line 125.
LaTeX Font Info:    Redeclaring math symbol \prod on input line 125.
LaTeX Font Info:    Redeclaring math symbol \sum on input line 125.
LaTeX Font Info:    Redeclaring math symbol \intop on input line 130.
LaTeX Font Info:    Redeclaring math symbol \ointop on input line 131.
LaTeX Font Info:    Redeclaring math symbol \braceld on input line 132.
LaTeX Font Info:    Redeclaring math symbol \bracerd on input line 133.
LaTeX Font Info:    Redeclaring math symbol \bracelu on input line 134.
LaTeX Font Info:    Redeclaring math symbol \braceru on input line 135.
LaTeX Font Info:    Redeclaring math symbol \infty on input line 136.
LaTeX Font Info:    Redeclaring math symbol \nearrow on input line 153.
LaTeX Font Info:    Redeclaring math symbol \searrow on input line 154.
LaTeX Font Info:    Redeclaring math symbol \nwarrow on input line 155.
LaTeX Font Info:    Redeclaring math symbol \swarrow on input line 156.
LaTeX Font Info:    Redeclaring math symbol \Leftrightarrow on input line
157.
LaTeX Font Info:    Redeclaring math symbol \Leftarrow on input line 158.
LaTeX Font Info:    Redeclaring math symbol \Rightarrow on input line
159.
LaTeX Font Info:    Redeclaring math symbol \leftrightarrow on input line
160.
LaTeX Font Info:    Redeclaring math symbol \leftarrow on input line 161.
LaTeX Font Info:    Redeclaring math symbol \rightarrow on input line
163.
LaTeX Font Info:    Redeclaring math delimiter \uparrow on input line
166.
LaTeX Font Info:    Redeclaring math delimiter \downarrow on input line
168.
LaTeX Font Info:    Redeclaring math delimiter \updownarrow on input line
170.
LaTeX Font Info:    Redeclaring math delimiter \Uparrow on input line
172.
LaTeX Font Info:    Redeclaring math delimiter \Downarrow on input line
174.
LaTeX Font Info:    Redeclaring math delimiter \Updownarrow on input line
176.
LaTeX Font Info:    Redeclaring math symbol \leftharpoonup on input line
177.
LaTeX Font Info:    Redeclaring math symbol \leftharpoondown on input
line 178.
```

```
LaTeX Font Info:    Redeclaring math symbol \rightharpoonup on input line
179.
LaTeX Font Info:    Redeclaring math symbol \rightharpoondown on input
line 180
.
LaTeX Font Info:    Redeclaring math delimiter \lbrace on input line 182.
LaTeX Font Info:    Redeclaring math delimiter \rbrace on input line 184.
\symcmmigroup=\mathgroup7
LaTeX Font Info:    Overwriting symbol font `cmmigroup' in version `bold'
(Font)                  OML/cmm/m/it --> OML/cmm/b/it on input line 200.
LaTeX Font Info:    Redeclaring math accent \vec on input line 201.
LaTeX Font Info:    Redeclaring math symbol \triangleleft on input line
202.
LaTeX Font Info:    Redeclaring math symbol \triangleright on input line
203.
LaTeX Font Info:    Redeclaring math symbol \star on input line 204.
LaTeX Font Info:    Redeclaring math symbol \lhook on input line 205.
LaTeX Font Info:    Redeclaring math symbol \rhook on input line 206.
LaTeX Font Info:    Redeclaring math symbol \flat on input line 207.
LaTeX Font Info:    Redeclaring math symbol \natural on input line 208.
LaTeX Font Info:    Redeclaring math symbol \sharp on input line 209.
LaTeX Font Info:    Redeclaring math symbol \smile on input line 210.
LaTeX Font Info:    Redeclaring math symbol \frown on input line 211.
LaTeX Font Info:    Redeclaring math accent \grave on input line 245.
LaTeX Font Info:    Redeclaring math accent \acute on input line 246.
LaTeX Font Info:    Redeclaring math accent \tilde on input line 247.
LaTeX Font Info:    Redeclaring math accent \ddot on input line 248.
LaTeX Font Info:    Redeclaring math accent \check on input line 249.
LaTeX Font Info:    Redeclaring math accent \breve on input line 250.
LaTeX Font Info:    Redeclaring math accent \bar on input line 251.
LaTeX Font Info:    Redeclaring math accent \dot on input line 252.
LaTeX Font Info:    Redeclaring math accent \hat on input line 254.
) (c:/TeXLive/2022/texmf-dist/tex/latex/merriweather/merriweather.sty
Package: merriweather 2022/09/20 (Bob Tennent) Supports
Merriweather(Sans) font
s for all LaTeX engines.
(c:/TeXLive/2022/texmf-dist/tex/generic/iftex/ifxetex.sty
Package: ifxetex 2019/10/25 v0.7 ifxetex legacy package. Use iftex
instead.
) (c:/TeXLive/2022/texmf-dist/tex/generic/iftex/ifluatex.sty
Package: ifluatex 2019/10/25 v1.5 ifluatex legacy package. Use iftex
instead.
) (c:/TeXLive/2022/texmf-dist/tex/latex/base/textcomp.sty
Package: textcomp 2020/02/02 v2.0n Standard LaTeX package
) (c:/TeXLive/2022/texmf-dist/tex/latex/xkeyval/xkeyval.sty
Package: xkeyval 2022/06/16 v2.9 package option processing (HA)
(c:/TeXLive/2022/texmf-dist/tex/generic/xkeyval/xkeyval.tex
(c:/TeXLive/2022/te
xmf-dist/tex/generic/xkeyval/xkvutils.tex
\XKV@toks=\toks21
\XKV@tempa@toks=\toks22
)
\XKV@depth=\count196
File: xkeyval.tex 2014/12/03 v2.7a key=value parser (HA)
```

```
)) (c:/TeXLive/2022/texmf-dist/tex/latex/base/fontenc.sty
Package: fontenc 2021/04/29 v2.0v Standard LaTeX package
) (c:/TeXLive/2022/texmf-dist/tex/latex/fontaxes/fontaxes.sty
Package: fontaxes 2020/07/21 v1.0e Font selection axes
LaTeX Info: Redefining \upshape on input line 29.
LaTeX Info: Redefining \itshape on input line 31.
LaTeX Info: Redefining \slshape on input line 33.
LaTeX Info: Redefining \swshape on input line 35.
LaTeX Info: Redefining \scshape on input line 37.
LaTeX Info: Redefining \sscshape on input line 39.
LaTeX Info: Redefining \ulcshape on input line 41.
LaTeX Info: Redefining \textsw on input line 47.
LaTeX Info: Redefining \textssc on input line 48.
LaTeX Info: Redefining \textulc on input line 49.
)) (c:/TeXLive/2022/texmf-dist/tex/latex/mathastext/mathastext.sty
Package: mathastext 2022/11/04 v1.3y Use the text font in math mode (JFB)
\mst@exists@muskip=\muskip16
\mst@forall@muskip=\muskip17
\mst@prime@muskip=\muskip18
\mst@do@nonletters=\toks23
\mst@do@easynonletters=\toks24
\mst@do@az=\toks25
\mst@do@AZ=\toks26
\symmtoperatorfont=\mathgroup8
\symmtletterfont=\mathgroup9
** ! and ?
** punctuation: , . : ; and \colon
LaTeX Info: Redefining \relbar on input line 844.
LaTeX Info: Redefining \rightarrowfill on input line 847.
LaTeX Info: Redefining \leftarrowfill on input line 852.
** + and =
LaTeX Info: Redefining \Relbar on input line 943.
** adding = ; and + to \nfss@catcodes
** parentheses ( ) [ ] and slash /
** alldelims: < > \backslash \setminus | \vert \mid \{ and \}
LaTeX Font Info:    Redeclaring math delimiter \backslash on input line
989.
LaTeX Font Info:    Redeclaring math symbol \setminus on input line 1001.
LaTeX Info: Redefining \models on input line 1010.
** \# \mathdollar \% \&
** \imath and \jmath
LaTeX Font Info:    Overwriting math alphabet `\mathnormalbold' in
version `nor
mal'
(Font)                  T1/Merriwthr-OsF/b/it --> T1/Merriwthr-OsF/b/it
on inpu
t line 2370.
LaTeX Font Info:    Overwriting math alphabet `\mathnormalbold' in
version `bol
d'
(Font)                  T1/Merriwthr-OsF/b/it --> T1/Merriwthr-OsF/b/it
on inpu
t line 2370.
```

```
LaTeX Font Info:    Overwriting symbol font `mtletterfont' in version
`normal'
(Font)                  T1/Merriwthr-OsF/m/it --> T1/Merriwthr-OsF/m/it
on inpu
t line 2370.
LaTeX Font Info:    Overwriting symbol font `mtletterfont' in version
`bold'
(Font)                  T1/Merriwthr-OsF/m/it --> T1/Merriwthr-OsF/b/it
on inpu
t line 2370.
LaTeX Font Info:    Overwriting symbol font `mtoperatorfont' in version
`normal
'
(Font)                  T1/Merriwthr-OsF/m/n --> T1/Merriwthr-OsF/m/n on
input
line 2370.
LaTeX Font Info:    Overwriting symbol font `mtoperatorfont' in version
`bold'
(Font)                  T1/Merriwthr-OsF/m/n --> T1/Merriwthr-OsF/b/n on
input
line 2370.
LaTeX Font Info:    Overwriting math alphabet `\Mathbf' in version
`normal'
(Font)                  T1/Merriwthr-OsF/b/n --> T1/Merriwthr-OsF/b/n on
input
line 2370.
LaTeX Font Info:    Overwriting math alphabet `\Mathbf' in version `bold'
(Font)                  T1/Merriwthr-OsF/b/n --> T1/Merriwthr-OsF/b/n on
input
line 2370.
LaTeX Font Info:    Overwriting math alphabet `\Mathit' in version
`normal'
(Font)                  T1/Merriwthr-OsF/m/it --> T1/Merriwthr-OsF/m/it
on inpu
t line 2370.
LaTeX Font Info:    Overwriting math alphabet `\Mathit' in version `bold'
(Font)                  T1/Merriwthr-OsF/m/it --> T1/Merriwthr-OsF/b/it
on inpu
t line 2370.
LaTeX Font Info:    Overwriting math alphabet `\Mathsf' in version
`normal'
(Font)                  T1/MerriwthrSans-OsF/m/n --> T1/MerriwthrSans-
OsF/m/n o
n input line 2370.
LaTeX Font Info:    Overwriting math alphabet `\Mathsf' in version `bold'
(Font)                  T1/MerriwthrSans-OsF/m/n --> T1/MerriwthrSans-
OsF/b/n o
n input line 2370.
LaTeX Font Info:    Overwriting math alphabet `\Mathtt' in version
`normal'
(Font)                  T1/lmtt/m/n --> T1/lmtt/m/n on input line 2370.
LaTeX Font Info:    Overwriting math alphabet `\Mathtt' in version `bold'
(Font)                  T1/lmtt/m/n --> T1/lmtt/b/n on input line 2370.
** Latin letters in the `normal' (resp. `bold') math versions are now
```

```
** set up to use the fonts T1/Merriwthr-OsF/m(b)/it
** Other characters (digits, ...) and \log-like names will be
** typeset with the n shape.
** \hbar
** minus as endash
** \HUGE has been (re)-defined.
** mathastext has declared larger sizes for subscripts.
** To keep LaTeX defaults, use option `defaultmathsizes'.
) (c:/TeXLive/2022/texmf-dist/tex/latex/relsize/relsize.sty
Package: relsize 2013/03/29 ver 4.1
) (c:/TeXLive/2022/texmf-dist/tex/latex/ragged2e/ragged2e.sty
Package: ragged2e 2023/02/25 v3.4 ragged2e Package
\CenteringLeftskip=\skip51
\RaggedLeftLeftskip=\skip52
\RaggedRightLeftskip=\skip53
\CenteringRightskip=\skip54
\RaggedLeftRightskip=\skip55
\RaggedRightRightskip=\skip56
\CenteringParfillskip=\skip57
\RaggedLeftParfillskip=\skip58
\RaggedRightParfillskip=\skip59
\JustifyingParfillskip=\skip60
\CenteringParindent=\skip61
\RaggedLeftParindent=\skip62
\RaggedRightParindent=\skip63
\JustifyingParindent=\skip64
) (c:/TeXLive/2022/texmf-dist/tex/latex/xcolor/xcolor.sty
Package: xcolor 2022/06/12 v2.14 LaTeX color extensions (UK)
(c:/TeXLive/2022/texmf-dist/tex/latex/graphics-cfg/color.cfg
File: color.cfg 2016/01/02 v1.6 sample color configuration
)
Package xcolor Info: Driver file: pdftex.def on input line 227.
(c:/TeXLive/2022/texmf-dist/tex/latex/graphics-def/pdftex.def
File: pdftex.def 2022/09/22 v1.2b Graphics/color driver for pdftex
) (c:/TeXLive/2022/texmf-dist/tex/latex/graphics/mathcolor.ltx)
Package xcolor Info: Model `cmy' substituted by `cmy0' on input line
1353.
Package xcolor Info: Model `hsb' substituted by `rgb' on input line 1357.
Package xcolor Info: Model `RGB' extended on input line 1369.
Package xcolor Info: Model `HTML' substituted by `rgb' on input line
1371.
Package xcolor Info: Model `Hsb' substituted by `hsb' on input line 1372.
Package xcolor Info: Model `tHsb' substituted by `hsb' on input line
1373.
Package xcolor Info: Model `HSB' substituted by `hsb' on input line 1374.
Package xcolor Info: Model `Gray' substituted by `gray' on input line
1375.
Package xcolor Info: Model `wave' substituted by `hsb' on input line
1376.
) (c:/TeXLive/2022/texmf-dist/tex/latex/colortbl/colortbl.sty
Package: colortbl 2022/06/20 v1.0f Color table columns (DPC)
(c:/TeXLive/2022/texmf-dist/tex/latex/tools/array.sty
Package: array 2022/09/04 v2.5g Tabular extension package (FMi)
\col@sep=\dimen142
```

```
\ar@mcellbox=\box52
\extrarowheight=\dimen143
\NC@list=\toks27
\extratabsurround=\skip65
\backup@length=\skip66
\ar@cellbox=\box53
)
\everycr=\toks28
\minrowclearance=\skip67
\rownum=\count197
) (c:/TeXLive/2022/texmf-dist/tex/latex/graphics/graphicx.sty
Package: graphicx 2021/09/16 v1.2d Enhanced LaTeX Graphics (DPC,SPQR)
(c:/TeXLive/2022/texmf-dist/tex/latex/graphics/graphics.sty
Package: graphics 2022/03/10 v1.4e Standard LaTeX Graphics (DPC,SPQR)
(c:/TeXLive/2022/texmf-dist/tex/latex/graphics/trig.sty
Package: trig 2021/08/11 v1.11 sin cos tan (DPC)
) (c:/TeXLive/2022/texmf-dist/tex/latex/graphics-cfg/graphics.cfg
File: graphics.cfg 2016/06/04 v1.11 sample graphics configuration
)
Package graphics Info: Driver file: pdftex.def on input line 107.
)
\Gin@req@height=\dimen144
\Gin@req@width=\dimen145
) (c:/TeXLive/2022/texmf-dist/tex/latex/xpatch/xpatch.sty
(c:/TeXLive/2022/texm
f-dist/tex/latex/l3kernel/expl3.sty
Package: expl3 2023-02-22 L3 programming layer (loader)
(c:/TeXLive/2022/texmf-dist/tex/latex/l3backend/l3backend-pdftex.def
File: l3backend-pdftex.def 2023-01-16 L3 backend support: PDF output
(pdfTeX)
\l__color_backend_stack_int=\count198
\l__pdf_internal_box=\box54
))
Package: xpatch 2020/03/25 v0.3a Extending etoolbox patching commands
(c:/TeXLive/2022/texmf-dist/tex/latex/l3packages/xparse/xparse.sty
Package: xparse 2023-02-02 L3 Experimental document command parser
)) (c:/TeXLive/2022/texmf-dist/tex/latex/environ/environ.sty
Package: environ 2014/05/04 v0.3 A new way to define environments
(c:/TeXLive/2022/texmf-dist/tex/latex/trimspaces/trimspaces.sty
Package: trimspaces 2009/09/17 v1.1 Trim spaces around a token list
)
\@envbody=\toks29
) (c:/TeXLive/2022/texmf-dist/tex/latex/lastpage/lastpage.sty
Package: lastpage 2023/03/07 v2.0a lastpage: 2.09 or 2e? (HMM)
(c:/TeXLive/2022/texmf-dist/tex/latex/lastpage/lastpage2e.sty
Package: lastpage2e 2023/03/07 v2.0a Decide which 2e lastpage version to
use (H
MM)
(c:/TeXLive/2022/texmf-dist/tex/latex/lastpage/lastpagemodern.sty
Package: lastpagemodern 2023-03-07 v2.0a Refers to last page's name (HMM;
JPG)
)
)) (c:/TeXLive/2022/texmf-dist/tex/latex/graphics/rotating.sty
Package: rotating 2016/08/11 v2.16d rotated objects in LaTeX
```

```
(c:/TeXLive/2022/texmf-dist/tex/latex/base/ifthen.sty
Package: ifthen 2022/04/13 v1.1d Standard LaTeX ifthen package (DPC)
)
\c@r@tfl@t=\count199
\rotFPtop=\skip68
\rotFPbot=\skip69
\rot@float@box=\box55
\rot@mess@toks=\toks30
) (c:/TeXLive/2022/texmf-dist/tex/latex/graphics/lscape.sty
Package: lscape 2020/05/28 v3.02 Landscape Pages (DPC)
) (c:/TeXLive/2022/texmf-dist/tex/latex/tools/afterpage.sty
Package: afterpage 2014/10/28 v1.08 After-Page Package (DPC)
\AP@output=\toks31
\AP@partial=\box56
\AP@footins=\box57
) (c:/TeXLive/2022/texmf-dist/tex/latex/textpos/textpos.sty
Package: textpos 2022/07/23 v1.10.1
Package textpos Info: choosing support for LaTeX3 on input line 60.
\TP@textbox=\box58
\TP@holdbox=\box59
\TPHorizModule=\dimen146
\TPVertModule=\dimen147
\TP@margin=\dimen148
\TP@absmargin=\dimen149
Grid set 16 x 16 = 37.34424pt x 52.81541pt
\TPboxrulesize=\dimen150
\TP@ox=\dimen151
\TP@oy=\dimen152
\TP@tbargs=\toks32
TextBlockOrigin set to 0pt x 0pt
) (c:/TeXLive/2022/texmf-dist/tex/latex/url/url.sty
\Urlmuskip=\muskip19
Package: url 2013/09/16  ver 3.4  Verb mode for urls, etc.
) (c:/TeXLive/2022/texmf-dist/tex/latex/newfloat/newfloat.sty
Package: newfloat 2019/09/02 v1.1l Defining new floating environments
(AR)
Package newfloat Info: `rotating' package detected.
) (c:/TeXLive/2022/texmf-dist/tex/latex/mdframed/mdframed.sty
Package: mdframed 2013/07/01 1.9b: mdframed
(c:/TeXLive/2022/texmf-dist/tex/latex/kvoptions/kvoptions.sty
Package: kvoptions 2022-06-15 v3.15 Key value format for package options
(HO)
(c:/TeXLive/2022/texmf-dist/tex/generic/ltxcmds/ltxcmds.sty
Package: ltxcmds 2020-05-10 v1.25 LaTeX kernel commands for general use
(HO)
) (c:/TeXLive/2022/texmf-dist/tex/latex/kvsetkeys/kvsetkeys.sty
Package: kvsetkeys 2022-10-05 v1.19 Key value parser (HO)
)) (c:/TeXLive/2022/texmf-dist/tex/latex/zref/zref-abspage.sty
Package: zref-abspage 2022-04-07 v2.34 Module abspage for zref (HO)
(c:/TeXLive/2022/texmf-dist/tex/latex/zref/zref-base.sty
Package: zref-base 2022-04-07 v2.34 Module base for zref (HO)
(c:/TeXLive/2022/texmf-dist/tex/generic/infwarerr/infwarerr.sty
Package: infwarerr 2019/12/03 v1.5 Providing info/warning/error messages
(HO)
```

```
) (c:/TeXLive/2022/texmf-dist/tex/generic/kvdefinekeys/kvdefinekeys.sty
Package: kvdefinekeys 2019-12-19 v1.6 Define keys (HO)
) (c:/TeXLive/2022/texmf-dist/tex/generic/pdftexcmds/pdftexcmds.sty
Package: pdftexcmds 2020-06-27 v0.33 Utility functions of pdfTeX for
LuaTeX (HO
)
Package pdftexcmds Info: \pdf@primitive is available.
Package pdftexcmds Info: \pdf@ifprimitive is available.
Package pdftexcmds Info: \pdfdraftmode found.
) (c:/TeXLive/2022/texmf-dist/tex/generic/etexcmds/etexcmds.sty
Package: etexcmds 2019/12/15 v1.7 Avoid name clashes with e-TeX commands
(HO)
) (c:/TeXLive/2022/texmf-dist/tex/latex/auxhook/auxhook.sty
Package: auxhook 2019-12-17 v1.6 Hooks for auxiliary files (HO)
)
Package zref Info: New property list: main on input line 767.
Package zref Info: New property: default on input line 768.
Package zref Info: New property: page on input line 769.
) (c:/TeXLive/2022/texmf-dist/tex/latex/base/atbegshi-ltx.sty
Package: atbegshi-ltx 2021/01/10 v1.0c Emulation of the original atbegshi
package with kernel methods
)
\c@abspage=\count266
Package zref Info: New property: abspage on input line 65.
) (c:/TeXLive/2022/texmf-dist/tex/latex/needspace/needspace.sty
Package: needspace 2010/09/12 v1.3d reserve vertical space
)
\mdf@templength=\skip70
\c@mdf@globalstyle@cnt=\count267
\mdf@skipabove@length=\skip71
\mdf@skipbelow@length=\skip72
\mdf@leftmargin@length=\skip73
\mdf@rightmargin@length=\skip74
\mdf@innerleftmargin@length=\skip75
\mdf@innerrightmargin@length=\skip76
\mdf@innertopmargin@length=\skip77
\mdf@innerbottommargin@length=\skip78
\mdf@splittopskip@length=\skip79
\mdf@splitbottomskip@length=\skip80
\mdf@outermargin@length=\skip81
\mdf@innermargin@length=\skip82
\mdf@linewidth@length=\skip83
\mdf@innerlinewidth@length=\skip84
\mdf@middlelinewidth@length=\skip85
\mdf@outerlinewidth@length=\skip86
\mdf@roundcorner@length=\skip87
\mdf@footenotedistance@length=\skip88
\mdf@userdefinedwidth@length=\skip89
\mdf@needspace@length=\skip90
\mdf@frametitleaboveskip@length=\skip91
\mdf@frametitlebelowskip@length=\skip92
\mdf@frametitlerulewidth@length=\skip93
\mdf@frametitleleftmargin@length=\skip94
\mdf@frametitlerightmargin@length=\skip95
```

```
\mdf@shadowsize@length=\skip96
\mdf@extratopheight@length=\skip97
\mdf@subtitleabovelinewidth@length=\skip98
\mdf@subtitlebelowlinewidth@length=\skip99
\mdf@subtitleaboveskip@length=\skip100
\mdf@subtitlebelowskip@length=\skip101
\mdf@subtitleinneraboveskip@length=\skip102
\mdf@subtitleinnerbelowskip@length=\skip103
\mdf@subsubtitleabovelinewidth@length=\skip104
\mdf@subsubtitlebelowlinewidth@length=\skip105
\mdf@subsubtitleaboveskip@length=\skip106
\mdf@subsubtitlebelowskip@length=\skip107
\mdf@subsubtitleinneraboveskip@length=\skip108
\mdf@subsubtitleinnerbelowskip@length=\skip109
(c:/TeXLive/2022/texmf-dist/tex/latex/mdframed/md-frame-0.mdf
File: md-frame-0.mdf 2013/07/01\ 1.9b: md-frame-0
)
\mdf@frametitlebox=\box60
\mdf@footnotebox=\box61
\mdf@splitbox@one=\box62
\mdf@splitbox@two=\box63
\mdf@splitbox@save=\box64
\mdfsplitboxwidth=\skip110
\mdfsplitboxtotalwidth=\skip111
\mdfsplitboxheight=\skip112
\mdfsplitboxdepth=\skip113
\mdfsplitboxtotalheight=\skip114
\mdfframetitleboxwidth=\skip115
\mdfframetitleboxtotalwidth=\skip116
\mdfframetitleboxheight=\skip117
\mdfframetitleboxdepth=\skip118
\mdfframetitleboxtotalheight=\skip119
\mdffootnoteboxwidth=\skip120
\mdffootnoteboxtotalwidth=\skip121
\mdffootnoteboxheight=\skip122
\mdffootnoteboxdepth=\skip123
\mdffootnoteboxtotalheight=\skip124
\mdftotallinewidth=\skip125
\mdfboundingboxwidth=\skip126
\mdfboundingboxtotalwidth=\skip127
\mdfboundingboxheight=\skip128
\mdfboundingboxdepth=\skip129
\mdfboundingboxtotalheight=\skip130
\mdf@freevspace@length=\skip131
\mdf@horizontalwidthofbox@length=\skip132
\mdf@verticalmarginwhole@length=\skip133
\mdf@horizontalspaceofbox=\skip134
\mdfsubtitleheight=\skip135
\mdfsubsubtitleheight=\skip136
\c@mdfcountframes=\count268

****** mdframed patching \endmdf@trivlist

****** -- success******
```

```
\mdf@envdepth=\count269
\c@mdf@env@i=\count270
\c@mdf@env@ii=\count271
\c@mdf@zref@counter=\count272
Package zref Info: New property: mdf@pagevalue on input line 895.
) (c:/TeXLive/2022/texmf-dist/tex/latex/titlesec/titlesec.sty
Package: titlesec 2021/07/05 v2.14 Sectioning titles
\ttl@box=\box65
\beforetitleunit=\skip137
\aftertitleunit=\skip138
\ttl@plus=\dimen153
\ttl@minus=\dimen154
\ttl@toksa=\toks33
\titlewidth=\dimen155
\titlewidthlast=\dimen156
\titlewidthfirst=\dimen157
) (c:/TeXLive/2022/texmf-dist/tex/latex/koma-script/scrextend.sty
Package: scrextend 2022/10/12 v3.38 KOMA-Script package (extend other
classes w
ith features of KOMA-Script classes)
(c:/TeXLive/2022/texmf-dist/tex/latex/koma-script/scrkbase.sty
Package: scrkbase 2022/10/12 v3.38 KOMA-Script package (KOMA-Script-
dependent b
asics and keyval usage)
(c:/TeXLive/2022/texmf-dist/tex/latex/koma-script/scrbase.sty
Package: scrbase 2022/10/12 v3.38 KOMA-Script package (KOMA-Script-
independent
basics and keyval usage)
(c:/TeXLive/2022/texmf-dist/tex/latex/koma-script/scrlfile.sty
Package: scrlfile 2022/10/12 v3.38 KOMA-Script package (file load hooks)
(c:/TeXLive/2022/texmf-dist/tex/latex/koma-script/scrlfile-hook.sty
Package: scrlfile-hook 2022/10/12 v3.38 KOMA-Script package (using LaTeX
hooks)

(c:/TeXLive/2022/texmf-dist/tex/latex/koma-script/scrlogo.sty
Package: scrlogo 2022/10/12 v3.38 KOMA-Script package (logo)
)))
Applying: [2021/05/01] Usage of raw or classic option list on input line
252.
Already applied: [0000/00/00] Usage of raw or classic option list on
input line
 368.
))
Package scrextend Info: unexpected definition of `\@makefnmark'.
(scrextend)             Trying to patch it on input line 1709.
Package scrextend Info: patch seems to be successfull on input line 1709.
)

LaTeX Font Warning: Font shape `T1/cmr/m/n' in size <7.5> not available
(Font)              size <7> substituted on input line 65.

(c:/TeXLive/2022/texmf-dist/tex/latex/tools/calc.sty
Package: calc 2017/05/25 v4.3 Infix arithmetic (KKT,FJ)
```

```
\calc@Acount=\count273
\calc@Bcount=\count274
\calc@Adimen=\dimen158
\calc@Bdimen=\dimen159
\calc@Askip=\skip139
\calc@Bskip=\skip140
LaTeX Info: Redefining \setlength on input line 80.
LaTeX Info: Redefining \addtolength on input line 81.
\calc@Ccount=\count275
\calc@Cskip=\skip141
) (c:/TeXLive/2022/texmf-dist/tex/latex/geometry/geometry.sty
Package: geometry 2020/01/02 v5.9 Page Geometry
(c:/TeXLive/2022/texmf-dist/tex/generic/iftex/ifvtex.sty
Package: ifvtex 2019/10/25 v1.7 ifvtex legacy package. Use iftex instead.
)
\Gm@cnth=\count276
\Gm@cntv=\count277
\c@Gm@tempcnt=\count278
\Gm@bindingoffset=\dimen160
\Gm@wd@mp=\dimen161
\Gm@odd@mp=\dimen162
\Gm@even@mp=\dimen163
\Gm@layoutwidth=\dimen164
\Gm@layoutheight=\dimen165
\Gm@layouthoffset=\dimen166
\Gm@layoutvoffset=\dimen167
\Gm@dimlist=\toks34
) (c:/TeXLive/2022/texmf-dist/tex/latex/hyperref/hyperref.sty
Package: hyperref 2023-02-07 v7.00v Hypertext links for LaTeX
(c:/TeXLive/2022/texmf-dist/tex/generic/pdfescape/pdfescape.sty
Package: pdfescape 2019/12/09 v1.15 Implements pdfTeX's escape features
(HO)
) (c:/TeXLive/2022/texmf-dist/tex/latex/hycolor/hycolor.sty
Package: hycolor 2020-01-27 v1.10 Color options for hyperref/bookmark
(HO)
) (c:/TeXLive/2022/texmf-dist/tex/latex/letltxmacro/letltxmacro.sty
Package: letltxmacro 2019/12/03 v1.6 Let assignment for LaTeX macros (HO)
) (c:/TeXLive/2022/texmf-dist/tex/latex/hyperref/nameref.sty
Package: nameref 2022-05-17 v2.50 Cross-referencing by name of section
(c:/TeXLive/2022/texmf-dist/tex/latex/refcount/refcount.sty
Package: refcount 2019/12/15 v3.6 Data extraction from label references
(HO)
) (c:/TeXLive/2022/texmf-
dist/tex/generic/gettitlestring/gettitlestring.sty
Package: gettitlestring 2019/12/15 v1.6 Cleanup title references (HO)
)
\c@section@level=\count279
)
\@linkdim=\dimen168
\Hy@linkcounter=\count280
\Hy@pagecounter=\count281
(c:/TeXLive/2022/texmf-dist/tex/latex/hyperref/pd1enc.def
File: pd1enc.def 2023-02-07 v7.00v Hyperref: PDFDocEncoding definition
(HO)
```

```
Now handling font encoding PD1 ...
... no UTF-8 mapping file for font encoding PD1
) (c:/TeXLive/2022/texmf-dist/tex/generic/intcalc/intcalc.sty
Package: intcalc 2019/12/15 v1.3 Expandable calculations with integers
(HO)
)
\Hy@SavedSpaceFactor=\count282
(c:/TeXLive/2022/texmf-dist/tex/latex/hyperref/puenc.def
File: puenc.def 2023-02-07 v7.00v Hyperref: PDF Unicode definition (HO)
Now handling font encoding PU ...
... no UTF-8 mapping file for font encoding PU
)
Package hyperref Info: Option `colorlinks' set `true' on input line 4060.
Package hyperref Info: Hyper figures OFF on input line 4177.
Package hyperref Info: Link nesting OFF on input line 4182.
Package hyperref Info: Hyper index ON on input line 4185.
Package hyperref Info: Plain pages OFF on input line 4192.
Package hyperref Info: Backreferencing OFF on input line 4197.
Package hyperref Info: Implicit mode ON; LaTeX internals redefined.
Package hyperref Info: Bookmarks ON on input line 4425.
\c@Hy@tempcnt=\count283
LaTeX Info: Redefining \url on input line 4763.
\XeTeXLinkMargin=\dimen169
(c:/TeXLive/2022/texmf-dist/tex/generic/bitset/bitset.sty
Package: bitset 2019/12/09 v1.3 Handle bit-vector datatype (HO)
(c:/TeXLive/2022/texmf-dist/tex/generic/bigintcalc/bigintcalc.sty
Package: bigintcalc 2019/12/15 v1.5 Expandable calculations on big
integers (HO
)
))
\Fld@menulength=\count284
\Field@Width=\dimen170
\Fld@charsize=\dimen171
Package hyperref Info: Hyper figures OFF on input line 6042.
Package hyperref Info: Link nesting OFF on input line 6047.
Package hyperref Info: Hyper index ON on input line 6050.
Package hyperref Info: backreferencing OFF on input line 6057.
Package hyperref Info: Link coloring ON on input line 6060.
Package hyperref Info: Link coloring with OCG OFF on input line 6067.
Package hyperref Info: PDF/A mode OFF on input line 6072.
\Hy@abspage=\count285
\c@Item=\count286
\c@Hfootnote=\count287
)
Package hyperref Info: Driver (autodetected): hpdftex.
(c:/TeXLive/2022/texmf-dist/tex/latex/hyperref/hpdftex.def
File: hpdftex.def 2023-02-07 v7.00v Hyperref driver for pdfTeX
(c:/TeXLive/2022/texmf-dist/tex/latex/base/atveryend-ltx.sty
Package: atveryend-ltx 2020/08/19 v1.0a Emulation of the original
atveryend pac
kage
with kernel methods
)
\HyAnn@Count=\count288
```

```
\Fld@listcount=\count289
\c@bookmark@seq@number=\count290
(c:/TeXLive/2022/texmf-dist/tex/latex/rerunfilecheck/rerunfilecheck.sty
Package: rerunfilecheck 2022-07-10 v1.10 Rerun checks for auxiliary files
(HO)
(c:/TeXLive/2022/texmf-dist/tex/generic/uniquecounter/uniquecounter.sty
Package: uniquecounter 2019/12/15 v1.4 Provide unlimited unique counter
(HO)
)
Package uniquecounter Info: New unique counter `rerunfilecheck' on input
line 2
85.
)
\Hy@SectionHShift=\skip142
) (c:/TeXLive/2022/texmf-dist/tex/latex/preprint/authblk.sty
Package: authblk 2001/02/27 1.3 (PWD)
\affilsep=\skip143
\@affilsep=\skip144
\c@Maxaffil=\count291
\c@authors=\count292
\c@affil=\count293
) (c:/TeXLive/2022/texmf-dist/tex/latex/footmisc/footmisc.sty
Package: footmisc 2022/03/08 v6.0d a miscellany of footnote facilities
\FN@temptoken=\toks35
\footnotemargin=\dimen172
\@outputbox@depth=\dimen173
Package footmisc Info: Declaring symbol style bringhurst on input line
695.
Package footmisc Info: Declaring symbol style chicago on input line 703.
Package footmisc Info: Declaring symbol style wiley on input line 712.
Package footmisc Info: Declaring symbol style lamport-robust on input
line 723.

Package footmisc Info: Declaring symbol style lamport* on input line 743.
Package footmisc Info: Declaring symbol style lamport*-robust on input
line 764
.
) (c:/TeXLive/2022/texmf-dist/tex/latex/fancyhdr/fancyhdr.sty
Package: fancyhdr 2022/11/09 v4.1 Extensive control of page headers and
footers

\f@nch@headwidth=\skip145
\f@nch@O@elh=\skip146
\f@nch@O@erh=\skip147
\f@nch@O@olh=\skip148
\f@nch@O@orh=\skip149
\f@nch@O@elf=\skip150
\f@nch@O@erf=\skip151
\f@nch@O@olf=\skip152
\f@nch@O@orf=\skip153
) (c:/TeXLive/2022/texmf-dist/tex/generic/alphalph/alphalph.sty
Package: alphalph 2019/12/09 v2.6 Convert numbers to letters (HO)
)
\c@authorfn=\count294
```

```
(c:/TeXLive/2022/texmf-dist/tex/latex/abstract/abstract.sty
Package: abstract 2009/06/08 v1.2a configurable abstracts
\abstitleskip=\skip154
\absleftindent=\skip155
\absrightindent=\skip156
\absparindent=\skip157
\absparsep=\skip158
)
Package newfloat Info: New float `keypoints' with options
`placement=t!,name=kp
t' on input line 286.
\c@keypoints=\count295
\newfloat@ftype=\count296
Package newfloat Info: float type `keypoints'=8 on input line 286.
(c:/TeXLive/2022/texmf-dist/tex/latex/enumitem/enumitem.sty
Package: enumitem 2019/06/20 v3.9 Customized lists
\labelindent=\skip159
\enit@outerparindent=\dimen174
\enit@toks=\toks36
\enit@inbox=\box66
\enit@count@id=\count297
\enitdp@description=\count298
) (c:/TeXLive/2022/texmf-dist/tex/latex/quoting/quoting.sty
Package: quoting 2014/01/28 v0.1c Consolidated environment for displayed
text
\quo@toppartop=\skip160
) (c:/TeXLive/2022/texmf-dist/tex/latex/sttools/stfloats.sty
Package: stfloats 2017/03/27 v3.3 Improve float mechanism and
baselineskip sett
ings
\@dblbotnum=\count299
\c@dblbotnumber=\count300
) (c:/TeXLive/2022/texmf-dist/tex/latex/booktabs/booktabs.sty
Package: booktabs 2020/01/12 v1.61803398 Publication quality tables
\heavyrulewidth=\dimen175
\lightrulewidth=\dimen176
\cmidrulewidth=\dimen177
\belowrulesep=\dimen178
\belowbottomsep=\dimen179
\aboverulesep=\dimen180
\abovetopsep=\dimen181
\cmidrulesep=\dimen182
\cmidrulekern=\dimen183
\defaultaddspace=\dimen184
\@cmidla=\count301
\@cmidlb=\count302
\@aboverulesep=\dimen185
\@belowrulesep=\dimen186
\@thisruleclass=\count303
\@lastruleclass=\count304
\@thisrulewidth=\dimen187
) (c:/TeXLive/2022/texmf-dist/tex/latex/tools/tabularx.sty
Package: tabularx 2020/01/15 v2.11c `tabularx' package (DPC)
\TX@col@width=\dimen188
```

```
\TX@old@table=\dimen189
\TX@old@col=\dimen190
\TX@target=\dimen191
\TX@delta=\dimen192
\TX@cols=\count305
\TX@ftn=\toks37
)
\enitdp@tablenotes=\count306
(c:/TeXLive/2022/texmf-dist/tex/latex/caption/caption.sty
Package: caption 2022/03/01 v3.6b Customizing captions (AR)
(c:/TeXLive/2022/texmf-dist/tex/latex/caption/caption3.sty
Package: caption3 2022/03/17 v2.3b caption3 kernel (AR)
\caption@tempdima=\dimen193
\captionmargin=\dimen194
\caption@leftmargin=\dimen195
\caption@rightmargin=\dimen196
\caption@width=\dimen197
\caption@indent=\dimen198
\caption@parindent=\dimen199
\caption@hangindent=\dimen256
Package caption Info: Standard document class detected.
)
\c@caption@flags=\count307
\c@continuedfloat=\count308
Package caption Info: hyperref package is loaded.
Package caption Info: rotating package is loaded.
) (c:/TeXLive/2022/texmf-dist/tex/latex/natbib/natbib.sty
Package: natbib 2010/09/13 8.31b (PWD, AO)
\bibhang=\skip161
\bibsep=\skip162
LaTeX Info: Redefining \cite on input line 694.
\c@NAT@ctr=\count309
)) (c:/TeXLive/2022/texmf-dist/tex/latex/siunitx/siunitx.sty
Package: siunitx 2023-03-04 v3.2.2 A comprehensive (SI) units package
\l__siunitx_angle_tmp_dim=\dimen257
\l__siunitx_angle_marker_box=\box67
\l__siunitx_angle_unit_box=\box68
\l__siunitx_compound_count_int=\count310
(c:/TeXLive/2022/texmf-dist/tex/latex/translations/translations.sty
Package: translations 2022/02/05 v1.12 internationalization of LaTeX2e
packages
 (CN)
)
\l__siunitx_number_exponent_fixed_int=\count311
\l__siunitx_number_min_decimal_int=\count312
\l__siunitx_number_min_integer_int=\count313
\l__siunitx_number_round_precision_int=\count314
\l__siunitx_number_lower_threshold_int=\count315
\l__siunitx_number_upper_threshold_int=\count316
\l__siunitx_number_group_first_int=\count317
\l__siunitx_number_group_size_int=\count318
\l__siunitx_number_group_minimum_int=\count319
(c:/TeXLive/2022/texmf-dist/tex/latex/amsmath/amstext.sty
Package: amstext 2021/08/26 v2.01 AMS text
```

```
(c:/TeXLive/2022/texmf-dist/tex/latex/amsmath/amsgen.sty
File: amsgen.sty 1999/11/30 v2.0 generic functions
\@emptytoks=\toks38
\ex@=\dimen258
))
\l__siunitx_table_tmp_box=\box69
\l__siunitx_table_tmp_dim=\dimen259
\l__siunitx_table_column_width_dim=\dimen260
\l__siunitx_table_integer_box=\box70
\l__siunitx_table_decimal_box=\box71
\l__siunitx_table_uncert_box=\box72
\l__siunitx_table_before_box=\box73
\l__siunitx_table_after_box=\box74
\l__siunitx_table_before_dim=\dimen261
\l__siunitx_table_carry_dim=\dimen262
\l__siunitx_unit_tmp_int=\count320
\l__siunitx_unit_position_int=\count321
\l__siunitx_unit_total_int=\count322
)
Package translations Info: No language package found. I am going to use
`englis
h' as default language. on input line 55.
LaTeX Font Info:    Trying to load font information for T1+Merriwthr-OsF
on inp
ut line 55.
(c:/TeXLive/2022/texmf-dist/tex/latex/merriweather/T1Merriwthr-OsF.fd
File: T1Merriwthr-OsF.fd 2020/08/30 (autoinst) Font definitions for
T1/Merriwth
r-OsF.
)
LaTeX Font Info:    Font shape `T1/Merriwthr-OsF/m/n' will be
(Font)              scaled to size 7.5pt on input line 55.
(./main.aux)
\openout1 = `main.aux'.

LaTeX Font Info:    Checking defaults for OML/cmm/m/it on input line 55.
LaTeX Font Info:    ... okay on input line 55.
LaTeX Font Info:    Checking defaults for OMS/cmsy/m/n on input line 55.
LaTeX Font Info:    ... okay on input line 55.
LaTeX Font Info:    Checking defaults for OT1/cmr/m/n on input line 55.
LaTeX Font Info:    ... okay on input line 55.
LaTeX Font Info:    Checking defaults for T1/cmr/m/n on input line 55.
LaTeX Font Info:    ... okay on input line 55.
LaTeX Font Info:    Checking defaults for TS1/cmr/m/n on input line 55.
LaTeX Font Info:    ... okay on input line 55.
LaTeX Font Info:    Checking defaults for OMX/cmex/m/n on input line 55.
LaTeX Font Info:    ... okay on input line 55.
LaTeX Font Info:    Checking defaults for U/cmr/m/n on input line 55.
LaTeX Font Info:    ... okay on input line 55.
LaTeX Font Info:    Checking defaults for PD1/pdf/m/n on input line 55.
LaTeX Font Info:    ... okay on input line 55.
LaTeX Font Info:    Checking defaults for PU/pdf/m/n on input line 55.
LaTeX Font Info:    ... okay on input line 55.
LaTeX Info: Redefining \microtypecontext on input line 55.
```

```
Package microtype Info: Applying patch `item' on input line 55.
Package microtype Info: Applying patch `toc' on input line 55.
Package microtype Info: Applying patch `eqnum' on input line 55.

Package microtype Warning: Unable to apply patch `footnote' on input line
55.

Package microtype Info: Applying patch `verbatim' on input line 55.
Package microtype Info: Generating PDF output.
Package microtype Info: Character protrusion enabled (level 2).
Package microtype Info: Using default protrusion set `alltext'.
Package microtype Info: Automatic font expansion enabled (level 2),
(microtype)             stretch: 20, shrink: 20, step: 1, non-selected.
Package microtype Info: Using default expansion set `alltext-nott'.
LaTeX Info: Redefining \showhyphens on input line 55.
Package microtype Info: No adjustment of tracking.
Package microtype Info: No adjustment of interword spacing.
Package microtype Info: No adjustment of character kerning.
Package microtype Info: Loading generic protrusion settings for font
family
(microtype)             `Merriwthr-OsF' (encoding: T1).
(microtype)             For optimal results, create family-specific
settings.
(microtype)             See the microtype manual for details.
LaTeX Font Info:    Redeclaring symbol font `operators' on input line 55.
LaTeX Font Info:    Encoding `OT1' has changed to `T1' for symbol font
(Font)              `operators' in the math version `normal' on input
line 55.
LaTeX Font Info:    Overwriting symbol font `operators' in version
`normal'
(Font)                  OT1/cmr/m/n --> T1/Merriwthr-OsF/m/up on input
line 55.

LaTeX Font Info:    Encoding `OT1' has changed to `T1' for symbol font
(Font)              `operators' in the math version `bold' on input line
55.
LaTeX Font Info:    Overwriting symbol font `operators' in version `bold'
(Font)                  OT1/cmr/bx/n --> T1/Merriwthr-OsF/m/up on input
line 55
.
LaTeX Font Info:    Overwriting symbol font `operators' in version `bold'
(Font)                  T1/Merriwthr-OsF/m/up --> T1/Merriwthr-OsF/b/up
on inpu
t line 55.
LaTeX Font Info:    Redeclaring math alphabet \mathbf on input line 55.
LaTeX Font Info:    Overwriting math alphabet `\mathbf' in version
`normal'
(Font)                  OT1/cmr/bx/n --> T1/Merriwthr-OsF/b/up on input
line 55
.
LaTeX Font Info:    Overwriting math alphabet `\mathbf' in version `bold'
(Font)                  OT1/cmr/bx/n --> T1/Merriwthr-OsF/b/up on input
line 55
.
```

```
LaTeX Font Info:    Redeclaring math alphabet \mathsf on input line 55.
LaTeX Font Info:    Overwriting math alphabet `\mathsf' in version
`normal'
(Font)                  OT1/cmss/m/n --> T1/MerriwthrSans-OsF/m/up on
input lin
e 55.
LaTeX Font Info:    Overwriting math alphabet `\mathsf' in version `bold'
(Font)                  OT1/cmss/bx/n --> T1/MerriwthrSans-OsF/m/up on
input li
ne 55.
LaTeX Font Info:    Redeclaring math alphabet \mathit on input line 55.
LaTeX Font Info:    Overwriting math alphabet `\mathit' in version
`normal'
(Font)                  OT1/cmr/m/it --> T1/Merriwthr-OsF/m/it on input
line 55
.
LaTeX Font Info:    Overwriting math alphabet `\mathit' in version `bold'
(Font)                  OT1/cmr/bx/it --> T1/Merriwthr-OsF/m/it on input
line 5
5.
LaTeX Font Info:    Redeclaring math alphabet \mathtt on input line 55.
LaTeX Font Info:    Overwriting math alphabet `\mathtt' in version
`normal'
(Font)                  OT1/cmtt/m/n --> T1/lmtt/m/up on input line 55.
LaTeX Font Info:    Overwriting math alphabet `\mathtt' in version `bold'
(Font)                  OT1/cmtt/m/n --> T1/lmtt/m/up on input line 55.
LaTeX Font Info:    Overwriting math alphabet `\mathsf' in version `bold'
(Font)                  T1/MerriwthrSans-OsF/m/up --> T1/MerriwthrSans-
OsF/b/up
 on input line 55.
LaTeX Font Info:    Overwriting math alphabet `\mathit' in version `bold'
(Font)                  T1/Merriwthr-OsF/m/it --> T1/Merriwthr-OsF/b/it
on inpu
t line 55.
\c@mv@tabular=\count323
\c@mv@boldtabular=\count324
(c:/TeXLive/2022/texmf-dist/tex/context/base/mkii/supp-pdf.mkii
[Loading MPS to PDF converter (version 2006.09.02).]
\scratchcounter=\count325
\scratchdimen=\dimen263
\scratchbox=\box75
\nofMPsegments=\count326
\nofMParguments=\count327
\everyMPshowfont=\toks39
\MPscratchCnt=\count328
\MPscratchDim=\dimen264
\MPnumerator=\count329
\makeMPintoPDFobject=\count330
\everyMPtoPDFconversion=\toks40
) (c:/TeXLive/2022/texmf-dist/tex/latex/epstopdf-pkg/epstopdf-base.sty
Package: epstopdf-base 2020-01-24 v2.11 Base part for package epstopdf
Package epstopdf-base Info: Redefining graphics rule for `.eps' on input
line 4
85.
```

```
(c:/TeXLive/2022/texmf-dist/tex/latex/latexconfig/epstopdf-sys.cfg
File: epstopdf-sys.cfg 2010/07/13 v1.3 Configuration of (r)epstopdf for
TeX Liv
e
))
*geometry* driver: auto-detecting
*geometry* detected driver: pdftex
*geometry* verbose mode - [ preamble ] result:
* driver: pdftex
* paper: a4paper
* layout: <same size as paper>
* layoutoffset:(h,v)=(0.0pt,0.0pt)
* modes: includefoot twoside
* h-part:(L,W,R)=(54.64pt, 488.22787pt, 54.64pt)
* v-part:(T,H,B)=(66.0pt, 745.04684pt, 34.0pt)
* \paperwidth=597.50787pt
* \paperheight=845.04684pt
* \textwidth=488.22787pt
* \textheight=715.04684pt
* \oddsidemargin=-17.62999pt
* \evensidemargin=-17.62999pt
* \topmargin=-47.76999pt
* \headheight=17.5pt
* \headsep=24.0pt
* \topskip=10.0pt
* \footskip=30.0pt
* \marginparwidth=48.0pt
* \marginparsep=10.0pt
* \columnsep=18.0pt
* \skip\footins=22.0pt plus 2.0pt
* \hoffset=0.0pt
* \voffset=0.0pt
* \mag=1000
* \@twocolumntrue
* \@twosidetrue
* \@mparswitchtrue
* \@reversemarginfalse
* (1in=72.27pt=25.4mm, 1cm=28.453pt)

Package hyperref Info: Link coloring ON on input line 55.
(./main.out) (./main.out)
\@outlinefile=\write3
\openout3 = `main.out'.

\@gscitedetails=\box76
\@gscitedetailsheight=\skip163
\@gsheadbox=\box77
\@gsheadboxheight=\skip164
LaTeX Font Info:    Font shape `T1/Merriwthr-OsF/b/n' will be
(Font)              scaled to size 6.5pt on input line 55.
LaTeX Font Info:    Calculating math sizes for size <7.5> on input line
55.

LaTeX Font Warning: Font shape `T1/Merriwthr-OsF/m/up' undefined
```

```
(Font)              using `T1/Merriwthr-OsF/m/n' instead on input line
55.

LaTeX Font Info:    Font shape `T1/Merriwthr-OsF/m/up' will be
(Font)              scaled to size 6.24973pt on input line 55.
LaTeX Font Info:    Font shape `T1/Merriwthr-OsF/m/up' will be
(Font)              scaled to size 5.24997pt on input line 55.
LaTeX Font Info:    Trying to load font information for U+eur on input
line 55.

(c:/TeXLive/2022/texmf-dist/tex/latex/amsfonts/ueur.fd
File: ueur.fd 2013/01/14 v3.01 Euler Roman
) (c:/TeXLive/2022/texmf-dist/tex/latex/microtype/mt-eur.cfg
File: mt-eur.cfg 2006/07/31 v1.1 microtype config. file: AMS Euler Roman
(RS)
)

LaTeX Font Warning: Font shape `OMS/cmsy/m/n' in size <7.5> not available
(Font)              size <7> substituted on input line 55.

LaTeX Font Info:    External font `cmex10' loaded for size
(Font)              <7.5> on input line 55.
LaTeX Font Info:    External font `cmex10' loaded for size
(Font)              <6.24973> on input line 55.
LaTeX Font Info:    External font `cmex10' loaded for size
(Font)              <5.24997> on input line 55.
LaTeX Font Info:    Trying to load font information for U+euf on input
line 55.

(c:/TeXLive/2022/texmf-dist/tex/latex/amsfonts/ueuf.fd
File: ueuf.fd 2013/01/14 v3.01 Euler Fraktur
) (c:/TeXLive/2022/texmf-dist/tex/latex/microtype/mt-euf.cfg
File: mt-euf.cfg 2006/07/03 v1.1 microtype config. file: AMS Euler
Fraktur (RS)

)
LaTeX Font Info:    Trying to load font information for U+eus on input
line 55.

(c:/TeXLive/2022/texmf-dist/tex/latex/amsfonts/ueus.fd
File: ueus.fd 2013/01/14 v3.01 Euler Script
) (c:/TeXLive/2022/texmf-dist/tex/latex/microtype/mt-eus.cfg
File: mt-eus.cfg 2006/07/28 v1.2 microtype config. file: AMS Euler Script
(RS)
)
LaTeX Font Info:    Trying to load font information for U+euex on input
line 55
.
(c:/TeXLive/2022/texmf-dist/tex/latex/amsfonts/ueuex.fd
File: ueuex.fd 2013/01/14 v3.01 Euler extra symbols
)

LaTeX Font Warning: Font shape `OML/cmm/m/it' in size <7.5> not available
(Font)              size <7> substituted on input line 55.
```

```
LaTeX Font Info:    Font shape `T1/Merriwthr-OsF/m/n' will be
(Font)              scaled to size 6.24973pt on input line 55.
LaTeX Font Info:    Font shape `T1/Merriwthr-OsF/m/n' will be
(Font)              scaled to size 5.24997pt on input line 55.
LaTeX Font Info:    Font shape `T1/Merriwthr-OsF/m/it' will be
(Font)              scaled to size 7.5pt on input line 55.
LaTeX Font Info:    Font shape `T1/Merriwthr-OsF/m/it' will be
(Font)              scaled to size 6.24973pt on input line 55.
LaTeX Font Info:    Font shape `T1/Merriwthr-OsF/m/it' will be
(Font)              scaled to size 5.24997pt on input line 55.
LaTeX Font Info:    Font shape `T1/Merriwthr-OsF/m/n' will be
(Font)              scaled to size 8.0pt on input line 55.
LaTeX Font Info:    Font shape `T1/Merriwthr-OsF/m/it' will be
(Font)              scaled to size 8.0pt on input line 55.
LaTeX Font Info:    Font shape `T1/Merriwthr-OsF/b/it' will be
(Font)              scaled to size 8.0pt on input line 55.
Package caption Info: Begin \AtBeginDocument code.
Package caption Info: End \AtBeginDocument code.

(c:/TeXLive/2022/texmf-dist/tex/latex/translations/translations-basic-
dictionar
y-english.trsl
File: translations-basic-dictionary-english.trsl (english translation
file `tra
nslations-basic-dictionary')
)
Package translations Info: loading dictionary `translations-basic-
dictionary' f
or `english'. on input line 55.
TextBlockOrigin set to 4pc+6.64pt x 4pc+6pt
<oup.pdf, id=113, 49.18375pt x 48.18pt>
File: oup.pdf Graphic file (type pdf)
<use oup.pdf>
Package pdftex.def Info: oup.pdf  used on input line 72.
(pdftex.def)             Requested size: 59.24683pt x 58.038pt.
<gigascience-logo.pdf, id=114, 99.37125pt x 33.12375pt>
File: gigascience-logo.pdf Graphic file (type pdf)
<use gigascience-logo.pdf>
Package pdftex.def Info: gigascience-logo.pdf  used on input line 72.
(pdftex.def)             Requested size: 126.00902pt x 42.0pt.

Overfull \hbox (54.64pt too wide) in paragraph at lines 72--72
[][]
 []

LaTeX Font Info:    Font shape `T1/Merriwthr-OsF/m/n' will be
(Font)              scaled to size 14.0pt on input line 72.
LaTeX Font Info:    Font shape `T1/Merriwthr-OsF/m/n' will be
(Font)              scaled to size 8.99997pt on input line 72.
LaTeX Font Info:    Calculating math sizes for size <14> on input line
72.
LaTeX Font Info:    Font shape `T1/Merriwthr-OsF/m/up' will be
(Font)              scaled to size 14.0pt on input line 72.
```

```
LaTeX Font Info:    Font shape `T1/Merriwthr-OsF/m/up' will be
(Font)              scaled to size 11.66617pt on input line 72.
LaTeX Font Info:    Font shape `T1/Merriwthr-OsF/m/up' will be
(Font)              scaled to size 9.79996pt on input line 72.
LaTeX Font Info:    External font `cmex10' loaded for size
(Font)              <14> on input line 72.
LaTeX Font Info:    External font `cmex10' loaded for size
(Font)              <11.66617> on input line 72.
LaTeX Font Info:    External font `cmex10' loaded for size
(Font)              <9.79996> on input line 72.
LaTeX Font Info:    Font shape `T1/Merriwthr-OsF/m/n' will be
(Font)              scaled to size 11.66617pt on input line 72.
LaTeX Font Info:    Font shape `T1/Merriwthr-OsF/m/n' will be
(Font)              scaled to size 9.79996pt on input line 72.
LaTeX Font Info:    Font shape `T1/Merriwthr-OsF/m/it' will be
(Font)              scaled to size 14.0pt on input line 72.
LaTeX Font Info:    Font shape `T1/Merriwthr-OsF/m/it' will be
(Font)              scaled to size 11.66617pt on input line 72.
LaTeX Font Info:    Font shape `T1/Merriwthr-OsF/m/it' will be
(Font)              scaled to size 9.79996pt on input line 72.
LaTeX Font Info:    Font shape `T1/Merriwthr-OsF/b/n' will be
(Font)              scaled to size 18.0pt on input line 72.
LaTeX Font Info:    Font shape `T1/Merriwthr-OsF/m/n' will be
(Font)              scaled to size 13.0pt on input line 72.
LaTeX Font Info:    Calculating math sizes for size <13> on input line
72.
LaTeX Font Info:    Font shape `T1/Merriwthr-OsF/m/up' will be
(Font)              scaled to size 13.0pt on input line 72.
LaTeX Font Info:    Font shape `T1/Merriwthr-OsF/m/up' will be
(Font)              scaled to size 10.83287pt on input line 72.
LaTeX Font Info:    Font shape `T1/Merriwthr-OsF/m/up' will be
(Font)              scaled to size 9.09996pt on input line 72.

LaTeX Font Warning: Font shape `OMS/cmsy/m/n' in size <13> not available
(Font)              size <12> substituted on input line 72.

LaTeX Font Info:    External font `cmex10' loaded for size
(Font)              <13> on input line 72.
LaTeX Font Info:    External font `cmex10' loaded for size
(Font)              <10.83287> on input line 72.
LaTeX Font Info:    External font `cmex10' loaded for size
(Font)              <9.09996> on input line 72.

LaTeX Font Warning: Font shape `OML/cmm/m/it' in size <13> not available
(Font)              size <12> substituted on input line 72.

LaTeX Font Info:    Font shape `T1/Merriwthr-OsF/m/n' will be
(Font)              scaled to size 10.83287pt on input line 72.
LaTeX Font Info:    Font shape `T1/Merriwthr-OsF/m/n' will be
(Font)              scaled to size 9.09996pt on input line 72.
LaTeX Font Info:    Font shape `T1/Merriwthr-OsF/m/it' will be
(Font)              scaled to size 13.0pt on input line 72.
LaTeX Font Info:    Font shape `T1/Merriwthr-OsF/m/it' will be
(Font)              scaled to size 10.83287pt on input line 72.
```

```
LaTeX Font Info:    Font shape `T1/Merriwthr-OsF/m/it' will be
(Font)              scaled to size 9.09996pt on input line 72.
LaTeX Font Info:    Trying to load font information for TS1+Merriwthr-OsF
on in
put line 72.
(c:/TeXLive/2022/texmf-dist/tex/latex/merriweather/TS1Merriwthr-OsF.fd
File: TS1Merriwthr-OsF.fd 2020/08/30 (autoinst) Font definitions for
TS1/Merriw
thr-OsF.
)
LaTeX Font Info:    Font shape `TS1/Merriwthr-OsF/m/n' will be
(Font)              scaled to size 10.83287pt on input line 72.
Package microtype Info: Loading generic protrusion settings for font
family
(microtype)             `Merriwthr-OsF' (encoding: TS1).
(microtype)             For optimal results, create family-specific
settings.
(microtype)             See the microtype manual for details.
LaTeX Font Info:    Font shape `T1/Merriwthr-OsF/m/n' will be
(Font)              scaled to size 9.0pt on input line 72.
LaTeX Font Info:    Font shape `T1/Merriwthr-OsF/m/up' will be
(Font)              scaled to size 9.0pt on input line 72.
LaTeX Font Info:    Font shape `T1/Merriwthr-OsF/m/up' will be
(Font)              scaled to size 7.0pt on input line 72.
LaTeX Font Info:    Font shape `T1/Merriwthr-OsF/m/up' will be
(Font)              scaled to size 5.0pt on input line 72.
LaTeX Font Info:    External font `cmex10' loaded for size
(Font)              <9> on input line 72.
LaTeX Font Info:    External font `cmex10' loaded for size
(Font)              <7> on input line 72.
LaTeX Font Info:    External font `cmex10' loaded for size
(Font)              <5> on input line 72.
LaTeX Font Info:    Font shape `T1/Merriwthr-OsF/m/n' will be
(Font)              scaled to size 7.0pt on input line 72.
LaTeX Font Info:    Font shape `T1/Merriwthr-OsF/m/n' will be
(Font)              scaled to size 5.0pt on input line 72.
LaTeX Font Info:    Font shape `T1/Merriwthr-OsF/m/it' will be
(Font)              scaled to size 9.0pt on input line 72.
LaTeX Font Info:    Font shape `T1/Merriwthr-OsF/m/it' will be
(Font)              scaled to size 7.0pt on input line 72.
LaTeX Font Info:    Font shape `T1/Merriwthr-OsF/m/it' will be
(Font)              scaled to size 5.0pt on input line 72.
LaTeX Font Info:    Font shape `T1/Merriwthr-OsF/m/n' will be
(Font)              scaled to size 6.5pt on input line 72.
LaTeX Font Info:    Calculating math sizes for size <6.5> on input line
72.
LaTeX Font Info:    Font shape `T1/Merriwthr-OsF/m/up' will be
(Font)              scaled to size 6.5pt on input line 72.
LaTeX Font Info:    Font shape `T1/Merriwthr-OsF/m/up' will be
(Font)              scaled to size 5.41643pt on input line 72.
LaTeX Font Info:    Font shape `T1/Merriwthr-OsF/m/up' will be
(Font)              scaled to size 4.54997pt on input line 72.

LaTeX Font Warning: Font shape `OMS/cmsy/m/n' in size <6.5> not available
```

```
(Font)              size <6> substituted on input line 72.


LaTeX Font Warning: Font shape `OMS/cmsy/m/n' in size <5.41643> not
available
(Font)              size <5> substituted on input line 72.


LaTeX Font Warning: Font shape `OMS/cmsy/m/n' in size <4.54997> not
available
(Font)              size <5> substituted on input line 72.

LaTeX Font Info:    External font `cmex10' loaded for size
(Font)              <6.5> on input line 72.
LaTeX Font Info:    External font `cmex10' loaded for size
(Font)              <5.41643> on input line 72.
LaTeX Font Info:    External font `cmex10' loaded for size
(Font)              <4.54997> on input line 72.

LaTeX Font Warning: Font shape `OML/cmm/m/it' in size <6.5> not available
(Font)              size <6> substituted on input line 72.


LaTeX Font Warning: Font shape `OML/cmm/m/it' in size <5.41643> not
available
(Font)              size <5> substituted on input line 72.


LaTeX Font Warning: Font shape `OML/cmm/m/it' in size <4.54997> not
available
(Font)              size <5> substituted on input line 72.

LaTeX Font Info:    Font shape `T1/Merriwthr-OsF/m/n' will be
(Font)              scaled to size 5.41643pt on input line 72.
LaTeX Font Info:    Font shape `T1/Merriwthr-OsF/m/n' will be
(Font)              scaled to size 4.54997pt on input line 72.
LaTeX Font Info:    Font shape `T1/Merriwthr-OsF/m/it' will be
(Font)              scaled to size 6.5pt on input line 72.
LaTeX Font Info:    Font shape `T1/Merriwthr-OsF/m/it' will be
(Font)              scaled to size 5.41643pt on input line 72.
LaTeX Font Info:    Font shape `T1/Merriwthr-OsF/m/it' will be
(Font)              scaled to size 4.54997pt on input line 72.
LaTeX Font Info:    Font shape `TS1/Merriwthr-OsF/m/n' will be
(Font)              scaled to size 5.41643pt on input line 72.

Overfull \hbox (54.64pt too wide) in paragraph at lines 72--72
[][][]
 []

LaTeX Font Info:    Font shape `T1/Merriwthr-OsF/b/n' will be
(Font)              scaled to size 10.0pt on input line 72.
LaTeX Font Info:    Font shape `T1/Merriwthr-OsF/b/n' will be
(Font)              scaled to size 8.0pt on input line 72.
```

```
Overfull \hbox (54.64pt too wide) in paragraph at lines 72--72
[][][]
 []

Package mdframed Info: mdframed works in twoside mode on input line 75.
LaTeX Font Info:    Font shape `T1/Merriwthr-OsF/b/n' will be
(Font)              scaled to size 8.2pt on input line 75.
LaTeX Font Info:    Font shape `TS1/Merriwthr-OsF/m/n' will be
(Font)              scaled to size 7.5pt on input line 77.
Package mdframed Info: mdframed inside float
 mdframed uses option nobreak mdframed on input line 84.
Package mdframed Info: mdframed inside a box
 mdframed uses option nobreak mdframed on input line 84.
LaTeX Font Info:    Font shape `T1/Merriwthr-OsF/b/n' will be
(Font)              scaled to size 7.5pt on input line 88.

Package natbib Warning: Citation `giani2020long' on page 1 undefined on
input l
ine 88.


Package natbib Warning: Citation `lewin2018earth' on page 1 undefined on
input
line 88.


Package natbib Warning: Citation `bordon2020animal' on page 1 undefined
on inpu
t line 88.


Package natbib Warning: Citation `casewell2020causes' on page 1 undefined
on in
put line 88.


Package natbib Warning: Citation `barua2021ancient' on page 1 undefined
on inpu
t line 88.


Package natbib Warning: Citation `perry2022snake' on page 1 undefined on
input
line 88.


Package natbib Warning: Citation `zancolli2022convergent' on page 1
undefined o
n input line 88.


Package natbib Warning: Citation `zancolli2020venom' on page 1 undefined
on inp
ut line 88.
```

```
Underfull \vbox (badness 10000) has occurred while \output is active []


Underfull \vbox (badness 10000) has occurred while \output is active []

LaTeX Font Info:    Font shape `T1/Merriwthr-OsF/m/n' will be
(Font)              scaled to size 7.8pt on input line 89.
LaTeX Font Info:    Font shape `T1/Merriwthr-OsF/b/n' will be
(Font)              scaled to size 7.8pt on input line 89.
[1{c:/TeXLive/2022/texmf-var/fonts/map/pdftex/updmap/pdftex.map}


 <./oup.pdf> <./gigascience-logo.pdf>]

Package natbib Warning: Citation `drukewitzVonreumont2019' on page 2
undefined
on input line 90.


Package natbib Warning: Citation `fry2009evolution' on page 2 undefined
on inpu
t line 90.


Package natbib Warning: Citation `casewell2013complex' on page 2
undefined on i
nput line 90.


Package natbib Warning: Citation `zancolli2020venom' on page 2 undefined
on inp
ut line 90.


Package natbib Warning: Citation `casewell2013complex' on page 2
undefined on i
nput line 90.


Package natbib Warning: Citation `fry2006early' on page 2 undefined on
input li
ne 90.


Package natbib Warning: Citation `fry2009evolution' on page 2 undefined
on inpu
t line 90.


Package natbib Warning: Citation `bordon2020animal' on page 2 undefined
on inpu
t line 90.
```

Package natbib Warning: Citation `casewell2020causes' on page 2 undefined on in
put line 90.

Package natbib Warning: Citation `post2020snake' on page 2 undefined on input l
ine 90.

Package natbib Warning: Citation `radis2021cell' on page 2 undefined on input l
ine 90.

Package natbib Warning: Citation `drukewitzVonreumont2019' on page 2 undefined
on input line 92.

Package natbib Warning: Citation `vonk2013king' on page 2 undefined on input li
ne 92.

Package natbib Warning: Citation `yin2016evolutionary' on page 2 undefined on i
nput line 92.

Package natbib Warning: Citation `schield2019Cviridis' on page 2 undefined on i
nput line 92.

Package natbib Warning: Citation `peng2020Hcurtus' on page 2 undefined on input
 line 92.

Package natbib Warning: Citation `almeida2021jararaca' on page 2 undefined on i
nput line 92.

Package natbib Warning: Citation `myers2022Afeae' on page 2 undefined on input
line 92.

Package natbib Warning: Citation `zhang2022bungarus' on page 2 undefined on inp

ut line 92.

Package natbib Warning: Citation `yandell2012beginner' on page 2 undefined on i
nput line 94.

Package natbib Warning: Citation `richardson2013prokaryotic' on page 2 undefine
d on input line 94.

Package natbib Warning: Citation `salzberg2019' on page 2 undefined on input li
ne 94.

Package natbib Warning: Citation `ejigu2020review' on page 2 undefined on input
 line 96.

Package natbib Warning: Citation `stanke2003augustus' on page 2 undefined on in
put line 96.

Package natbib Warning: Citation `korf2004snap' on page 2 undefined on input li
ne 96.

Package natbib Warning: Citation `hoff2019braker' on page 2 undefined on input
line 96.

Package natbib Warning: Citation `bruuna2021braker2' on page 2 undefined on inp
ut line 96.

Package natbib Warning: Citation `cantarel2008maker' on page 2 undefined on inp
ut line 96.

LaTeX Font Info: Font shape `T1/Merriwthr-OsF/m/up' will be
(Font)   scaled to size 7.5pt on input line 96.

Package natbib Warning: Citation `bruuna2020genemark' on page 2 undefined on in
put line 96.

```
Package natbib Warning: Citation `solovyev2006fgenesh' on page 2
undefined on i
nput line 96.


Package natbib Warning: Citation `keilwagen2019gemoma' on page 2
undefined on i
nput line 96.


Package natbib Warning: Citation `shumate2021liftoff' on page 2 undefined
on in
put line 96.


Package natbib Warning: Citation `kirilenko2022integrating' on page 2
undefined
 on input line 96.


Package natbib Warning: Citation `lu2018removing' on page 2 undefined on
input
line 96.


Package natbib Warning: Citation `mudge2016state' on page 2 undefined on
input
line 96.


Package natbib Warning: Citation `peel2022best' on page 2 undefined on
input li
ne 96.


Package natbib Warning: Citation `frankish2019gencode' on page 2
undefined on i
nput line 96.


Package natbib Warning: Citation `salzberg2019' on page 2 undefined on
input li
ne 96.


Package natbib Warning: Citation `vonReumont2022venomics' on page 2
undefined o
n input line 98.


Package natbib Warning: Citation `almeida2021jararaca' on page 2
undefined on i
nput line 98.
```

Package natbib Warning: Citation `dowell2016deep' on page 2 undefined on
input
line 98.

Package natbib Warning: Citation `giorgianni2020svmp' on page 2 undefined
on in
put line 98.

Package natbib Warning: Citation `almeida2021jararaca' on page 2
undefined on i
nput line 98.

Package natbib Warning: Citation `cao2013mesobuthus' on page 2 undefined
on inp
ut line 98.

Package natbib Warning: Citation `drukewitz2019dipteraVenom' on page 2
undefine
d on input line 98.

Package natbib Warning: Citation `almeida2021jararaca' on page 2
undefined on i
nput line 98.

Package natbib Warning: Citation `smith2023micro' on page 2 undefined on
input
line 98.

Package natbib Warning: Citation `dowell2016deep' on page 2 undefined on
input
line 98.

Package natbib Warning: Citation `giorgianni2020svmp' on page 2 undefined
on in
put line 98.

Package natbib Warning: Citation `koludarov2020familysaga' on page 2
undefined
on input line 98.

Package natbib Warning: Citation `barua2021svsp' on page 2 undefined on
input l

ine 98.

Package natbib Warning: Citation `zhang2022bungarus' on page 2 undefined on inp
ut line 98.

Package natbib Warning: Citation `smith2023micro' on page 2 undefined on input
line 98.

Package natbib Warning: Citation `margres2021tigris' on page 2 undefined on inp
ut line 98.

Package natbib Warning: Citation `smith2023micro' on page 2 undefined on input
line 98.

Package natbib Warning: Citation `dowell2016deep' on page 2 undefined on input
line 98.

Package natbib Warning: Citation `giorgianni2020svmp' on page 2 undefined on in
put line 98.

Package natbib Warning: Citation `smith2023micro' on page 2 undefined on input
line 98.

Package natbib Warning: Citation `cao2013mesobuthus' on page 2 undefined on inp
ut line 98.

Package natbib Warning: Citation `vonk2013king' on page 2 undefined on input li
ne 98.

Package natbib Warning: Citation `yin2016evolutionary' on page 2 undefined on i
nput line 98.

```
Package natbib Warning: Citation `drukewitz2019dipteraVenom' on page 2
undefine
d on input line 98.


Package natbib Warning: Citation `schield2019Cviridis' on page 2
undefined on i
nput line 98.


Package natbib Warning: Citation `almeida2021jararaca' on page 2
undefined on i
nput line 98.


Package natbib Warning: Citation `margres2021tigris' on page 2 undefined
on inp
ut line 98.


Package natbib Warning: Citation `zhang2022bungarus' on page 2 undefined
on inp
ut line 98.


Package natbib Warning: Citation `ye2022waspsgenome' on page 2 undefined
on inp
ut line 98.


Package natbib Warning: Citation `smith2023micro' on page 2 undefined on
input
line 98.


Underfull \vbox (badness 10000) has occurred while \output is active []

LaTeX Font Info:    Font shape `T1/Merriwthr-OsF/m/it' will be
(Font)              scaled to size 7.8pt on input line 99.
[2]
LaTeX Font Info:    Font shape `T1/Merriwthr-OsF/b/n' will be
(Font)              scaled to size 8.5pt on input line 104.

Package natbib Warning: Citation `slater2005exonerate' on page 3
undefined on i
nput line 106.


Package natbib Warning: Citation `nachtigall2021toxcodan' on page 3
undefined o
n input line 108.
```

```
Package natbib Warning: Citation `macrander2018venomix' on page 3
undefined on
input line 108.


Package natbib Warning: Citation `holding2018evaluating' on page 3
undefined on
 input line 108.


Package natbib Warning: Citation `kim2019hisat' on page 3 undefined on
input li
ne 108.


Package natbib Warning: Citation `pertea2015stringtie' on page 3
undefined on i
nput line 108.


Package natbib Warning: Citation `haas2013trinity' on page 3 undefined on
input
 line 108.


Package natbib Warning: Citation `bushmanova2019rnaspades' on page 3
undefined
on input line 108.

<toxcodan_genome_workflow.pdf, id=146, 1662.21pt x 867.24pt>
File: toxcodan_genome_workflow.pdf Graphic file (type pdf)
<use toxcodan_genome_workflow.pdf>
Package pdftex.def Info: toxcodan_genome_workflow.pdf  used on input line
114.
(pdftex.def)             Requested size: 488.22787pt x 254.72202pt.
LaTeX Font Info:    Font shape `T1/Merriwthr-OsF/m/n' will be
(Font)              scaled to size 6.0pt on input line 115.
LaTeX Font Info:    Font shape `T1/Merriwthr-OsF/b/n' will be
(Font)              scaled to size 6.0pt on input line 115.

Package natbib Warning: Citation `ToxCodanGenomeGuide' on page 3
undefined on i
nput line 122.


Package natbib Warning: Citation `sayers2023ncbi' on page 3 undefined on
input
line 126.


Package natbib Warning: Citation `fu2012cdhit' on page 3 undefined on
input lin
e 126.
```

```
Package natbib Warning: Citation `king2008rational' on page 3 undefined
on inpu
t line 126.


Package natbib Warning: Citation `VenomZone' on page 3 undefined on input
line
126.


Package natbib Warning: Citation `jungo2012toxprot' on page 3 undefined
on inpu
t line 126.


Package natbib Warning: Citation `kaas2012conoserver' on page 3 undefined
on in
put line 126.


Package natbib Warning: Citation `pineda2018arachnoserver' on page 3
undefined
on input line 126.

[3]

Package natbib Warning: Citation `kirchhoff2022stingray' on page 4
undefined on
 input line 130.

LaTeX Font Info:    Font shape `T1/Merriwthr-OsF/b/n' will be
(Font)              scaled to size 7.0pt on input line 138.

Package natbib Warning: Citation `myers2022Afeae' on page 4 undefined on
input
line 144.


Package natbib Warning: Citation `almeida2021jararaca' on page 4
undefined on i
nput line 145.


Package natbib Warning: Citation `margres2021tigris' on page 4 undefined
on inp
ut line 146.


Package natbib Warning: Citation `peng2020Hcurtus' on page 4 undefined on
input
 line 148.
```

```
Package natbib Warning: Citation `li2021seaSnakes' on page 4 undefined on
input
 line 149.


Package natbib Warning: Citation `suryamohan2020indian' on page 4
undefined on
input line 150.


Package natbib Warning: Citation `zhou2022Pleopoldi' on page 4 undefined
on inp
ut line 152.


Package natbib Warning: Citation `cao2013mesobuthus' on page 4 undefined
on inp
ut line 154.


Package natbib Warning: Citation `park2015Acerana' on page 4 undefined on
input
 line 155.


Package natbib Warning: Citation `wallberg2019Amellifera' on page 4
undefined o
n input line 156.


Package natbib Warning: Citation `deGraaf2010nasonia' on page 4 undefined
on in
put line 157.


Package natbib Warning: Citation `fletcher2023genome' on page 4 undefined
on in
put line 158.


Package natbib Warning: Citation `li2011rsem' on page 4 undefined on
input line
 168.

[4 <./toxcodan_genome_workflow.pdf>]
LaTeX Font Info:    Font shape `T1/Merriwthr-OsF/b/it' will be
(Font)              scaled to size 8.5pt on input line 176.

Package natbib Warning: Citation `martins2002ecological' on page 5
undefined on
 input line 178.
```

```
Package natbib Warning: Citation `nogueira2019atlas' on page 5 undefined
on inp
ut line 178.


Package natbib Warning: Citation `cardoso2010alternatus' on page 5
undefined on
 input line 178.


Package natbib Warning: Citation `dePaula2014alternatus' on page 5
undefined on
 input line 178.


Package natbib Warning: Citation `nachtigall2021toxcodan' on page 5
undefined o
n input line 178.


Package natbib Warning: Citation `ToxCodanGenomeGuide' on page 5
undefined on i
nput line 180.

LaTeX Font Info:    Font shape `T1/Merriwthr-OsF/b/sl' in size <7.5> not
availa
ble
(Font)              Font shape `T1/Merriwthr-OsF/b/it' tried instead on
input l
ine 183.
LaTeX Font Info:    Font shape `T1/Merriwthr-OsF/b/it' will be
(Font)              scaled to size 7.5pt on input line 183.

Package natbib Warning: Citation `margres2021tigris' on page 5 undefined
on inp
ut line 185.


Package natbib Warning: Citation `nachtigall2021toxcodan' on page 5
undefined o
n input line 187.


Underfull \vbox (badness 2941) has occurred while \output is active []


Package natbib Warning: Citation `cheng2021hifiasm' on page 5 undefined
on inpu
t line 193.


Package natbib Warning: Citation `chen2021inspector' on page 5 undefined
on inp
ut line 193.
```

```
Package natbib Warning: Citation `waterhouse2018busco' on page 5
undefined on i
nput line 193.


Package natbib Warning: Citation `ou2019edta' on page 5 undefined on
input line
 193.


Package natbib Warning: Citation `nachtigall2021toxcodan' on page 5
undefined o
n input line 197.


Package natbib Warning: Citation `almeida2021jararaca' on page 5
undefined on i
nput line 197.


Package natbib Warning: Citation `freitas2020jararacussu' on page 5
undefined o
n input line 197.


Package natbib Warning: Citation `nachtigall2022differences' on page 5
undefine
d on input line 197.


Package natbib Warning: Citation `solovyev2006fgenesh' on page 5
undefined on i
nput line 197.


Package natbib Warning: Citation `funannotate' on page 5 undefined on
input lin
e 199.


Package natbib Warning: Citation `jones2014interproscan' on page 5
undefined on
 input line 199.


Package natbib Warning: Citation `rozewicki2019mafft' on page 5 undefined
on in
put line 201.


Package natbib Warning: Citation `nguyen2015iqtree' on page 5 undefined
on inpu
```

```
t line 201.


Package natbib Warning: Citation `figtree' on page 5 undefined on input line 20
1.

LaTeX Font Info:    Font shape `T1/Merriwthr-OsF/m/n' will be
(Font)              scaled to size 10.0pt on input line 204.
[5]

Package natbib Warning: Citation `drukewitzVonreumont2019' on page 6 undefined
on input line 210.


! LaTeX Error: File `\centering ' not found.

See the LaTeX manual or LaTeX Companion for explanation.
Type  H <return>  for immediate help.
 ...

l.216   \centering

I could not locate the file with any of these extensions:
.pdf,.png,.jpg,.mps,.jpeg,.jbig2,.jb2,.PDF,.PNG,.JPG,.JPEG,.JBIG2,.JB2,.e
ps
Try typing  <return>  to proceed.
If that doesn't work, type  X <return>  to quit.

<Figure2_tgtests.pdf, id=187, 615.57536pt x 931.40103pt>
File: Figure2_tgtests.pdf Graphic file (type pdf)
<use Figure2_tgtests.pdf>
Package pdftex.def Info: Figure2_tgtests.pdf  used on input line 217.
(pdftex.def)             Requested size: 463.81499pt x 701.7894pt.
LaTeX Font Info:    Font shape `T1/Merriwthr-OsF/m/it' will be
(Font)              scaled to size 6.0pt on input line 218.

LaTeX Warning: Float too large for page by 50.74255pt on input line 221.


Package natbib Warning: Citation `kirchhoff2022stingray' on page 6 undefined on
 input line 224.


Package natbib Warning: Citation `cao2013mesobuthus' on page 6 undefined on inp
ut line 226.


Package natbib Warning: Citation `peel2022best' on page 6 undefined on input li
ne 226.
```

```
Package natbib Warning: Citation `smith2023micro' on page 6 undefined on
input
line 230.


Package natbib Warning: Citation `smith2023micro' on page 6 undefined on
input
line 230.


Package natbib Warning: Citation `smith2023micro' on page 6 undefined on
input
line 230.


! LaTeX Error: File `\centering ' not found.

See the LaTeX manual or LaTeX Companion for explanation.
Type  H <return>  for immediate help.
 ...

l.238   \centering

I could not locate the file with any of these extensions:
.pdf,.png,.jpg,.mps,.jpeg,.jbig2,.jb2,.PDF,.PNG,.JPG,.JPEG,.JBIG2,.JB2,.e
ps
Try typing  <return>  to proceed.
If that doesn't work, type  X <return>  to quit.

<Figure_3_tgtests.pdf, id=192, 615.57536pt x 922.86511pt>
File: Figure_3_tgtests.pdf Graphic file (type pdf)
<use Figure_3_tgtests.pdf>
Package pdftex.def Info: Figure_3_tgtests.pdf  used on input line 239.
(pdftex.def)             Requested size: 463.81499pt x 695.35779pt.

LaTeX Warning: Float too large for page by 44.31094pt on input line 243.


Package natbib Warning: Citation `myers2022Afeae' on page 6 undefined on
input
line 250.

[6]

Package natbib Warning: Citation `almeida2021jararaca' on page 7
undefined on i
nput line 252.


Package natbib Warning: Citation `almeida2021jararaca' on page 7
undefined on i
nput line 252.
```

```
Underfull \hbox (badness 1383) in paragraph at lines 252--253
\T1/Merriwthr-OsF/m/up/7.5 (+20) par-tial in the pub-lished an-no-ta-tion
(\T1/
Merriwthr-OsF/m/it/7.5 (+20) i.e.\T1/Merriwthr-OsF/m/up/7.5 (+20) ,
KAG5858131.
1_SVMP
 []


! LaTeX Error: File `\centering ' not found.

See the LaTeX manual or LaTeX Companion for explanation.
Type  H <return>  for immediate help.
 ...

l.263   \centering

I could not locate the file with any of these extensions:
.pdf,.png,.jpg,.mps,.jpeg,.jbig2,.jb2,.PDF,.PNG,.JPG,.JPEG,.JBIG2,.JB2,.e
ps
Try typing  <return>  to proceed.
If that doesn't work, type  X <return>  to quit.

<Fig_proof_concept.pdf, id=204, 682.95152pt x 704.6325pt>
File: Fig_proof_concept.pdf Graphic file (type pdf)
<use Fig_proof_concept.pdf>
Package pdftex.def Info: Fig_proof_concept.pdf  used on input line 264.
(pdftex.def)             Requested size: 463.81499pt x 478.54167pt.

Package natbib Warning: Citation `almeida2021jararaca' on page 7
undefined on i
nput line 276.


Package natbib Warning: Citation `schield2019Cviridis' on page 7
undefined on i
nput line 276.


Package natbib Warning: Citation `margres2021tigris' on page 7 undefined
on inp
ut line 276.


Package natbib Warning: Citation `myers2022Afeae' on page 7 undefined on
input
line 276.


! LaTeX Error: File `\centering ' not found.

See the LaTeX manual or LaTeX Companion for explanation.
```

```
Type  H <return>  for immediate help.
 ...

l.283   \centering

I could not locate the file with any of these extensions:
.pdf,.png,.jpg,.mps,.jpeg,.jbig2,.jb2,.PDF,.PNG,.JPG,.JPEG,.JBIG2,.JB2,.e
ps
Try typing  <return>  to proceed.
If that doesn't work, type  X <return>  to quit.

<Balternatus_studycase.pdf, id=207, 615.57536pt x 596.2275pt>
File: Balternatus_studycase.pdf Graphic file (type pdf)
<use Balternatus_studycase.pdf>
Package pdftex.def Info: Balternatus_studycase.pdf  used on input line
284.
(pdftex.def)             Requested size: 488.22787pt x 472.88869pt.

Package natbib Warning: Citation `dowell2016deep' on page 7 undefined on
input
line 290.


Package natbib Warning: Citation `almeida2021jararaca' on page 7
undefined on i
nput line 290.


Package natbib Warning: Citation `margres2021tigris' on page 7 undefined
on inp
ut line 290.


Package natbib Warning: Citation `queiroz2008venomtoxicity' on page 7
undefined
 on input line 290.


Package natbib Warning: Citation `dowell2016deep' on page 7 undefined on
input
line 290.


Package natbib Warning: Citation `koludarov2020familysaga' on page 7
undefined
on input line 290.


Package natbib Warning: Citation `schield2019Cviridis' on page 7
undefined on i
nput line 292.
```

Package natbib Warning: Citation `giorgianni2020svmp' on page 7 undefined on in
put line 292.

Package natbib Warning: Citation `margres2021tigris' on page 7 undefined on inp
ut line 292.

Package natbib Warning: Citation `almeida2021jararaca' on page 7 undefined on i
nput line 292.

Package natbib Warning: Citation `nachtigall2021toxcodan' on page 7 undefined o
n input line 292.

Package natbib Warning: Citation `almeida2021jararaca' on page 7 undefined on i
nput line 292.

Package natbib Warning: Citation `giorgianni2020svmp' on page 7 undefined on in
put line 292.

Package natbib Warning: Citation `almeida2021jararaca' on page 7 undefined on i
nput line 294.

Package natbib Warning: Citation `nachtigall2021toxcodan' on page 7 undefined o
n input line 294.

Package natbib Warning: Citation `almeida2021jararaca' on page 7 undefined on i
nput line 294.

[7]

Package natbib Warning: Citation `gopalan2022crotamine' on page 8 undefined on
input line 296.

Package natbib Warning: Citation `almeida2021jararaca' on page 8 undefined on i
nput line 298.

```
Package natbib Warning: Citation `schield2019Cviridis' on page 8
undefined on i
nput line 298.


Package natbib Warning: Citation `margres2021tigris' on page 8 undefined
on inp
ut line 298.


Package natbib Warning: Citation `vonReumont2022venomics' on page 8
undefined o
n input line 310.


Package natbib Warning: Citation `schield2019Cviridis' on page 8
undefined on i
nput line 314.


Package natbib Warning: Citation `giorgianni2020svmp' on page 8 undefined
on in
put line 314.


Package natbib Warning: Citation `almeida2021jararaca' on page 8
undefined on i
nput line 314.


Package natbib Warning: Citation `margres2021tigris' on page 8 undefined
on inp
ut line 314.


Package natbib Warning: Citation `cardoso2010alternatus' on page 8
undefined on
 input line 314.


Package natbib Warning: Citation `dePaula2014alternatus' on page 8
undefined on
 input line 314.


Package natbib Warning: Citation `nachtigall2021toxcodan' on page 8
undefined o
n input line 314.


Package natbib Warning: Citation `giorgianni2020svmp' on page 8 undefined
on in
```

```
put line 314.


Package natbib Warning: Citation `perry2022snake' on page 8 undefined on
input
line 314.


Package natbib Warning: Citation `giorgianni2020svmp' on page 8 undefined
on in
put line 314.

[8]

Package natbib Warning: Citation `cao2013mesobuthus' on page 9 undefined
on inp
ut line 316.


Package natbib Warning: Citation `vonk2013king' on page 9 undefined on
input li
ne 316.


Package natbib Warning: Citation `yin2016evolutionary' on page 9
undefined on i
nput line 316.


Package natbib Warning: Citation `drukewitz2019dipteraVenom' on page 9
undefine
d on input line 316.


Package natbib Warning: Citation `schield2019Cviridis' on page 9
undefined on i
nput line 316.


Package natbib Warning: Citation `almeida2021jararaca' on page 9
undefined on i
nput line 316.


Package natbib Warning: Citation `margres2021tigris' on page 9 undefined
on inp
ut line 316.


Package natbib Warning: Citation `zhang2022bungarus' on page 9 undefined
on inp
ut line 316.
```

Package natbib Warning: Citation `ye2022waspsgenome' on page 9 undefined
on inp
ut line 316.

Package natbib Warning: Citation `whalen2022ml' on page 9 undefined on
input li
ne 316.

Package natbib Warning: Citation `VenomZone' on page 9 undefined on input
line
318.

Package natbib Warning: Citation `jungo2012toxprot' on page 9 undefined
on inpu
t line 318.

Package natbib Warning: Citation `kaas2012conoserver' on page 9 undefined
on in
put line 318.

Package natbib Warning: Citation `pineda2018arachnoserver' on page 9
undefined
on input line 318.

Package natbib Warning: Citation `li2021seaSnakes' on page 9 undefined on
input
line 320.

Package natbib Warning: Citation `hogan2021chemosensory' on page 9
undefined on
input line 320.

Package natbib Warning: Citation `macias2019opsin' on page 9 undefined on
input
line 320.

Package natbib Warning: Citation `hughes2018birth' on page 9 undefined on
input
line 320.

Package natbib Warning: Citation `miller2015mhc' on page 9 undefined on
input l
ine 320.

```
Package natbib Warning: Citation `ukken2022fetuin' on page 9 undefined on
input
 line 320.


Package natbib Warning: Citation `gaunt2022hox' on page 9 undefined on
input li
ne 320.


Package natbib Warning: Citation `frankish2019gencode' on page 9
undefined on i
nput line 320.


Package natbib Warning: Citation `peel2022best' on page 9 undefined on
input li
ne 320.

LaTeX Font Info:    Trying to load font information for T1+lmtt on input
line 3
32.
(c:/TeXLive/2022/texmf-dist/tex/latex/lm/t1lmtt.fd
File: t1lmtt.fd 2015/05/01 v1.6.1 Font defs for Latin Modern
)
Package microtype Info: Loading generic protrusion settings for font
family
(microtype)             `lmtt' (encoding: T1).
(microtype)             For optimal results, create family-specific
settings.
(microtype)             See the microtype manual for details.

Underfull \hbox (badness 4291) in paragraph at lines 332--333
[]\T1/Merriwthr-OsF/m/up/7.5 (+20) Project home page:
[][]$\T1/lmtt/m/n/7.5 htt
ps : / / github . com / pedronachtigall /
 []


Package natbib Warning: Citation `sayers2023ncbi' on page 9 undefined on
input
line 345.


Package natbib Warning: Citation `Balt_assembly' on page 9 undefined on
input l
ine 345.


Package natbib Warning: Citation `Balt_toxin' on page 9 undefined on
input line
 345.
```

```
Package natbib Warning: Citation `Balt_nontoxin' on page 9 undefined on
input l
ine 345.


Package natbib Warning: Citation `Balt_BUSCO' on page 9 undefined on
input line
 345.


Package natbib Warning: Citation `ToxCodanGenomeRepo' on page 9 undefined
on in
put line 345.


Package natbib Warning: Citation `ToxCodanGenomeGuide' on page 9
undefined on i
nput line 345.


Package natbib Warning: Citation `ToxCodanGenomeGigadb' on page 9
undefined on
input line 345.


Underfull \vbox (badness 1881) has occurred while \output is active []

[9]
No file main.bbl.

Package natbib Warning: There were undefined citations.

[10

] [11 <./Figure2_tgtests.pdf>] [12 <./Figure_3_tgtests.pdf>] [13
<./Fig_proof_c
oncept.pdf>] [14 <./Balternatus_studycase.pdf>]
enddocument/afterlastpage: lastpage setting LastPage.
(./main.aux)

LaTeX Font Warning: Size substitutions with differences
(Font)              up to 1.0pt have occurred.


LaTeX Font Warning: Some font shapes were not available, defaults
substituted.

Package rerunfilecheck Info: File `main.out' has not changed.
(rerunfilecheck)             Checksum:
3154FBE866DFFEB946020B6C61BC9B90;4488.
 )
Here is how much of TeX's memory you used:
 24080 strings out of 476024
```

```
 471499 string characters out of 5794017
 1909382 words of memory out of 5000000
 43349 multiletter control sequences out of 15000+600000
 1852885 words of font info for 619 fonts, out of 8000000 for 9000
 1141 hyphenation exceptions out of 8191
 123i,13n,131p,2946b,1033s stack positions out of
10000i,1000n,20000p,200000b,200000s
{c:/TeXLive/2022/texmf-dist/fonts/enc/dvips/lm/lm-
ec.enc}{c:/TeXLive/2022/tex
mf-
dist/fonts/enc/dvips/merriweather/merriwthr_posqbl.enc}{c:/TeXLive/2022/t
exm
f-
dist/fonts/enc/dvips/merriweather/merriwthr_owzwzj.enc}<c:/TeXLive/2022/t
exmf
-dist/fonts/type1/sorkin/merriweather/Merriwthr-
Bold.pfb><c:/TeXLive/2022/texmf
-dist/fonts/type1/sorkin/merriweather/Merriwthr-
BoldItalic.pfb><c:/TeXLive/2022
/texmf-dist/fonts/type1/sorkin/merriweather/Merriwthr-
Italic.pfb><c:/TeXLive/20
22/texmf-dist/fonts/type1/sorkin/merriweather/Merriwthr-
Regular.pfb><c:/TeXLive
/2022/texmf-
dist/fonts/type1/public/amsfonts/cm/cmsy6.pfb><c:/TeXLive/2022/texm
f-dist/fonts/type1/public/lm/lmtt8.pfb>
Output written on main.pdf (14 pages, 2343630 bytes).
PDF statistics:
 412 PDF objects out of 1000 (max. 8388607)
 331 compressed objects within 4 object streams
 50 named destinations out of 1000 (max. 500000)
 211708 words of extra memory for PDF output out of 221844 (max.
10000000)
```

Manuscript
Click here to access/download;Manuscript;ToxCodAnGenome_GigaScience

OXFORD (GIGA)^n SCIENCE

GigaScience, 2017, 1–17

doi: xx.xxxx/xxxx
Manuscript in Preparation
Paper

PAPER

ToxCodAn-Genome: an automated pipeline for toxin-gene annotation in genome assembly of venomous lineages

Pedro G. Nachtigall[1,2,*,†], Alan M. Durham[3,‡], Darin R. Rokyta[2,§] and Inácio L.M. Junqueira-de-Azevedo[1,¶]

[1]Laboratório de Toxinologia Aplicada, CeTICS, Instituto Butantan, Av. Vital Brasil, 1500, São Paulo, 05503-900, SP,Brazil and [2]Department of Biological Science, Florida State University, 319 Stadium Drive, Tallahassee, 32306-4295, FL, USA and [3]Departamento de Ciência da Computação, Instituto de Matemática e Estatística, Universidade de São Paulo (USP), R. do Matão, 1010, São Paulo, 05508-090, SP, Brazil

[*]pedronachtigall@gmail.com

Abstract

Background: The rapid development of sequencing technologies resulted in a wide expansion of genomics studies using venomous lineages. This facilitated research focusing on understanding the evolution of adaptive traits and the search for novel compounds that can be applied in agriculture and medicine. However, the toxin annotation of genomes is a laborious and time-consuming task, and no consensus pipeline is currently available. No computational tool currently exists to address the challenges specific to toxin annotation and to ensure the reproducibility of the process.
Results: Here, we present ToxCodAn-Genome, the first software designed to perform automated toxin annotation in genomes of venomous lineages. This pipeline was designed to retrieve the full-length coding sequences of toxins and to allow the detection of novel truncated paralogs and pseudogenes. We tested ToxCodAn-Genome using 12 genomes of venomous lineages and achieved high performance on recovering their current toxin annotations. This tool can be easily customized to allow improvements in the final toxin annotation set and can be expanded to virtually any venomous lineage. ToxCodAn-Genome is fast, allowing it to run on any personal computer, but it can also be executed in multi-core mode, taking advantage of large high-performance servers. In addition, we provide a guide to direct future research in the venomics field to ensure a confident toxin annotation in the genome being studied. As a case study, we sequenced and annotated the toxin repertoire of *Bothrops alternatus*, which may facilitate future evolutionary and biomedical studies using vipers as models.
Conclusions: ToxCodAn-Genome is suitable to perform toxin annotation in the genome of venomous species and may help to improve the reproducibility of further studies. ToxCodAn-Genome and the guide are freely available at https://github.com/pedronachtigall/ToxCodAn-Genome.

Key words: WGS, DNA-seq, genome annotation, gene model, venomics

Introduction

Over the last two decades, the rapid development of sequencing technologies, which includes wet- and dry-bench protocols, has decreased the cost and time to generate high-quality genome assemblies (reviewed in [1]). This resulted in a wide expansion of the number of species in the Tree of Life with a sequenced genome [2]. In particular, the genome sequencing of venomous lineages has

Compiled on: December 11, 2023.
Draft manuscript prepared by the author.

Key Points

- We present ToxCodAn-Genome, the first automated computational pipeline designed specifically for toxin-gene annotation in genome assemblies of venomous species.
- The analysis using 12 available genomes from snakes, stingrays, scorpions, Hymenoptera, and Anthozoa showed that ToxCodAn-Genome is suitable for use on any venomous species.
- The proof-of-concept test showed that ToxCodAn-Genome can annotate most of the toxins in the genome, which integrates the set of highly expressed toxins in the venom-tissue transcriptome.
- ToxCodAn-Genome is fast, accurate, and can be used on any personal computer or taking advantage of supercomputers.
- Our case study based on sequencing the genome of *Bothrops alternatus* revealed that ToxCodAn-Genome and our guide can be applied to understand the genomic context and evolution of venom genes.
- The draft genome of *Bothrops alternatus* allowed the recovery of the first complete SVMP loci in lancehead vipers.

become an useful approach to search for novel toxin compounds, which may help in the development of new medicines (reviewed in [3]), ensure the production of effective antivenoms [4], elucidate the genetic regulatory mechanisms related to complex phenotypes [5, 6, 7], and understand the evolutionary history of adaptive traits [8].

Venoms, along with their production and injection apparatus, have evolved independently more than 100 times in diverse lineages throughout the Tree of Life (reviewed in [9]). They are composed of a complex cocktail of proteins and peptides (also known as toxins) and are mainly used for prey capture and defense against predators, but may also be used in intraspecific competition [10, 11, 8]. The toxin composition of venom is a polygenic trait, frequently evolving under strong selection, and represents a key adaptive innovation [11]. Moreover, venoms and their toxins are excellent model systems to trace the impact of gene sequence mutations over protein function, as the majority of proteinaceous toxins are adapted to specific functions when injected into their targets [12, 10]. The biological effects of toxins and their remarkable target specificity are of high interest to the research community due to their potential in the fields of pharmacology, medicine, biotechnology, and agrochemistry [3, 4, 13, 14]. Sequencing the genomes of venomous lineages and deciphering their toxin repertoire within the genomic context, therefore, represents an outstanding opportunity across diverse research fields.

Despite the relevance of genomic studies to venomics, only a small percentage of venomous species have had their genomes sequenced and used to understand the genomic context of their toxin repertoire (reviewed in [9]). Of these, snakes represent the venomous clade with the most representatives studied, which has revealed some remarkable features in the evolution and novelty of venom systems [15, 16, 17, 18, 19, 20, 21]. However, many other snakes and venomous species are being studied, and their genomes are being widely sequenced to generate a high-quality assembly. In this context, a tool for performing fast and accurate toxin annotation will help to improve our knowledge of the biological roles and track the evolutionary history of venom and its toxic compounds.

Genome annotation is an important step for many biological studies, because it helps to decipher the biological pathways that lead to specific phenotypes [22]. Characterizing genes in bacterial genomes is relatively easy, because most of their genes do not present exon-intron structures and have short intergenic regions [23]. On the other hand, characterizing genes in eukaryotes is far more complex, because the genes are sparse in the genome (*i.e.*, there are long intergenic regions) and the genes are structured into an exon-intron context. Thus, the precise identification of exon-intron boundaries and exact localization of genes is not easily determined. These features make the annotation of eukaryotic genomes error-prone by nature and require development of suitable tools to help mitigate erroneous annotations [24].

Currently, several tools exist to perform gene annotation in the genomes of eukaryotic species (reviewed in [25]). These tools comprise distinct strategies that may range from *ab initio* prediction using pre-trained models to self-training algorithms to similarity search. The *ab initio* prediction tools, such as AUGUSTUS [26] and SNAP [27], search for genes based on a generic gene model, but they may also integrate protein and transcript sequences as evidence to validate the predicted genes. Some tools, like BRAKER [28, 29], MAKER [30], GeneMark–ES [31], and AUGUSTUS, can perform self-training of gene models specific to the genome being analyzed by using the outputs of preliminary runs to improve the performance of gene prediction on subsequent runs. These approaches may also integrate alignments of proteins and transcripts to use as evidence in the gene-prediction process. Other tools rely on using pre-trained species-specific models that can be integrated with protein evidence of closely related species, such as FGENESH+ [32]. These tools have been used in several published genomes, but they are dependent on powerful computing resources. This feature may result in a slow running time that may take up to a few weeks when insufficient computing resources are available. Other applications, such as GEMOMA [33], Liftoff [34], and TOGA [35], rely on the use of similarity searches using a high-quality and well-annotated genome of a closely-related species as a reference. These tools consider genome alignment and the homology and orthology inference of genes to build gene models and/or transfer annotation to the target species. However, in non-model organisms and less studied groups, a well-annotated genome from a closely-related species is commonly not available. Moreover, if the genome used as reference is not well-annotated and contains erroneous and incomplete annotations, these may be propagated to the target genome [36]. Independently of the strategy adopted, it is known that automated genome annotation tools do not accurately characterize complex gene families [37, 38], which requires laborious manual curation for a reliable and comprehensive annotation of a genome [39]. Therefore, the genome annotation task is a puzzle not easily solved that can benefit from improvements for specific cases [24].

Despite the plethora of available tools to perform an automated annotation of genomes, none of them were designed to solve the issues specific to the toxin annotation task [40]. The annotation of toxin genes presents some tricky issues when compared to general gene annotation in eukaryotes that may lead to incorrect or incomplete identification of gene structure and wrong assignment of gene names. These errors may result from the following features not adopted by the general annotation tools: (1) toxin repertoire are highly diverse among venomous taxa, which difficulties to set a reliable “toxin” feature to assist in the toxin gene identification; (2) toxin genes may share high similarity with their ancestral gene [19], which make it difficult to distinguish toxin genes from related nontoxin genes; (3) toxin genes may have originated from *in locus* duplication of an ancestral nontoxin gene [41, 42, 19]; (4) the duplicated toxin genes are commonly arranged in tandem arrays and can be highly similar [43, 44, 19, 45]; (5) the genomic regions of these

highly duplicated toxin genes are marked by the presence of orphan exons and pseudogenes (*e.g.*, commonly observed in metalloproteinases, serine proteases, phospholipases, and three-finger toxins loci of snake genomes), which complicates the correct annotation of these regions [41, 42, 46, 47, 21, 45]; (6) the high mutation rate of these toxin loci may also result in truncated paralogs, which may present a complete gene structure with a premature stop codon [48, 45]; and (7) the toxin loci can present high levels of genomic rearrangements [41, 42, 45]. All these features together introduce extra layers of complexity when annotating toxin genes in genomes. In fact, the genomes of venomous lineages published so far revealed that general annotation tools do not perform well on correctly characterizing toxin genes in genomes, which must be further checked using several distinct strategies and approaches that are not easily reproducible, require strong programming skills, and are laborious and time consuming [43, 15, 16, 44, 17, 19, 48, 21, 49, 45]. In this sense, the development of a tool able to quickly characterize the toxin repertoire in the genome of venomous lineages will help to minimize efforts in checking toxin annotations, mitigate the effects of erroneous annotations, and improve the reproducibility of analyses.

Here, we present ToxCodAn-Genome, an automated computational pipeline to annotate toxin loci in genomes of virtually any venomous lineage. Using genomic data from snakes, stingrays, scorpions, Hymenoptera, and Anthozoa species, we show that ToxCodAn-Genome has high performance and can be used to annotate toxin genes in different lineages. In fact, it can be easily configured to use on any venomous lineage by designing specific toxin databases and/or using venom-tissue transcriptomic data. To facilitate the use of ToxCodAn-Genome and help in future venomics research, we also provide an extended guide to perform toxin annotation of genomes. Finally, we sequenced and assembled the genome of *Bothrops alternatus* and annotated its toxin gene repertoire as a case study.

Materials and methods

Software implementation

ToxCodAn-Genome was developed using Python (v3.6) and third-party tools to perform the automated analysis (Figure 1). The pipeline consists of a step to detect putative toxin loci in the genome using a comprehensive toxin database, followed by a step to select bona fide toxin loci that are used to build gene models specific to each toxin loci and generate the toxin annotation file. Specifically, the "detection of putative toxin loci" step performs a similarity search using BLAST (v2.9 or higher) against toxin coding sequences (CDS) present in the toxin database (toxinDB; see "Toxin Databases" section). Then, all putative toxin loci are analyzed in the "selection of bona fide toxin loci" step, which consists of keeping matching regions containing only full-length toxin CDSs for the next step (*i.e.*, matching regions with partial toxin CDSs are not considered for building gene models). The "build gene models for each toxin loci" step uses the putative toxin loci containing full-length toxin CDSs to build gene models using Exonerate (v2.4.0 or higher; [50]), which performs refinement of the intron/exon boundaries, and generates the annotation file in GTF format containing the CDSs of the identified toxin loci.

ToxCodAn-Genome can also use a user-designed toxin database to complement any of the provided toxin databases. The custom toxin database can help improve annotations with the inclusion of more specific data from public or private databases, published manuscripts, and/or the user's own unpublished data. In particular, it can be built using venom-tissue transcriptomic data specific to the lineage/species being studied. The venom tissue transcriptome can be analyzed using tools designed specifically for this task, such as ToxCodAn [51] and/or Venomix [52]. However, to help users analyze the transcriptomic data, we implemented two scripts to assemble venom tissue transcripts and identify their toxin CDSs (Figure S1 in Supplementary file 1). The script to assemble transcripts (named "TRassembly.py") performs four assemblies considering genome-guided and *de novo* strategies to ensure the recovery of most toxin transcripts [53]. The genome-guided strategy uses Hisat2 (v2.2.1; [54]) to map reads against the genome and use the mapped read information to recover transcripts using StringTie (v2.2.1; [55]) and the genome-guided mode of Trinity (v2.8.5; [56]). The *de novo* strategy performs two *de novo* transcriptome assemblies using the *de novo* mode of Trinity and rnaSPAdes (v3.15.5; [57]). Then, all four assemblies are concatenated to generate the final set of transcripts to be used in the toxin screening step. The script designed to identify toxin CDSs in the assembled transcripts (named "CDSscreening.py") performs a BLAST search against a ToxinDB and identifies the full-length toxin CDSs. Both additional scripts can be run independently by the user or set to run directly within the main ToxCodAn-Genome pipeline.

By default, ToxCodAn-Genome outputs the toxin annotation file in GTF format, the CDS and peptide sequences in a FASTA format, and a file with "warning" annotations in TXT format, which contains information about annotations that may represent truncated isoforms, pseudogenes, or novelties that need further inspection. It also generates an annotation file containing all genomic regions matching full-length toxin CDSs in GTF format to be further inspected as needed by the user.

Guide to annotate toxins in genomes

To complement ToxCodAn-Genome, we produced a detailed guide for toxin annotation. Specifically, we provide the command-line code and links to useful resources to learn basic bioinformatics, to build a toxin database from public resources and/or using a venom-tissue transcriptomic data, to perform toxin and nontoxin annotation, to perform inspection of specific annotations when needed, and to perform quantification of annotated genes using transcriptomic data. We also provide an R script containing useful functions for plotting the toxin loci annotated through our pipeline. Our guide is available in Markdown format on our ToxCodAn-Genome GitHub repository [58] and in an archived PDF format at Supplementary file 2.

Toxin Databases

We built ToxinDB using sequences from species of the widely studied venomous clades of Viperidae, Elapidae, Myliobatoidea, Scorpiones, Hymenoptera, and Anthozoa. To build the ToxinDB, we retrieved full-length toxin CDSs from the nucleotide archive and the TSA databases of NCBI [59]. The full-length toxin CDSs of each lineage were clusterized with 99% similarity using cd-hit (v4.8.1; [60]) to reduce redundancy and generate a final toxin database for each lineage. The Viperidae database was composed of 1546 toxin CDSs from 108 species that clustered into a total of 1278 toxin CDSs. The Elapidae database was composed of 1592 toxin CDSs from 76 species that clustered into a total of 1150 toxin CDSs. The Myliobatoidei database was composed of 254 toxin CDSs from 7 species that clustered into a total of 192 toxin CDSs. The Scorpiones database was composed of 1879 toxin CDSs from 39 species that clustered into a total of 1122 toxin CDSs. The Hymenoptera database was composed of 432 toxin CDSs from 52 species that clustered into a total of 397 toxin CDSs. The Anthozoa database was composed of 1506 toxin CDSs from 29 species that clustered into a total of 980 toxin CDSs. Of note, the classification of toxins and toxin-like components can vary from one study to another, and from one lineage to another. Despite some rational nomenclature have been proposed for spiders to be extended to other lineages [61], the current studies seem to not follow such standards. In this sense, we used the VenomZone

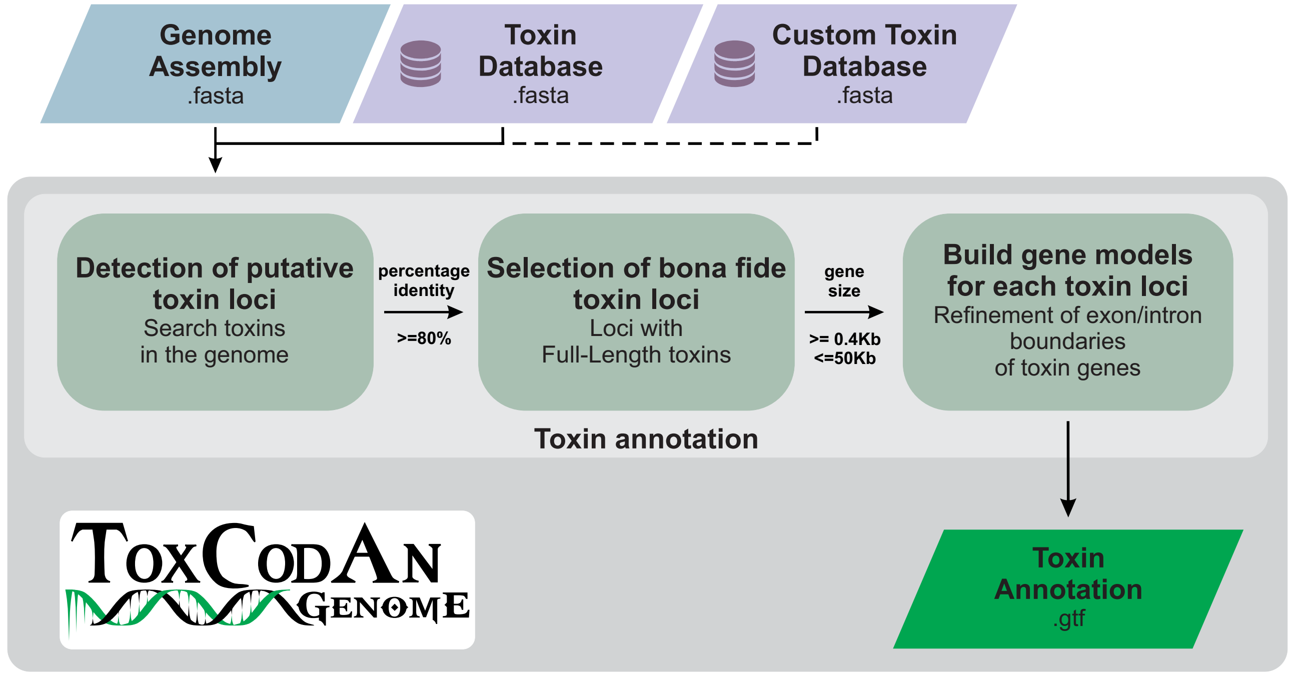

Figure 1. Flowchart of the ToxCodAn-Genome pipeline. The genome assembly is searched to detect putative toxin loci using a toxin database containing full-length toxin CDSs from several species. Putative toxin loci are analyzed to select bona fide toxin loci that are used to build gene models specific to that toxin loci and output the final toxin annotation.

resource [62] and published manuscripts to track down the toxin genes within each lineage studied in the present study. We strongly recommend the users of ToxCodAn-Genome to review the literature about venom components and databases such as VenomZone, ToxProt [63], as well as other resources [64, 65], to make a better choice of toxin annotation classes for the target lineage.

Testing sets

To test the performance of ToxCodAn-Genome, we downloaded genomes of three Viperidae, three Elapidae, one Myliobatoidea, one Scorpiones, and three Hymenoptera species previously published along with the descriptions of their toxin gene repertoire (Table 1; Table S1 in Supplementary file 3). The number of toxin genes in each species was considered as reported by the original publication, except for *Potamotrygon leopoldi*, where no toxin annotations were reported and the number of toxin genes was considered based on its venom tissue transcriptome report [66], and for *Nematostella vectensis*, where the toxin annotations were considered as annotated in a recent venomics study of the Anthozoa lineage (Smith et al., 2023). Then, we compared the total number of toxins identified by ToxCodAn-Genome to the number reported in the previously published annotations. The ToxCodAn-Genome's annotations were characterized as "reliable" or "warning" based on the output files (see Figure S2 in Supplementary file 1 for more details). Briefly, the "reliable" annotations represent bona fide toxin annotations with a well defined exon-intron structure and a full-length toxin CDS, whereas the "warning" annotations represents an annotation corresponding to a full-length toxin CDS, with a well defined exon-intron structure but with a premature stop codon. The "warning" annotations may represent truncated paralogs, pseudogenes, an erroneous annotation, or may be a result of errors in the genomic region assembly.

To assess the quality of ToxCodAn-Genome's annotation, we computed the toxin recovery rate (TRR) for each major toxin family in the target testing set. The TRR is computed by dividing the number of ToxCodAn-Genome's annotations by the number of toxin loci reported in the original genome annotation for each toxin family. A TRR value equal to 1 indicates an exact match between the number of toxin loci annotated and the number described in the original report. A TRR higher than 1 indicates that ToxCodAn-Genome detected more toxin loci than that originally reported. A TRR lower than 1 indicates that ToxCodAn-Genome detected fewer toxin loci than that originally reported.

To check the effects of database and transcriptomic data on ToxCodAn-Genome's performance, we set three distinct scenarios: (1) using only the toxin database (DB), (2) integrating toxin database and venom tissue transcriptomic data (DBTR); and (3) using only the toxin-annotated venom tissue transcriptome (TR). Of note, to ensure a fair performance analysis, we removed the species-specific toxin CDSs of the target species from the toxinDB to perform the tests and annotate the venom tissue transcriptomes.

Proof-of-concept test

To evaluate the capability of ToxCodAn-Genome to perform a reliable toxin annotation in a more controlled way, we performed a proof-of-concept test by using the three Viperidae species from the initial testing set (Table 1). In this test, we specified their available and published toxin CDSs as the only database source to annotate their genomes and ran ToxCodAn-Genome with default parameters. We compared the published annotations and ToxCodAn-Genome's output available in two files: "toxin_annotation.gtf" and "matched_regions.gtf". The "toxin_annotation.gtf" contains the final gene model for each toxin and reveals if ToxCodAn-Genome was able to correctly annotate or not that specific toxin (*i.e.*, toxins were labeled as "annotated" or "not annotated"). As an independent measure from the correct annotation, we also analyzed the "matched_regions.gtf" to check when ToxCodAn-Genome considered a toxin as a putative toxin locus (*i.e.*, when identified their full-length toxin match in the genome). Here, we labeled each toxin as "matched and annotated" (*i.e.*, when the toxin was also correctly annotated in the "toxin_annotation.gtf" file), "matched but not annotated" (*i.e.*, when the toxin was not annotated in the "toxin_annotation.gtf" file but has a match in their correct genomic position), or "not matched" (*i.e.*, when the toxin was not detected

Table 1. Genomic and transcriptomic data used to test ToxCodAn-Genome.

Lineage	Species	Genome	Venom tissue RNA-seq (SRA)	References
Viperidae	*Azemiops feae*	GCA_023970755.1	SRR18397788	[20]
	Bothrops jararaca	GCA_018340635.1	SRR13839799	[19]
	Crotalus tigris	GCA_016545835.1	SRR11545022	[48]
Elapidae	*Hydrophis curtus*	PRJNA597425	SRR11659669	[18]
	Hydrophis cyanocinctus	JAAZTL000000000	SRR11659657	[67]
	Naja naja	GCA_009733165.1	SRR8754977	[68]
Myliobatoidei	*Potamotrygon leopoldi*	GWH:GWHAOTN00000000	SRR11049204	[69]
Scorpiones	*Mesobuthus martensii*	GCA_000484575.1	SRR2592960	[43]
Hymenoptera	*Apis cerana*	GCF_001442555.1	SRR1406762	[70]
	Apis mellifera	GCF_003254395.2	SRR13213116	[71]
	Nasonia vitripennis	GCF_009193385.2	SRR3046453	[72]
Anthozoa	*Nematostella vectensis*	GCF_932526225.1	SRR11600272	[73]

as a putative toxin locus). Additionally, we estimated the expression level of toxins using their venom tissue transcriptomic data, their toxin CDSs, and RSEM (setting the mismatch rate parameter to 2%; v1.3.1; [74]) to measure when ToxCodAn-Genome was able to correctly annotate highly and/or lowly expressed toxin genes.

Running time analysis

To assess the running time of ToxCodAn-Genome, we used the *Crotalus tigris* genome (genome size of 1.6Gb) with the Viperidae toxinDB (containing 1278 toxin CDSs) and its assembled venom gland transcriptome (total of 257734 transcripts). We performed the analyses using a personal computer (Intel 6-Core i7 with 16-Gb memory) and set the number of threads to 6 ("-c 6"). Of note, we only considered the running times to generate the custom toxin database using the venom tissue transcriptome assembly (*i.e*, running time of "CDSscreening.py") and to perform the genome annotation (*i.e*, running time of ToxCodAn-Genome), because the running times of assembling transcripts is solely related to the processing times needed by each third-party tools used in the "transcriptome assembly" module (*i.e*, Hisat2, StringTie, Trinity, and rnaSPAdes).

Bothrops alternatus case study

As a case study, we sequenced, assembled, and annotated the toxin repertoire of the genome of the urutu lancehead (Viperidae: *Bothrops alternatus*). The urutu lancehead is a large pit viper, with an average size of 754.5 mm, and it is considered to be a dietary specialist, feeding almost exclusively on mammals [75]. Its geographical distribution ranges from Northern Argentina to the South/Central Brazil, Paraguay and Uruguay [76]. Although the venom of *B. alternatus* has been broadly investigated through transcriptomics [77, 78, 51], its genomic background has yet to be determined.

Here, we briefly describe each step of data analysis but a detailed description can be found in our guide ([58]; Supplementary file 2).

Blood sampling and DNA extraction

One specimen (SB0060) was collected in September 2017 in Mato Grosso do Sul state, Brazil. Blood was extracted from the caudal vein, transferred to a tube containing 100% ethanol solution, and stored at -80 °C until use. High-molecular-weight (HMW) genomic DNA (gDNA) was extracted by using a pipette-free protocol as previously described [48]. The snake was handled and collected under Protocol Number 4479020217 from the Ethic Committee on Animal Use of the Butantan Institute (CEUAIB).

The transcriptomic data of venom gland from the same individual used for genome sequencing was obtained as previously described [51], and it is available at the SRA database in the NCBI (access number SRR13153633).

Genome sequencing and assembling

HMW DNA was used to construct the PacBio HiFi sequencing libraries with the SMRTbell Express Template Prep Kit 2.0 following the manufacturer's protocol. Sequencing was performed with two cells on the PacBio Sequel II system in CCS mode at the University of Delaware Sequencing and Genotyping Center. Two cells were sequenced and resulted in 3,446,639 reads (total of 48,922,414,841 bp; >28-fold coverage; and an average read size of 14.2 Kb).

The PacBio HiFi reads were assembled using hifiasm (v0.16.1; [79]) and polished using Inspector (v1.0.1; [80]). The genome assembly statistics were obtained within Inspector and the completeness was assessed by using BUSCO (v5.2.2; [81]) with the tetrapoda gene set (odb10; total of 5310 genes). We annotated repetitive elements using the EDTA pipeline (v2.0.0; [82]).

Genome annotation

For toxin annotation, we used ToxCodAn-Genome with default parameters, the venom gland transcriptome assembly and the Viperidae database. We then inspected the annotated toxins as follows: (1) compared the toxin CDSs to the toxins annotated in previous transcriptomic studies of *B. alternatus* [51] and other *Bothrops* species (*i.e.*, *B. cotiara*, *B. fonsecai*, *B. jararaca*, and *B. jararacussu*; [19, 83, 84]), to ensure it represents confident toxin annotations; (2) checked the annotated toxins present in the output "annotation_warning.txt", which may represent truncated paralogs, pseudogenes, or erroneous annotations, to confirm its gene model, we re-annotated it using the free version of FGENESH+ [32] with the protein sequence of toxin matched in that region, as stated in the output "matched_regions.gtf", and the gene model designed for *Anolis carolinensis*, which is the closely related species with a trained model in the FGENESH+ server; and (3) checked the regions matching to full-length toxin CDSs (*i.e.*, available in the output "matched_regions.gtf") with no toxin annotated in the final annotation output (*i.e.*, "toxin_annotation.gtf") using FGENESH+ with the protein sequence of the matched toxin to ensure the region does not contain any toxin gene and may represent an intergenic region.

To annotate nontoxin genes, we used the funannotate pipeline [85]. This annotation pipeline consists of the integration of several *ab initio* gene predictors to build gene models (*i.e.*, AUGUSTUS, SNAP, GeneMark–ES, and GlimmerHMM) and uses transcript and protein evidence to generate the final annotation set. Then, we set to use the venom gland transcriptomic data of the species as transcript evidence and the protein sequences available for the Tetrapoda clade in Uniprot and NCBI databases as protein evidence. We also performed the functional annotation step using InterProScan5 (v5.54; [86]).

We also performed a phylogenetic inference for the CTL genes to better characterize them as alpha and beta chains. We retrieved available venom CTL sequences from other snakes, aligned their peptide sequences using MAFFT (v7.450; [87]) and used IQTree (v1.6.12; [88]) to search for the maximum likelihood tree. The final tree was adjusted using FigTree (v1.4.4; [89]).

Results

Toxin annotation performance

Overall, the analysis using the genomes of 12 venomous species, including snakes (Figure 2), stingrays, scorpions, hymenopterans, and anthozoans (Figure 3), revealed that ToxCodAn-Genome can annotate most of their toxin gene repertoires in all tested scenarios (*i.e.*, using the toxin database only and/or integrating a venom-tissue transcriptomic data). In fact, ToxCodAn-Genome was able to match or surpass the number of toxin annotations in 7 of the 11 testing sets. This is particularly relevant when we consider that ToxCodAn-Genome can be executed in a single step, while the original genome annotations applied distinct protocols not easily reproducible. When using only the database or only the transcriptome, we noticed a lower number of annotated toxins when compared to the expected annotations in all testing sets. The integration of a database and venom-tissue transcriptomic data obtained the best performance for recovering the toxin gene repertoire in all testing sets. These results indicate that RNA-seq experiments alone are not able to identify low-expression toxin loci and that the combined use of homologous and orthologous sequences of closely related species (*i.e.*, a toxinDB) improves the final toxin annotations. Also, the use of a toxinDB containing homologous toxin sequences allows the detection of pseudogenes and truncated paralogs, which may represent novel toxin compounds.

In the snake testing sets (Figure 2), ToxCodAn-Genome presented a high performance in all genomes analyzed. This result may derive from the fact that snakes represent the most studied venomous lineage [9], and the availability of diverse toxin CDSs from several species may improve the toxin annotation performance. A higher number of toxin CDSs in the database may increase the probability of identifying orthologous and homologous toxins for target species, which results in a better recovery of toxin loci when using only the database but also when integrating the transcriptome. Interestingly, ToxCodAn-Genome annotated extra loci for some toxins in the *B. jararaca* testing set (*i.e.*, toxins with TRR values of 2). This may represent bona fide duplication events not previously detected or may be a result of duplicated genomic regions in the genome assembly as observed in the BUSCO score of this genome assembly (Table S1 in Supplementary file 3).

In the stingrays testing set (Figure 3), the results revealed that most toxin genes were recovered when using the integration of database and transcriptome; however, most toxins were identified with more copies than expected (*i.e.*, toxins with TRR values of 2). It may be related to the fact that we considered the annotations obtained from a *de novo* transcriptome assembly report as the expected number of toxins [66], which does not account for lowly or not expressed toxins in the genome. Therefore, efforts to perform a deep characterization and confirm the toxin repertoire of the *Potamotrygon leopoldi* genome may reveal a better picture of ToxCodAn-Genome performance within this venomous clade. Nonetheless, the availability of a Myliobatoidea toxin database will certainly help further genomic studies to retrieve the complete toxin repertoire of freshwater and saltwater stingrays.

In the Scorpiones testing set (Figure 3), ToxCodAn-Genome returned a lower number of annotations than expected. This result may be related to the *Mesobuthus martensii* genome assembly quality, which only presents 53% of BUSCO score (Table S1 in Supplementary file 3). Their genome publication did not describe if all annotated toxins were full-length CDSs or if they may also be represented by partial CDSs in fragmented genomic regions [43]. The assembly quality is a feature shown to affect the annotation of complex genes [38], which may affect the toxin annotation performance of ToxCodAn-Genome as well. However, the TRR was consistent with the number of toxin annotations, which indicates that ToxCodAn-Genome may be able to recover most toxin loci in a high-quality genome assembly of scorpion species using the complete Scorpiones toxin database integrated with a species-specific venom-tissue transcriptome.

In the Hymenoptera testing set (Figure 3), the number of toxin loci annotated were below the expected annotations in two datasets (*i.e*, *Apis mellifera* and *Nasonia vitripennis*) and achieved a good match in *A. cerana*. However, the TRR value was close to 1 for most toxins in all testing sets. Interestingly, the *A. cerana* testing set returned the best performance in toxin annotation within the Hymenoptera clade. This may be related with the fact that most toxins studied and available for this clade in the toxin database are from *A. mellifera* (95 from 397) and *N. vitripennis* (71 from 397). In this sense, the lower performance obtained in the *A. mellifera* and *N. vitripennis* testing sets when compared to *A. cerana* may be related to the lower diversity of toxin sequences in the testing database when removing these target species. It indicates that the diversity of sequences in the database being used (*i.e.*, the abundance of homologous and orthologous sequences) may interfere in the final toxin annotation set.

In the Anthozoa testing set (Figure 3), the number of annotated toxins presented a good match to the number of expected annotations for most toxins. The TRR of the main toxins composing the venom of *Nemastotella vectensis* was close to 1 in all scenarios tested. Two toxin families were underrepresented in the final toxin annotation set (*i.e*, the TRR is lower than 1 for toxin families NEP3 and KTx2), but they represent lowly expressed components in the species [45]. The low performance to retrieve these two lowly expressed venom components of *N. vectensis* may be related to the high divergence observed in toxins among Anthozoa species [45]. However, ToxCodAn-Genome was able to fully annotate the most abundant toxin gene of the venom tissue of *N. vectensis* (*i.e*, the toxin NaTx), which also present the most number of copies in their genome (*i.e*, 18 genes of NaTx in a total of 33 toxin genes) and a higher diversity of paralogs among *N. vectensis* populations [45]. In this sense, our tests indicate that ToxCodAn-Genome has high performance to retrieve the most complex toxin families in the genomes of Anthozoa species.

In summary, our tests revealed that any strategy alone allows a confident toxin annotation. Nonetheless, the integration of a toxin database with species-specific venom-tissue transcriptomic data presented the best performance and allowed the recovery of most toxin loci. In this sense, ToxCodAn-Genome is suitable for the toxin gene annotation task and can be applied to virtually any venomous lineage with the availability of curated toxin sequences from closely related species and/or a species-specific venom-tissue transcriptomic data.

Proof-of-concept test of ToxCodAn-Genome

The proof-of-concept test revealed that ToxCodAn-Genome is able to correctly annotate most of the toxins (Figure 4). For the toxins not annotated by ToxCodAn-Genome, most of them were identified as matching into their specific genomic positions, which allows the user to easily retrieve their annotations by inspecting the "matched_regions.gtf" file when performing a manual curation. For the few toxins missing a confident match in the genome, we noticed that they were missing due to their partial annotation in the original publications or their gene sizes ranging out of the ToxCodAn-Genome default parameters (as discussed below in details for each testing set); however, it is a feature that can be modified to allow the annotation of such toxin genes with longer or shorter gene sizes. It is noticeable that most of the toxin genes annotated are among the highly expressed toxin genes, which shows the capability of ToxCodAn-Genome to correctly annotate major venom components in the genome.

In the *A. feae* testing set, ToxCodAn-Genome was able to confidently annotate 42 from a total of 51 toxin genes in the original publication (82.4% of precision in automatic annotation; Figure

4). From the toxins not automatically annotated, 5 were identified in their correct positions in the "matched_regions.gtf" file, which indicate them as putative toxins (92.1% of precision in supervised annotation). Among the four missing toxins, two were marked as "partial" (PLA2-1 and SVMP-6; see Supplementary Information of [20]) and two presented a gene size longer than the threshold considered by ToxCodAn-Genome (*i.e.*, ENPP2 has a gene size around 52 Kb and VenomFactor has a gene size around 380 Kb in *A. feae* genome, whereas the threshold considers only genes with size shorter than 50 Kb). In this sense, modifying the gene size parameter when running ToxCodAn-Genome may allow to correctly identify both missing toxin genes.

The *B. jararaca* testing set revealed that 44 from the 51 toxin genes annotated in the publication were correctly annotated, which represents around 86.3% of precision in automatic annotation (Figure 4). From the 7 toxins not properly annotated, 4 were correctly identified in the "matched_regions.gtf" file, which indicates putative toxins and they can be annotated with further inspections (94.1% of precision in supervised annotation). From the 3 toxins missing in the match file, we noticed that 2 were partial in the published annotation (*i.e.*, KAG5858131.1_SVMP and KAG5858131.1_SVMP; [19]). The other missing toxin gene, KAG5858164.1_LAO, is marked as non conclusive by the authors of their original publication (see Supplementary Information from [19]), which indicates that the gene may be splitted into two or more scaffolds. Although the authors were able to annotate parts of the gene and retrieve a full-length CDS for this toxin gene, their draft assembly and their approach to detect venom genes did not allow them to retrieve a complete annotation of this gene. In this sense, the missing toxin genes in the *B. jararaca* testing set can be related to incomplete genomic loci in the assembled genome.

Analyzing the *C. tigris* testing set showed that 42 toxin genes were correctly annotated from the total of 50 toxin genes, representing 84% of precision in automatic annotation (Figure 4). From the 8 toxins not annotated, five had their genomic positions detected in the "matched_regions.gtf" file, whereas 3 were missed (94.0% of precision in supervised annotation). The 3 missing annotations, all of them presented annotations outside the gene size and CDS size range. Specifically, PDE and VF have a gene size longer than the gene size threshold (*i.e.*, greater than 50 Kb), whereas MYO has a CDS shorter than the CDS size threshold (*i.e.*, MYO has a CDS size of 196 bp, whereas the minimum threshold is 200 bp). Adjusting such parameters may help to identify and annotate such toxin genes.

In summary, the proof-of-concept testing set allowed us to measure the capability of ToxCodAn-Genome to annotate most of the toxin genes, which included highly expressed toxin genes in the venom tissue transcriptome. Among the missing genes, the user can deeply inspect the "matched_regions.gtf" file and modify some parameters to retrieve a complete set of annotated toxins. In this sense, ToxCodAn-Genome presents a high precision and also generated hints that allows the annotation of a complete set of toxins.

Running time

We measured the processing times of ToxCodAn-Genome on annotating toxins in the *C. tigris* genome using a personal computer (Intel 6-Core i7 with 16-Gb memory). The test revealed that ToxCodAn-Genome can perform the toxin annotation task in 1m51s when using only the database, and 16m23s when also using the transcriptome assembly to complement the toxin survey, by using 6 threads (parameter "-c 6"). The running time of both strategies can be decreased by setting more CPUs to perform the annotation when available. It indicates that ToxCodAn-Genome is a fast tool that can be used on any personal computer with a UNIX operating system or can take advantage of supercomputers.

Bothrops alternatus case study

The assembled genome of the urutu lancehead snake was of a higher quality than the available genome of the closely related species *B. jararaca* [19]. The assembled genome of *B. alternatus* had a total size of 1.7Gb and is composed of 1555 contigs with a N50 value of 13.9Mb. BUSCO analysis revealed 95.8% of complete conserved tetrapoda orthologous genes, which indicates high contiguity and completeness (Figure S3 in Supplementary file 1 and Table S2 in Supplementary file 3). We obtained a sequencing depth of 28.12 and a QV score of 36.78, which indicates an accurate assembly with low error rate. The assembly revealed that 46.64% of the genome is composed of repetitive elements, which is in agreement with previous published genomes of vipers [17, 48, 20]. The funannotate pipeline allowed us to annotate 29,245 protein-coding genes, of which only 15 toxin genes were correctly annotated (Supplementary file 4). The toxin genes correctly annotated by funannotate were mainly composed of single copy genes, which represent minor components of the venom.

Using ToxCodAn-Genome, we annotated 59 toxin genes from 16 toxin families in the *B. alternatus* genome (Figure 5; Table S3 in Supplementary file 3). Similar to previously observed in *B. jararaca*, most toxin families are represented by a single locus (*i.e.*, BPP, VEGF-F, LAO, PLB, HYAL, NGF, CRISP, KUN, NUC, CYS, and Waprin), whereas the other toxin families were organized as tandem arrays (*i.e.*, SVMP, PLA2, and SVSP) and the CTLs were detected as pairs in several genomic regions. Among the expressed toxins, we noticed that PLA2s, SVMPs, SVSPs, and CTLs compose the major components of the venom gland transcriptome, which are also toxins with multiple copies in the genome (Figure 5).

The PLA2s were arranged in tandem array between other non-toxin PLA2 genes (*i.e.*, PLA2E, PLA2F, and PLA2F) and flanked by OTUD3 and MUL1 genes, which is a pattern broadly conserved across vipers and other non-venomous tetrapods [41, 19, 48]. The PLA2 gene presenting the highest expression level among all toxins is an acidic D49 type and may be responsible for the phospholipase activity observed in the venom of *B. alternatus* [90]. Although we highlighted the PL2C in the toxin set, it is lowly expressed and may have minor roles in the venom toxicity of the species, and it is hypothesized to be the precursor of PLA2s with high toxic functions in vipers [41, 46].

We were able to retrieve the complete SVMP array in *B. alternatus* genome, which is composed of a total of 25 SVMP genes and flanked by ADAM28 and NEFM genes. This genomic context observed in SVMPs is broadly conserved among other vipers [17, 42, 48, 19], but it represents the first report of this complete locus in a *Bothrops* species. Of the total 25 SVMP genes, 20 represented a PIII class and 5 represented a PII class, corroborating a previous venom gland transcriptomics report [51]. Moreover, we did not identify any PI class, which is consistent with the *B. jararaca* genome [19]. Interestingly, the SVMP gene with lowest expression level is a PIII neighboring the ADAM28 gene (SVMP-1), similar to that observed in other vipers [42]. On the other hand, the SVMPs with the highest expression level were detected in the central region of the SVMP loci. These SVMPs comprise one PIII class (SVMP-17) and one PII class (SVMP-18), and they are close to each other, which may indicate a similar evolutionary pressure shaping their expression levels. However, further epigenomics studies must be performed in *B. alternatus* to better understand the genetic regulatory network governing the expression level observed of SVMP genes.

We retrieved the complete SVSP array in *B. alternatus*, which is composed of 9 SVSP genes arranged in tandem arrays. This pattern was also reported in *B. jararaca* and other vipers [19]. Moreover, the number of SVSP genes detected is similar to that observed in the venom gland transcriptomic data of *B. alternatus* [51], but this number is lower than that identified in the *B. jararaca* genome [19]. This may be a result of lineage-specific duplications in *B. jararaca* or deletions in *B. alternatus*; however, further genomic studies includ-

ing other *Bothrops* species must be performed to better understand the evolutionary history of SVSP genes in lancehead snakes.

We identified 11 CTL genes, from which 8 were highly expressed and 3 were lowly expressed. Differently from the other multicopy toxin families (PLA2, SVMP, and SVSP) that were clustered in a single contig, CTLs were spread in different genomic contigs and are arranged in pairs with an inverted pattern. Each pair has an average distance of 30Kb between them. Moreover, we also detected that two pairs of CTLs (*i.e.*, CTL-1 and CTL-2 pair and CTL-9 and CTL-10 pair) were composed of alpha and beta chain representatives (Figure S4 in Supplementary file 1; see Supplementary file 5 for alignment and tree). Interestingly, the genomic context observed in CTLs have a similar pattern to that observed in the crotamine toxin genes in *C. viridis* [91], which is a toxin uniquely identified in some *Crotalus* species. It indicates that such arrangements may be also present in other toxins not deeply analyzed in genomics studies. The genomic context and arrangement of CTLs has not been previously described, but the draft assembly still leaves an open question of whether the CTL loci are located in the same chromosome region and if they are organized in tandem arrays. In this sense, assembling a chromosome-level genome of *B. alternatus* and other *Bothrops* species may help to elucidate whether this arrangement is broadly conserved and decipher the evolutionary history of CTL genes. This will bring fruitful insights about their biological roles and the regulatory mechanisms shaping the expression levels of CTLs in the venom gland of the *Bothrops* genus and other viperids as well.

All other toxin genes were identified as single copy genes with a similar genome context to that previously observed in *B. jararaca* [19] and other *Crotalus* species [17, 48]. In fact, we were able to characterize the toxin repertoire of the species, which may help in future research focusing on the evolution of toxins and solving the common ancestor toxin repertoire of *Bothrops* as well as in viper species.

Discussion

The revolution in genome sequencing technologies has broadly transformed biological studies across research fields, including venomics, which mainly focuses on non-model organisms. However, the genome annotation tools currently available do not handle the issues accompanying the annotation of toxins, which can be extremely laborious and challenging. In fact, none of the genomes of venomous lineages published provide an automated script or a detailed description and documentation of the toxin annotation strategies applied, which hinders the reproducibility of results. Here, we provide a convenient computational tool, ToxCodAn-Genome, that can quickly identify most toxins in the genome, thereby minimizing the workload of checking toxin annotations and allowing improved reproducibility in further studies.

Our tests revealed that ToxCodAn-Genome can retrieve toxin annotations on virtually any venomous lineage by using a custom toxin database and/or species-specific venom-tissue transcriptomic data. We noticed that the integration of both datasets (*i.e.*, a toxin database and a venom tissue transcriptome) improves the final toxin annotation. Despite the high performance of ToxCodAn-Genome across all venomous clades tested, we noticed that a few limitations emerged, which can be related to: (1) the diversity of toxin sequences availability in the toxin database, which can be improved by surveying more toxin sequences in literature and by the use a well-curated venom tissue transcriptome of the target species; and (2) the quality of the genome assembly being analyzed, which may disrupt the final toxin annotation; however, this is an extrinsic issue not related to the capabilities of ToxCodAn-Genome. To bypass such limitations, we designed a guide to help the users to improve the final toxin set. This guide was designed to direct the user to take advantage of all outputs generated by ToxCodAn-Genome, to learn how to improve the final toxin annotation by checking specific genomic regions (*i.e.*, toxin matched regions with no annotations as identified by ToxCodAn-Genome), and to ensure a well-annotated genome. The guide also contains detailed descriptions of the processes to build a custom toxin database when venom-tissue transcriptomic data is available or not, to perform nontoxin annotation, to quantify gene expression, and to plot the toxin loci for reports and publications.

The proof-of-concept test showed that ToxCodAn-Genome can annotate most of the toxins in the genome, which integrates the set of highly expressed toxins in the venom tissue transcriptome. The few missing toxin genes were not annotated due to fragmented or unresolved genome assembly in the toxin regions, which may generate partial toxin annotations, or due to their genomic architecture that were not in the default range considered by ToxCodan-Genome. The genome assembly quality is a feature extrinsic to ToxCodAn-Genome, whereas the genomic architecture parameters can be modified to allow the user to retrieve a complete set of annotated toxins.

It is important to note that ToxCodAn-Genome was designed to be customizable and the user can test distinct parameters to improve the final toxin annotation set for the studied lineage. For instance, the user can set different percent identity thresholds, gene sizes, CDS lengths, and include or not a custom toxin database generated with published and/or unpublished data. Additionally, the user can include the UniProt or ToxProt databases to generate a report containing the best match between the annotated toxins and the database entries. Finally, the user can follow the guide to better interpret the outputs and fill the gaps of the limitations observed in the current tests.

Despite the availability of toxin databases for only a few venomous lineages to date, ToxCodAn-Genome can be expanded to annotate any venomous clade and species by using a specific set of full-length toxin CDSs. The user can follow our guide to design specific toxin databases by surveying sequences and/or analyzing venom-tissue transcriptomic data available in several databases, such as GenBank and TSA from NCBI, ENA from EMBL, and China National GeneBank DataBase (CNGBdb). Moreover, the constant expansion of genomic and transcriptomic data deposited and available for venomous lineages in these databases will allow us to keep these pre-designed toxin databases up-to-date and also expand the set of toxin databases to encompass other venomous clades in the near future [40].

ToxCodAn-Genome can be easily installed on any UNIX-like operating system and is fast, taking only a few minutes to analyze a genome in a personal computer (Intel 6-Core i7 with 16-Gb memory). These resources are available on most modern desktop and laptop computers, demonstrating the applicability of ToxCodAn-Genome for projects of any size, regardless of available computational resources. Moreover, the fast running time allows the user to perform several tests with distinct parameters to reach a high-quality final toxin annotation set.

ToxCodAn-Genome allowed us to easily characterize the toxin gene repertoire of *B. alternatus*. It revealed that the most abundant toxin families comprising the venom of *B. alternatus* and also in other Viperidae species are those that underwent more expansion (*i.e.*, SVMP, SVSP, PLA2, and CTL). The first complete SVMP locus obtained for a lancehead revealed a similar genomic context to that observed in other viperids [17, 42, 19, 48], with the SVMP gene located closer to the ADAM28 gene being the lowest expressed among all SVMP genes. The other loci presented a similar genomic context as previously described, but we also identified an interesting genomic arrangement of CTL genes, which may be further analyzed using chromosome-level genomes to confirm if this pattern is widely conserved among vipers. Moreover, the draft genome assembly and the complete toxin repertoire obtained for *B. alternatus* in the present study can be a useful resource for further experiments focusing on better understanding the intraspecific variation

of venom composition observed in *B. alternatus* [77, 78, 51]. Such experiments can increase the sampling size and apply genomic and epigenomic approaches to reveal if it may be related to deletion and/or duplication events within toxin genes [42] or if it may be related to nucleotide changes in promoter and enhancer regions of these toxin genes [6]. Furthermore, the current assembly and toxin annotation can be integrated into comparative analysis with other *Bothrops* and viper species to reconstruct the toxin genomic repertoire of their common ancestor and improve the evolutionary history of venom components within the genus and also within viperids [42].

The genome annotation step of *B. alternatus* revealed that even sophisticated approaches, like funannotate, which integrates several tools and strategies in their pipeline to perform an automated genome annotation, fails to correctly annotate the entire set of toxin genes (*i.e.*, only 14 from a total of 59 toxin genes; Supplementary file 4). It reveals that common features considered when annotating most genes do not fit well when annotating toxin genes, which are genomic regions commonly accompanied by high mutation rates, recent duplication and loss events, and the presence of orphan exons. Our case study and previous reports show that extra features are needed to be considered when annotating and studying toxin genes [43, 15, 16, 44, 17, 19, 48, 21, 49]. In this sense, ToxCodAn-Genome takes into consideration key features to correctly annotate toxins (*i.e.*, 49 from 59 toxin genes in *B. alternatus*; Supplementary file 4), but it still needs improvements to solve some pitfalls related to automatically confirm the status of "warning" annotations as truncated paralogs or pseudogenes (*i.e.*, 1 from 59 toxin genes in *B. alternatus*), and to better interpret matched regions with no annotations (*i.e.*, 9 from 59 toxin genes in *B. alternatus*). Of note, the constant expansion of available high-quality genomes and well-annotated toxin annotations of venomous lineages may represent an outstanding opportunity to apply machine learning algorithms to help on the toxin annotation task in the near future [92].

Although ToxCodAn-Genome performed very satisfactory in the tests performed here, users should be aware of some limitations: (1) Our tool does not perform annotation of partial genes located in fragmented or incomplete genomic contigs; (2) ToxCodAn-Genome only consider canonical start and stop codons, and splicing sites, which may inhibit the annotation of toxin genes with non-canonical signals; (3) ToxCodAn-Genome is dependent on the user knowledge about the toxin gene repertoire of the species being studied to set and test the best parameters for the species being studied. Such limitations may be surpassed in further updates by integrating pre-built and self-training gene models to predict the toxin gene structures, and also integrating the possibility to consider non-canonical start and stop codons and splicing sites, which can be set by the user. Also, the user's knowledge about the toxins and putative toxins may help to better characterize the complete set of toxins in the analyzed genome and can be acquired in scientific resources, like VenomZone [62], ToxProt [63], ConoServer [64], ArachnoServer [65], and scientific literature. Of note, we intend to keep ToxCodAn-Genome up to date by releasing a major update every year, such updates will include improvements in the code to retrieve better performance in toxin annotations and integration of novel high-performance tools, and increasing the toxin database entries, as soon as more genomes and transcriptomes of venomous lineages are being available in the years to come. Also, we are open to receive feedback to improve the tool and add the toxin sequences annotated and/or entire custom toxin databases designed by users who want to assist ToxCodAn-Genome and the scientific community working with venomous lineages.

ToxCodAn-Genome was designed to annotate toxin genes, however, we believe that it may also be applied to annotate analogous cases of functional gene categories presenting similar genomic features to those observed in toxin families. For example, chemosensory genes [67, 93], opsin genes [94], olfactory receptor genes [95], major histocompatibility complex genes [96], fetuin metalloproteinase inhibitor genes [97], hox genes [98], and other gene families expanded during evolution and adaptation of specific lineages can, in theory, be annotated by this tool. In fact, these genomic regions are poorly characterized by automated genome annotation tools and require laborious manual inspection to accurately annotate and identify the complete set of genes [39, 38]. In this sense, ToxCodAn-Genome may represent a suitable tool to help on specific gene type annotation tasks and improve research on any genomic study.

Conclusion

ToxCodAn-Genome is the first tool that can be easily applied to annotate toxin genes in genome assemblies of any venomous species. It is fast and suitable for use on projects of any size. We also provide a guide to help researchers perform such toxin gene annotations and also check for truncated paralogs and pseudogenes. We provide pre-built toxin databases for snakes (Viperidae and Elapidae clades), Myliobatoidei, Scorpiones, Hymenoptera, and Anthozoa, which can be integrated to the use of venom-tissue transcriptomic data. Moreover, ToxCodAn-Genome can be expanded to use in any venomous lineages by designing novel and custom toxin databases and also using venom transcriptomic data specific to the studied lineage. In addition, through our study case, we revealed the toxin genomic repertoire of the urutu lancehead, a widely distributed pit-viper in South America.

Availability of source code and requirements

- Project name: ToxCodAn-Genome
- Project home page: `https://github.com/pedronachtigall/ToxCodAn-Genome`
- Operating system: UNIX
- Programming language: Python
- Other requirements: Biopython, Pandas, BLAST, Exonerate, and GffRead
- License: GNU GPLv3
- Biotools ID: toxcodan-genome
- RRID: SCR_024718

Data Availability

The genome assembly and the PacBio HiFi reads of *B. alternatus* are available under the accession numbers JARGCV000000000 and SRR23725375 in the NCBI [59]. Besides, the assembled genome [99], annotations [100, 101], and BUSCO analysis [102] are available in the figshare database. ToxCodAn-Genome and the guide are freely available via the GitHub repository [103, 58]. An archival copy of the code and supporting data is available via the GigaScience repository, GigaDB 102487 [104].

Declarations

List of abbreviations

CDS - Coding sequence; PLA2 - Phospholipase A_2; SVMP - Snake venom metalloproteinase; SVSP - Snake venom serine proteinase; CTL - C-type lectin; BPP - Bradykinin potentiating peptide; VEGF-F - Snake venom vascular endothelial growth factor; LAO/LAAO - L-amino Acid Oxidase; CRISP - Cysteine-rich secretory protein; HYAL - Hyaluronidase; NGF - Venom nerve growth factor; PDE - phosphodiesterase; PLB - Phospholipase B; NUC - Snake venom 5′ nucleotidase; TPM - Transcripts per million

Ethical Approval

The snake specimen was handled and collected under Protocol Number 4479020217 from the Ethic Committee on Animal Use of the Butantan Institute (CEUAIB).

Consent for publication

Not applicable.

Competing Interests

The authors declare no competing interests.

Funding

This work was supported by Fundação de Amparo à Pesquisa no Estado de São Paulo (FAPESP processes numbers: 2013/07467-1, 2016/50127-5, 2018/26520-4, and 2022/04988-0) and National Science Foundation (NSF DEB 1638902).

Author's Contributions

PGN and ILMJA conceived and designed the experiments. PGN wrote the python script and performed all experiments and bioinformatics analysis. PGN, AMD, DRR, and ILMJA analyzed the data. PGN wrote the manuscript. PGN, AMD, DRR, and ILMJA critically edited the final manuscript. All authors read and approved the final manuscript.

Acknowledgements

We thank Arthur D. Abegg for the photograph of *B. alternatus*. The authors thank the reviewers Choo Hock Tan, Zachary Kenneth Stewart, and Jason Macrander for their valuable suggestions.

References

1. Giani AM, Gallo GR, Gianfranceschi L, Formenti G. Long walk to genomics: History and current approaches to genome sequencing and assembly. Computational and Structural Biotechnology Journal 2020;18:9–19.
2. Lewin HA, Robinson GE, Kress WJ, Baker WJ, Coddington J, Crandall KA, et al. Earth BioGenome Project: Sequencing life for the future of life. Proceedings of the National Academy of Sciences 2018;115(17):4325–4333.
3. Bordon KdCF, Cologna CT, Fornari-Baldo EC, Pinheiro-Júnior EL, Cerni FA, Amorim FG, et al. From animal poisons and venoms to medicines: achievements, challenges and perspectives in drug discovery. Frontiers in pharmacology 2020;11:1132.
4. Casewell NR, Jackson TN, Laustsen AH, Sunagar K. Causes and consequences of snake venom variation. Trends in pharmacological sciences 2020;41(8):570–581.
5. Barua A, Mikheyev AS. An ancient, conserved gene regulatory network led to the rise of oral venom systems. Proceedings of the National Academy of Sciences 2021;118(14):e2021311118.
6. Perry BW, Gopalan SS, Pasquesi GI, Schield DR, Westfall AK, Smith CF, et al. Snake venom gene expression is coordinated by novel regulatory architecture and the integration of multiple co-opted vertebrate pathways. Genome Research 2022;32(6):1058–1073.
7. Zancolli G, Reijnders M, Waterhouse RM, Robinson-Rechavi M. Convergent evolution of venom gland transcriptomes across Metazoa. Proceedings of the National Academy of Sciences 2022;119(1):e2111392119.
8. Zancolli G, Casewell NR. Venom systems as models for studying the origin and regulation of evolutionary novelties. Molecular Biology and Evolution 2020;37(10):2777–2790.
9. Drukewitz SH, Von Reumont BM. The significance of comparative genomics in modern evolutionary venomics. Frontiers in Ecology and Evolution 2019;7:163.
10. Fry BG, Vidal N, Van der Weerd L, Kochva E, Renjifo C. Evolution and diversification of the Toxicofera reptile venom system. Journal of proteomics 2009;72(2):127–136.
11. Casewell NR, Wüster W, Vonk FJ, Harrison RA, Fry BG. Complex cocktails: the evolutionary novelty of venoms. Trends in ecology & evolution 2013;28(4):219–229.
12. Fry BG, Vidal N, Norman JA, Vonk FJ, Scheib H, Ramjan SR, et al. Early evolution of the venom system in lizards and snakes. Nature 2006;439(7076):584–588.
13. Post Y, Puschhof J, Beumer J, Kerkkamp HM, de Bakker MA, Slagboom J, et al. Snake venom gland organoids. Cell 2020;180(2):233–247.
14. Rádis-Baptista G. Cell-penetrating peptides derived from animal venoms and toxins. Toxins 2021;13(2):147.
15. Vonk FJ, Casewell NR, Henkel CV, Heimberg AM, Jansen HJ, McCleary RJ, et al. The king cobra genome reveals dynamic gene evolution and adaptation in the snake venom system. Proceedings of the National Academy of Sciences 2013;110(51):20651–20656.
16. Yin W, Wang Zj, Li Qy, Lian Jm, Zhou Y, Lu Bz, et al. Evolutionary trajectories of snake genes and genomes revealed by comparative analyses of five-pacer viper. Nature communications 2016;7(1):1–11.
17. Schield DR, Card DC, Hales NR, Perry BW, Pasquesi GM, Blackmon H, et al. The origins and evolution of chromosomes, dosage compensation, and mechanisms underlying venom regulation in snakes. Genome research 2019;29(4):590–601.
18. Peng C, Ren JL, Deng C, Jiang D, Wang J, Qu J, et al. The genome of Shaw's sea snake (Hydrophis curtus) reveals secondary adaptation to its marine environment. Molecular Biology and Evolution 2020;37(6):1744–1760.
19. Almeida DD, Viala VL, Nachtigall PG, Broe M, Gibbs HL, Serrano SMdT, et al. Tracking the recruitment and evolution of snake toxins using the evolutionary context provided by the Bothrops jararaca genome. Proceedings of the National Academy of Sciences 2021;118(20):e2015159118.
20. Myers EA, Strickland JL, Rautsaw RM, Mason AJ, Schramer TD, Nystrom GS, et al. De Novo Genome Assembly Highlights the Role of Lineage-Specific Gene Duplications in the Evolution of Venom in Fea's Viper (Azemiops feae). Genome biology and evolution 2022;14(7):evac082.
21. Zhang ZY, Lv Y, Wu W, Yan C, Tang CY, Peng C, et al. The structural and functional divergence of a neglected three-finger toxin subfamily in lethal elapids. Cell Reports 2022;40(2):111079.
22. Yandell M, Ence D. A beginner's guide to eukaryotic genome annotation. Nature Reviews Genetics 2012;13(5):329–342.
23. Richardson EJ, Watson M. The automatic annotation of bacterial genomes. Briefings in bioinformatics 2013;14(1):1–12.
24. Salzberg SL. Next-generation genome annotation: we still struggle to get it right. Genome biology 2019;20(1):1–3.
25. Ejigu GF, Jung J. Review on the computational genome annotation of sequences obtained by next-generation sequencing. Biology 2020;9(9):295.
26. Stanke M, Waack S. Gene prediction with a hidden Markov model and a new intron submodel. Bioinformatics 2003;19(suppl_2):ii215–ii225.
27. Korf I. Gene finding in novel genomes. BMC bioinformatics 2004;5(1):1–9.
28. Hoff KJ, Lomsadze A, Borodovsky M, Stanke M. Whole-

genome annotation with BRAKER. Gene prediction: methods and protocols 2019;p. 65–95.
29. Bruna T, Hoff KJ, Lomsadze A, Stanke M, Borodovsky M. BRAKER2: automatic eukaryotic genome annotation with GeneMark-EP+ and AUGUSTUS supported by a protein database. NAR genomics and bioinformatics 2021;3(1):lqaa108.
30. Cantarel BL, Korf I, Robb SM, Parra G, Ross E, Moore B, et al. MAKER: an easy-to-use annotation pipeline designed for emerging model organism genomes. Genome research 2008;18(1):188–196.
31. Bruna T, Lomsadze A, Borodovsky M. GeneMark-EP+: eukaryotic gene prediction with self-training in the space of genes and proteins. NAR genomics and bioinformatics 2020;2(2):lqaa026.
32. Solovyev V, Kosarev P, Seledsov I, Vorobyev D. Automatic annotation of eukaryotic genes, pseudogenes and promoters. Genome biology 2006;7(1):1–12.
33. Keilwagen J, Hartung F, Grau J. GeMoMa: homology-based gene prediction utilizing intron position conservation and RNA-seq data. Gene prediction: Methods and protocols 2019;p. 161–177.
34. Shumate A, Salzberg SL. Liftoff: accurate mapping of gene annotations. Bioinformatics 2021;37(12):1639–1643.
35. Kirilenko BM, Munegowda C, Osipova E, Jebb D, Sharma V, Blumer M, et al. Integrating gene annotation with orthology inference at scale. bioRxiv 2022;p. 2022–09.
36. Lu J, Salzberg SL. Removing contaminants from databases of draft genomes. PLoS computational biology 2018;14(6):e1006277.
37. Mudge JM, Harrow J. The state of play in higher eukaryote gene annotation. Nature Reviews Genetics 2016;17(12):758–772.
38. Peel E, Silver L, Brandies P, Zhu Y, Cheng Y, Hogg CJ, et al. Best genome sequencing strategies for annotation of complex immune gene families in wildlife. GigaScience 2022;11.
39. Frankish A, Diekhans M, Ferreira AM, Johnson R, Jungreis I, Loveland J, et al. GENCODE reference annotation for the human and mouse genomes. Nucleic acids research 2019;47(D1):D766–D773.
40. von Reumont BM, Anderluh G, Antunes A, Ayvazyan N, Beis D, Caliskan F, et al. Modern venomics—Current insights, novel methods, and future perspectives in biological and applied animal venom research. GigaScience 2022;11.
41. Dowell N, Giorgianni M, Kassner V, Selegue J, Sanchez E, Carroll S. The deep origin and recent loss of venom toxin genes in rattlesnakes. Current Biology 2016;26(18):2434–2445.
42. Giorgianni MW, Dowell NL, Griffin S, Kassner VA, Selegue JE, Carroll SB. The origin and diversification of a novel protein family in venomous snakes. Proceedings of the National Academy of Sciences 2020;117(20):10911–10920.
43. Cao Z, Yu Y, Wu Y, Hao P, Di Z, He Y, et al. The genome of Mesobuthus martensii reveals a unique adaptation model of arthropods. Nature communications 2013;4(1):1–10.
44. Drukewitz SH, Bokelmann L, Undheim EA, von Reumont BM. Toxins from scratch? Diverse, multimodal gene origins in the predatory robber fly Dasypogon diadema indicate a dynamic venom evolution in dipteran insects. GigaScience 2019;8(7):giz081.
45. Smith EG, Surm JM, Macrander J, Simhi A, Amir G, Sachkova MY, et al. Micro and macroevolution of sea anemone venom phenotype. Nature Communications 2023;14(1):249.
46. Koludarov I, Jackson TN, Suranse V, Pozzi A, Sunagar K, Mikheyev AS. Reconstructing the evolutionary history of a functionally diverse gene family reveals complexity at the genetic origins of novelty. BioRxiv 2020;p. 583344.
47. Barua A, Koludarov I, Mikheyev AS. Co-option of the same ancestral gene family gave rise to mammalian and reptilian toxins. BMC biology 2021;19(1):1–12.
48. Margres MJ, Rautsaw RM, Strickland JL, Mason AJ, Schramer TD, Hofmann EP, et al. The Tiger Rattlesnake genome reveals a complex genotype underlying a simple venom phenotype. Proceedings of the National Academy of Sciences 2021;118(4):e2014634118.
49. Ye X, Yang Y, Zhao C, Xiao S, Sun YH, He C, et al. Genomic signatures associated with maintenance of genome stability and venom turnover in two parasitoid wasps. Nature communications 2022;13(1):1–17.
50. Slater GSC, Birney E. Automated generation of heuristics for biological sequence comparison. BMC bioinformatics 2005;6(1):1–11.
51. Nachtigall PG, Rautsaw RM, Ellsworth SA, Mason AJ, Rokyta DR, Parkinson CL, et al. ToxCodAn: a new toxin annotator and guide to venom gland transcriptomics. Briefings in Bioinformatics 2021;22(5):bbab095.
52. Macrander J, Panda J, Janies D, Daly M, Reitzel AM. Venomix: a simple bioinformatic pipeline for identifying and characterizing toxin gene candidates from transcriptomic data. PeerJ 2018;6:e5361.
53. Holding ML, Margres MJ, Mason AJ, Parkinson CL, Rokyta DR. Evaluating the performance of de novo assembly methods for venom-gland transcriptomics. Toxins 2018;10(6):249.
54. Kim D, Paggi JM, Park C, Bennett C, Salzberg SL. Graph-based genome alignment and genotyping with HISAT2 and HISAT-genotype. Nature biotechnology 2019;37(8):907–915.
55. Pertea M, Pertea GM, Antonescu CM, Chang TC, Mendell JT, Salzberg SL. StringTie enables improved reconstruction of a transcriptome from RNA-seq reads. Nature biotechnology 2015;33(3):290–295.
56. Haas BJ, Papanicolaou A, Yassour M, Grabherr M, Blood PD, Bowden J, et al. De novo transcript sequence reconstruction from RNA-seq using the Trinity platform for reference generation and analysis. Nature protocols 2013;8(8):1494–1512.
57. Bushmanova E, Antipov D, Lapidus A, Prjibelski AD. rnaSPAdes: a de novo transcriptome assembler and its application to RNA-Seq data. GigaScience 2019;8(9):giz100.
58. Nachtigall PG, Guide to annotate toxin genes in the genome of venomous lineages; 2023. https://github.com/pedronachtigall/ToxCodAn-Genome/tree/main/Guide.
59. Sayers EW, Bolton EE, Brister JR, Canese K, Chan J, Comeau DC, et al. Database resources of the National Center for Biotechnology Information in 2023. Nucleic acids research 2023;51(D1):D29–D38.
60. Fu L, Niu B, Zhu Z, Wu S, Li W. CD-HIT: accelerated for clustering the next-generation sequencing data. Bioinformatics 2012;28(23):3150–3152.
61. King GF, Gentz MC, Escoubas P, Nicholson GM. A rational nomenclature for naming peptide toxins from spiders and other venomous animals. Toxicon 2008;52(2):264–276.
62. Jungo F, Castro E, group SP, editor, VenomZone. SIB Swiss Institute of Bioinformatics; 2017. Accessed November 20, 2023. https://venomzone.expasy.org/.
63. Jungo F, Bougueleret L, Xenarios I, Poux S. The UniProtKB/Swiss-Prot Tox-Prot program: a central hub of integrated venom protein data. Toxicon 2012;60(4):551–557.
64. Kaas Q, Yu R, Jin AH, Dutertre S, Craik DJ. ConoServer: updated content, knowledge, and discovery tools in the conopeptide database. Nucleic acids research 2012;40(D1):D325–D330.
65. Pineda SS, Chaumeil PA, Kunert A, Kaas Q, Thang MW, Le L, et al. ArachnoServer 3.0: an online resource for automated discovery, analysis and annotation of spider toxins. Bioinformatics 2018;34(6):1074–1076.
66. Kirchhoff KN, Billion A, Voolstra CR, Kremb S, Wilke T, Vilcinskas A. Stingray venom proteins: Mechanisms of action revealed using a novel network pharmacology approach. Marine Drugs 2022;20(1):27.

67. Li A, Wang J, Sun K, Wang S, Zhao X, Wang T, et al. Two reference-quality sea snake genomes reveal their divergent evolution of adaptive traits and venom systems. Molecular Biology and Evolution 2021;38(11):4867–4883.
68. Suryamohan K, Krishnankutty SP, Guillory J, Jevit M, Schröder MS, Wu M, et al. The Indian cobra reference genome and transcriptome enables comprehensive identification of venom toxins. Nature Genetics 2020;52(1):106–117.
69. Zhou J, Liu A, He F, Zhang Y, Shen L, Yu J, et al. Draft Genome of White-blotched River Stingray Provides Novel Clues for Niche Adaptation and Skeleton Formation. Genomics, Proteomics & Bioinformatics 2022;.
70. Park D, Jung JW, Choi BS, Jayakodi M, Lee J, Lim J, et al. Uncovering the novel characteristics of Asian honey bee, Apis cerana, by whole genome sequencing. BMC genomics 2015;16(1):1–16.
71. Wallberg A, Bunikis I, Pettersson OV, Mosbech MB, Childers AK, Evans JD, et al. A hybrid de novo genome assembly of the honeybee, Apis mellifera, with chromosome-length scaffolds. BMC genomics 2019;20:1–19.
72. De Graaf DC, Aerts M, Brunain M, Desjardins CA, Jacobs FJ, Werren JH, et al. Insights into the venom composition of the ectoparasitoid wasp Nasonia vitripennis from bioinformatic and proteomic studies. Insect molecular biology 2010;19:11–26.
73. Fletcher C, da Conceicoa LP, of Life Consortium DT, et al. The genome sequence of the starlet sea anemone, Nematostella vectensis (Stephenson, 1935). Wellcome Open Research 2023;8(79):79.
74. Li B, Dewey CN. RSEM: accurate transcript quantification from RNA-Seq data with or without a reference genome. BMC bioinformatics 2011;12:1–16.
75. Martins M, Marques OA, Sazima I. Ecological and phylogenetic correlates of feeding habits in Neotropical pitvipers of the genus Bothrops. Biology of the Vipers 2002;307:328.
76. Nogueira CC, Argôlo AJ, Arzamendia V, Azevedo JA, Barbo FE, Bérnils RS, et al. Atlas of Brazilian snakes: verified point-locality maps to mitigate the Wallacean shortfall in a megadiverse snake fauna. South American Journal of Herpetology 2019;14(sp1):1–274.
77. Cardoso KC, Da Silva MJ, Costa GG, Torres TT, Del Bem LEV, Vidal RO, et al. A transcriptomic analysis of gene expression in the venom gland of the snake Bothrops alternatus (urutu). BMC genomics 2010;11(1):1–22.
78. de Paula FFP, Ribeiro JU, Santos LM, de Souza DHF, Leonardecz E, Henrique-Silva F, et al. Molecular characterization of metalloproteases from Bothrops alternatus snake venom. Comparative Biochemistry and Physiology Part D: Genomics and Proteomics 2014;12:74–83.
79. Cheng H, Concepcion GT, Feng X, Zhang H, Li H. Haplotype-resolved de novo assembly using phased assembly graphs with hifiasm. Nature methods 2021;18(2):170–175.
80. Chen Y, Zhang Y, Wang AY, Gao M, Chong Z. Accurate long-read de novo assembly evaluation with Inspector. Genome biology 2021;22(1):1–21.
81. Waterhouse RM, Seppey M, Simão FA, Manni M, Ioannidis P, Klioutchnikov G, et al. BUSCO applications from quality assessments to gene prediction and phylogenomics. Molecular biology and evolution 2018;35(3):543–548.
82. Ou S, Su W, Liao Y, Chougule K, Agda JR, Hellinga AJ, et al. Benchmarking transposable element annotation methods for creation of a streamlined, comprehensive pipeline. Genome biology 2019;20(1):1–18.
83. Freitas-de Sousa LA, Nachtigall PG, Portes-Junior JA, Holding ML, Nystrom GS, Ellsworth SA, et al. Size matters: an evaluation of the molecular basis of ontogenetic modifications in the composition of Bothrops jararacussu snake venom. Toxins 2020;12(12):791.
84. Nachtigall PG, Freitas-de Sousa LA, Mason AJ, Moura-da Silva AM, Grazziotin FG, Junqueira-de Azevedo IL. Differences in PLA2 constitution distinguish the venom of two endemic Brazilian mountain lanceheads, Bothrops cotiara and Bothrops fonsecai. Toxins 2022;14(4):237.
85. Palmer J, Stajich J, Funannotate: Eukaryotic genome annotation; 2017. https://github.com/nextgenusfs/funannotate.
86. Jones P, Binns D, Chang HY, Fraser M, Li W, McAnulla C, et al. InterProScan 5: genome-scale protein function classification. Bioinformatics 2014;30(9):1236–1240.
87. Rozewicki J, Li S, Amada KM, Standley DM, Katoh K. MAFFT-DASH: integrated protein sequence and structural alignment. Nucleic acids research 2019;47(W1):W5–W10.
88. Nguyen LT, Schmidt HA, Von Haeseler A, Minh BQ. IQ-TREE: a fast and effective stochastic algorithm for estimating maximum-likelihood phylogenies. Molecular biology and evolution 2015;32(1):268–274.
89. Rambaut A, FigTree; 2007. http://tree.bio.ed.ac.uk/software/figtree/. https://github.com/rambaut/figtree/.
90. Queiroz GP, Pessoa LA, Portaro FC, Maria de Fátima DF, Tambourgi DV. Interspecific variation in venom composition and toxicity of Brazilian snakes from Bothrops genus. Toxicon 2008;52(8):842–851.
91. Gopalan SS, Perry BW, Schield DR, Smith CF, Mackessy SP, Castoe TA. Origins, genomic structure and copy number variation of snake venom myotoxins. Toxicon 2022;216:92–106.
92. Whalen S, Schreiber J, Noble WS, Pollard KS. Navigating the pitfalls of applying machine learning in genomics. Nature Reviews Genetics 2022;23(3):169–181.
93. Hogan MP, Whittington AC, Broe MB, Ward MJ, Gibbs HL, Rokyta DR. The chemosensory repertoire of the Eastern Diamondback Rattlesnake (Crotalus adamanteus) reveals complementary genetics of olfactory and vomeronasal-type receptors. Journal of Molecular Evolution 2021;89:313–328.
94. Macias-Muñoz A, Murad R, Mortazavi A. Molecular evolution and expression of opsin genes in Hydra vulgaris. BMC genomics 2019;20(1):1–19.
95. Hughes GM, Boston ES, Finarelli JA, Murphy WJ, Higgins DG, Teeling EC. The birth and death of olfactory receptor gene families in mammalian niche adaptation. Molecular biology and evolution 2018;35(6):1390–1406.
96. Miller HC, O'Meally D, Ezaz T, Amemiya C, Marshall-Graves JA, Edwards S. Major histocompatibility complex genes map to two chromosomes in an evolutionarily ancient reptile, the tuatara Sphenodon punctatus. G3: Genes, Genomes, Genetics 2015;5(7):1439–1451.
97. Ukken FP, Dowell NL, Hajra M, Carroll SB. A novel broad spectrum venom metalloproteinase autoinhibitor in the rattlesnake Crotalus atrox evolved via a shift in paralog function. Proceedings of the National Academy of Sciences 2022;119(51):2214880119.
98. Gaunt SJ. Seeking sense in the Hox gene cluster. Journal of Developmental Biology 2022;10(4):48.
99. Nachtigall PG, Bothrops_alternatus_genome_assembly. figshare; 2023. https://doi.org/10.6084/m9.figshare.22227565.
100. Nachtigall PG, Bothrops_alternatus_toxin_annotation. figshare; 2023. https://doi.org/10.6084/m9.figshare.22227583.
101. Nachtigall PG, Bothrops_alternatus_genome_annotation. figshare; 2023. https://doi.org/10.6084/m9.figshare.22227580.
102. Nachtigall PG, Bothrops_alternatus_BUSCO_tetrapoda_odb10. figshare; 2023. https://doi.org/10.6084/m9.figshare.23631492.
103. Nachtigall PG, ToxCodAn-Genome; 2023. https://github.com/pedronachtigall/ToxCodAn-Genome.
104. Nachtigall PG, Durham AM, Rokyta DR, Junqueira-de Azevedo IL, Supporting data for "ToxCodAn-Genome: an automated

pipeline for toxin-gene annotation in genome assembly of venomous lineages" GigaScience Database; 2023. http://dx.doi.org/10.5524/102487.

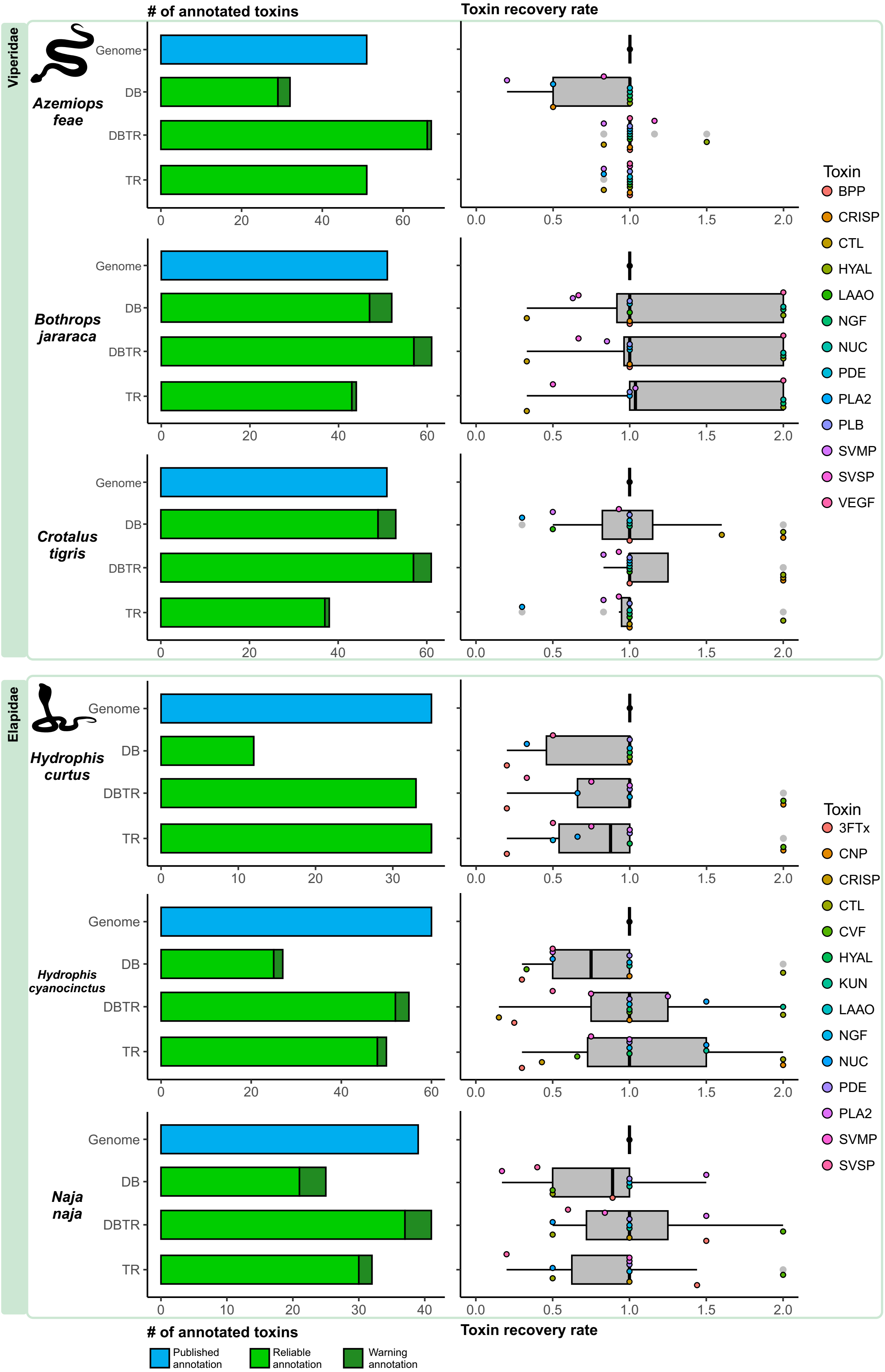

Figure 2. ToxCodAn-Genome performance in Viperidae and Elapidae testing sets. The barplots at the left indicate the number of toxins annotated in the published genome and in the tested scenarios. The genome represents the number of annotations as stated in the published manuscript (represented in blue color), whereas the ToxCodAn-Genome outputs are classified as "reliable" (*i.e*, confident toxin annotations; represented in green color) and "warning" (*i.e*, annotations that need further inspections; represented in dark green color). The boxplots at right represent the toxin recovery rate (TRR) for major components of venom within each clade. The TRR is calculated as described in the methods section. DB, ToxCodAn-Genome annotation using the toxin database only. DBTR, ToxCodAn-Genome annotation using the toxin database and the species-specific toxin-annotated transcriptome. TR, ToxCodAn-Genome annotations using the species-specific toxin-annotated transcriptome only.

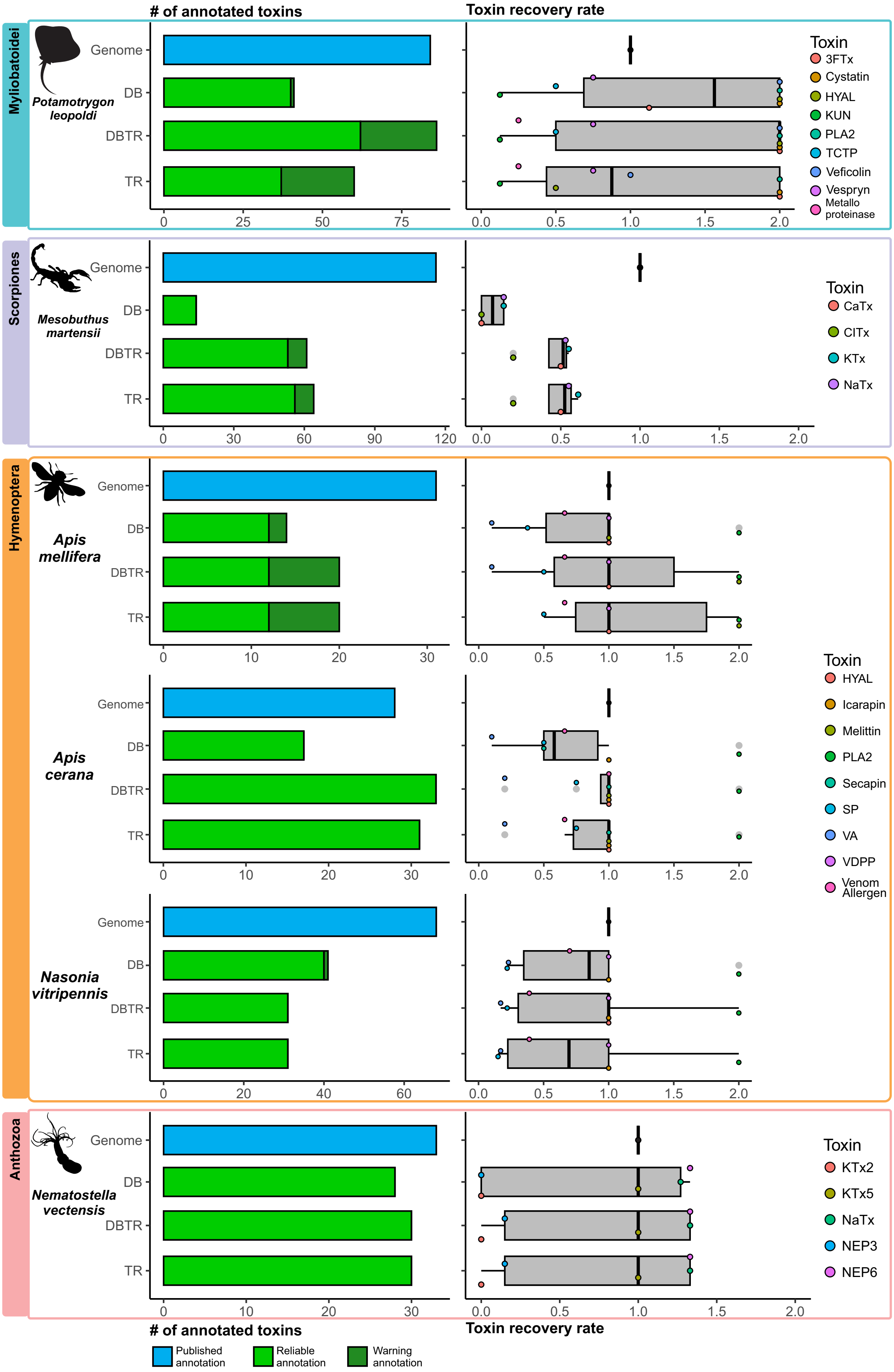

Figure 3. ToxCodAn-Genome performance in Myliobatoidea, Scorpiones, Hymenoptera, and Anthozoa testing sets. The barplots at the left indicate the number of toxins annotated in the published genome and in the tested scenarios. The genome represents the number of annotations as stated in the published manuscript (represented in blue color), whereas the ToxCodAn-Genome outputs are classified as "reliable" (*i.e*, confident toxin annotations; represented in green color) and "warning" (*i.e*, annotations that need further inspections; represented in dark green color). The boxplots at right represent the toxin recovery rate (TRR) for major components of venom within each clade. The TRR is calculated as described in the methods section. DB, ToxCodAn-Genome annotation using the toxin database only. DBTR, ToxCodAn-Genome annotation using the toxin database and the species-specific toxin-annotated transcriptome. TR, ToxCodAn-Genome annotations using the species-specific toxin-annotated transcriptome only.

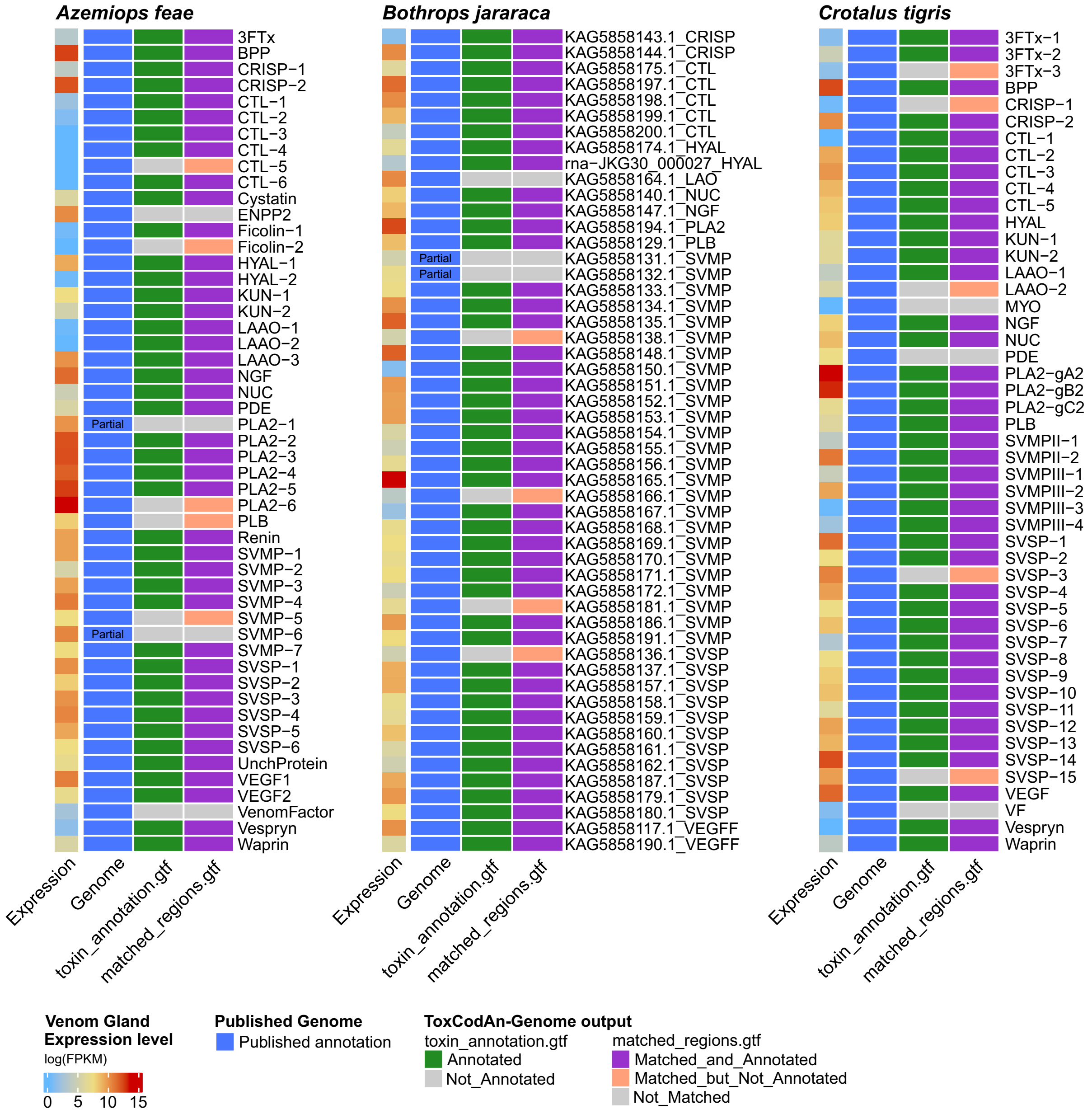

Figure 4. ToxCodAn-Genome performance to correctly annotate toxin genes and detect them as putative toxin loci using the Viperidae testing sets. The rows represent the toxin genes within each species' genome, whereas the columns show the expression level, published genome annotation and ToxCodAn-Genome output. The expression level was estimated using the venom gland transcriptome. The genome annotation shows mainly full-length toxins annotated in the genome, but also shows when that specific toxin was partially annotated due to fragmented or incomplete genomic regions. The "toxin_annotation.gtf" shows when the toxin was annotated, in green color, or not annotated, in gray color. The "matched_regions.gtf" shows when the toxin was identified as a putative toxin loci and annotated, in purple color, or not annotated, in salmon color, or when it was not identified as a putative toxin loci, in gray color.

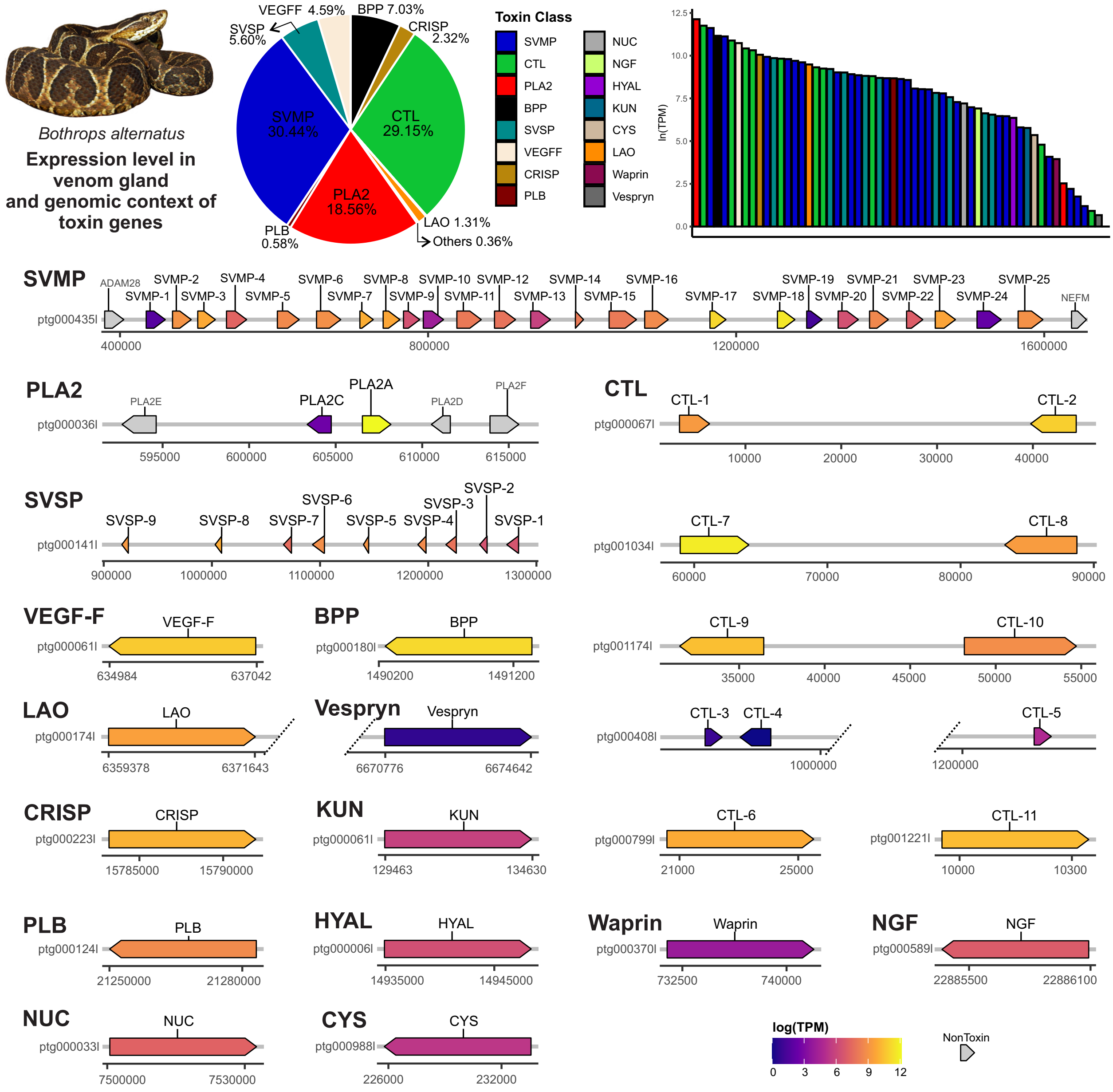

Figure 5. Expression level in venom gland and genomic context of toxin genes of *Bothrops alternatus*. The pie chart and barplot at the top refer to the expression level of toxin genes in the venom gland transcriptome, in which the toxins are color coded by their toxin class. The toxin loci plots at the bottom are color coded by expression level and the nontoxin genes flanking toxin loci are coloured in light gray. BPP, bradykinin-potentiating peptides; CRISP, cysteine-rich secretory proteins; CTL, C-type lectins; CYS, Cystatin. HYAL, hyaluronidase; KUN, Kunitz-type proteinase inhibitor; LAO, L-amino acid oxidase; NGF, nerve growth factor; NUC, Ecto 5' nucleotidase; PDE, phosphodiesterase; PLA2, phospholipase A2; PLB, phospholipase B; SVMP, snake venom metalloproteinase; SVSP, snake venom serine protease; VEGF-F, vascular endothelial growth factor.

Supplementary_file_1

Click here to access/download

Supplementary Material

Supplementary_file_1.pdf

Supplementary_file_2

Click here to access/download
Supplementary Material
Supplementary_file_2.pdf

Supplementary_file_3

Click here to access/download
Supplementary Material
Supplementary_file_3.xlsx

Supplementary_file_4

Click here to access/download
Supplementary Material
Supplementary_file_4.pdf

Supplementary_file_5

Click here to access/download
Supplementary Material
CTL_alphabeta_ALIGNED.fasta

Supplementary_file_5

Click here to access/download

Supplementary Material

CTL_alphabeta_ALIGNED.fasta.contree
