# Supplementary material for: ToxCodAn-Genome: an automated pipeline for toxin-gene annotation in genome assembly of venomous lineages
Source: Gigascience. 2024 Jan 18;13:giad116. doi: 10.1093/gigascience/giad116 (PMC10797961; doi:10.1093/gigascience/giad116)
Supplement: giad116_Supplemental_Files [file giad116_supplemental_files.zip › Supplementary_file_1.pdf]

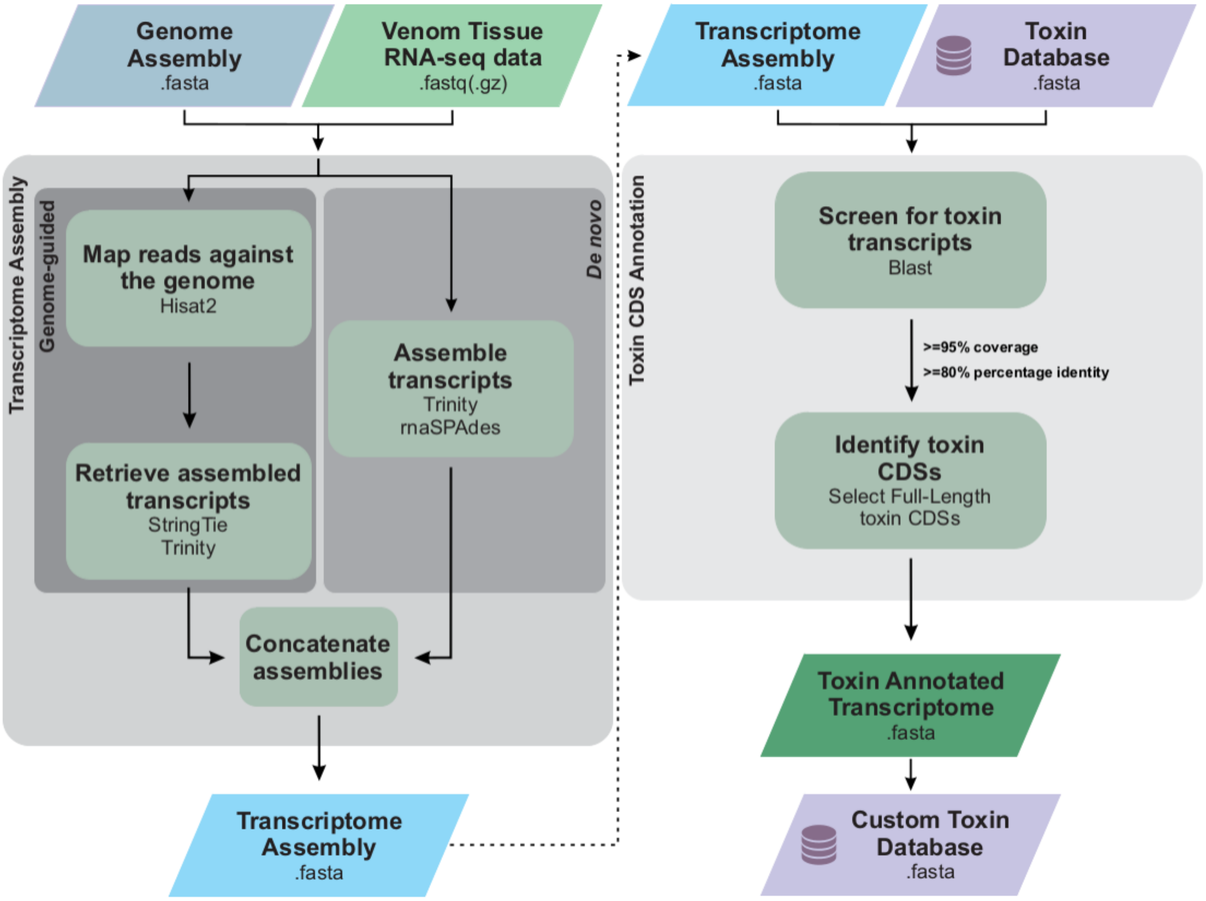

Figure S1. The "Transcriptome assembly" and "Toxin CDS annotation" workflows that can be used to generate the custom toxin database through the use of a venom tissue transcriptomic data. The transcriptome assembly performs two strategies to recover transcripts: (1) a genome-guided transcriptome assembly, which map reads using Hisat2 and retrieve transcripts using StringTie and Trinity; and (2) a *de novo* transcriptome assembly, which uses Trinity and rnaSPAdes to assemble transcripts. The "Toxin CDS annotation" step identifies full-length toxin CDSs in the assembled transcripts by performing BLAST search against a Toxin Database.

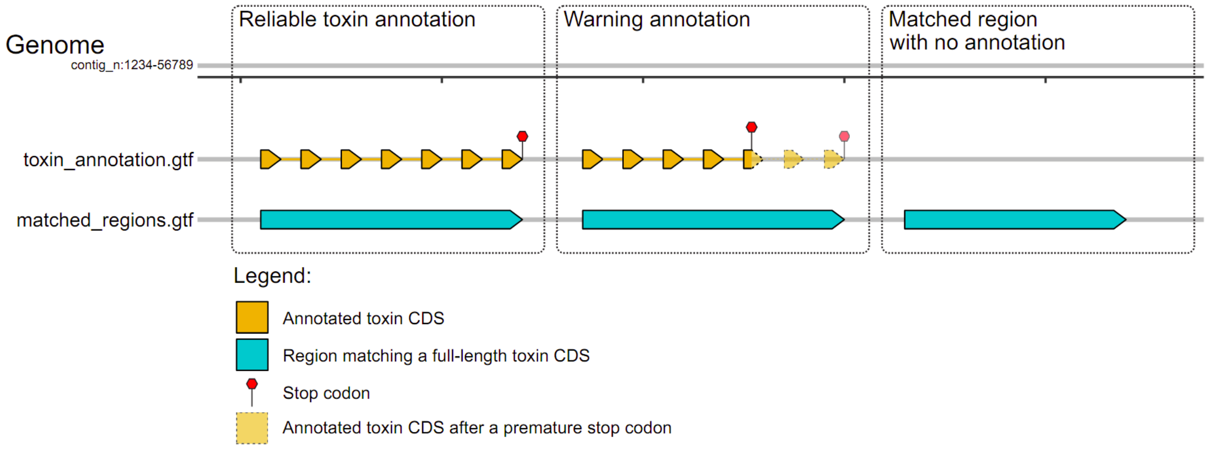

Figure S2. The annotations performed by ToxCodAn-Genome can be characterized into: “reliable toxin annotation”, “warning annotation”, and “matched region with no annotation”. The “reliable toxin annotation” represents an annotation identified in a genomic region containing a full-length toxin CDS and a confident gene structure. The “warning annotation” indicates an annotation in a genomic region containing a full-length toxin CDS with a confident gene structure but containing a premature stop codon, which may reveal a putative novelty, a truncated paralog toxin gene, a pseudogene, or an erroneous annotation. The annotations characterized as “warning annotation” must be manually inspected to confirm its status. The “matched region with no annotation” represents a genomic region matching a full-length toxin CDS but the refinement of the exon/intron boundaries step does not return a toxin annotation. The region marked as “matched region with no annotation” can be manually inspected to confirm if it has or not a toxin gene.

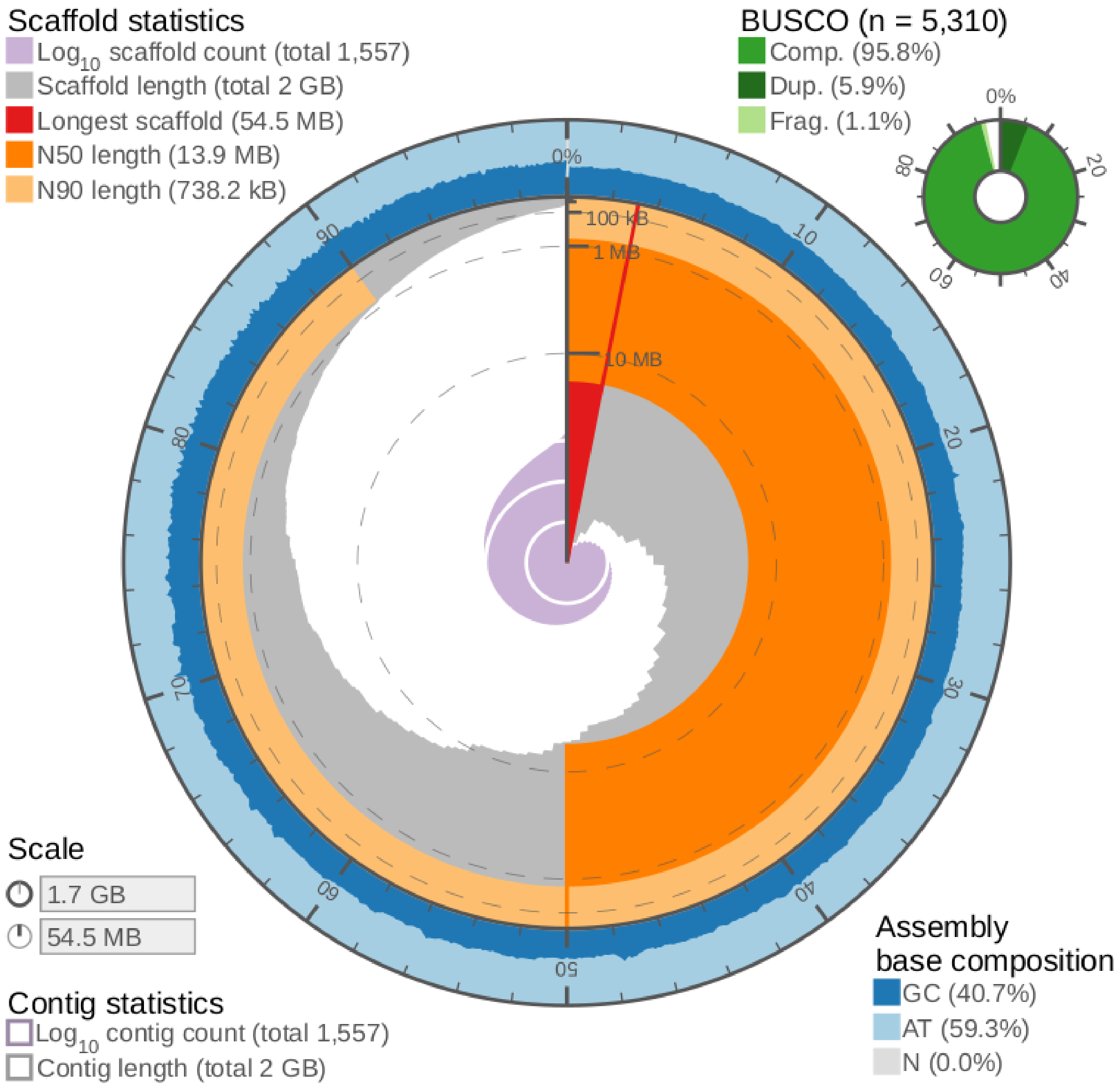

Figure S3. Basic assembly statistics of the *Bothrops alternatus* genome and BUSCO completeness using the tetrapoda gene set (odb10; total of 5310 genes).

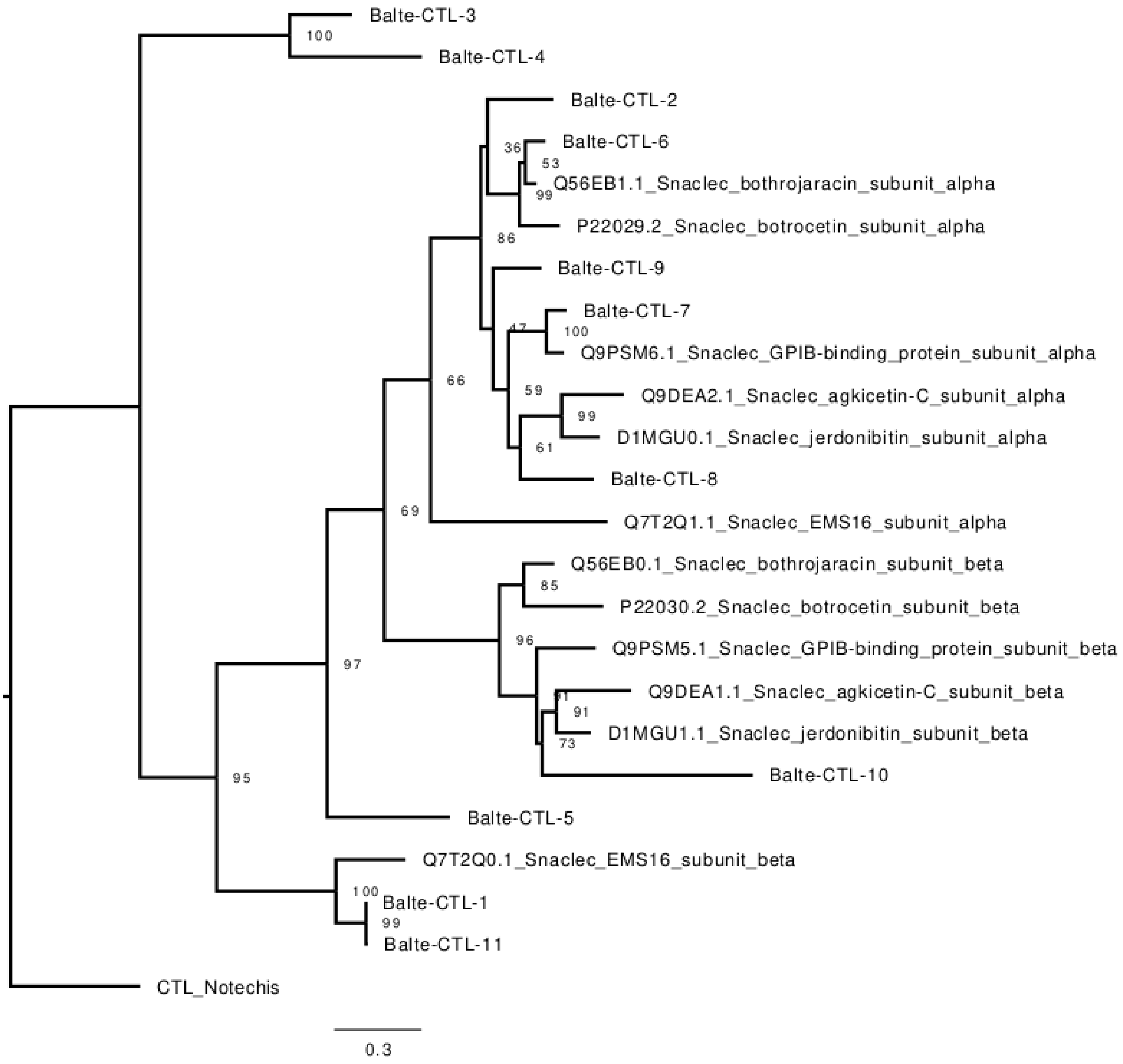

Figure S4. CTL phylogeny with known alpha and beta chain CTL homologs of several snake species. The support values of bootstrap are given in tree branches.
