# Supplementary material for: ToxCodAn-Genome: an automated pipeline for toxin-gene annotation in genome assembly of venomous lineages
Source: Gigascience. 2024 Jan 18;13:giad116. doi: 10.1093/gigascience/giad116 (PMC10797961; doi:10.1093/gigascience/giad116)
Supplement: giad116_Supplemental_Files [file giad116_supplemental_files.zip › Supplementary_file_2.pdf]

pedronachtigall / ToxCodAn-Genome Public

Code Issues Pull requests Actions Projects Security ...

main ToxCodAn-Genome / Guide /

pedronachtigall Update README.md ... yesterday History

..

example_data 5 months ago

figures 2 months ago

README.md yesterday

README.md

The guide to annotate toxin genes in genomes

Pedro G. Nachtigall

Summary

- Introduction
- The guide to toxin annotation
  - ToxCodAn-Genome
    - Toxin database
    - Custom toxin database
      - Curated toxin CDS set

            - Venom tissue transcriptome
        - Running ToxCodAn-Genome
    - Checking annotations
        - Checking toxin annotations
        - Checking warning annotations
        - Checking matched regions with no annotation
- NonToxin annotation
- Extra: Estimating expression level
- Extra: Plotting toxin loci
- Extra: NCBI submission alias

Introduction

The Guide to performing toxin gene annotation in genomes is part of **ToxCodAn-Genome** and designed to walk you through our toxin annotation pipeline.

Before start and walking through the guide, it will be good to have some basic knowledge about bioinformatics.

If you don't have much experience in using command lines, running programs, or bioinformatics in general, then you can follow the "Basic Bioinformatics" section in the ToxCodAn's guide to venom gland transcriptomes [Nachtigall et al., 2021]. This section has some general resources and information that may help you to better understand bioinformatics and the rest of this Guide. The Guide is not designed to teach you *everything*, so we highly recommend working through the training resources first!

The guide to toxin annotation

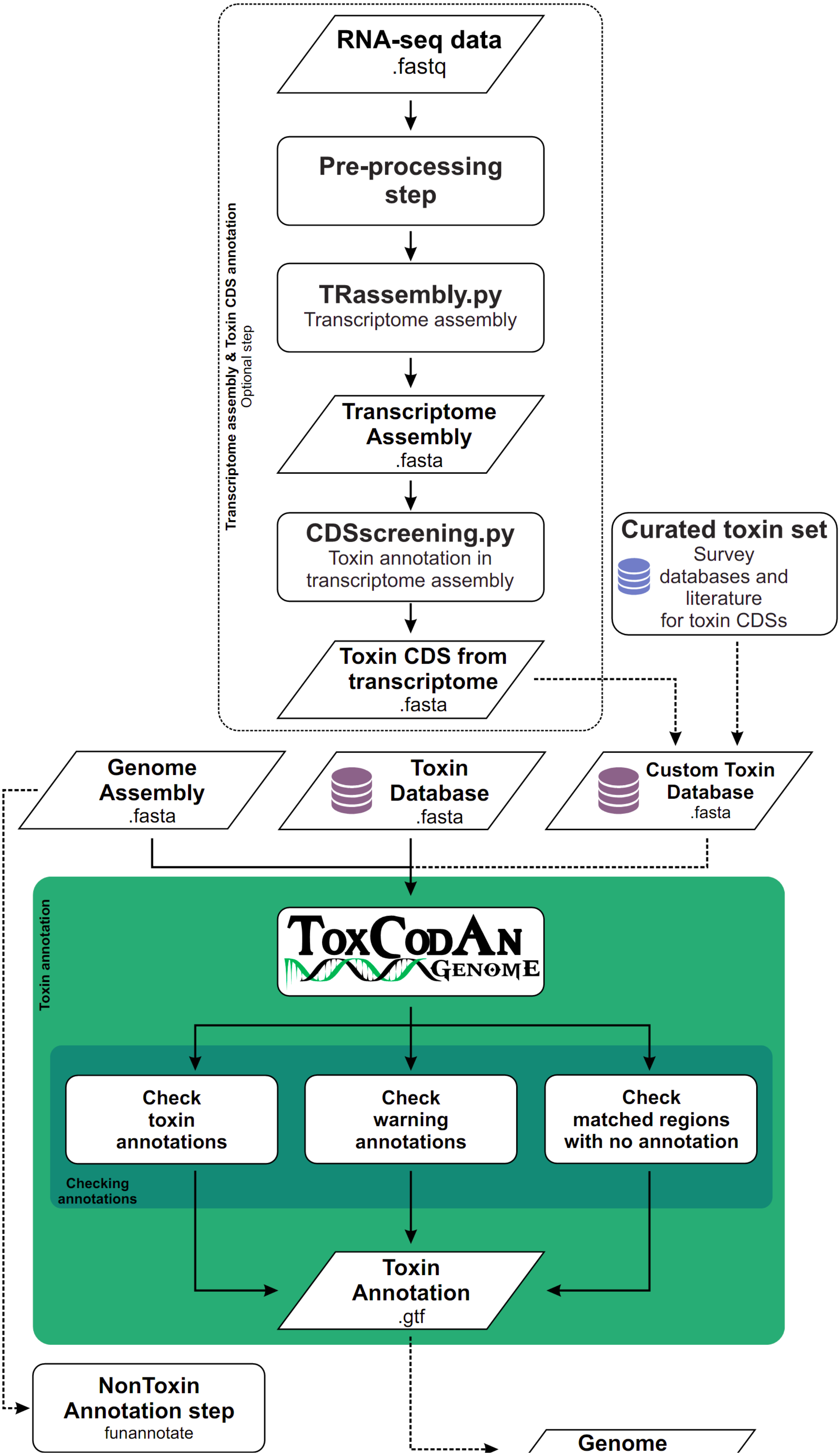

Transcriptome assembly & Toxin CDS annotation
Optional step
RNA-seq data
.fastq
Pre-processing step
TRassembly.py
Transcriptome assembly
Transcriptome Assembly
.fasta
CDSscreening.py
Toxin annotation in transcriptome assembly
Toxin CDS from transcriptome
.fasta
Curated toxin set
Survey databases and literature for toxin CDSs
Genome Assembly
.fasta
Toxin Database
.fasta
Custom Toxin Database
.fasta
Toxin annotation
ToxCodAn Genome
Check toxin annotations
Check warning annotations
Check matched regions with no annotation
Checking annotations
Toxin Annotation
.gtf
NonToxin Annotation step
funannotate
Genome

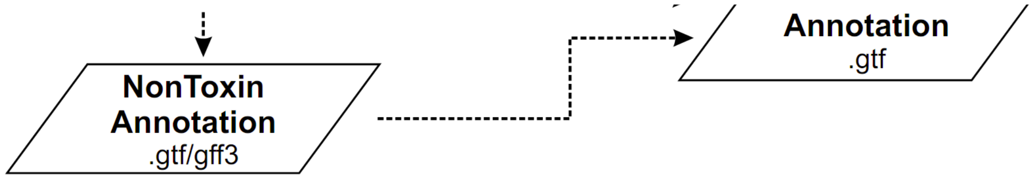

ToxCodAn-Genome

ToxCodAn-Genome was designed to identify and annotate toxins in genomes of venomous lineages. It consists of a step to detect putative toxin loci among the genome using a comprehensive toxin database, followed by a step to select bonafide toxin loci that are used to build gene models specific to each toxin loci and generate the toxin annotation file.

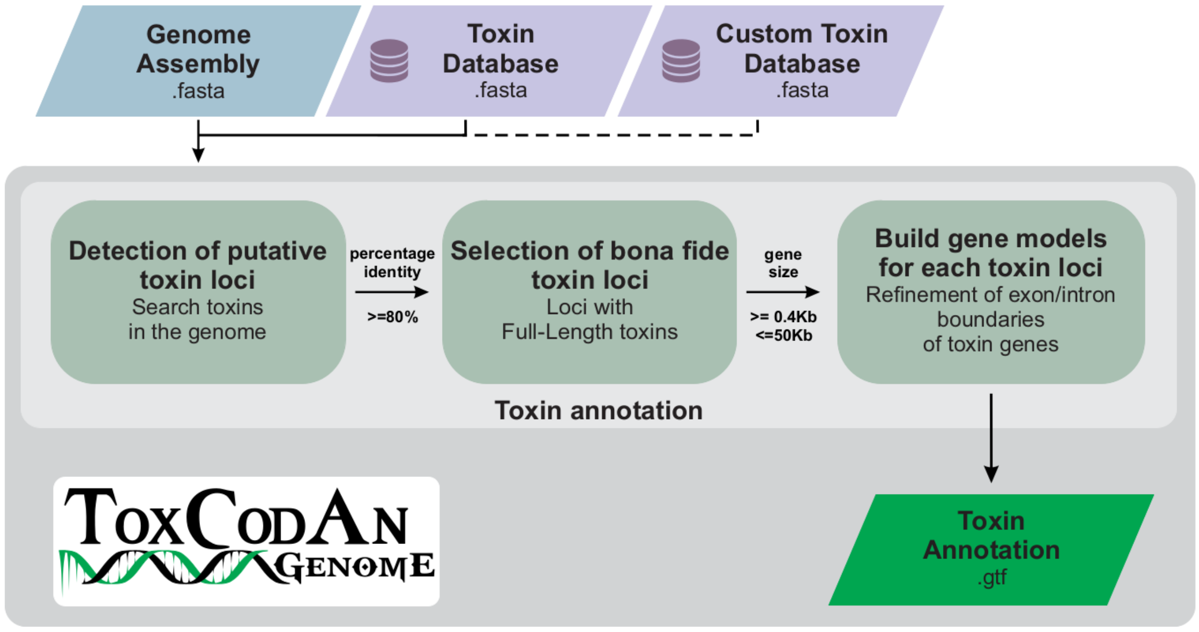

Toxin database

We have designed a toxin database with curated toxin CDSs for some venomous lineages, which can be downloaded here.

The toxin database must be specified with the `-d` parameter (e.g., `-d toxin_database.fasta`).

If you are working with some venomous lineage that is still not available in our toxin database list, you can design a custom toxin database as described below.

Custom toxin database

If you and your research group have been extensively working within the venomous taxa of the species being analyzed and have a well-curated set of toxin CDSs available in FASTA format, it can be used as the main toxin database or it can be integrate to some of the pre-built toxin databases.

If using a custom toxin database as the only toxin database, just set the path to the custom database file with the parameter `-d` . In this case, ensure you have the toxin family annotated in the header of each toxin CDS after an `_` ("underscore") symbol (e.g., `>Sequence1_TOXIN`).

- ***Tip:*** If your database only contains one toxin family, it can be easily done with Perl:
  - `perl -p -e 's/^(>.*)$/$1_TOXIN/g' custom_toxin_db.fasta > custom_toxin_db_renamed.fasta`
  - Replace `"_TOXIN"` to the target toxin family (e.g., for snake venom metalloproteinase use `"_SVMP"` and so on).

If you want to integrate some of the ToxCodAn-Genome's toxin database, just indicate the path to the custom toxin database file with the parameter `-C` (e.g., it must something like `-d toxin_database.fasta -C custom_toxin_db.fasta`). In this case, you will not need to specify the toxin family in the header (as previously described); however, if it is not specified, the ToxCodAn-Genome will consider the sequence as a "generic" toxin, by adding a string `_TOXIN` at the end of header of each sequence (e.g., `>Sequence1_TOXIN`). We strongly recommend to perform the annotation and add the toxin family in each sequence header to facilitate downstream analysis.

If you don't have a set of curated toxin CDS to use as the main toxin database or as a custom toxin database, you can follow any or both strategies to survey for "curated toxin CDSs" in scientific databases and literature and to survey for toxin CDSs from "venom tissue transcriptome".

Curated toxin CDS set

To build a toxin database with a set of curated toxin CDSs, you may survey available data in sequence databases, such as TSA and Genbank from NCBI, EMBL-EBI, ENSEMBL, CNGBdb (China National GeneBank DataBase), and repositories and databases designed to specific genomes/purposes. You can take advantage of the well-designed search engine of these databases by using "keywords" that may help you to retrieve a high-quality set of toxin CDSs.

Here, we briefly described how to search for complete CDSs of the toxin SVMP (snake venom metalloproteinase) of vipers available in NCBI as an example, but you can easily modify the search parameters and use other databases to design a specific set of sequences for your purposes.

- Access the GenBank website: https://www.ncbi.nlm.nih.gov/genbank/
- Use the following set of keywords in the: `Viperidae AND snake venom metalloproteinase AND "complete cds"`

- Download the result in GenBank format:
  - "send to:" -> "Complete Record" -> "File" -> "Format: GenBank" -> "Creat File"
  - You can download the data in any other format, like downloading the "coding sequence" in FASTA format. But you may adjust the header of sequences accordingly (see "Custom toxin database" above).
- Run our script to retrieve the toxin CDSs and have it ready to use with ToxCodAn-Genome:
  - `ParseGenBank.py inut.gb output_cds.fasta TOXIN`
  - Replace `"TOXIN"` to the target toxin family (e.g., `"SVMP"` for snake venom metalloproteinase).

You can also search for RNA-seq data from venom tissue to be re-analyzed. These datasets can be downloaded, the toxin transcripts assembled and its toxin CDSs annotated and retrieved to be used as a Toxin Database. You can follow any available pipeline, ToxCodAn's guide to venom gland transcriptomes, or the "Venom tissue transcriptome" section below.

Additionally, you can also survey for accession numbers and links to the datasets of toxin CDSs in published manuscripts (in the "data availability" sections) or within its supplementary files (some authors usually keep its curated sequences in supplementary files or deposited in other databases rather than NCBI's GenBank/TSA, such as figshare and dryad). You can also contact authors to request such toxin CDSs when not available in any specific database and/or supplementary file.

- ***Tip:*** To better curate a toxin set and ensure that you have designed a high-quality set, it is good to acquire knowledge about the toxins, their protein domains, and also about the venomous lineage being studied.
  - You can find details about venomous lineages and their toxins in VenomZone, which is a good resource to retrieve information on venoms from six taxa (snakes, scorpions, spiders, cone snails, sea anemones, and insects), as well as on their biological targets and effects. It also has a description of the protein domains of each toxin family and links to retrieve its peptide sequences from ToxProt/Uniprot.
  - You can also use the protein id from specific toxins to access its information in Uniprot and better understand the structure of domains of these toxins (e.g., https://legacy.uniprot.org/uniprot/Q9W6M5).
  - To identify protein domains and signal peptide in the identified toxins and check if it presents the expected toxin structure, you can use some web-servers like HMMER, InterProScan, and SignalP.
  - You can also survey the literature to find reviews about specific toxin

families and venomous lineages.

Venom tissue transcriptome

If you have venom tissue transcriptomic data available for the species being annotated, you should consider using this data in the toxin annotation step to improve the final set generated by ToxCodAn-Genome. In this sense, you can detect the toxin CDSs and annotate them by following your own pipeline, or follow the guide to venom gland transcriptomics available within ToxCodAn tool and published in Briefings in Bioinformatics [Nachtigall et al., 2021].

If you and your research group do not have a strong background in performing such transcriptome annotation, we designed two scripts to help on this task that can be run separately or integrated into the main ToxCodAn-Genome pipeline.

▾ Expand "Transcriptome Assembly" Section

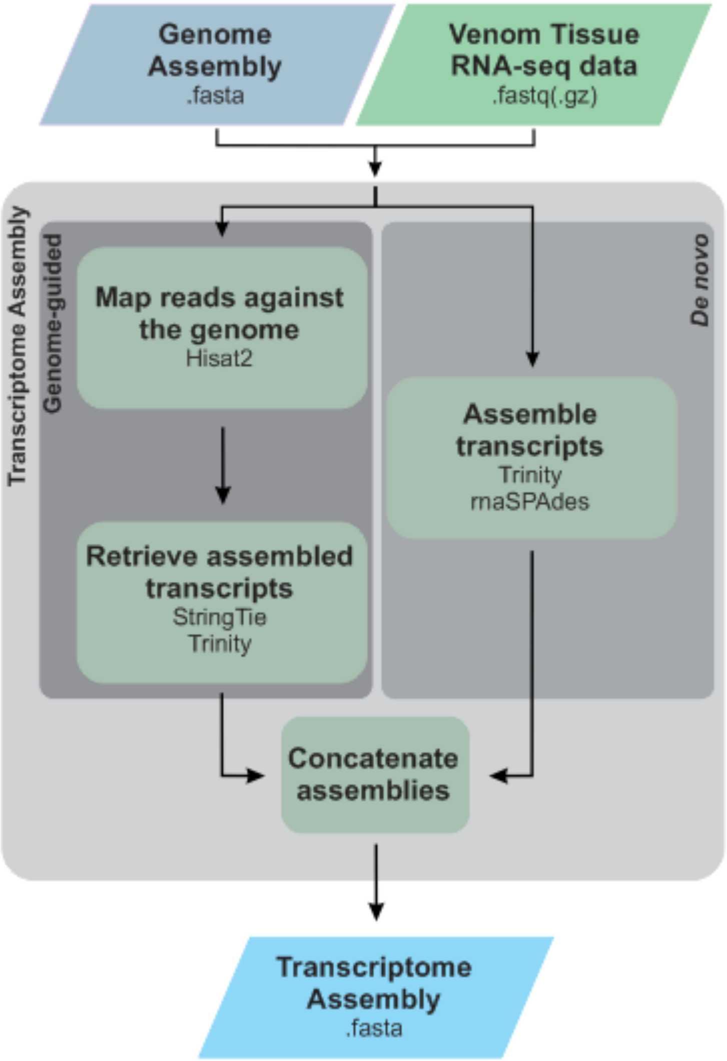

Pre-processing of reads

First, ensure that the adapters are trimmed and low-quality reads filtered. It can be performed by using any available tool, such as trim_galore!, fastp, and many others. Here, we just set a simple command to run `trim_galore`:

- `trim_galore --paired --phred33 --length 75 -q 25 --stringency 1 -e 0.1 -o sample_trimmed sample_r1.fastq.gz sample_r2.fastq.gz`

After removing adapters and low-quality reads, you can move to the transcriptome assembly step.

Transcriptome assembly

We designed a script to perform transcriptome assembly using the genome-guided mode of Trinity and StringTie and the *de novo* mode of Trinity and rnaSPAdes. We use more than one tool to assembly transcripts to ensure the recovery of most toxin transcripts from the dataset [Holding et al., 2018].

If using paired-end reads:

```
TRassembly.py -g genome.fasta -r
sample_r1.fastq.gz,sample_r2.fastq.gz -c 20 -M 20G
```

If using single-end reads (or merged reads):

```
TRassembly.py -g genome.fasta -r sample_reads.fastq.gz -c 20 -M 20G
```

- The final transcriptome assembly can be found at `assembly/transcripts.fasta`. The output directory can be changed by using the parameter `-o`.
- Please adjust the number of threads `-c` and memory usage `-M` accordingly to your system.
- Run `TRassembly.py -h` to print the help message.
- `TRassembly.py` handles uncompressed and compressed files (i.e., `.fastq` and `.fastq.gz`).
- **It may take a while to finish!**

If you are using venom tissue transcriptomic data from a closely related species you may consider using its genome as a reference (and not the genome of the target species you are analyzing). However, if it has no genome available, you can only run the *de novo* method from `TRassembly.py`. It can be set with the parameter `-m`. By default, it runs both assembly methods (`-m both`), but you can set it to run only the *de novo* strategy (`-m dn`) or only the genome-guided strategy (`-m gg`).

You may also run each assembler and method separately and also consider using other tools (e.g., extender, NGEN, velvet, etc) to increase the probability of retrieving most of the toxin transcripts. We strongly recommend you to test several assemblers and paremeters to ensure a high-quality toxin transcripts assembly, because it was not thoroughly tested to all venomous lineage and it is a step that still needs improvement. You can follow the instructions to run other assemblers here.

- ***Tip about transcriptome assembly:*** if using a paired-end read dataset, you may consider merging reads to improve the *de novo* assembly by using PEAR. It will increase the size of the reads by merging pairs with high-quality overlap.
    - `pear -k -j 20 -f sample_r1.fastq.gz -r sample_r1.fastq.gz -o sample_merged_reads`
    - `-j` is the number of threads, adjust accordingly.
- ***Tip about Trinity:*** if using a paired-end read dataset, ensure the number of reads in both files match. If the number of reads does not match, Trinity may return an error and stop the assembling step. To avoid this issue, you can merge reads as described above or you can take advantage of seqtk to subsample your dataset to a specific number of reads in both files.
    - `seqtk sample -s100 sample_r1.fastq 5000000 > sample_sub1.fastq`
    - `seqtk sample -s100 sample_r2.fastq 5000000 > sample_sub2.fastq`
    - Always use the same random seed to keep a proper pair in both files (parameters `-s`, which is set to `100` in our example).
    - Here, we set the number of reads to 5 million (`5000000`), but it must be adjusted accordingly to your dataset.

▼ Expand "Toxin CDS annotation" Section

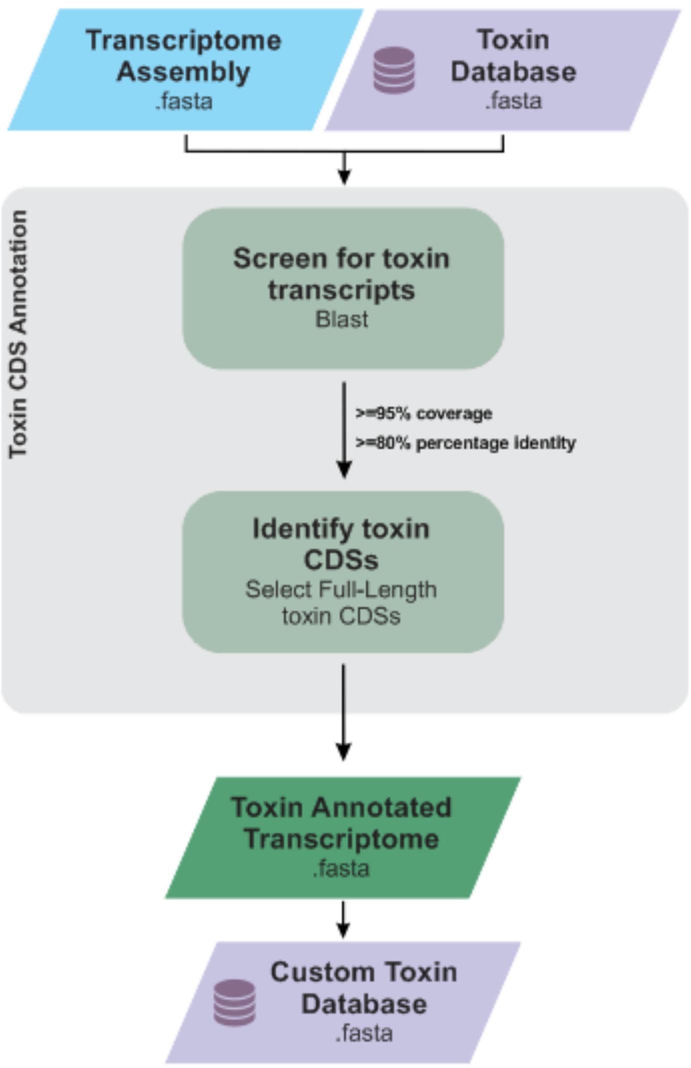

Toxin CDS annotation

We designed a script to screen a transcriptome assembly (assembled by our script `TRassembly.py` or any other pipeline) using any specific set of toxin CDS sequences. To identify toxin CDSs in the transcripome assembly, you can set one of the available Toxin databases or use any set of sequences with curated toxin CDSs designed by you.

The script `CDSscreening.py` can be easily run as follows:

```
CDSscreening.py -t transcripts.fasta -d CDS_database.fasta -c 20
```

- The toxin CDS output can be found at `screening/cds_screening.fasta`.
  - The output directory can be changed by using the parameter `-o`.
- Set the path to your transcriptome assembly accordingly.
  - e.g., if using the output from `TRassembly.py` just set `-t assembly/transcripts.fasta`.
- Set the path to your toxin CDS database accordingly (e.g., `$PATH/to`

`/Viperidae_db.fasta`).

- Adjust the minimum percent identity between CDS in the database and transcript using parameter `-p` (default = `-p 80`), if needed.
- Adjust the minimum size of CDSs using the parameter `-l` (default = `-l 200`), if needed.
- Adjust the number of threads `-c` accordingly to your system.
- Run `CDSscreening.py -h` to print the help message.

If you feel that some toxins are not being properly annotated by `CDSscreening.py` pipeline or wants to ensure that all toxins are being correctly screened, you can consider running ToxCodAn and follow its guide to venom gland transcriptomics to perform a manual curation of the toxins present in the transcriptome being analyzed. You may also consider running other annotation tools, like Venomix, Trinotate, and Dammit, followed by a manual inspection of annotated CDSs.

▾ Expand "Surveying RNA-seq databases" Section

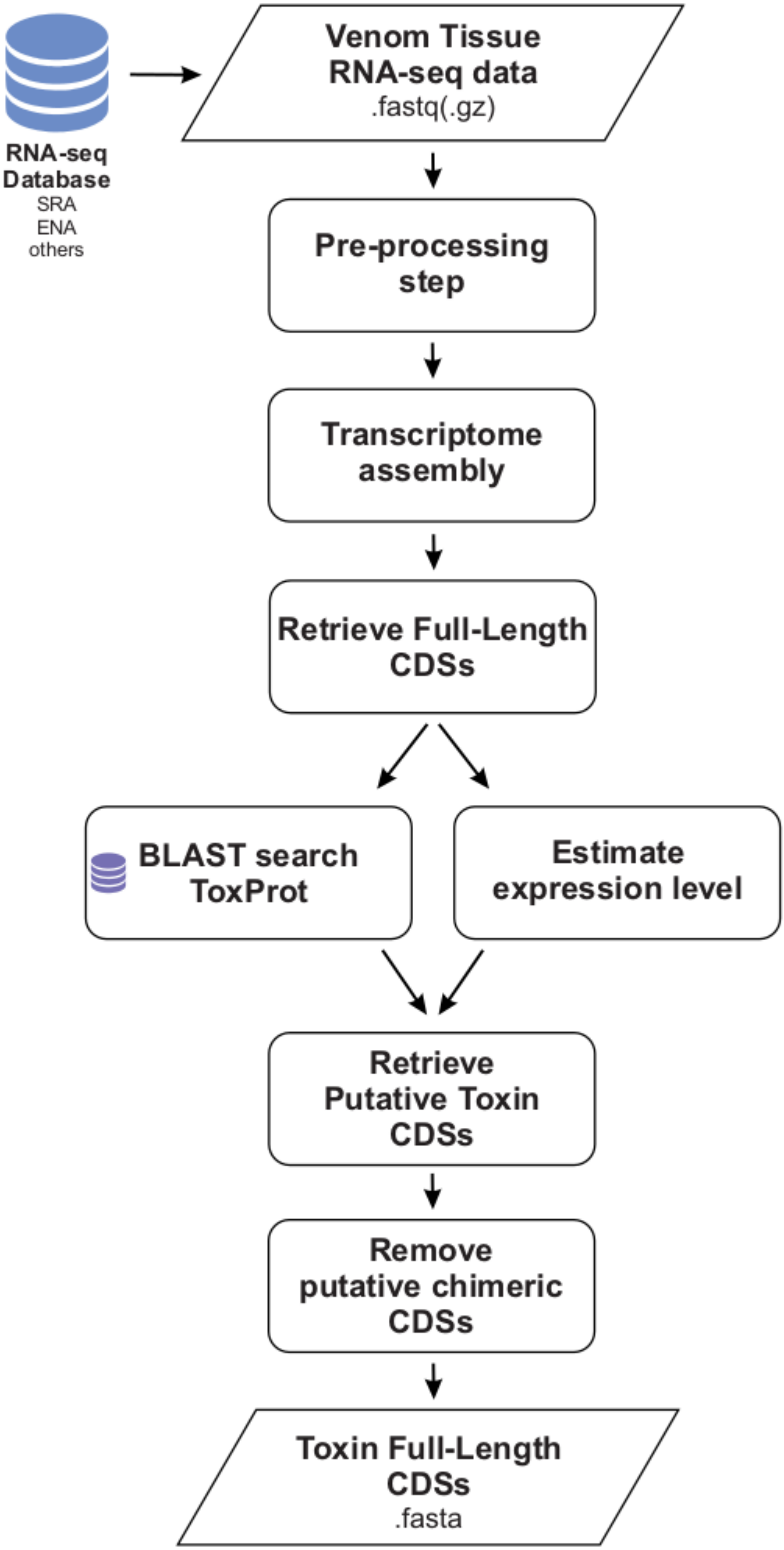
RNA-seq Database
SRA
ENA
others
Venom Tissue RNA-seq data
.fastq(.gz)
Pre-processing step
Transcriptome assembly
Retrieve Full-Length CDSs
BLAST search ToxProt
Estimate expression level
Retrieve Putative Toxin CDSs
Remove putative chimeric CDSs
Toxin Full-Length CDSs
.fasta

Surveying RNA-seq databases

To take advantage of databases containing venom tissue RNA-seq data available for use, like SRA from NCBI, ENA from EMBL, and many others. We designed a pipeline to survey RNA-seq databases for venom tissue transcriptomic data from any source to generate a custom toxin database for the venomous lineage being analyzed. Briefly, it consists in downloading the RNA-seq data, pre-processing the data, performing *de novo* assembly (using several tools), getting all full-length CDSs, estimating expression level (to filter out lowly expressed CDSs), performing similarity search against the ToxProt, removing putative chimeric transcripts/CDSs based on read coverage.

⚠️ ***Note:*** It is an experimental pipeline and no warranties to recover confident toxins CDSs are given. Use this pipeline with caution and consider curating the final toxin set.

Downloading RNA-seq data

First, you need to find the dataset(s) you want to download and perform the analysis. You can search for datasets using specific keywords, like `venom` , `transcriptome` , and the target species/clade (e.g., `Viperidae` , `Bothrops` , and/or `Bothrops alternatus`). You can also find the accession numbers of datasets within publications in their "Data availability" sections and/or in Supplementary files.

After choosing one or more datasets, you can download them and start this pipeline.

Here, we show how to easily download the RNA-seq data from SRA using the `fastq-dump` tool, which is part of the SRA toolkit. But you can use any other method and database.

```
fastq-dump --split-3 --origfmt --readids --gzip SRRxxxxxx
```

- You can use the parameter `--outdir` to specify a folder to save the files (e.g., `--outdir /path/to/outdir/`)
- Adjust `SRRxxxxxx` accordingly.
- The paired-end reads of the downloaded dataset will be saved as `SRRxxxxxx_1.fastq.gz` and `SRRxxxxxx_2.fastq.gz` .

Pre-processing of reads

Trim adapters and filter low-quality reads using trim_galore! (or another tool).

```
trim_galore --paired --phred33 --length 75 -q 25 --stringency 1 -e
0.1 -o SRRxxxxxx_trimmed SRRxxxxxx_1.fastq.gz SRRxxxxxx_2.fastq.gz
```

- It will output the trimmed reads into a folder named `SRRxxxxxx_trimmed`.
- Use the files `SRRxxxxxx_trimmed/SRRxxxxxx_1_val_1.fq.gz` and `SRRxxxxxx_trimmed/SRRxxxxxx_2_val_2.fq.gz`.

Merge trimmed paired-end reads using PEAR. It will increase the size of the reads by merging pairs with high-quality overlap.

```
pear -k -j 10 -f SRRxxxxxx_trimmed/SRRxxxxxx_1_val_1.fq.gz -r
SRRxxxxxx_trimmed/SRRxxxxxx_2_val_2.fq.gz -o SRRxxxxxx_merged_reads
```

- Adjust `-j` (number of threads) accordingly to your system.

De novo transcriptome assembly

Here, we consider that you are using transcriptomic data from species with no genome available and the intructions are related to *de novo* assembly. You must consider to use three or more assemblers to retrieve most of toxin transcripts in the dataset[Holding et al., 2018]. In this guide, we describe the commands to run Trinity and StringTie, rnaSPAdes, and Bridger; however, you can also use other tools, such as extender, NGEN, velvet, to increase the probability of assembling all toxin transcripts.

```
rnaspades.py --threads 10 --phred-offset 33 -1 SRRxxxxxx_trimmed/SRRxxxx

#add paired-data information into headers of reads to run Trinity assemb
zcat SRRxxxxxx_tg/SRRxxxxxx_1_val_1.fq.gz | awk '{ if (NR%4==1) { print
zcat SRRxxxxxx_tg/SRRxxxxxx_2_val_2.fq.gz | awk '{ if (NR%4==1) { print

Trinity --seqType fq --max_memory 16G --CPU 10 --full_cleanup --left SRR

#remove renamed reads
rm *_renamed.fastq

Bridger.pl --CPU 10 --kmer_length 30 --seqType fq --single SRRxxxxxx_mer

#concatenate assemblies
cat SRRxxxxxx_spades/transcripts.fasta SRRxxxxxx_trinity.Trinity.fasta b
```

- Adjust `--CPU/--threads` and `--max_memory` parameters accoridngly to your system.

Retrieve all full-length CDSs

Retrieve all full-length CDSs present in the assembled transcripts using orfipy. Then, remove redundancy by clustering 100% identical full-length CDSs using our script `RemoveRedundancy.py` and translate CDSs into peptides to perform a similarity search.

```
orfipy SRRxxxxxx_all_assemblies.fasta --dna orfs.fa --include-stop
--start ATG --min 200 --max 8000 --procs 20 --table 1 --outdir
orfs_out
RemoveRedundancy.py orfs_out/orfs.fa orfs_RR.fa orfs_RR_report.txt
translate_frame_1.py orfs_RR.fa orfs_RR_pep.fasta
```

BLAST search the ToxProt

To annotate the CDSs and identify putative toxins, we BLAST search the full-length CDSs against the ToxProt database. Here, you can only use the ToxProt sequences of the lineage being studied (e.g., if studying a snake species, you can download only toxin sequences available for vertebrates or snakes).

```
makeblastdb -in toxprot.fasta -out blastDB/TOXINS -dbtype prot
blastp -query orfs_RR_pep.fasta -db blastDB/TOXINS -out
orfs_RR_pep_blast.out -num_threads 20 -max_target_seqs 1 -outfmt '6
qseqid qlen sseqid slen pident length mismatch qstart qend sstart
send evalue bitscore'
```

- Adjust `-num_threads` accordingly to your system.
- Adjust the path to the `toxprot.fasta` accordingly.

Estimate expression level

Estimate the expression level of CDSs using RSEM (with Bowtie2).

```
rsem-prepare-reference --bowtie2 orfs_RR.fa orfs_RR_ref
rsem-calculate-expression -p 20 --bowtie2 --bowtie2-mismatch-rate
0.05 SRRxxxxxx_merged_reads.assembled.fastq(.gz) orfs_RR_ref rsem
```

- Adjust `-p` (number of threads) accordingly to your system.
- Check the "Estimating expression level" section for further details about the `--bowtie2-mismatch-rate` parameter.

Retrieve putative toxins

Use the script `ParseBlastRsem.py` to parse the BLAST and RSEM outputs. It uses the RSEM output to only keep CDSs with FPKM > 1.0 (to ensure it is expressed in the dataset analyzed) and keep CDSs with "good match" to the -ToxProt sequences used (i.e., keep CDSs with percent_identity >= 40%, query_coverage >= 40%, subject_coverage >= 40%, and score >= 30). It also uses the file `uniref_50.txt` (which is provided in the "ToxCodAn-Genome/misc/" folder) to rename the toxin family based on the BLAST hit. Then, you can cluster 99% similar putative toxin CDSs using CD-HIT to reduce redundancy for the next step.

```
ParseBlastRsem.py orfs_RR.fa orfs_RR_pep_blast.out
rsem.isoforms.results ToxCodAn-Genome/misc/uniref_50.txt SRRxxxxxx
cd-hit-est -c 0.99 -i SRRxxxxxx_toxins_cds.fasta -o
SRRxxxxxx_toxins_cds_ch99.fasta
```

Remove putative chimeric CDSs

Use the script `CoverageCheck.py`, which is integrated into ToxCodAn tool. See the "CoverageCheck" section in ToxCodAn's guide to venom gland transcriptomics for further details about this script.

- `CoverageCheck.py` requires Python (biopython, pandas, and dfply), BWA, Samtools, Bedtools, Bowtie2, and RSEM.

```
CoverageCheck.py -i SRRxxxxxx_toxins_cds_ch99.fasta -r
SRRxxxxxx_merged_reads.assembled.fastq(.gz) -m 0.05 -c 2 -t 20
```

- Adjust `-t` accordingly to your system.
- Use the `SRRxxxxxx_toxins_cds_ch99_FILTERED.fasta` as the final toxin set.
    - However, we strongly recommend to inspect this final toxin set to remove any spurious sequence that may still pass through the pipeline.

You can repeat this pipeline to any other dataset available to the venomous lineage you are analyzing and concatenate their toxin sets to generate the final custom toxin database.

- ***Tip:*** If using more than one dataset from the same species, you can consider to concatenate both final toxin sets and cluster 98% similar toxin CDSs using CD-HIT to generate a final species toxin set before concatenating it with datasets from other species.
    - `cat SRRxxx1_toxins_cds_ch99_FILTERED.fasta SRRxxx2_toxins_cds_ch99_FILTERED.fasta > species_toxins_cds.fasta`
    - `cd-hit-est -c 0.98 -i species_toxins_cds.fasta -o`

```
species_toxins_cds_ch98.fasta
```

Now that you ensure that you have a well-designed toxin database and, if available, a well-curated toxin CDSs from the venom tissue transcriptome, you are ready to run ToxCodAn-Genome and perform the toxin annotation.

Running ToxCodAn-Genome

ToxCodAn-Genome only requires the `genome.fasta` and the `toxin_database.fasta` files as mandatory. You can run it as follows:

```
toxcodan-genome.py -g genome.fasta -d toxin_database.fasta -c 6
```

- Set the path to the `genome.fasta` accordingly.
- Set the path to the `toxin_database.fasta` accordingly.
- Adjust the number of threads `-c` accordingly to your system.
- Adjust the output directory using the `-o` parameter.
  - By default, the outputs will be save in a folder named "ToxCodAnGenome_output" in the working directory.
- If you have designed a custom toxin database with curated toxin CDSs (retrieved from databases, literature, and/or venom tissue transcriptome), you can set it to be integrated into the toxin database using the parameter `-C path/to/custom_toxin_db.fasta`. See the "Curated toxin CDS set" section for more details.
- If you have a set of transcripts assembled from venom tissue RNA-seq data, you can also set it to be used directly into ToxCodAn-Genome command line through the parameter `-t path/to/transcripts.fasta`. However, you must specify a toxin database in the parameter `-d`, once the toxin CDSs are retrieved from the transcriptome using the toxin database. See the "Venom tissue transcriptome" section for more details about this process.
-

If you have the venom tissue transcriptome reads (in FASTQ or FASTQ.GZ format), you can set it to be assembled and analyzed directly into ToxCodAn-Genome command line through the parameter `-r`. However, you must specify a toxin database in the parameter `-d`, once the toxin CDSs are retrieved from the transcriptome using the toxin database. See the "Venom tissue transcriptome" section for more details about this process.

  - If single-end (or merged reads), specify only one file (e.g., `-r path/to/reads.fastq`).
  - If paired-end, specify both files in a comma-separated format (e.g., `-r path/to/reads_1.fastq,path/to/reads_2.fastq`).
  - Ensure the reads have the adapters trimmed, once this step is not performed by ToxCodAn-Genome.

- You can compare the annotated toxins to the UniProt/ToxProt database using the parameter `-u path/to/uniprot.fasta`. It will BLAST search the annotated toxin CDSs against the provided UniProt/ToxProt database file in FASTA format and it will output a report in TXT format. The output will contain the annotated toxin and the best hit in the UniProt/ToxProt database, alongside their percent identity, alignment length, toxin length, and uniprot/toxprot entry length. This report file may help to check the annotated toxins as described in the "Checking annotations" section.

Note: ToxCodAn-Genome has several parameters that can be tested and used in your analysis (run `toxcodan-genome.py -h` to print all of them).

Tip: If the genomic context of toxins in your target species is not well known, you can consider running ToxCodAn-Genome and testing some distinct threshold values for some parameters. It may help you ensure that you are retrieving a high-quality toxin annotation.

- You can test several values in the "percent identity" parameter (which is 80% by default; `-p 80`).

  - You can also run ToxCodAn-Genome using 70% (`-p 70`) and 90% (`-p 90`) to increase and decrease the number of matching regions between the toxin CDSs in the database and the genome.
  - By using high values (e.g., 90%), you will detect a lower number of toxin genes with most of them representing confident toxin annotations, but you may lose some genomic regions containing putative novelties and highly divergent toxins.
  - By using low values (e.g., 70%), you will detect a higher number of toxin

genes, which helps in the detection of putative novelties and highly divergent toxins. However, it may also generate false-positive annotations.

- You can test several values in both "gene size" parameters (which are 400 nts and 50000 nts by default; `-s 400 -S 50000`).
  - Toxin families widely vary in gene size, which may range from shorter than 2000 nts to longer than 50000 nts.
  - For instance, the toxins annotated in the *Bothrops jararaca* genome were shown to vary its gene size from 1.7Kb (1700 nts) to 40.6Kb (40600 nts) [Almeida et al., 2021].
  - The size distribution of toxin CDSs may help you have an approximate idea of what is the best gene size threshold to use. However, you can consider running it specifically for each toxin family. It may help you to improve the final toxin annotation set.
  - Bear in mind that this parameter is only related to the CDS size in the genome, it does not account for the exons, which also consider the UTRs.

Checking annotations

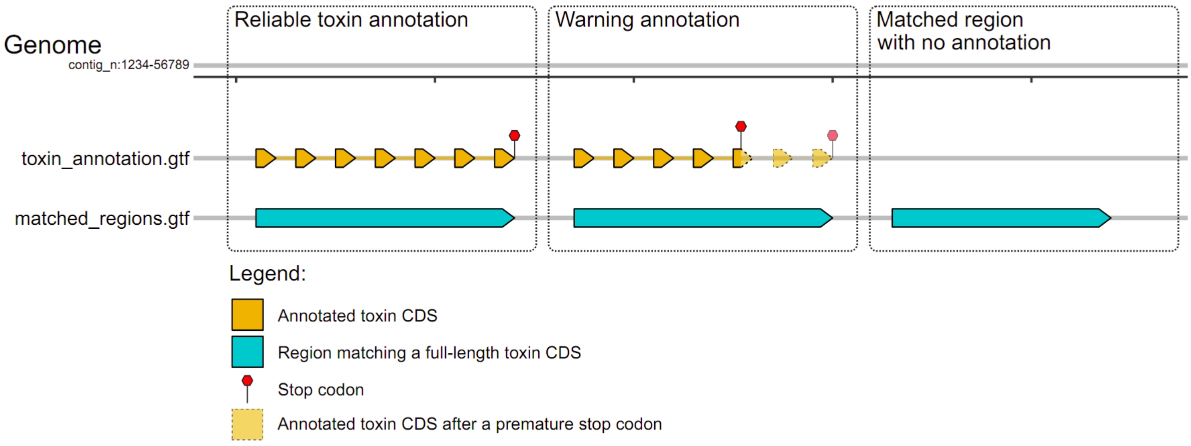

Here, we describe how to inspect the annotations generated by ToxCodAn-Genome and confirm its status or adjust its annotation accordingly to reach a well-curated toxin repertoire for the genome being analyzed.

Despite most of the ToxCodAn-Genome's annotated toxins representing reliable annotations, it is always good to double-check the annotated toxins (mainly the ones in the `annotation_warning.txt` file) and remove/correct any spurious annotation. In this process, you may also retrieve some regions where ToxCodAn-Genome may detect a matching full-length toxin but is not able to properly identify the exon/intron boundaries.

Checking the toxin annotations will certainly improve the quality of your research and findings and it will help to not propagate any spurious annotations by allowing you to remove/correct any erroneous annotation.

Checking toxin annotations

To check the toxins annotations, you can compare the annotated CDSs (and/or the peptide sequences) with previous available transcriptomic data from the same species and/or closely related species. For instance, when analyzing *Bothrops alternatus* genome, we confirmed most of the toxin annotations output by ToxCodAn-Genome by comparing it against the toxins sequences available in previous transcriptomic studies of *Bothrops alternatus* and its closely related species (i.e., *Bothrops cotiara*,*Bothrops fonsecai*,*Bothrops jararacussu*, and *Bothrops jararaca*).

- Also, you can align the toxin annotations (using the CDSs and/or peptide sequences) of specific toxin families with other sequences available for this toxin family (using tools like, MAFFT, MUSCLE, CLUSTAL, etc). It may reveal some feature (e.g., regions of long gaps, sequences that are too long or too short at the ends) that helps you decide if the annotation is reliable or need carefull inspections.
    - Sequences that present a similar size to other known toxins and not present long gap regions, may represent a reliable toxin.
    - Sequences presenting long regions of gap and/or being too long or too short at the ends, may represent annotations that need inspections.
- You can predict the signal peptide and protein domains of the annotated toxins to confirm it has a well-characterized structure of the toxin family being analyzed by using the web-server and/or the stand-alone version of tools like HMMER, InterProScan, and SignalP.
    - if the annotated toxin presents all expected protein domains, it may represent a reliable annotation.
    - if the annotated toxin presents discrepancies in the protein domains, it may represent an annotation that need inspections.

Alternatively, you can also BLAST search the nucleotide and/protein databases of NCBI using the blastn, blastx, and blasp tools.

- You can check for specific features in the BLAST output:
    - `Description` and `Accession` -> may help you understand the hits and if your annotation represents a bonafide toxin.
    - `Per. Ident` and `Query Cover` -> may indicate if the hit represents a good homologous sequence in the database, which may help confirm if

the annotated toxin is reliable or need further inspections.
    - `Scientific Name` -> the species that the hit was generated from. It may help you understand the similarities observed and also if it is a hit from a closely related species (which may indicate that it is a true toxin when it is a venomous lineage) or if it is from a distantly related species (which may indicate that your annotation may represent an endogenous gene when it is not a venomous lineage)

- If using the CDS and running the blastn tool, you can also take advantage of the TSA database in the BLAST search.
    - Set the parameter `Database` in the `Choose Search Set` options to `Transcriptome Shotgun Assembly (TSA)`
    - You can also set it to a specific taxonomy clade to have faster results. Set the parameter `Limit by` to the target clade (e.g., if working with snakes, set it to `snakes (taxid:8570)`).

- You can also compare the annotated toxins to the UniProt/ToxProt database. You can use the BLAST tool in the UniProt web-server by inputting the protein sequence of the annotated toxin (i.e., `toxin_annotation_pep.fasta`). Alternatively, you can download the database in FASTA format and use the built-in function in the ToxCodAn-Genome command line using the parameter `-u path/to/uniprot.fasta` (see "Running ToxCodAn-Genome" section). Or you can perform the BLAST search after running ToxCodAn-Genome as described below:
    - Download the UniProt or ToxProt database in FASTA format.
    - Make the BLAST database: `makeblastdb -dbtype prot -in path/to/uniprot.fasta -out blastDB/UNIPROT`
    - Run BLAST search: `blastx -evalue 0.01 -query path/to/toxin_annotation_cds.fasta -db blastDB/UNIPROT -out uniprot_blast.out -outfmt '6 qseqid sseqid pident length evalue qstart qend qlen sstart send slen salltitles'`
    - Check the output in tabular format (i.e., `uniprot_blast.out`).
    - It may help you to decide if the annotated toxin represents a confident toxin based on alignment features of the matched proteins in the UniProt/ToxProt database.

- ***Tip about toxins***: At this point, the tip in the "Curated toxin CDS set" section to acquire knowledge about toxins and their main features (like average size, protein domains structure, etc) is helpful. Briefly, search for these information in VenomZone, ToxProt/Uniprot, and published manuscripts.

- ***Tip about expression level***: If you have the venom gland transcriptome available for the target species, you can also use the expression level to help checking if the annotated toxin may represent a confident toxin. We expect to have high expression levels of toxin genes in venom gland transcriptomes. If you also have non-venom gland transcriptomes (i.e., transcriptomes from other tissues) available you can also use it to compare the annotated toxin expression levels among tissues. Comparing tissues may help to reveal if the annotated gene may represent a confident toxin (which may present high expression in venom glands), an endogenous gene (i.e., low or similar expression level in all tissues analyzed), or a pseudogene (i.e., no expression level in any tissue analyzed). See "Estimating expression level" to learn how to estimate the expression level.

Checking warning annotations

Most of the annotations marked as "warning" and added into the "annotation_warning.txt" file are mainly related to annotations containing a premature stop codon but keeping a complete gene structure (see the schematic representation below).

Status	Gene structure
Expected:	█████...........█████............█████.......████📍
Annotated:	█████...........█████............███📍▒▒.......▒▒▒▒📍

Note: █ - CDS; ▒ - CDS after a premature stop codon; 📍 - stop codon; . - intron

In many cases, it may represent a toxin with a premature stop codon, but it is important to double-check it. That's the reason we decide to add a "warning" file about annotations.

Here, we describe some features to double-check these "warning" annotations:

1 - You can check it by aligning its toxin to other toxins in the same species (when it is a multi-loci toxin family) or to closely-related species (when it is a single-locus toxin family). This may indicate if this premature stop codon is a result of an indel (insertion or deletion), which may alter the frame of the translated protein, or a single nucleotide change, which may turn an amino acid codon into a stop codon.

- If you confirm it represents a paralog copy with a premature stop codon, which may represent a toxin novelty, you can keep it in the final toxin annotation set. But modify its annotation by keeping the CDS till the stop codon position (i.e., adjust the annotation by altering the CDS' start and end

in the GTF file and removing any CDS after the stop codon).

- If you believe it may be an erroneous annotation generated by ToxCodAn-Genome, you can try to re-annotate it following the instructions in "Checking matched regions with no annotation" or remove it from the final toxin annotation set.

2- You can check if the exon/intron boundaries are well annotated:

GT···AG

- It is expected to have a "GT" as the splice acceptor and an "AG" as the splice donor.
    - Splice acceptor sequence ("GT") in the first two nucleotides of an intron.
    - Donor acceptor sequence ("AG") in the last two nucleotides of an intron.
    - If you are not used to this terminology and the splicing process, you can survey the literature to learn more about the topic. But you can find a nice explanation about this topic here.
- We noticed that this feature can be wrongly assigned for some specific annotations (when the region represents a pseudogene, an orphan exon region, or has several mutations when compared to the matched protein used as evidence). In this case, we strongly recommend checking the matched protein and this specific genomic region and following the instructions in "Checking matched regions with no annotation" to adjust its annotation and confirm its status.
    - If the annotation is reliable, it may represent a truncated paralog copy or a pseudogene region. Adjust the annotation to keep it in the final toxin annotation set.
    - If the annotation is not reliable, it may represent a region containing orphan exons. Then, you can remove it from the final toxin annotation set

3 - You can check if it may be an error in the genome assembly by erroneously assigning the nucleotides in this specific toxin region.

- You can re-map the DNA-seq data into the genome assembly.
- You must follow a pipeline accordingly to the dataset used to assembly the genome being analyzed, which may range from short-reads to long-reads technologies, or a mix of them.
    - You can use tools like BWA for short-reads and Minimap2 for long-reads.
- After mapping the genomic reads in the genome assembly, you can check the genomic region of the toxin annotation to understand if the "warning"

annotation may be a result of an erroneous assembly. Specifically, check the coverage and nucleotide composition of mapped reads.
    - You can use free tools like IGV and Tablet.
- In this case, we strongly recommend to review your genome assembly pipeline, consider adding an extra polish step, and/or consider testing several methods that may help to improve the final genome assembly.
- If the genomic region is correct, you can decide if the "warning" annotation is reliable or not, by keeping or removing it from the final toxin set based on the descriptions of steps *"1"* and *"2"*.

Checking matched regions with no annotation

Here, we describe how to use the free version of FGENESH+ available in the softberry web-server to perform the prediction of gene structure of the toxin in specific genomic regions. FGENESH+ is a well developed tool that performs the prediction of gene structure using the genomic region, a protein sequence as evidence, and a pre-build gene model as inputs (as mentioned by the authors', it is an "HMM plus similar protein-based gene prediction"). It helps helps to identify if some specific genomic regions contain or not a toxin gene.

After inspecting the `matched_regions.gtf` and detecting putative genomic regions that must be worthy to annotate with any other method you can follow the instructions below.

1 - Retrieve the genomic region to be used in the annotation.

- `samtools faidx genome.fasta "contig:start-end"`
    - Adjust `genome.fasta` accordingly
    - Adjust `contig`, `start`, and `end` accordingly (e.g., `contig_1:1234-5678`).
- It will print the sequence in the terminal that can be copied.
- If you want to save the genomic region in a file you can just add `> contig:start-end.fasta` at the end of the command.
    - e.g., `samtools faidx genome.fasta "contig:start-end" > contig:start-end.fasta`

2 - Retrieve the sequence from the database used as input in ToxCodAn-Genome (it will be used as protein evidence).

- `grep -A1 ">Toxin" toxin_database.fasta`
    - `grep` is a tool used to search for patterns in text files and it is generally installed by default on any UNIX system.
    - `-A1` (similar to "1 line after") is to print the line where it detects a

match (i.e., the toxin header in this case) and also the next line (i.e., the CDS in this case).
    - Modify the `>Toxin` and `toxin_database.fasta` accordingly.
- It will print the sequence in the terminal, which can be copied. ">Toxin ATG ... TAA"
- If you want to save it as a new file, just run: `grep -A1 ">Toxin" toxin_database.fasta > Toxin.fasta`

3 - Translate the CDS to use its protein sequence as evidence in FGENESH+ prediction.

- It can be done using any available tool designed for this purpose. Feel free to use the one that fits well to you.
- Here, we describe how to use the Translate tool from the ExPASy web-server.
- Paste the CDS in the "DNA or RNA sequence" tab -> click on "TRANSLATE!" -> copy the translated CDS (protein sequence)
    - You must copy the translated CDS from Frame 1 (the first that will appear in the list).
- ***Tip:*** you can translate all proteins from the toxin database using the terminal and make your life easier. You can use the script we designed for this purpose:
    - `translate_sequences_frame1.py toxin_database.fasta toxin_database_pep.fasta`
    - Use the `toxin_database_pep.fasta` to retrieve the peptide sequence directly as stated in the *"step 2"*.

4 - Run FGENESH+ prediction.

- Paste the genomic region in the tab "Paste nucleotide sequence here:" or load a file with the genomic region in FASTA format in the "Local file name:".
- Paste the protein sequence of the toxin to be used as evidence in the tab "Paste protein sequence here:" or load it is as a FASTA file in the "Local file name:"
- Select one of the pre-designed HMM models in the "Select organism specific gene-finding parameters:" tab. If the species you are analyzing is not in the list, select the closely related species (e.g., when analyzing snakes, you can select the "Anolis carolinensis" model).
- You can use the predicted CDS and protein sequences (at the bottom of the output page) to check the prediction by following the instructions in the "Checking toxin annotations" section.
- If it is a reliable prediciton, you can copy and save it in a TXT file (e.g.,

`fgenesh_output.txt`). Also, you can check and save the PDF report of the prediction by clicking in the "Show picture of predicted genes in PDF file" at the top of the page.

5 - After confirming that the prediction returned a reliable toxin annotation and saving it in a TXT file (e.g., `fgenesh_output.txt`), you must convert it into a GTF/GFF format.

- You can use the script we designed to convert the FGENESH+ annotation output format into a GTF format as follows:
- `AdjustingFgenesh.py fgenesh_output.txt TOXIN contig-start > fgenesh_output.gtf`
    - `fgenesh_output.txt` is the output from FGENESH+ from *"step 4"* saved in a TXT file, modify it accordingly.
    - `TOXIN` is the id of the gene annotated. For instance, you can use `SVMP` (if there is only one SVMP gene) or `SVMP-1` (where "1" can be any other number if there are more SVMP genes), when annotating an SVMP gene.
    - `contig-start` is the start of the genomic position used for screening. For instance, if analyzing the genomic region `contig_1:1234:5678` you must set `contig_1-1234` . This parameter must be correct to return a reliable GTF file.
    - By default, this script prints the GTF at the terminal, which can be copied and pasted on any file. To save the output directly in a file, you can set the `> fgenesh_output.gtf` accordingly.

Tip: If no annotation was returned and you still want to confirm it, you may consider adding extra nucleotides (e.g., 100-500 nts) up and downstream of the matched region. For instance, if the matched region is `contig_n:1000-2000` , you can use the genomic region `contig_n:900-2100` or `contig_n:500-2500` .

⚠️***Warning about running FGENESH+ to check annotations***⚠️: the freely available web version of FGENESH+ only accepts one genomic region and one protein sequence as evidence per run, which makes it a bit laborious and time consuming.

- Alternatively, you can look for any additional tools that may help you perform this task. But, based on our experience so far, we take advantage of this specific tool cause it returns the best results when inputting a good match between the protein used as evidence and the genomic region being annotated, which is a feature well assigned in the ToxCodAn-Genome's outputs.

NonToxin annotation

To perform NonToxin annotation (which is a general gene annotation), you can use any available tool. Here, we describe how to use funannotate. It is a package that performs an automated gene prediction and a functional annotation, by integrating several methods and tools. This program is well documented, and you can modify the commands in this guide to reach your purposes.

In this guide, we follow 4 easy-to-run steps:

1. Training (using RNA-seq data)
2. Prediction (using training data and additional protein databases)
3. Updating (using RNA-seq data to back over predictions and add UTR annotations)
4. Functional Annotation (annotate the final set of predictions with gene names, GO terms, and functional information)

Install funannotate and all dependencies to ensure it is properly working (you can follow the instructions here and run a test to ensure it is properly working (`funannotate test -t all --cpus 10`).

Download the database specific to the species being analyzed.

- e.g., for snakes we use the tetrapoda database: `funannotate setup -d funannotate_db -b tetrapoda`

Ensure to use a trimmed and filtered paired-end reads (see "Pre-processing of reads" subsection in "Transcriptome Assembly").

Repetitive region annotation (*Masking*)

First, ensure you have a soft-masked version of the genome being analyzed. Soft-masked genomes use lower-case letters to indicate repetitive regions.

If you don't have a soft masked version of the genome being analyzed, just expand this section and follow its instructions.

▾ Expand "Masking the genome" Section

Masking the genome

Here, we describe how to use the Extensive De-novo TE Annotator (EDTA) to identify and annotate transposable elements, long-terminal repeats (LTRs), and other repetitive regions in the genome.

```
perl EDTA.pl --genome genome.fasta --threads 20 --sensitive 1 --anno
1
```

- Adjust the number of threads accordingly to your system (`--threads`)
- It is important to note that this step is slow and will take a while (up to a few days).
  - This is slow due to the use of parameter `--sensitive 1` that also runs RepeatModeler/RepeatMasker to improve its final annotation.

Soft-masking

EDTA automatically only hard-masks the genome (which consists of replacing repetitive regions from nucleotide to "N"). Then, follow the steps below to soft-mask the genome:

```
#rename the hard-masked genome
mv genome.fasta.mod.MAKER.masked genome.fasta.mod.MAKER.hard.masked

#create a new directory to perform soft-masking
mkdir softmask && cd softmask
ln -s ../genome.fasta .
ln -s ../genome.fasta.mod.EDTA.anno/genome.fasta.mod.EDTA.RM.out .

#mimic how EDTA performs hard-masking to soft-mask the genome
perl ~/.conda/envs/EDTA/share/EDTA/util/make_masked.pl -genome
genome.fasta -rmout genome.fasta.mod.EDTA.RM.out -maxdiv 30 -minscore
1000 -minlen 1000 -hardmask 0 -misschar N -threads 20
mv genome.fasta.new.masked ../genome.fasta.mod.MAKER.soft.masked
cd ..
```

- Use the soft-masked version of the genome (`genome.fasta.mod.MAKER.soft.masked`) in further steps.

Training

```
funannotate train -i genome.fasta.mod.MAKER.soft.masked -o annotate \
        --left sample_tissue1_r1.fastq.gz sample_tissue2_r1.fastq.gz
sample_tissue3_r1.fastq.gz \
        --right sample_tissue1_r2.fastq.gz sample_tissue2_r2.fastq.gz
sample_tissue3_r2.fastq.gz \
        --no_trimmomatic --max_intronlen 30000 \
        --cpus 20 --species "Genus species"
```

- `--cpus` - set number of threads accordingly to your system.
- `--left` and `--right` - set any available RNA-seq data, but it must follow similar positions in "left" and "right" parameters.
- `--species` - set the species accordingly.
- This step is slow and will take a while (up to a few days).

Prediction

```
funannotate predict -i genome.fasta.mod.MAKER.soft.masked -o annotate \
        --transcript_evidence annotate/training/funannotate_train.stringtie.gtf.fasta \
        --protein_evidence $FUNANNOTATE_DB/uniprot_sprot.fasta \
        --busco_db tetrapoda --busco_seed_species chicken --organism other --max_intronlen 30000 \
        --cpus 20 --species "Genus species"
```

- `--cpus` - set number of threads accordingly to your system.
- `--busco_db` - set this parameter accordingly.
- `--busco_seed_species` - set this parameter accordingly.
- `--species` - set the species accordingly.
- `$FUNANNOTATE_DB/uniprot_sprot.fasta` - if you set funannotate correctly, it must work. Otherwise, set the path to `uniprot_sprot.fasta`.
- This step is slow and will take a while (up to a few days).

Updating

```
funannotate update -i annotate --cpus 20
```

- `--cpus` - set number of threads accordingly to your system.
- This step is slow and will take a while.

Functional Annotation

```
funannotate iprscan -i annotate -c 20 -m local --iprscan_path $PATH/to/IPRSCAN/interproscan.sh
funannotate annotate -i annotate --busco_db tetrapoda --cpus 20
```

- `--cpus` - set number of threads accordingly to your system.

- `--busco_db` - set this parameter accordingly.
- `$PATH/to/IPRSCAN/` - set it accordingly to the installed path of `interproscan.sh` in your system.
- Both steps are slow and will take a while.

Estimating expression level

If you have venom tissue transcriptomic data available for the individual/species, you can estimate the expression level of toxins and nontoxins annotated through our guide. Here, we describe two alternatives to estimate the expression level: (1) mapping reads into the genome; and (2) mapping reads into CDSs. Use the one that fits well with your purposes.

In the commands provided below, we assume that you are only estimating the expression level of the annotated toxins. If you also performed nontoxin annotation and are integrating both annotations, which is ideal, modify each step accordingly. If using single-end reads (or merged reads), modify the commands to run each step as single-end.

Mapping reads into genome

Here, we describe how to use `featureCounts` from Subread package to estimate expression level using the annotation file and mapping the reads into the genome (using Hisat2).

1 - Map reads into genome using Hisat2.

```
hisat2-build -p 20 genome.fasta GINDEX
hisat2 -p 20 --rg-id SAMPLE --rg SM:SAMPLE --summary-file
SAMPLE_hisat2_summary.txt -x GINDEX -1 SAMPLE_r1.fastq.gz -2
SAMPLE_r2.fastq.gz -S SAMPLE_mapped.sam
```

- `-p` - set the number of threads accordingly to your system.
- Modify `SAMPLE` accordingly.
- Modify `GINDEX` accordingly.
- If using single-end reads: `-U SAMPLE_single.fastq.gz` .
- If you have multiple samples, you just need to generate the index file once and set it to all samples (i.e., `GINDEX`).

2 - Estimate expression level using `featureCounts` .

```
featureCounts -T 20 -t CDS -g gene_id -a toxin_annotation.gtf -o
SAMPLE_counts.txt SAMPLE_mapped.sam
```

- `-T` - set the number of threads accordingly to your system.
- Modify `SAMPLE` accordingly.
- ***Tip:*** if you have multiple samples, you can set each mapped file in a comma-separated format at the end of the command (e.g., `SAMPLE1.sam SAMPLE2.sam SAMPLE3.sam SAMPLEn.sam`).

Tip about disk usage: to save disk space, you can consider converting the mapped files in `.sam` format into `.bam` format using Samtools. Follow the steps below:

```
samtools view -@ 20 -b -S -o SAMPLE_mapped.bam SAMPLE_mapped.sam
rm SAMPLE_mapped.sam
samtools sort -@ 20 SAMPLE_mapped.bam -o SAMPLE_mapped_sorted.bam
rm SAMPLE_mapped.bam
samtools index SAMPLE_mapped_sorted.bam
```

- Use `SAMPLE_mapped_sorted.bam` as input to `featureCounts` .

Mapping reads into CDSs

Here, we describe how to use RSEM (with Bowtie2) to estimate expression level using the CDSs of annotations retrieved from the genome.

1 - Extract CDSs from the final annotation set using GffRead.

```
gffread -x toxin_annotation_cds.fasta -g genome.fasta
toxin_annotation.gtf
```

2 - Estimate expression level using RSEM.

```
rsem-prepare-reference --bowtie2 toxin_annotation_cds.fasta
toxin_reference
rsem-calculate-expression -p 20 --bowtie2 --paired-end
SAMPLE_r1.fastq.gz SAMPLE_r2.fastq.gz toxin_reference SAMPLE
```

- `-p` - set the number of threads accordingly to your system.
- Modify `SAMPLE` accordingly.
- If using single-end reads: `rsem-calculate-expression --bowtie2`

```
SAMPLE_single.fastq.gz toxin_reference SAMPLE .
```

- If you have more than one sample, you can run `rsem-calculate-expression` for each sample by setting the same reference (i.e., `toxin_reference`).
- You can use the `SAMPLE.genes.results` output by RSEM to check the `expected_count`, `TPM`, and/or `FPKM` values obtained for each toxin.

⚠️***Warning about RSEM:*** based on our experience analyzing snake's venomics, the default mismatch rate considered by RSEM and Bowtie2 is around 10% of the read (e.g., for a read size of 150 bp, it accepts alignments with up to 15 bp of mismatches). This feature may return less confident expression level estimates for toxins with multiple loci. The high similarity observed among these toxins may increase the number of multi-mapped reads that can lead to erroneous estimates. The parameter `--bowtie2-mismatch-rate` allows you to adjust this feature, you can modify it to accept a lower percentage of mismatches to have more reliable expression estimates. You can set it to 5% (`--bowtie2-mismatch-rate 0.05`) or 2% (`--bowtie2-mismatch-rate 0.02`) to account for individual differences. We strongly recommend testing it.

Tip: to visualize expression levels, you can follow the "Expression Visualization" section in ToxCodAn's guide to venom gland transcriptomics[Nachtigall et al., 2021].

Plotting toxin loci

For visualization of the toxin loci annotation, we will move into the R environment and the nice package gggenes (which is an extension of ggplot2).

Install R in your system (`sudo apt install r-base` in ubuntu, modify it accordingly). You can also use Conda to install it in your active environment (`conda install -c conda-forge r-base`).

Then, you can convert the `toxin_annotation.gtf` file into a tsv file to be used as input to plot the toxin loci using the script we designed:

```
fromCDStoGENE.py toxin_annotation.gtf toxin_annotation_GENE.tsv
```

- follow the example data folder to run it as a test (`ToxCodAn-Genome/Guide/example_data/`), or adjust all file names and PATH accordingly.

Use the `toxin_annotation_GENE.tsv` as input to run the `PlotToxinLoci.R` script:

```
PlotToxinLoci.R -i toxin_annotation_GENE.tsv -o output
```

- It will output a file named `output_toxin_loci.pdf` with a chart of the toxin loci in the file `toxin_annotation_GENE.tsv`.
- Modify the `-i` and `-o` arguments accordingly.

The following toxin loci plot will be generated (if using the example data):

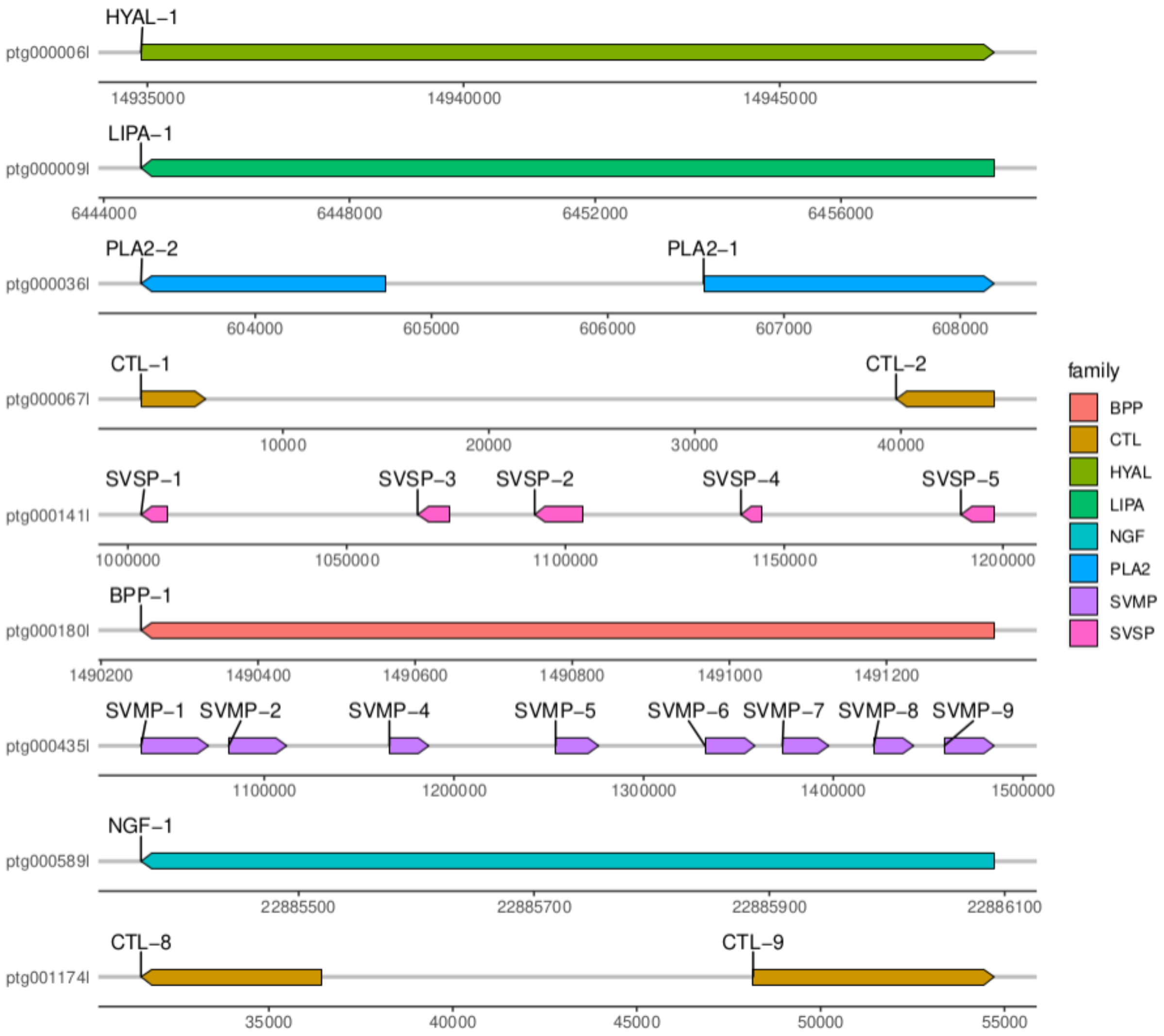

If you prefer to plot the chart manually, you can follow the steps below.

First, ensure that you have all the necessary packages installed properly. The script `PlotToxinLoci.R` install any package when needed, but here you need to ensure you have all of them installed.

Enter into R shell through the terminal (just type `R` and press "enter"), or using RStudio, and install the necessary packages to plot the toxin loci following this guide:

```
install.packages("tidyverse")
```

```
install.packages("ggplot2")
install.packages("ggrepel")
install.packages("gggenes")
q()
```

Then, convert the `toxin_annotation.gtf` file into a tsv file to be used as input to plot the toxin loci:

```
fromCDStoGENE.py toxin_annotation.gtf toxin_annotation_GENE.tsv
```

- The commands are assuming you are in the example data folder (`ToxCodAn-Genome/Guide/example_data/`), adjust all file names and PATH accordingly.

Enter into R shell using your terminal (just type `R` and press "enter") or using RStudio.

```
# first, set the environment
setwd("path/to/ToxCodAn-Genome/Guide/example_data/")
library(ggplot2)
library(gggenes)
library(ggrepel)
library(tidyverse)

# open the tsv file with gene information
df <- read.table("toxin_annotation_GENE.tsv", header=FALSE, sep = "\t")
colnames(df) <- c("gene","family","molecule","start","end","strand")
df <- df %>% mutate(strand = ifelse(strand == '+','forward','reverse'))

# plot and save toxin loci
pdf(file="output_toxin_loci.pdf")
ggplot(df, aes(xmin = start, xmax = end, y = molecule, fill = family, fc
  geom_gene_arrow(arrowhead_height = unit(3, "mm"), arrowhead_width = ur
  ggrepel::geom_text_repel(data = df, aes(x = start, y = molecule, label
  facet_wrap(~ molecule, scales = "free", ncol = 1) +
  ylab('') +
  xlab('') +
  theme_genes()
dev.off()

# end
```

- Modify `setwd("path/to/ToxCodAn-Genome/Guide/example_data/")` accordingly.

Tip: You can also modify the colors of each toxin family accordingly, by using a list indicating the toxin family symbol and the desired color:

```
toxin.colors <- c(SVMP = "blue", CTL = "green", PLA2 = "red", BPP = "bla
```

Then, indicate it into the function `scale_fill_manual(values=toxin.colors)` when running the plot command:

```
ggplot(df, aes(xmin = start, xmax = end, y = molecule, fill = family, fo
  geom_gene_arrow(arrowhead_height = unit(3, "mm"), arrowhead_width = ur
  ggrepel::geom_text_repel(data = df, aes(x = start, y = molecule, label
  facet_wrap(~ molecule, scales = "free", ncol = 1) +
  ylab('') +
  xlab('') +
  scale_fill_manual(values=toxin.colors) +
  theme_genes()
```

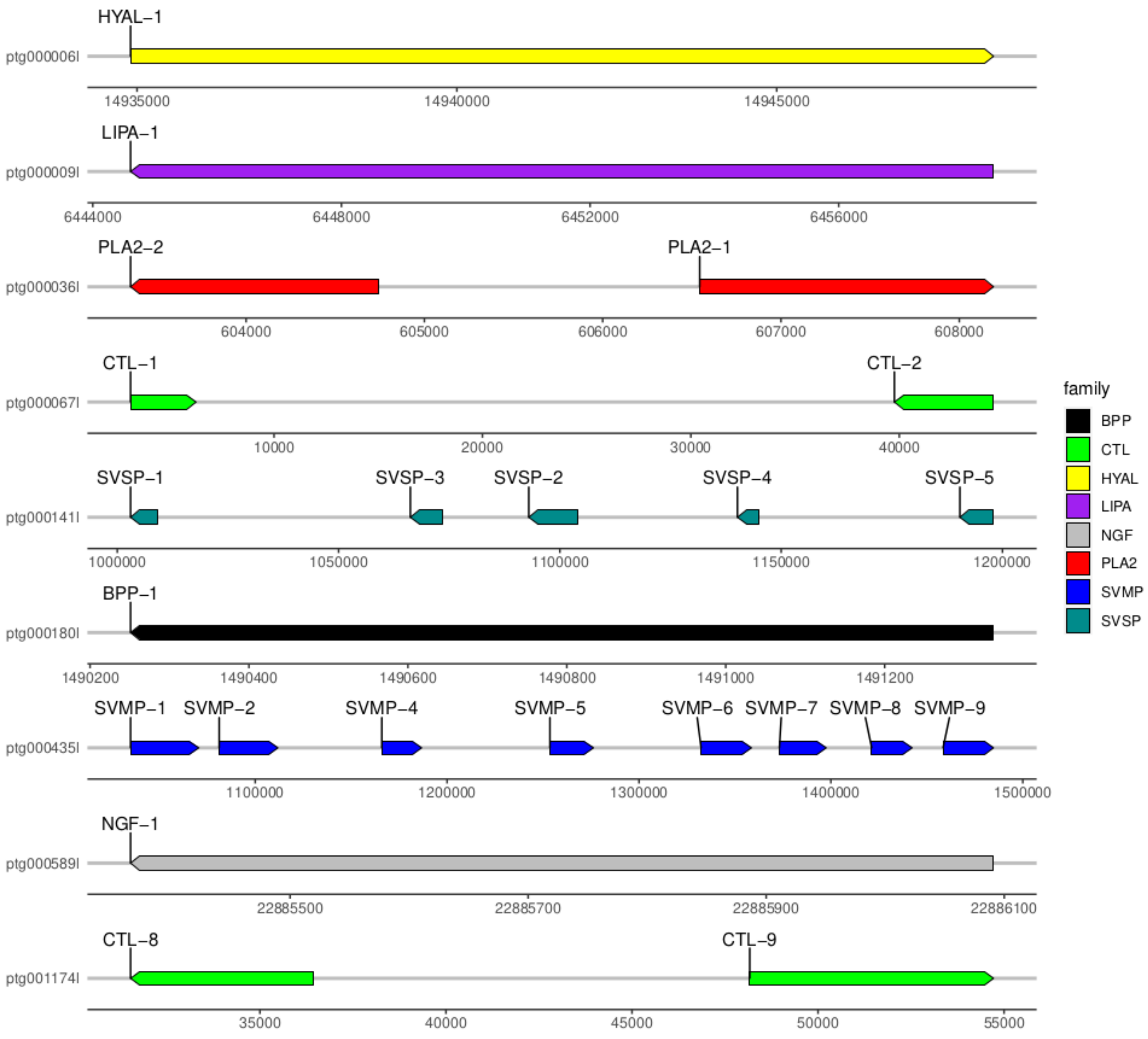

NCBI submission alias

To help the user to submit the final toxin annotation to the NCBI, we designed a script to add a `product` feature into the descrition column of the annotation file. This script was designed to follow the NCBI recommendations for CDS annotations (as described in the GenBank guidelines). Please, read their guidelines to generate a high-quality alias file.

This script takes the gene symbol of the annotate toxins and add a `product` feature with the gene name based on an alias file. This alias file is a tab-delimited TXT file containing two columns, whereas the first column must be the gene symbol (e.g., `SVMP`) and the second column must be the gene name with no abbreviations (e.g., `snake venom metalloproteinase`). Of note, this script has a built-in alias designed for most snake toxins (which is the venomous lineage our group often works), but we strongly recommend the user to design and provide their own alias file, because the gene symbols and names may differ among users working with distinct venomous lineages. As an example, we have the built-in alias available as a TXT file in the "ToxCodAn-Genome/misc/" folder.

After designing you alias file (or complementing the available alias file), you can generate the NCBI submission annotation file using the `AliasNCBIsubmission.py` script:

```
AliasNCBIsubmission.py -g toxin_annotation_final.gtf -a alias.txt -o
toxin_annotation_NCBI.gtf
```

- If there is no matched gene symbol in the alias file, the entry will be marked as "hypothetical protein" (i.e., `product "hypothetical protein"`). But this issue will be printed at the terminal.
