# Supplementary material for: ToxCodAn-Genome: an automated pipeline for toxin-gene annotation in genome assembly of venomous lineages
Source: Gigascience. 2024 Jan 18;13:giad116. doi: 10.1093/gigascience/giad116 (PMC10797961; doi:10.1093/gigascience/giad116)
Supplement: giad116_Supplemental_Files [file giad116_supplemental_files.zip › Supplementary_file_4.pdf]

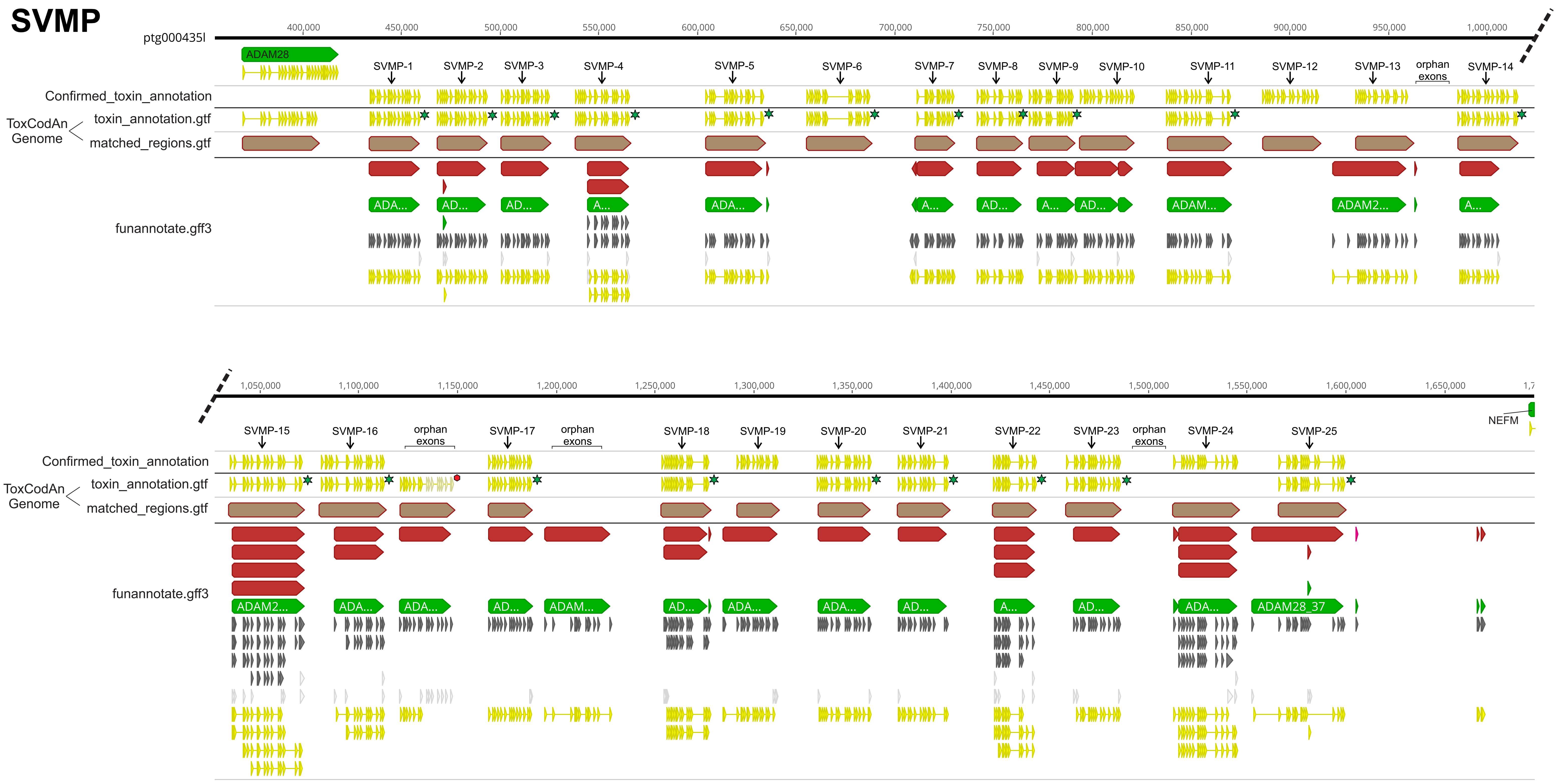

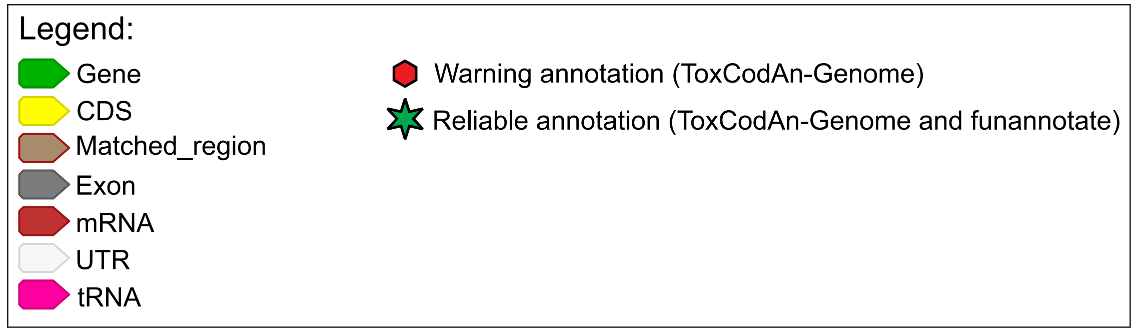

Supplementary file 4. Charts showing the differences between ToxCodAn-Genome and funannotate annotations in toxin loci of *Bothrops alternatus* genome.

The symbol "★" indicates when the annotation represents a confident toxin annotation.

The symbol "⬢" indicates a warning annotation marked by ToxCodAn-Genome.

The "matched_region" indicates regions matching full-length toxin CDSs as identified by ToxCodAn-Genome.

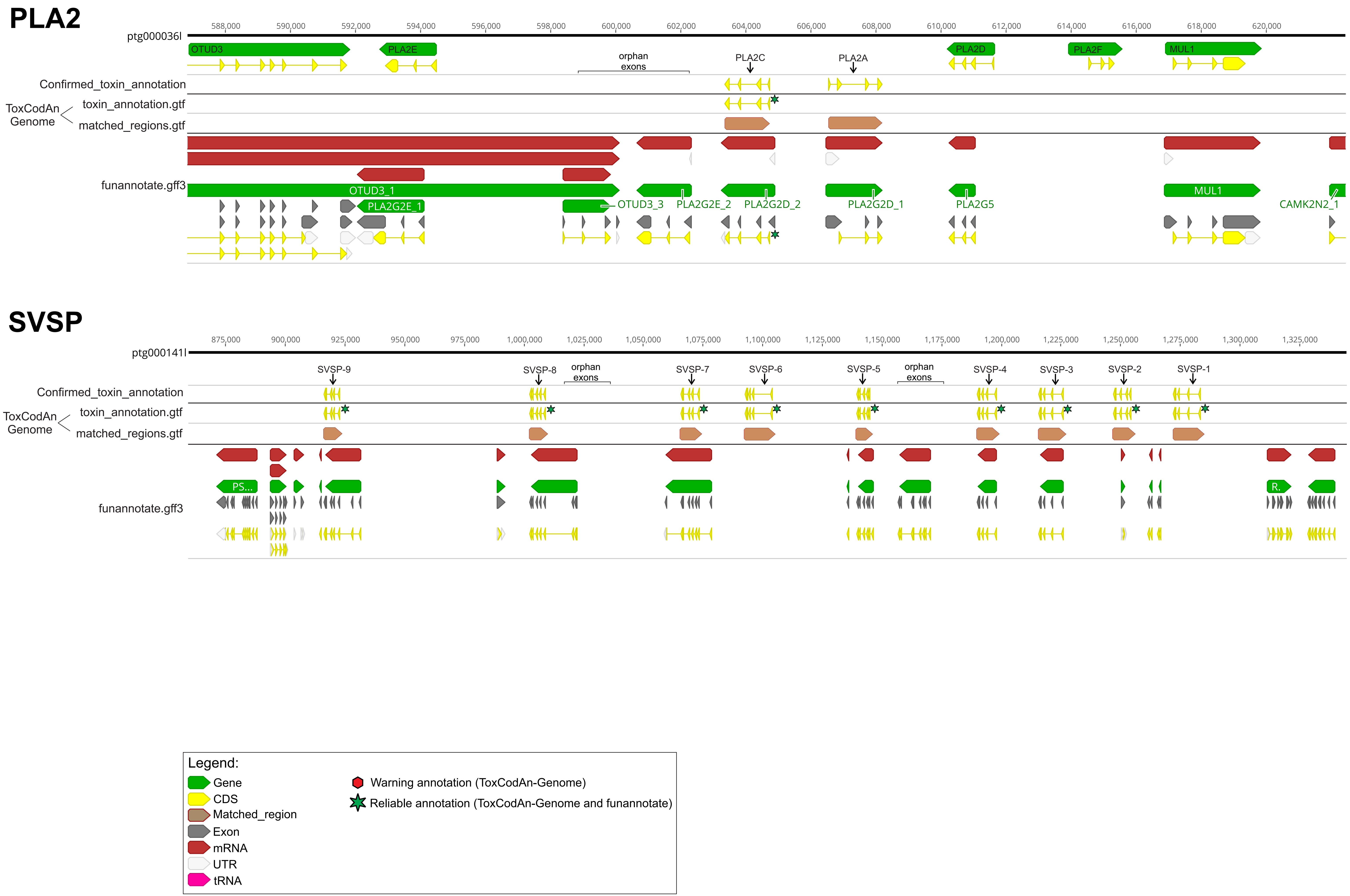

PLA2
ptg000036l
OTUD3
PLA2E
orphan exons
PLA2C
PLA2A
PLA2D
PLA2F
MUL1
Confirmed_toxin_annotation
ToxCodAn Genome
toxin_annotation.gtf
matched_regions.gtf
funannotate.gff3
OTUD3_1
PLA2G2E_1
OTUD3_3
PLA2G2E_2
PLA2G2D_2
PLA2G2D_1
PLA2G5
CAMK2N2_1
SVSP
ptg000141l
SVSP-9
SVSP-8
SVSP-7
SVSP-6
SVSP-5
SVSP-4
SVSP-3
SVSP-2
SVSP-1
PS...
R.
Legend:
Gene
CDS
Matched_region
Exon
mRNA
UTR
tRNA
Warning annotation (ToxCodAn-Genome)
Reliable annotation (ToxCodAn-Genome and funannotate)

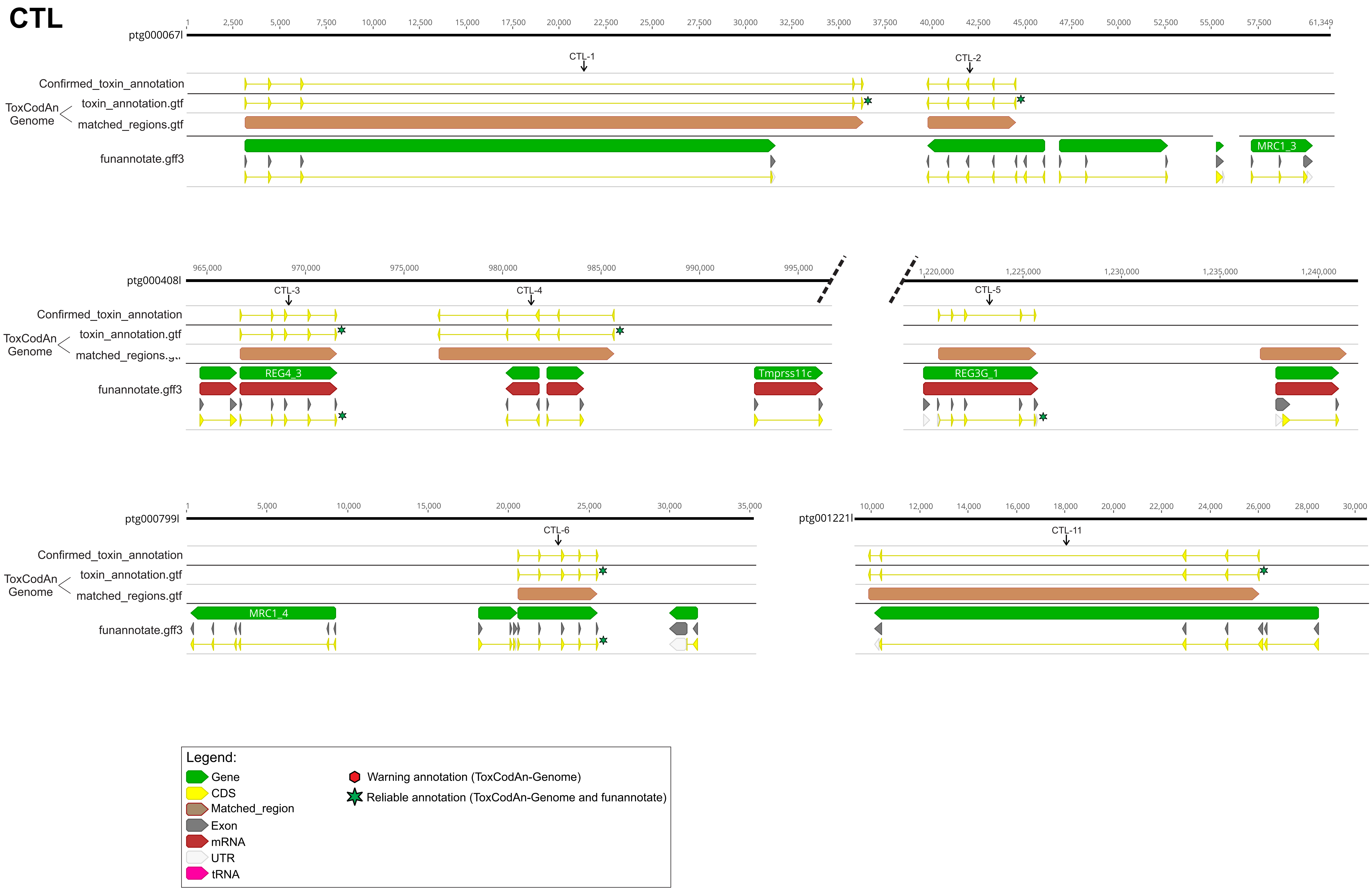

CTL
ptg000067l
CTL-1
CTL-2
Confirmed_toxin_annotation
ToxCodAn Genome
toxin_annotation.gtf
matched_regions.gtf
funannotate.gff3
MRC1_3
ptg000408l
CTL-3
CTL-4
CTL-5
REG4_3
Tmprss11c
REG3G_1
ptg000799l
CTL-6
MRC1_4
ptg001221l
CTL-11
Legend:
Gene
CDS
Matched_region
Exon
mRNA
UTR
tRNA
Warning annotation (ToxCodAn-Genome)
Reliable annotation (ToxCodAn-Genome and funannotate)

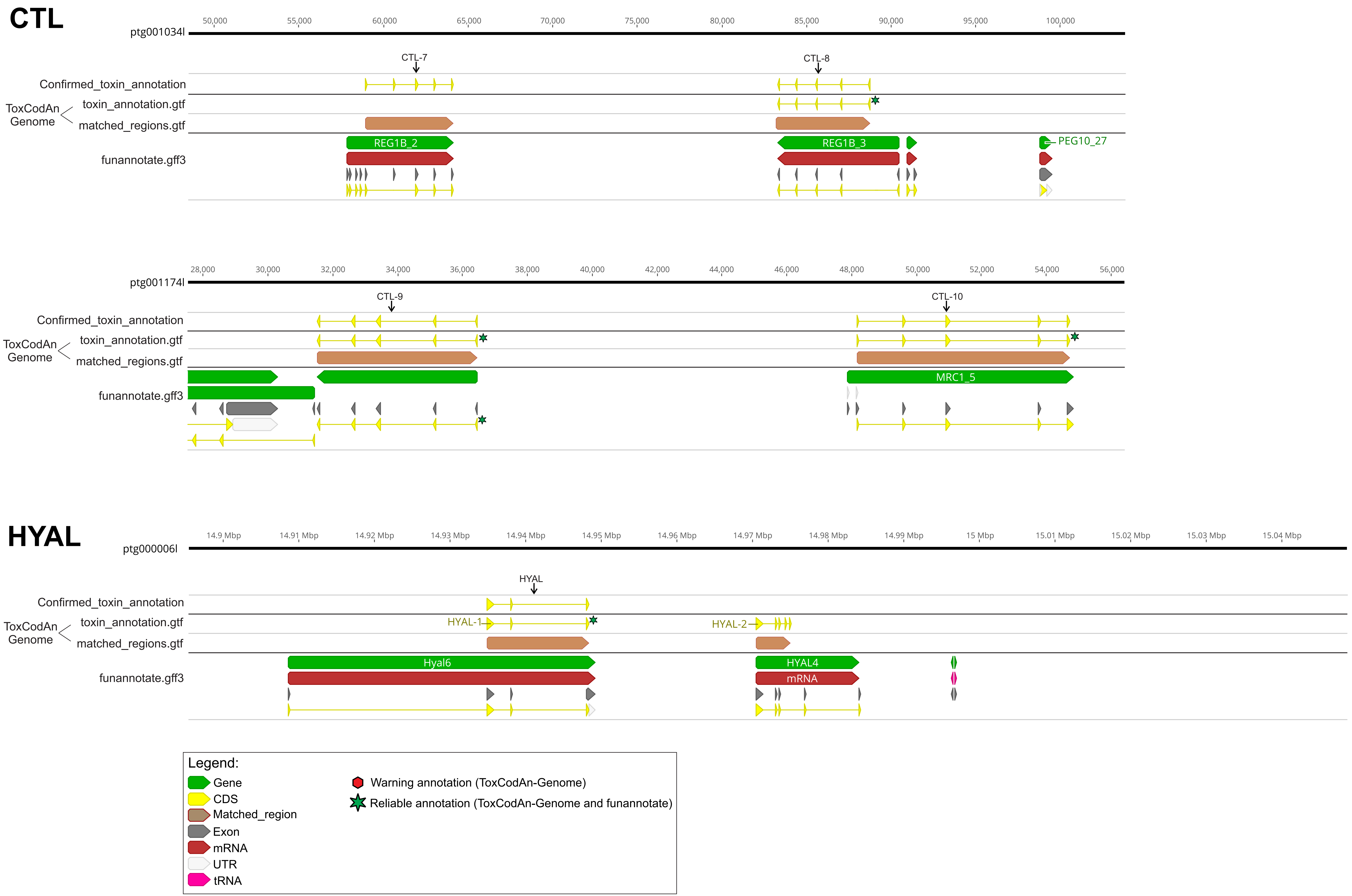

CTL
ptg001034l
50,000
55,000
60,000
65,000
70,000
75,000
80,000
85,000
90,000
95,000
100,000
CTL-7
CTL-8
Confirmed_toxin_annotation
ToxCodAn Genome
toxin_annotation.gtf
matched_regions.gtf
REG1B_2
REG1B_3
PEG10_27
funannotate.gff3
ptg001174l
28,000
30,000
32,000
34,000
36,000
38,000
40,000
42,000
44,000
46,000
48,000
50,000
52,000
54,000
56,000
CTL-9
CTL-10
Confirmed_toxin_annotation
ToxCodAn Genome
toxin_annotation.gtf
matched_regions.gtf
MRC1_5
funannotate.gff3
HYAL
ptg000006l
14.9 Mbp
14.91 Mbp
14.92 Mbp
14.93 Mbp
14.94 Mbp
14.95 Mbp
14.96 Mbp
14.97 Mbp
14.98 Mbp
14.99 Mbp
15 Mbp
15.01 Mbp
15.02 Mbp
15.03 Mbp
15.04 Mbp
HYAL
Confirmed_toxin_annotation
ToxCodAn Genome
toxin_annotation.gtf
HYAL-1
HYAL-2
matched_regions.gtf
Hyal6
HYAL4
mRNA
funannotate.gff3
Legend:
Gene
CDS
Matched_region
Exon
mRNA
UTR
tRNA
Warning annotation (ToxCodAn-Genome)
Reliable annotation (ToxCodAn-Genome and funannotate)

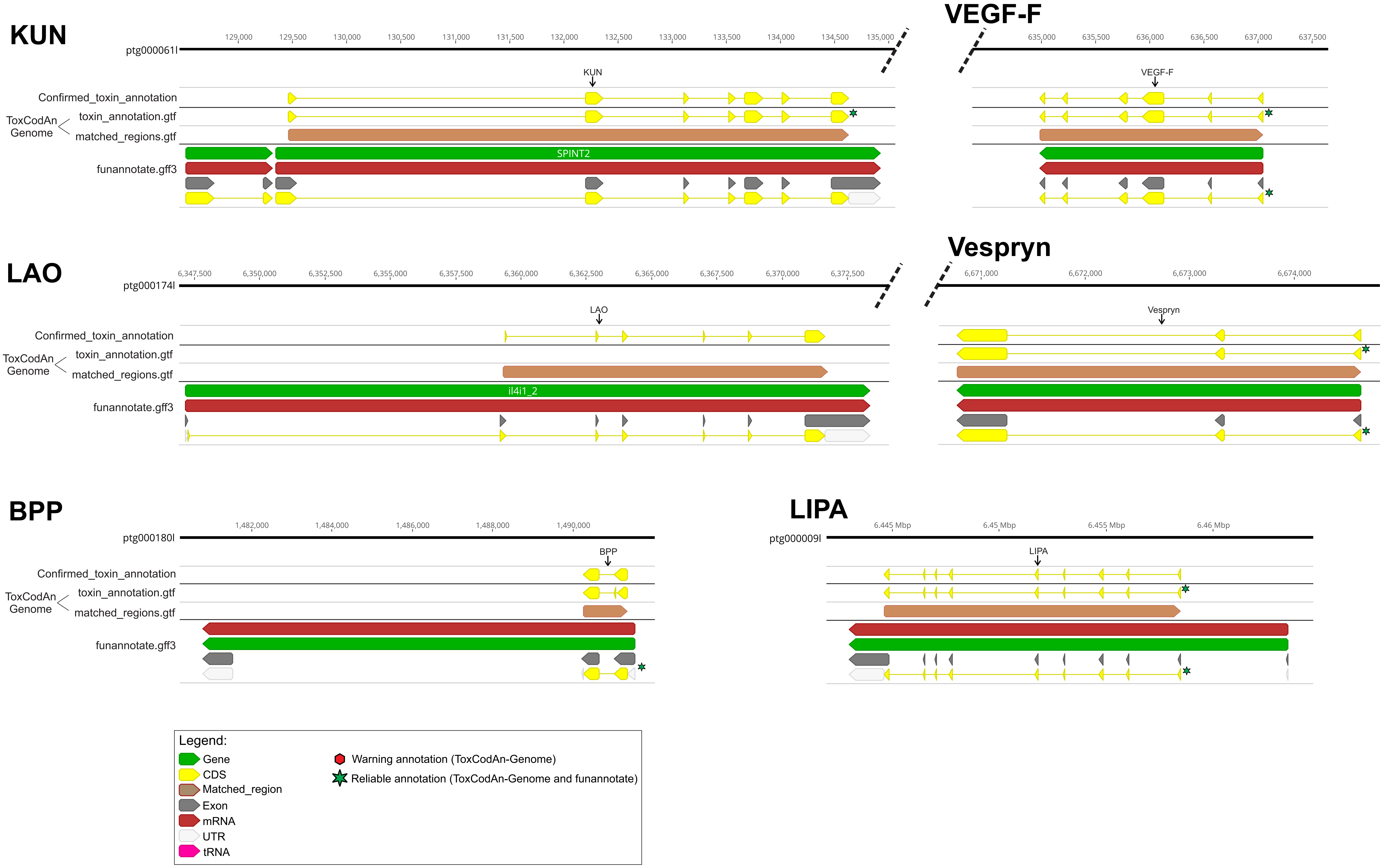

KUN
VEGF-F
ptg000061l
Confirmed_toxin_annotation
ToxCodAn Genome
toxin_annotation.gtf
matched_regions.gtf
funannotate.gff3
SPINT2
LAO
Vespryn
ptg000174l
il4i1_2
BPP
LIPA
ptg000180l
ptg000009l
Legend:
Gene
CDS
Matched_region
Exon
mRNA
UTR
tRNA
Warning annotation (ToxCodAn-Genome)
Reliable annotation (ToxCodAn-Genome and funannotate)

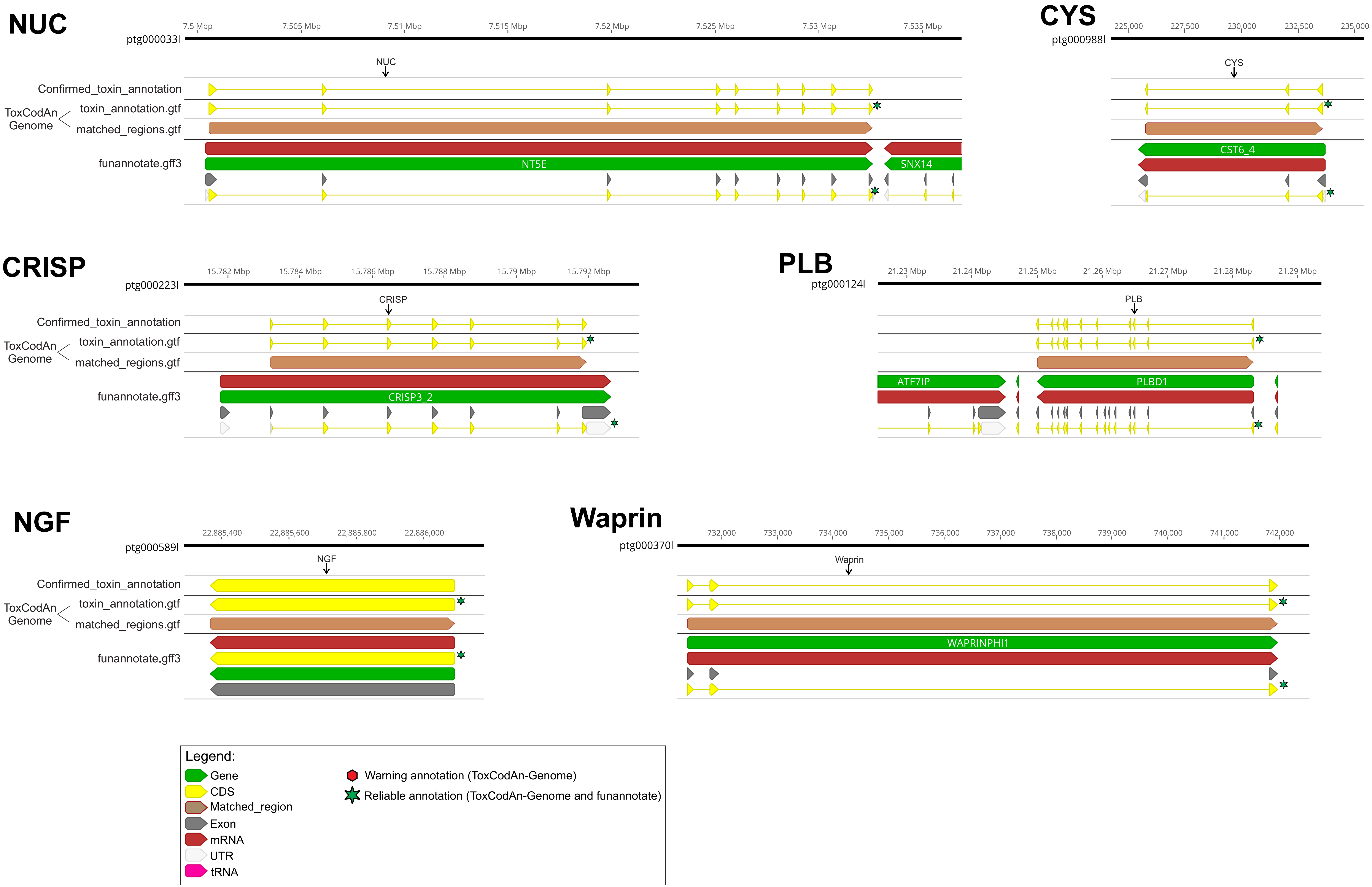
NUC
ptg000033l
7.5 Mbp
7.505 Mbp
7.51 Mbp
7.515 Mbp
7.52 Mbp
7.525 Mbp
7.53 Mbp
7.535 Mbp
Confirmed_toxin_annotation
ToxCodAn Genome
toxin_annotation.gtf
matched_regions.gtf
funannotate.gff3
NT5E
SNX14
CYS
ptg000988l
225,000
227,500
230,000
232,500
235,000
CST6_4
CRISP
ptg000223l
15.782 Mbp
15.784 Mbp
15.786 Mbp
15.788 Mbp
15.79 Mbp
15.792 Mbp
CRISP3_2
PLB
ptg000124l
21.23 Mbp
21.24 Mbp
21.25 Mbp
21.26 Mbp
21.27 Mbp
21.28 Mbp
21.29 Mbp
ATF7IP
PLBD1
NGF
ptg000589l
22,885,400
22,885,600
22,885,800
22,886,000
Waprin
ptg000370l
732,000
733,000
734,000
735,000
736,000
737,000
738,000
739,000
740,000
741,000
742,000
WAPRINPHI1
Legend:
Gene
CDS
Matched_region
Exon
mRNA
UTR
tRNA
Warning annotation (ToxCodAn-Genome)
Reliable annotation (ToxCodAn-Genome and funannotate)
