# Supplementary material for: ToxCodAn-Genome: an automated pipeline for toxin-gene annotation in genome assembly of venomous lineages
Source: Gigascience. 2024 Jan 18;13:giad116. doi: 10.1093/gigascience/giad116 (PMC10797961; doi:10.1093/gigascience/giad116)
Supplement: giad116_Response_to_Reviewer_Comments_Original_Submission [file giad116_response_to_reviewer_comments_original_submission.pdf]

Author's Response To Reviewer Comments

Author's response to Editor:
===> First, we thank the editor for considering our manuscript to be published in the journal “GigaScience”. We also thank all the reviewers very much for the thorough reading of the manuscript, and for bringing excellent points to improve it. Please find below a point by point response and comments in attention to your concerns. Also, the modifications in the revised manuscript are marked in red to facilitate the review process.

Reviewer reports:
Reviewer #1: Authors reported the development of a software designed to perform automated toxin annotation in genomes of venomous lineages. The pipeline design enables users to retrieve full-length coding sequences of toxins, allowing the detection of novel truncated paralogs and pseudogenes. The tool (ToxCodAn-Genome(was tested using 12 genomes of venomous lineages, covering both vertebrate and invertebrate animals, and further validated in a case study on Bothrops alternatus genome. The study is well executed, and the work is useful for future evolutionary and biomedical studies in the field of venomics. Authors also provide the tool freely on-line; this is commendable. There are a few suggestions, mainly for clarification, given below for authors' consideration. Addressing these may help improve the manuscript for reading especially for readers not familiar to the subject.

Author’s response:
===> We thank Reviewer #1 for raising some interesting concerns that helped to improve the manuscript. We believe we have addressed all points argued by the reviewer in the manuscript, which are also answered below.

1. Methods: "To build the ToxinDB, we retrieved full-length toxin CDSs from the nucleotide archive and the TSA databases of NCBI (https://www.ncbi.nlm.nih.gov/)". Please provide further details regarding the recruitment and selection of toxins for the construction of "Toxin Databases" (DB). It would be good if authors could pre-define toxins for the different lineages which they studied. The classification of toxins, non-toxins, and toxin-like components can vary from one study to another.

Author’s response:
===> We complemented the text to better clarify this point. Please, see the subsection “Toxin Databases” in the “Methods” section of the revised manuscript.

2. Results: Figure 2. Label and caption - Should be DBTR or TRDB? Also, some toxins/proteins anticipated in snake venoms such as phosphodiesterase, 5' nucleotidase, L-amino acid oxidase, were not included in the plot box and perhaps the numbers too. Please explain.

Author’s response:
===> We modified the caption to follow the manuscript text and figures (i.e., DBTR). Of note, 5' nucleotidase and L-amino acid oxidase are named as NUC and LAAO, respectively. The lack of phosphodiesterase is not related to a lack of annotation, but it is related to the fact that we decided to only keep moderate to highly expressed toxin genes for most of the snake species analyzed in the chart to have a cleaner plot. Following the reviewer's suggestion, we also included the phosphodiesterase in the snake species boxplot to show its TRR (named as PDE) in the revised manuscript. See Figure 2 in the revised manuscript.

3. Results: Authors presented the venom composition and toxin repertoire of Bothrops alternatus in the upper panel of Figure 4. The term "venom composition" is typically used to describe the proteins expressed in a venom, as in most proteomic studies. Perhaps authors can consider specifying in the caption and the text (manuscript) that these are the toxin transcripts for putative protein composition of the venom (not all transcripts identified in venom gland transcriptome are translated, and there are also post-translational

modifications which will further modify the final 'venom composition'). Also, please indicate the percentage values for the respective sections of the pie chart, and how the abundance (%?) is determined.

Author’s response:
===> We modified the figure and caption accordingly.

4. Results: The toxins annotated and identified are typical except LIPA (lipase) - is this a known protein family expressed in snake venom (secretory protein?) Please explain how or why LIPA is included as part of the venom toxin genes.

Author’s response:
===> We agree that LIPA should not be considered a toxin in viper species. However, it is detected at venom gland transcriptomes of some viper species at low expression levels (like in B. alternatus; Casewell et al., 2009) and can also be detected in some elapidae and dipsadidae snakes (Corrêa-Netto et al., 2011; Campos et al., 2016; Junqueira-de-Azevedo et al., 2016; Tioyama et al., 2023). However, considering the low expression level of LIPA in B. alternatus and following the reviewer’s concern, we removed it from the final toxin set of B. alternatus and updated the figure, results and discussions to account for this decision. Of note, we decided to keep LIPA and other lowly expressed toxin-like genes often detected in venom gland transcriptomes in the database to allow the user to have them annotated and then consider the guide, the expression level in venom gland transcriptome, the presence or absence in venom proteomics, and their own knowledge to decide whether to account for it in the toxin set or not.

References:
Casewell, N. R., Harrison, R. A., Wüster, W., & Wagstaff, S. C. (2009). Comparative venom gland transcriptome surveys of the saw-scaled vipers (Viperidae: Echis) reveal substantial intra-family gene diversity and novel venom transcripts. BMC genomics, 10(1), 1-12.

Campos, P. F., Andrade-Silva, D., Zelanis, A., Paes Leme, A. F., Rocha, M. M. T., Menezes, M. C., ... & Junqueira-de-Azevedo, I. D. L. M. (2016). Trends in the evolution of snake toxins underscored by an integrative omics approach to profile the venom of the colubrid Phalotris mertensi. Genome Biology and Evolution, 8(8), 2266-2287.

Corrêa-Netto, C., de LM Junqueira-de-Azevedo, I., Silva, D. A., Ho, P. L., Leitão-de-Araújo, M., Alves, M. L. M., ... & Calvete, J. J. (2011). Snake venomics and venom gland transcriptomic analysis of Brazilian coral snakes, Micrurus altirostris and M. corallinus. Journal of Proteomics, 74(9), 1795-1809.

Junqueira-de-Azevedo, I. L., Campos, P. F., Ching, A. T., & Mackessy, S. P. (2016). Colubrid venom composition: an-omics perspective. Toxins, 8(8), 230.

Tioyama, E. C., Bayona-Serrano, J. D., Portes-Junior, J. A., Nachtigall, P. G., de Souza, V. C., Beraldo-Neto, E., ... & Freitas-de-Sousa, L. A. (2023). The venom composition of the snake tribe Philodryadini:‘Omic’techniques reveal intergeneric variability among South American racers. Toxins, 15(7), 415.

5. In discussion or conclusion - Suggest to include limitations of the study and the tool developed. In addition, how is the tool maintained and if any updating or curation will be done from time to time.

Author’s response:
===> Following the reviewer's suggestion, we included a paragraph about limitations and update policy for maintenance of the code and databases in the “Discussion” section.

Reviewer #2: Please find relevant comments in the attached PDF. [Editor's note: this is available for download in Editorial Manager - please let me know should you have problems locating the file]

Author's response:
===> We thank Reviewer #2 for the thorough reading of the manuscript, debugging the tool, and for bringing excellent points to improve both of them. Please find below a point by point response and comments in attention to your concerns.

Problem1
As suggested by the reviewer, we modified ToxCodAn-Genome to be compatible with exonerate version 2.2 as well.

Problem2
The minor differences observed by the reviewer (i.e, LIPA <-> SVLIPA, VEGF <-> VEGFF, Vespryn <-> ohanin) are related to differences in names of homolog toxins. Unfortunately, there are no naming conventions determining what must be used as gene names/symbols for these and few other toxins. For these specific toxins, some authors frequently use the first alternatives, whereas other authors use the second alternatives to name them. Here, we opted to keep them as mentioned in their original entries in SRA/manuscripts and let the user decide what name to use.
We believe that such differences do not affect the final output, because these minor differences are related to homolog proteins. Moreover, our tool has a parameter that allows the user to indicate a protein database of known toxins (like ToxProt) to have a report about each annotated toxin and the best hit within the specified database. Our guide recommends the users to follow some steps to check all of the annotations and ensure a confident final toxin set. At the checking step, the user can rename the annotated toxins following any specific nomenclature.
Additionally, we dig deeper to check the reason behind this behavior. We noticed that the "pandas" module from python (https://pypi.org/project/pandas/) usually assigns the order of rows randomly when similar values are present in the specified column(s) using the "sort_values" function. We use this module and function to sort the BLAST output by their matching region position in the genome to detect matching overlaps and further processing each region with their matching toxins. It is an issue that will take place every time a hit has a similar matching position in the genome being analyzed, which is highly putative considering the completeness of the database and the highly similarity of homolog toxins.

Problem3
We wrote a function to perform a sanity check of the input files and return an explicit error to help the user fix it (if needed).

Problem4
We followed the reviewer's recommendation and performed an additional test. Specifically, we used the set of toxin genes annotated as described in the original publications of the viper species selected for the testing sets (i.e., Azemiops feae, Bothrops jararaca, and Crotalus tigris). We used their toxins CDSs as databases and analyzed when they were correctly annotated (in the file "toxin_annotation.gtf"), and also, when they were present as a matching toxin with or without an annotation (i.e., presence in the file "matched_regions.gtf"). We deeply analyzed when a toxin was not present in the "matched_regions.gtf" file to better understand the reason behind this behavior and point it to the readers. Additionally, we estimated the expression level of the toxin genes to check if the annotated and non-annotated toxin genes are highly or lowly expressed toxins in the venom tissue transcriptome.
Please, see the new subsections "Proof of concept test" and "Proof of concept test of ToxCodAn-Genome" in the "Methods" and "Results" sections, respectively. See also the discussion about this issue in the "Discussion" section.

Wording Fixes and Suggestions
We followed the reviewer's suggestions and modified the text accordingly.

Reviewer #3: First, I want to apologize for the delay in providing my comments/review. The review request arrived the same day the fall semester started and things have been a bit busy. I would have declined the review considering the busy schedule, however, based on the title and abstract I was excited to read this manuscript (and fit it in my schedule as I could).

Thankfully I was not disappointed. This manuscript is extremely well written. I have absolutely no suggestions for changes or modifications and think it's acceptable for publication in its current form. ToxCodAn-Genome addresses the challenges of accurately annotating toxin genes in genomic regions and circumvents issues surrounding poorly characterized lineages by using automated genome annotation tools. ToxCodAn-Genome is the first software designed to perform automated toxin annotation in genomes of venomous lineages. The package itself is customizable, allowing users to test different parameters to improve the final toxin annotation set for the studied lineage. The tools are build into the program and allow users to customize several aspects of the annotation procedures, with "warnings" for less than reliable annotaitons. ToxCodAn-Genome circumvents a lot of laborious procedures involved with the manual inspection to identify the complete set of genes. There are many
positive aspects of this program and its impact on the field of venomics and the resulting manuscript will no doubt have a positive impact on the field. Many of the existing pipelines rely too much on training datasets resulting in misguided results, or heavily rely on time consuming curation. The software also provides a detailed guide that describes the processes to build a custom toxin database, perform nontoxin annotation, quantify gene expression, and plot the toxin loci for reports and publications. ToxCodAn-Genome also has the potential to facilitate the discovery of new toxin compounds and the development of new medicines, accelerating these fields in areas with the ever growing genomic data.

The only feedback I have for manuscript improvement is that the authors should consider including x-axis labels in Figures 2 and 3. It is defined in the figure legend, but a label at the bottom of each A/B side for Fig 2 & 3 may assist in readers interpretations of these figures.

Unfortunately I did not have time to test the program myself (due to my schedule limiting my ability to do so, not because of the program or authors). Considering how the past month has been it'd probably be another month before I could find time in my schedule to run it. The github website is setup in a way to guide users through the implementation of the program and seems very straightforward with a user guide that is easy to follow. Although I am not able to identify any shortcomings of the ToxCodAn-Genome software, it is worth noting that the software is designed specifically for venomous lineages, and its performance on non-venomous lineages is unknown (although it has potential for characterizing other gene family types in other genomes (a benefit)). One down side is that poorly studied venomous lineages or atypical venomous organisms may result in a significant number of false positives or mis-characterized toxins. This shortcoming is not anything the authors could
remedy, much like a majority of similar software packages of this type out on the market, the users need to understand the programs own limitations and expectations to use them effectively.

Author’s response:
===> We thank the reviewer #3 for reading the manuscript alongside a busy schedule. We appreciate the commentaries and indication for acceptance of the manuscript.

Of note, the x-axis labels of Figure 2 and 3 were present at the top of each figure. However, we modified it to be at the bottom to avoid such misunderstanding from the readers. Hopefully, it is well suited now. Thank you for this feedback.
