# Supplementary material for: ToxCodAn-Genome: an automated pipeline for toxin-gene annotation in genome assembly of venomous lineages
Source: Gigascience. 2024 Jan 18;13:giad116. doi: 10.1093/gigascience/giad116 (PMC10797961; doi:10.1093/gigascience/giad116)
Supplement: giad116_Response_to_Reviewer_Comments_Revision_1 [file giad116_response_to_reviewer_comments_revision_1.pdf]

Author's Response To Reviewer Comments

We thank the editor and reviewers for bringing excellent points to improve the final code and manuscript and for accepting our manuscript for publication.
