# Supplementary material for: ToxCodAn-Genome: an automated pipeline for toxin-gene annotation in genome assembly of venomous lineages
Source: Gigascience. 2024 Jan 18;13:giad116. doi: 10.1093/gigascience/giad116 (PMC10797961; doi:10.1093/gigascience/giad116)
Supplement: giad116_Reviewer_1_Report_Original_Submission — Choo Hock Tan -- 8/12/2023 Reviewed [file giad116_reviewer_1_report_original_submission.pdf]

Reviewer Report

Title: ToxCodAn-Genome: an automated pipeline for toxin-gene annotation in genome assembly of venomous lineages

Version: Original Submission **Date:** 8/12/2023

Reviewer name: Choo Hock Tan

Reviewer Comments to Author:

Authors reported the development of a software designed to perform automated toxin annotation in genomes of venomous lineages. The pipeline design enables users to retrieve full-length coding sequences of toxins, allowing the detection of novel truncated paralogs and pseudogenes. The tool (ToxCodAn-Genome(was tested using 12 genomes of venomous lineages, covering both vertebrate and invertebrate animals, and further validated in a case study on Bothrops alternatus genome. The study is well executed, and the work is useful for future evolutionary and biomedical studies in the field of venomics. Authors also provide the tool freely on-line; this is commendable. There are a few suggestions, mainly for clarification, given below for authors' consideration. Addressing these may help improve the manuscript for reading especially for readers not familiar to the subject.1. Methods: "To build the ToxinDB, we retrieved full-length toxin CDSs fromthe nucleotide archive and the TSA databases of NCBI (https://www.ncbi.nlm.nih.gov/)". Please provide further details regarding the recruitment and selection of toxins for the construction of "Toxin Databases" (DB). It would be good if authors could pre-define toxins for the different lineages which they studied. The classification of toxins, non-toxins, and toxin-like components can vary from one study to another.2. Results: Figure 2. Label and caption - Should be DBTR or TRDB? Also, some toxins/proteins anticipated in snake venoms such as phosphodiesterase, 5' nucleotidase, L-amino acid oxidase, were not included in the plot box and perhaps the numbers too. Please explain.3. Results: Authors presented the venom composition and toxin repertoire of Bothrops alternatus in the upper panel of Figure 4. The term "venom composition" is typically used to describe the proteins expressed in a venom, as in most proteomic studies. Perhaps authors can consider specifying in the caption and the text (manuscript) that these are the toxin transcripts for putative protein composition of the venom (not all transcripts identified in venom gland transcriptome are translated, and there are also post-translational modifications which will further modify the final 'venom composition'). Also, please indicate the percentage values for the respective sections of the pie chart, and how the abundance (%?) is determined.4. Results: The toxins annotated and identified are typical except LIPA (lipase) - is this a known protein family expressed in snake venom (secretory protein?) Please explain how or why LIPA is included as part of the venom toxin genes.5. In discussion or conclusion - Suggest to include limitations of the study and the tool developed. In addition, how is the tool maintained and if any updating or curation will be done from time to time.

Level of Interest

Please indicate how interesting you found the manuscript: Choose an item.

Quality of Written English

Please indicate the quality of language in the manuscript: Choose an item.

Declaration of Competing Interests

Please complete a declaration of competing interests, considering the following questions:

- Have you in the past five years received reimbursements, fees, funding, or salary from an organisation that may in any way gain or lose financially from the publication of this manuscript, either now or in the future?
- Do you hold any stocks or shares in an organisation that may in any way gain or lose financially from the publication of this manuscript, either now or in the future?
- Do you hold or are you currently applying for any patents relating to the content of the manuscript?
- Have you received reimbursements, fees, funding, or salary from an organization that holds or has applied for patents relating to the content of the manuscript?
- Do you have any other financial competing interests?
- Do you have any non-financial competing interests in relation to this paper?

If you can answer no to all of the above, write 'I declare that I have no competing interests' below. If your reply is yes to any, please give details below.

I declare that I have no competing interests.

I agree to the open peer review policy of the journal. I understand that my name will be included on my report to the authors and, if the manuscript is accepted for publication, my named report including any attachments I upload will be posted on the website along with the authors' responses. I agree for my report to be made available under an Open Access Creative Commons CC-BY license (http://creativecommons.org/licenses/by/4.0/). I understand that any comments which I do not wish to be included in my named report can be included as confidential comments to the editors, which will not be published.

Choose an item.

To further support our reviewers, we have joined with Publons, where you can gain additional credit to further highlight your hard work (see: https://publons.com/journal/530/gigascience). On publication of this paper, your review will be automatically added to Publons, you can then choose whether or not to claim your Publons credit. I understand this statement.

Yes Choose an item.
