# Supplementary material for: ToxCodAn-Genome: an automated pipeline for toxin-gene annotation in genome assembly of venomous lineages
Source: Gigascience. 2024 Jan 18;13:giad116. doi: 10.1093/gigascience/giad116 (PMC10797961; doi:10.1093/gigascience/giad116)
Supplement: giad116_Reviewer_2_Report_Revision_1 — Zachary Kenneth Stewart -- 11/2/2023 Reviewed [file giad116_reviewer_2_report_revision_1.pdf]

Reviewer Report

Title: ToxCodAn-Genome: an automated pipeline for toxin-gene annotation in genome assembly of venomous lineages

Version: Revision 1 **Date:** 11/2/2023

Reviewer name: Zachary Kenneth Stewart

Reviewer Comments to Author:

I think the authors have done an excellent job considering all of the reviewer's comments including my own. The additional benchmark of the software's ability to rediscover known toxin annotations is well handled, and clearly indicates to the reader the relative level of performance they can expect from the software. And, I believe the included details in the discussion regarding its requirement for canonical splice signals among other things, provides necessary details to the reader to understand where and why some toxins may not be annotated by ToxCodAn-Genome.I have tested the software briefly and validated that it is compatible with exonerate versions 2.2 and 2.4, and has a new and useful validation function for input FASTA files. It otherwise performs identically to before which is good. It would have been nice to fix the determinism of the program, but I understand that it is due to an external package which would be non-trivial to replace, and that a domain expert should be able to identify when synonymous toxin family names are being used to allow for comparative analyses to take place.I believe this article should be accepted in its current form, and would love to see the program continue with yearly updates as I think it could become a staple tool in this area of research if the software is well maintained.

Level of Interest

Please indicate how interesting you found the manuscript: Choose an item.

Quality of Written English

Please indicate the quality of language in the manuscript: Choose an item.

Declaration of Competing Interests

Please complete a declaration of competing interests, considering the following questions:

- Have you in the past five years received reimbursements, fees, funding, or salary from an organisation that may in any way gain or lose financially from the publication of this manuscript, either now or in the future?
- Do you hold any stocks or shares in an organisation that may in any way gain or lose financially from the publication of this manuscript, either now or in the future?

- Do you hold or are you currently applying for any patents relating to the content of the manuscript?
- Have you received reimbursements, fees, funding, or salary from an organization that holds or has applied for patents relating to the content of the manuscript?
- Do you have any other financial competing interests?
- Do you have any non-financial competing interests in relation to this paper?

If you can answer no to all of the above, write 'I declare that I have no competing interests' below. If your reply is yes to any, please give details below.

I declare that I have no competing interests

I agree to the open peer review policy of the journal. I understand that my name will be included on my report to the authors and, if the manuscript is accepted for publication, my named report including any attachments I upload will be posted on the website along with the authors' responses. I agree for my report to be made available under an Open Access Creative Commons CC-BY license (http://creativecommons.org/licenses/by/4.0/). I understand that any comments which I do not wish to be included in my named report can be included as confidential comments to the editors, which will not be published.

Choose an item.

To further support our reviewers, we have joined with Publons, where you can gain additional credit to further highlight your hard work (see: https://publons.com/journal/530/gigascience). On publication of this paper, your review will be automatically added to Publons, you can then choose whether or not to claim your Publons credit. I understand this statement.

Yes Choose an item.
