# Supplementary material for: ToxCodAn-Genome: an automated pipeline for toxin-gene annotation in genome assembly of venomous lineages
Source: Gigascience. 2024 Jan 18;13:giad116. doi: 10.1093/gigascience/giad116 (PMC10797961; doi:10.1093/gigascience/giad116)
Supplement: giad116_Reviewer_3_Report_Original_Submission — Jason Macrander, Ph. D. -- 9/18/2023 Reviewed [file giad116_reviewer_3_report_original_submission.pdf]

Reviewer Report

Title: ToxCodAn-Genome: an automated pipeline for toxin-gene annotation in genome assembly of venomous lineages

Version: Original Submission **Date:** 9/18/2023

Reviewer name: Jason Macrander, Ph. D.

Reviewer Comments to Author:

First, I want to apologize for the delay in providing my comments/review. The review request arrived the same day the fall semester started and things have been a bit busy. I would have declined the review considering the busy schedule, however, based on the title and abstract I was excited to read this manuscript (and fit it in my schedule as I could).Thankfully I was not disappointed. This manuscript is extremely well written. I have absolutely no suggestions for changes or modifications and think it's acceptable for publication in its current form. ToxCodAn-Genome addresses the challenges of accurately annotating toxin genes in genomic regions and circumvents issues surrounding poorly characterized lineages by using automated genome annotation tools. ToxCodAn-Genome is the first software designed to perform automated toxin annotation in genomes of venomous lineages. The package itself is customizable, allowing users to test different parameters to improve the final toxin annotation set for the studied lineage. The tools are build into the program and allow users to customize several aspects of the annotation procedures, with "warnings" for less than reliable annotaitons. ToxCodAn-Genome circumvents a lot of laborious procedures involved with the manual inspection to identify the complete set of genes. There are many positive aspects of this program and its impact on the field of venomics and the resulting manuscript will no doubt have a positive impact on the field. Many of the existing pipelines rely too much on training datasets resulting in misguided results, or heavily rely on time consuming curation. The software also provides a detailed guide that describes the processes to build a custom toxin database, perform nontoxin annotation, quantify gene expression, and plot the toxin loci for reports and publications. ToxCodAn-Genome also has the potential to facilitate the discovery of new toxin compounds and the development of new medicines, accelerating these fields in areas with the ever growing genomic data.The only feedback I have for manuscript improvement is that the authors should consider including x-axis labels in Figures 2 and 3. It is defined in the figure legend, but a label at the bottom of each A/B side for Fig 2 & 3 may assist in readers interpretations of these figures.Unfortunately I did not have time to test the program myself (due to my schedule limiting my ability to do so, not because of the program or authors). Considering how the past month has been it'd probably be another month before I could find time in my schedule to run it. The github website is setup in a way to guide users through the implementation of the program and seems very straightforward with a user guide that is easy to follow. Although I am not able to identify any shortcomings of the ToxCodAn-Genome software, it is worth noting that the software is designed specifically for venomous lineages, and its performance on non-venomous lineages is unknown (although it has potential for characterizing other gene family types in other genomes (a benefit)). One down side is that poorly studied venomous lineages or atypical venomous organisms may result in a significant number of false

positives or mis-characterized toxins. This shortcoming is not anything the authors could remedy, much like a majority of similar software packages of this type out on the market, the users need to understand the programs own limitations and expectations to use them effectively.

Level of Interest

Please indicate how interesting you found the manuscript: Choose an item.

Quality of Written English

Please indicate the quality of language in the manuscript: Choose an item.

Declaration of Competing Interests

Please complete a declaration of competing interests, considering the following questions:

- Have you in the past five years received reimbursements, fees, funding, or salary from an organisation that may in any way gain or lose financially from the publication of this manuscript, either now or in the future?
- Do you hold any stocks or shares in an organisation that may in any way gain or lose financially from the publication of this manuscript, either now or in the future?
- Do you hold or are you currently applying for any patents relating to the content of the manuscript?
- Have you received reimbursements, fees, funding, or salary from an organization that holds or has applied for patents relating to the content of the manuscript?
- Do you have any other financial competing interests?
- Do you have any non-financial competing interests in relation to this paper?

If you can answer no to all of the above, write 'I declare that I have no competing interests' below. If your reply is yes to any, please give details below.

I declare that I have no competing interests

I agree to the open peer review policy of the journal. I understand that my name will be included on my report to the authors and, if the manuscript is accepted for publication, my named report including any attachments I upload will be posted on the website along with the authors' responses. I agree for my report to be made available under an Open Access Creative Commons CC-BY license (http://creativecommons.org/licenses/by/4.0/). I understand that any comments which I do not wish to be included in my named report can be included as confidential comments to the editors, which will not be published.

Choose an item.

To further support our reviewers, we have joined with Publons, where you can gain additional credit to further highlight your hard work (see: https://publons.com/journal/530/gigascience). On publication of this paper, your review will be automatically added to Publons, you can then choose whether or not to claim your Publons credit. I understand this statement.

Yes Choose an item.
